# A Desaturative Approach for Aromatic Aldehyde Synthesis via Synergistic Enamine, Photoredox and Cobalt Triple Catalysis

**DOI:** 10.1002/anie.202201870

**Published:** 2022-03-09

**Authors:** Huaibo Zhao, Henry P. Caldora, Oliver Turner, James J. Douglas, Daniele Leonori

**Affiliations:** ^1^ Department of Chemistry University of Manchester Oxford Road Manchester M13 9PL UK; ^2^ Oncology R&DI Medicinal Chemistry, AstraZeneca Darwin Building, Unit 310, Cambridge Science Park, Milton Road Cambridge CB4 0WG UK; ^3^ Early Chemical Development, Pharmaceutical Sciences R&D AstraZeneca Macclesfield UK; ^4^ Institute of Organic Chemistry RWTH Aachen University Landoltweg 1 52056 Aachen Germany

**Keywords:** Aldehydes, Cobalt Catalysis, Desaturation, Organocatalysis, Photoredox Catalysis

## Abstract

Aromatic aldehydes are fundamental intermediates that are widely utilised for the synthesis of important materials across the broad spectrum of chemical industries. Accessing highly substituted derivatives can often be difficult as their functionalizations are generally performed via electrophilic aromatic substitution, S_E_Ar. Here we provide an alternative and mechanistically distinct approach whereby aromatic aldehydes are assembled from saturated precursors via a desaturative process. This novel strategy harnesses the high‐fidelity of Diels–Alder cycloadditions to quickly construct multi‐substituted cyclohexenecarbaldehyde cores which undergo desaturation via the synergistic interplay of enamine, photoredox and cobalt triple catalysis.

## Graphical abstract

Synergistic enamine, photoredox and cobalt triple catalysis enables a desaturative approach for the preparation of aromatic aldehydes.

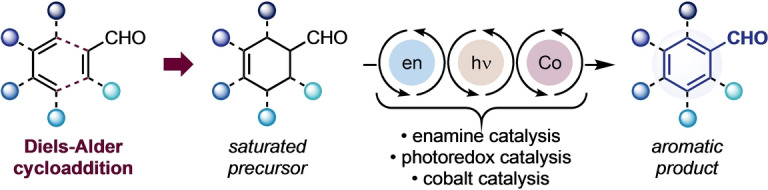

Aromatic aldehydes are integral building blocks in organic chemistry that, through their rich and diverse reactivity profile, provide a handle to access a large number of bond‐forming strategies.^[1]^ This powerful synthetic versatility means they are often encountered as intermediates in the preparation of high‐value materials such as pharmaceuticals and agrochemicals, as well as valuable end‐products in the fragrance and food industries (Scheme 1A).^[2]^

**Scheme 1:**
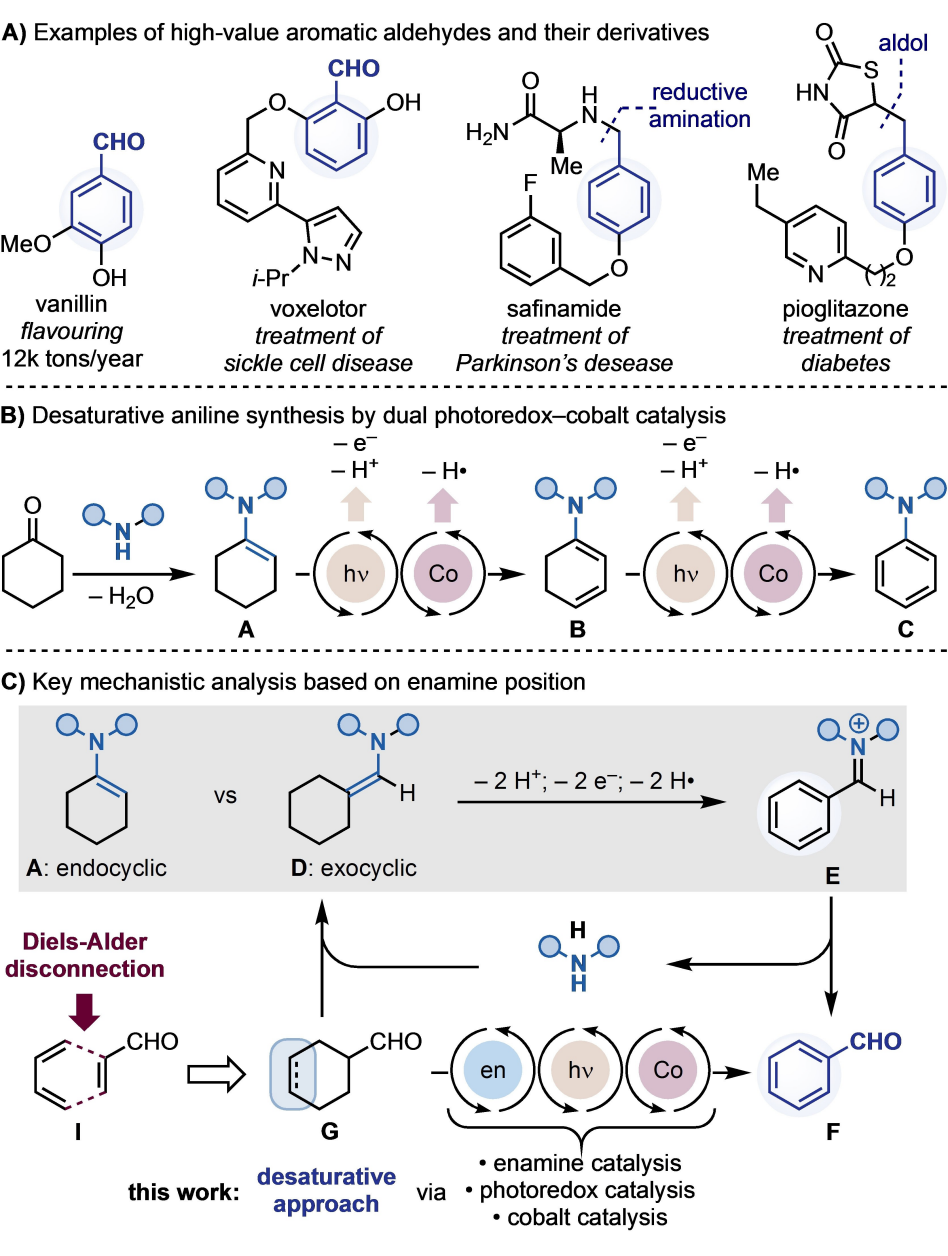
A) Examples of high‐value aromatic aldehydes and their derivatives. B) Desaturative coupling of ketones and aldehydes: key mechanistic blueprint. C) Mechanistic analysis on desaturation chemistry for cyclic enamines.

Despite their synthetic prominence, complex and multi‐functionalised aromatic aldehydes can still be challenging to prepare. Simple derivatives can be accessed by carbonylation (e.g. Vilsmeier–Haack reaction) of electron‐rich arenes or Friedel–Crafts functionalisation of benzaldehyde derivatives.^[3]^ These processes utilise electrophilic intermediates and therefore are restricted to the selectivity rules of electrophilic aromatic substitution (S_E_Ar) chemistry.^[4]^ As an example, Friedel–Crafts functionalization of benzaldehydes usually delivers the *meta* products leaving unreacted the *ortho* and *para* positions.^[5]^ A common alternative approach is firstly aromatic halogenation, followed by either Li/Mg‐exchange (and quench with DMF or other electrophilic “CHO” synthons)^[6]^ or Pd‐catalysed carbonylation, to introduce the aldehyde functionality.^[7]^ However, these methods still require a prior S_E_Ar reaction to introduce the halogen, which poses related selectivity issues to the ones discussed above.^[8]^ Furthermore, the use of pyrophoric organometallic reagents as well as the potentially explosive syngas at high temperatures and pressures can further limit applications. ^[9]^

We recently became interested in the possibility of assembling substituted benzaldehydes from non‐aromatic precursors via a mechanistically distinct approach.^[10]^ Our group has previously reported the utilisation of dual photoredox‐cobalt catalysis^[11]^ for the preparation of anilines from cyclohexanone and amine building blocks (Scheme 1B).[^12^, ^13^] The key aspect of this reactivity mode is the formation of a redox‐active enamine **A** that, through two sequences of SET oxidation→deprotonation→dehydrogenation (formal removal of 2 H^+^, 2 e^−^ and 2 H⋅), is converted first into a dienamine **B** and then into a stable aromatic material **C**.

In this prior approach, the enamine functionality is a constituent of the cyclohexene ring (i.e. endocyclic enamine **A**) and therefore its C−N bond is incorporated in the final aromatic product **C**. We questioned whether this desaturative approach could be translated onto an exocyclic enamine **D** as the formal removal of 2 H^+^, 2 e^−^ and 2 H⋅ would lead to an aromatic iminium ion **E** that could be hydrolysed to furnish benzaldehyde **F** (Scheme 1C). This proposal revealed the opportunity of exploiting the transient condensation between a cyclohexanecarbaldehyde **G** and a secondary amine organocatalyst **H** as a stepping stone into redox chemistry for subsequent desaturation. However, cyclohexanecarbaldehydes are somewhat difficult‐to‐make with limited opportunity for site‐selective functionalization. Hence, we realised that unsaturation between the cyclohexyl C3 and C4 positions would be ideal for our purposes, providing Diels–Alder cycloaddition (**I**) as a retrosynthetic tactic.^[14]^ Indeed, this pericyclic reactivity could be used to facilitate the selective and straightforward introduction of substituents around the “future” benzaldehyde aromatic ring, bypassing the aforementioned drawbacks of S_E_Ar. Herein, we describe the implementation of this proposal and introduce a novel strategy for aromatic aldehyde synthesis through the synergistic integration of enamine, photoredox and cobalt catalysis.

The proposed mechanism for this desaturative strategy is depicted in Scheme 2A. Condensation between aldehyde **1** and secondary amine **H** initiates the organocatalytic cycle by the formation of exocyclic enamine **D**. In parallel, an oxidative quenching photoredox manifold would be used to enable single‐electron transfer (SET) between a visible‐light excited [Ir^III^] photocatalyst [for Ir(dtbbpy)(ppy)_2_PF_6_: *E_1/2_(IV/III)=−0.96 V vs. SCE]^[15]^ and a [Co^III^] cobaloxime [for Co(dmgH)_2_(Py)Cl: *E*_1/2_^red^=−0.32 V versus SCE].^[16]^ The subsequently formed [Ir^IV^] species [*E*_1/2_(IV/III)=+1.21 V vs. SCE]^[15]^ ought to be able to engage in a second SET event with **D** (for **3**: *E*_1/2_^ox^=+0.55 V vs. SCE)^[16]^ to regenerate the ground state [Ir^III^] and give **J** (via deprotonation of the intermediate enamine radical cation, not shown).^[17]^ A H‐atom transfer (HAT) reaction between the 17 e^−^ [Co^II^] metalloradical and **J** would set the first desaturation furnishing the trienamine **K** and a putative [Co^III^]−H.^[18]^ In the presence of a weak Brønsted acid, [Co^III^]−H can evolve H_2_, regenerating [Co^III^].^[19]^ **K** could now be subjected to another round of photoredox oxidation, to give **L**, and Co‐mediated desaturation to give iminium **E**. Hydrolysis of this species would close the organocatalytic cycle and yield the desired benzaldehyde product **2**.

**Scheme 2:**
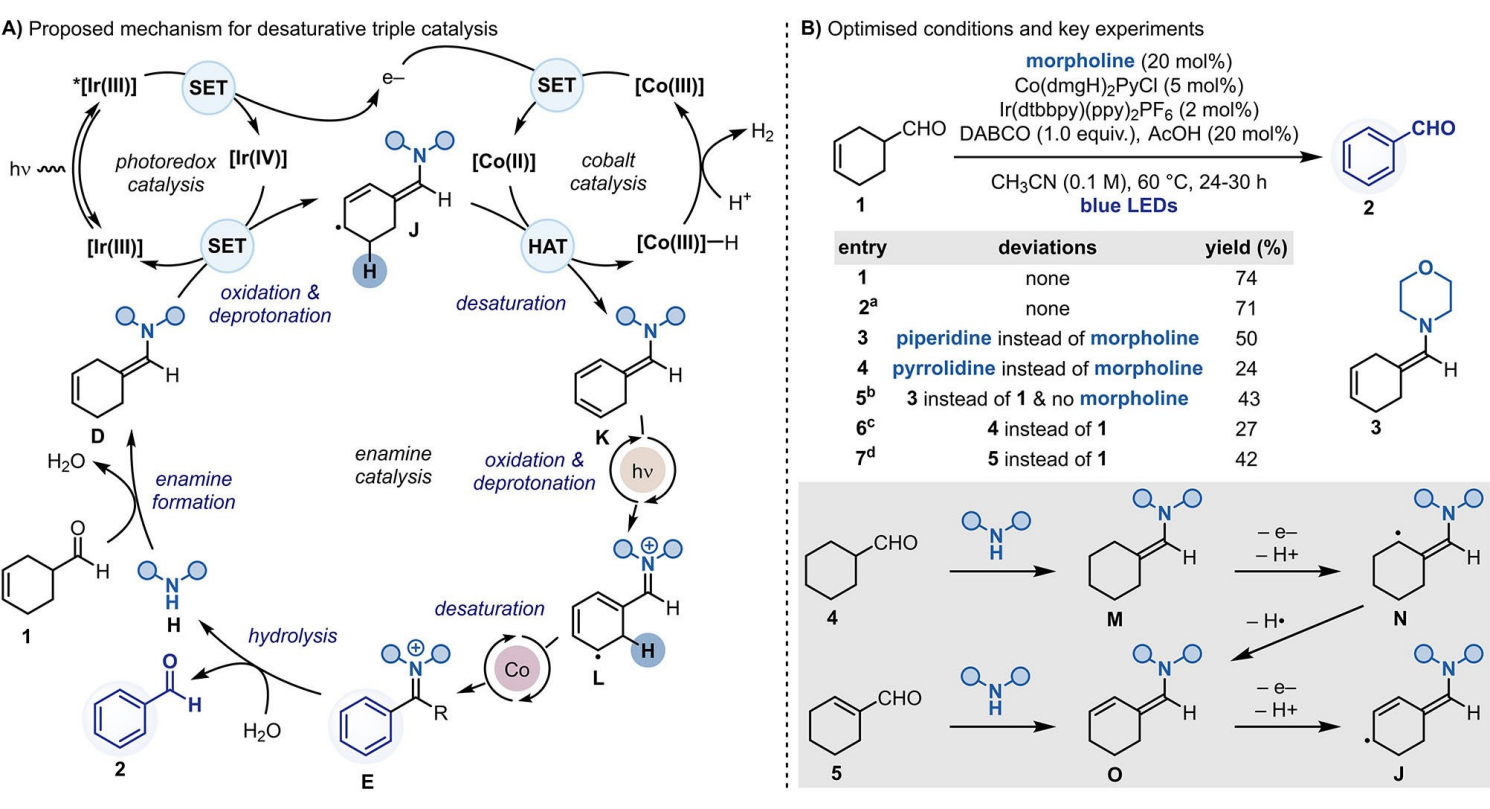
A) Proposed synergistic enamine‐photoredox‐cobalt triple catalysis for the desaturation of **1** to **2**. B) Optimized conditions for the desaturation of **1** to **2** and relevant control reactions. [a] Reaction run at AstraZeneca. [b] Reaction run at r.t. [c] Reaction run with 1.2 equiv of morpholine and 3.0 equiv of DABCO. [d] Reaction run with 1.2 equiv of morpholine.

As shown in Scheme 2B, this proposal for desaturative aldehyde synthesis was first implemented by treatment of **1** with morpholine (20 mol %) as the organocatalyst, Ir(dtbbpy)(ppy)_2_PF_6_ (2 mol %) as the photocatalyst and Co(dmgH)_2_PyCl (5 mol %) as the cobaloxime. The use of this tri‐catalytic system under buffered conditions [DABCO (1.0 equiv)+AcOH (20 mol %)] in CH_3_CN solvent and blue LED irradiation at 60 °C gave **2** in a 74 % (entry 1). These conditions were successfully reproduced within the laboratory at AstraZeneca in a comparable yield of 71 %, using a Lucent360 photochemical reactor (entry 2). A yield of 60 % was observed after only 3 h, likely resulting from the high photon‐flux of the Lucent360 reactor.^[16]^ Whilst the Supporting Information encompasses all the optimisation and the control experiments, it is worthwhile highlighting that the structure of the organocatalyst had a large impact on the success of the reactivity. Indeed, performing the desaturations with piperidine or pyrrolidine (as well as other amines), in place of morpholine, led to significantly lower yields (entries 3 and 4). To further support the proposed mechanism for this process, we ran cyclic voltammetry and performed Stern–Volmer luminescence quenching experiments that support the proposed sequence of SET events in the photoredox cycle.^[16]^ Good reactivity could also be achieved on the pre‐formed enamine **3** (entry 5),^[16]^ which enabled clear detection of H_2_ by ^1^H NMR spectroscopy.

While cyclohex‐3‐enecarbaldehydes such as **1** are the most versatile class of building blocks for scope evaluation, we were interested to see if other types of precursors could be engaged in this reactivity. Pleasingly, both Cy‐CHO **4** and α,β‐unsaturated **5** led to productive reactivity, albeit in lower yields (entries 6 and 7). The successful formation of **2** from **4** and **5** suggests that enamines **M** and **O** are also useful intermediates in providing access to **J** for subsequent desaturation.

With the optimised conditions in hand, we next looked to evaluate the scope of our desaturative methodology in the synthesis of poly‐substituted aromatic aldehydes (Scheme 3). We started by exploiting the Diels–Alder reaction between 2,3‐dimethyl‐1,2‐butadiene and several β‐substituted enals as entry into 2,4,5‐trisubstituted aldehydes,^[16]^ which are often used in the preparation of bioactive materials.^[20]^ These saturated derivatives enabled the synthesis of benzaldehyde derivatives with *ortho* alkyl (**6**–**9**), aryl (**10**, **12** and **13**) and ester (**14**) substituents in high yields. The complementarity that this strategy might provide to approaches based on standard aromatic chemistry, can be realised considering **10**. Despite its structural simplicity, this building block requires a seven steps synthesis from **11**,^[21]^ which can now be streamlined to just two steps by our approach (Diels–Alder cycloaddition followed by desaturation).

**Scheme 3:**
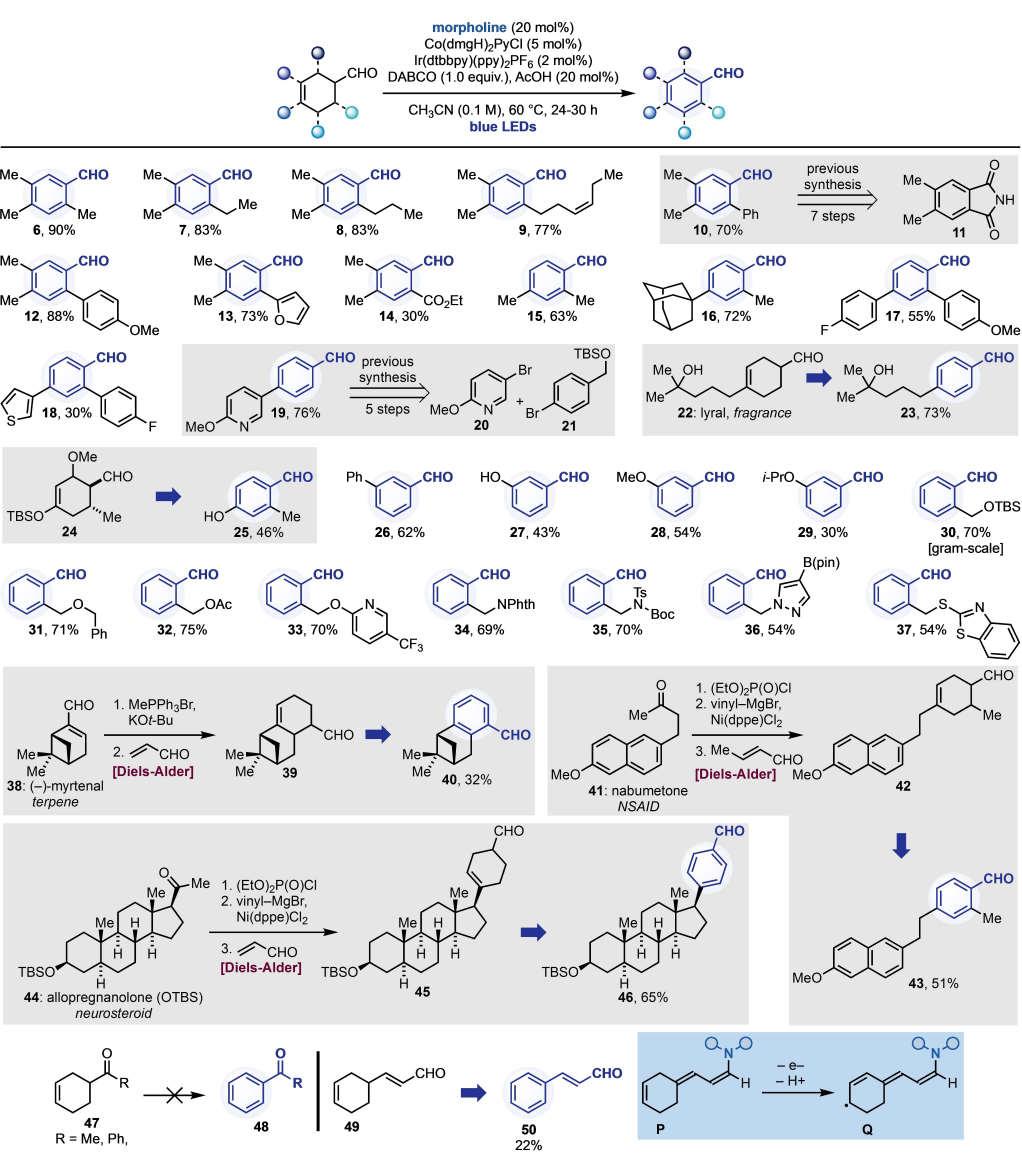
Substrate scope for the synergistic enamine‐photoredox‐cobalt catalytic desaturations.

We then evaluated the use of other substrates prepared by cycloaddition of 2‐substituted butadienes with various enals,^[16]^ which enabled the selective assembly of 2,4‐disubstituted benzaldehydes. These examples are represented by products with two alkyl substituents (**15** and **16**) as well as derivatives with two aryl groups (**17** and **18**). Derivative **19** has been recently prepared by Taisho Pharmaceutical (Japan) and Sanofi (EU) as part of a MedChem program and required a 5 steps synthesis from **20** and **21** via the formation of organozinc intermediates.^[22]^ Our alternative strategy avoids the use of such reagents, highlighting the potential synthetic impact of this work. Compound **23** was obtained by direct desaturation of the fragrance compound lyral **22**, which also demonstrates that a tertiary alcohol functionality was tolerated.

Danishefsky's diene is one of the most exploited building blocks in Diels–Alder chemistry and its corresponding cycloadduct **24** would be a convenient precursor for the preparation of *ortho*,*para*‐dihydroxylated aldehydes.^[23]^ Subjecting **24** to our desaturative conditions was possible but led to the competing formation of **25**. We believe that upon enamine formation, a fast E1cB elimination takes place removing the *ortho* OMe group, followed by dual photoredox‐cobalt desaturation.^[24]^

To further explore the types of functionalisation patterns amenable to this strategy, we looked at *meta*‐functionalised aldehydes that can be difficult to access by standard S_E_Ar chemistry. Pleasingly, desaturation enabled the preparation of **26**–**29** in good to moderate yields. **27**–**29** represent a challenging class of derivatives since *meta*‐carbonylation of electron‐rich phenols is disfavoured, whilst *meta*‐oxygenation of aldehydes is possible but usually requires prior nitration.^[25]^

Next, we looked at *ortho*‐substituted derivatives and by exploiting the simple functionalization chemistry of tetrahydrophtalic anhydride^[16]^ we prepared **30**–**37** (also on gram‐scale) that are frequently encountered in the patented literature on drug development. Importantly, these derivatives demonstrated compatibility of several O‐, N‐ and S‐based groups as well as HAT‐labile positions^[26]^ (**31**) and the B(pin) functionality (**36**).

To further probe the chemistry on bioactive templates, we took the terpene (−)‐myrtenal (**38**), the NSAID nabumetone (**41**) and the OTBS‐protected neurosteroid allopregnanolone (**44**) and converted them into the corresponding dienes (1–3 steps).^[27]^ Subsequent Diels–Alder cycloaddition (**39**, **42** and **45**) and catalytic desaturation provided access to **40**, **43** and **46** in moderate to good yields.

In terms of limitations, this approach could not be extended to the desaturation of cyclohexene ketones **47** to **48**. We currently believe that the more challenging enamine formation does not enable integration with photoredox and cobalt catalysis.^[28]^ However, we pleasingly succeeded in engaging vinylogous aldehyde **49** to obtain cinnamaldehyde **50** in moderate yield. We believe this example demonstrates that upon dienamine **P** formation, photoredox oxidation leads to the extended 9π e^−^ system **Q**, from which cobalt‐mediated desaturation takes place.

In conclusion, we have demonstrated an alternative approach for the preparation of substituted aromatic aldehydes from easily accessible saturated cyclohexanecarbaldehyde precursors. This strategy exploits the synergistic interplay of three catalytic manifolds, that sequentially activate the aldehyde (organocatalysis), oxidise the corresponding enamine (photoredox catalysis) and perform desaturation (cobalt catalysis). Since cyclohexenecarbaldehydes can be conveniently prepared by Diels–Alder reactivity, we hope that this strategy might become a complementary tool in the preparation of high‐value aldehyde materials.

## Conflict of interest

The authors declare no conflict of interest.

## Supporting information

As a service to our authors and readers, this journal provides supporting information supplied by the authors. Such materials are peer reviewed and may be re‐organized for online delivery, but are not copy‐edited or typeset. Technical support issues arising from supporting information (other than missing files) should be addressed to the authors.

Supporting Information

## Data Availability

The data that support the findings of this study are available from the corresponding author upon reasonable request.
