# Supplementary material for: A Desaturative Approach for Aromatic Aldehyde Synthesis via Synergistic Enamine, Photoredox and Cobalt Triple Catalysis
Source: Angew Chem Int Ed Engl. 2022 Mar 9;61(18):e202201870. doi: 10.1002/anie.202201870 (PMC9311220; doi:10.1002/anie.202201870)
Supplement: Supplementary file 1 — Supporting Information [file ANIE-61-0-s001.pdf]

Angewandte Chemie
Eine Zeitschrift der Gesellschaft Deutscher Chemiker

Supporting Information

A Desaturative Approach for Aromatic Aldehyde Synthesis via Synergistic Enamine, Photoredox and Cobalt Triple Catalysis

*H. Zhao, H. P. Caldora, O. Turner, J. J. Douglas, D. Leonori**

Table of Contents

1 General Experimental Details 2

2 Starting Material Synthesis 3

3 Reaction Optimizations 48

4 Picture of Reaction Set-Up 52

5 Reaction Performed at AstraZeneca 53

6 Substrate Scope 54

7 Mechanistic Considerations 66

7.1 Cyclic Voltammetry and Emission Quenching Studies 66

7.2 Reaction on Preformed Enamine 69

7.3 Detection of H_2 70

7.4 Quantum Yields (Φ) Determination 72

8 NMR Spectra 73

9 References 145

1 General Experimental Details

All required fine chemicals were used directly without purification unless stated otherwise. All air and moisture sensitive reactions were carried out under nitrogen atmosphere using standard Schlenk manifold technique. All solvents were bought from Acros as 99.8% purity. ^{1}H and ^{13}C Nuclear Magnetic Resonance (NMR) spectra were acquired at various field strengths as indicated and were referenced to $CDCl_3$ (7.26 and 77.2 ppm for ^{1}H and ^{13}C respectively). ^{1}H NMR coupling constants are reported in Hertz and refer to apparent multiplicities and not true coupling constants. Data are reported as follows: chemical shift, integration, multiplicity (s = singlet, br s = broad singlet, d = doublet, t = triplet, q = quartet, qi = quintet, sx = sextet, sp = septet, m = multiplet, dd = doublet of doublets, etc.), proton assignment (determined by 2D NMR experiments: NOESY, HSQC and HMBC) where possible. High-resolution mass spectra were obtained using a JEOL JMS-700 spectrometer or a Fissions VG Trio 2000 quadrupole mass spectrometer. Spectra were obtained using electron impact ionization (EI) and chemical ionization (CI) techniques, or positive electrospray (ES). Analytical TLC: aluminum backed plates pre-coated (0.25 mm) with Merck Silica Gel 60 F254. Compounds were visualized by exposure to UV-light or by dipping the plates in permanganate ($KMnO_4$) stain followed by heating. Flash column chromatography was performed using Merck Silica Gel 60 (40–63 μm). All mixed solvent eluents are reported as v/v solutions. Absorption and emission spectra were obtained using a Horiba Duetta spectrometer and 1 mm High Precision Cell made of quartz from Hellma Analytics. The LEDs used are Kessil PR 160 440 nm. All the reactions were conducted in CEM 10 mL glass microwave tubes.

2 Starting Material Synthesis

GP1 – General Procedure for Diels-Alder Cycloadditions

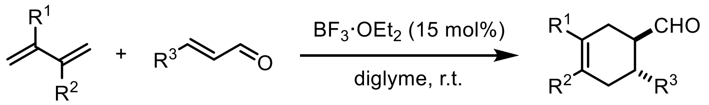

An oven-dried flask was charged with diglyme (5 mL, 1.0 M), the diene (5.0 mmol, 1.0 equiv.) and the dienophile (7.5 mmol, 1.5 equiv.), followed by $BF_3 \cdot OEt_2$ (92 µL, 0.75 mmol, 15 mol%) at room temperature. The reaction was stirred at room temperature for 6–24 h and then was transferred to a separatory funnel containing saturated aqueous $NaHCO_3$ (10 mL) and pentane (10 mL). The phases were separated, and the organic phase was washed with saturated $NaHCO_3$ (10 mL) and water (3 × 10 mL). The combined organic layers were dried (Na_2SO_4), filtered and evaporated. The crude was purified by column chromatography on silica gel eluting with petrol–EtOAc.

GP2 – General Procedure for Preparation of Enol Phosphates

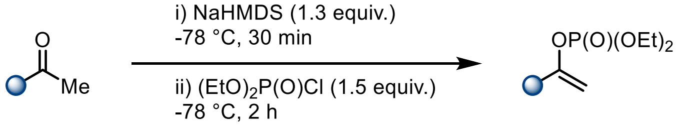

A dry flask was charged with the ketone (1.0 equiv.), dry THF (0.27 M) and cooled to –78 ºC. NaHMDS (1.3 equiv., 2M solution in THF) was added dropwise. The solution was allowed to stir for 30 mins at which point $(EtO)_2P(O)Cl$ (1.5 equiv.) was added dropwise and the reaction mixture was stirred at –78 °C for an additional 2 h. The mixture was allowed to warm up to room temperature and stirred for 15 mins. The reaction was quenched with saturated aqueous NH_4Cl (30 mL) and then extracted with ethyl acetate (3 x 30 mL). The combined organic layers were dried ($MgSO_4$), filtered, and evaporated. The crude was purified by flash chromatography on silica gel eluting with petrol–EtOAc.

GP3 – General Procedure for Nickel Catalysed Kumada Coupling of Enol Phosphates

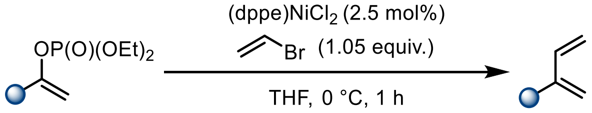

A dry flask was charged with [(dppe)$NiCl_2$] (2.5 mol%), dry THF (0.73 M) and degassed for 20 mins with a stream of argon. The heterogeneous suspension was then cooled to 0 °C and the enol phosphate (1.0 equiv.) was added followed by the dropwise addition of vinyl

magnesium bromide (1.05 equiv., 1 M in THF). The ice bath was removed, and the reaction mixture was stirred for 1 h at room temperature. The reaction was quenched by addition of saturated aqueous NH_4Cl (30 mL) at 0 °C and extracted with Et_2O (3 x 30 mL). The combined organic layers were dried ($MgSO_4$), filtered and evaporated. The crude was purified by column chromatography on silica gel eluting with petrol–EtOAc.

GP4 – General Procedure for $LiAlH_4$ Reduction

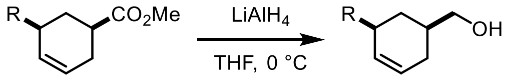

The ester (1.0 equiv.) was slowly added to a slurry of $LiAlH_4$ (1.2 equiv.) in dry THF (0.2 M) at 0 °C and the reaction mixture was stirred at the same temperature until TLC analysis indicated its complete consumption (ca. 1–3 h). The reaction was diluted with Et_2O (20 mL), quenched by successive addition of H_2O, NaOH (15% aq.). The suspension was warmed to room temperature for 15 mins and $MgSO_4$ was added. After 10 mins, the solid were removed by filtration through a pad of celite, and the solid was rinsed with EtOAc. The filtrate was evaporated and purified by flash column chromatography on silica gel eluting with petrol–EtOAc.

GP5 – General Procedure for Swern Oxidation

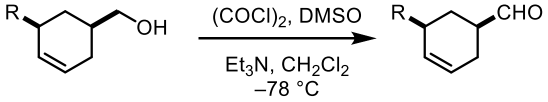

DMSO (3.0 equiv.) was added to a stirred solution of oxalyl chloride (1.5 equiv.) in dry CH_2Cl_2 (0.2 M) at −78 °C and the mixture was stirred at the same temperature for 15 mins. The alcohol (1.0 equiv.) in dry CH_2Cl_2 (2 mL, 0.5 M) was added and the mixture was stirred at −78 °C for 45 mins. Et_3N (6.0 equiv.) was added, and the mixture was gradually warmed to room temperature during 1 h. H_2O (10 mL) was added, and the layers were separated. The aqueous layer was extracted with CH_2Cl_2 (2 x 10 mL) and the combined organic layers were washed with brine (10 mL), dried (Na_2SO_4), filtered and evaporated. The crude was purified by column chromatography on silica gel eluting with petrol–EtOAc.

GP6 – General Procedure for Oxidation with Dess–Martin's Periodinane

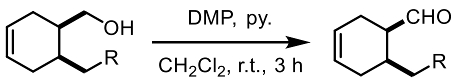

Pyridine (10.0 equiv.) and Dess–Martin periodinane (3.0 equiv.) were added to a stirred solution of alcohol (1.0 equiv.) in dry CH_2Cl_2 (0.25 M) at 0 °C and the reaction mixture was stirred at room temperature until TLC analysis indicated that the reaction was complete (ca. 1–3 h). The mixture was diluted with CH_2Cl_2 (10 mL) and washed successively with $Na_2S_2O_3$ (10% aq., 10 mL) and saturated aqueous $NaHCO_3$ (10 mL). The layers were separated, and the aqueous layers were extracted with CH_2Cl_2 (3 × 20 mL). The combined organic layers were washed with brine (15 mL), dried (Na_2SO_4), filtered and evaporated. The crude was purified by column chromatography on silica gel eluting with petrol–EtOAc.

GP7 – General Procedure for Mitsunobu Reaction

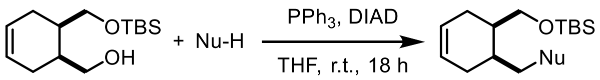

Diisopropyl azodicarboxylate (DIAD) (1.2 equiv.) was added dropwise to a stirred solution of the alcohol (1.0 equiv.), the nucleophile (1.2 equiv) and PPh_3 (1.2 equiv) in dry THF (0.2 M) at 0 °C. The reaction mixture was stirred at room temperature until TLC analysis indicated that the reaction was complete, and the solvent was evaporated. The crude was purified by flash column chromatography on silica gel eluting with petrol–EtOAc.

GP8 – General Procedure for CSA Deprotection

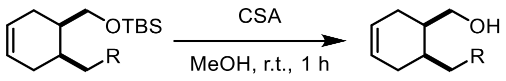

CSA (0.1 equiv.) was added to a stirred solution of TBS-protected alcohol (1.0 equiv.) in MeOH (0.25 M) at room temperature. The reaction mixture was stirred at room temperature until TLC monitoring showed no starting material left (ca. 1 h). Then the solvent was evaporated, and the crude was purified by flash column chromatography on silica gel eluting with petrol–EtOAc.

***trans*-3,4,6-Trimethyl-3-cyclohexene-1-carbaldehyde (6s)**

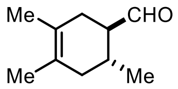

Following **GP1**, 2,3-dimethyl-1,3-butadiene (0.56 mL, 5.0 mmol, 1.0 equiv.) and crotonaldehyde (0.62 mL, 7.5 mmol, 1.5 equiv.) gave **6s** (500 mg, 60%) as an oil. ^{1}H NMR (400 MHz, $CDCl_3$) δ 9.60 (1H, d, *J* = 3.1 Hz), 2.24–2.09 (2H, m), 2.08–1.91 (3H, m), 1.76–1.65 (1H, m), 1.63 (3H, s), 1.59 (3H, s), 0.99 (3H, d, *J* = 6.5 Hz); ^{13}C NMR (101 MHz, $CDCl_3$) δ 205.5, 125.3, 122.8, 53.7, 39.1, 30.5, 28.9, 19.7, 19.0, 18.8. Data in accordance with the literature.[1]

***trans*-6-Ethyl-3,4-dimethyl-3-cyclohexene-1-carbaldehyde (7s)**

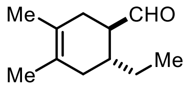

Following **GP1**, 2,3-dimethyl-1,3-butadiene (0.56 mL, 5.0 mmol, 1.0 equiv.) and *trans*-2-pentenal (0.73 mL, 7.5 mmol, 1.5 equiv.) gave **7s** (720 mg, 87%) as an oil. R_f 0.45 [petrol–EtOAc (20:1)]; ^{1}H NMR (400 MHz, $CDCl_3$) δ 9.60 (1H, d, *J* = 3.1 Hz), 2.27 (1H, tdd, *J* = 8.4, 5.7, 3.1 Hz), 2.18 (1H, dd, *J* = 17.4, 8.0 Hz), 2.07 (1H, dd, *J* = 17.4, 5.4 Hz), 1.96 (1H, dd, *J* = 16.6, 4.3 Hz), 1.92–1.82 (1H, m), 1.72–1.64 (1H, m), 1.62 (3H, s), 1.59 (3H, s), 1.52–1.40 (1H, m), 1.32–1.18 (1H, m), 0.90 (3H, t, *J* = 7.4 Hz); ^{13}C NMR (101 MHz, $CDCl_3$) δ 205.6, 125.0, 122.7, 51.6, 35.2, 35.1, 29.9, 26.6, 19.2, 18.8, 11.3; HRMS (ASAP): Found MH^+ 167.1426, $C_{11}H_{19}O$ requires 167.1430.

***trans*-3,4-Dimethyl-6-propyl-3-cyclohexene-1-carbaldehyde (8s)**

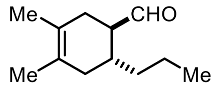

Following **GP1**, 2,3-dimethyl-1,3-butadiene (0.56 mL, 5.0 mmol, 1.0 equiv.) and *trans*-2-hexenal (0.87 mL, 7.5 mmol, 1.5 equiv.) gave **8s** (790 mg, 88%) as an oil. R_f 0.45 [petrol–EtOAc (20:1)]; ^{1}H NMR (400 MHz, $CDCl_3$) δ 9.57 (1H, d, *J* = 3.1 Hz), 2.24 (1H, tdd, *J* = 8.1, 5.4, 3.1 Hz), 2.21–2.12 (1H, m), 2.07 (1H, dd, *J* = 17.7, 5.4 Hz), 2.01–1.89 (2H, m), 1.78–1.63 (1H, m), 1.61 (3H, s), 1.57 (3H, s), 1.44–1.16 (4H, m), 0.87 (3H, t, *J* = 6.7 Hz); ^{13}C NMR (101 MHz, $CDCl_3$) δ 205.5, 125.0, 122.6, 51.9, 36.3, 35.6, 33.2, 29.9, 20.0, 19.1, 18.8, 14.3; HRMS (ASAP): Found MH^+ 181.1581, $C_{12}H_{21}O$ requires 181.1587.

***trans*-6-((*Z*)-Hex-3-en-1-yl)-3,4-dimethyl-3-cyclohexene-1-carbaldehyde (9s)**

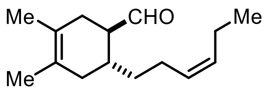

Following **GP1**, 2,3-dimethyl-1,3-butadiene (0.34 mL, 3.0 mmol, 1.2 equiv.) and *trans,cis*-2,6-nonadienal (0.40 mL, 2.5 mmol, 1.0 equiv.) gave **9s** (170 mg, 31%) as an oil. R_f 0.45 [petrol–EtOAc (20:1)]; ^{1}H NMR (400 MHz, $CDCl_3$) δ 9.58 (1H, d, *J* = 3.1 Hz), 5.41–5.31 (1H, m), 5.31–5.23 (1H, m), 2.27 (1H, tdd, *J* = 8.1, 5.6, 3.1 Hz), 2.24–2.14 (1H, m), 2.13–1.94 (7H, m), 1.73–1.65 (1H, m), 1.62 (3H, s), 1.59 (3H, s), 1.50–1.40 (1H, m), 1.35–1.24 (1H, m), 0.94 (3H, t, *J* = 7.5 Hz); ^{13}C NMR (101 MHz, $CDCl_3$) δ 205.4, 132.2, 128.5, 124.9, 122.7, 51.8, 35.4, 34.0, 32.9, 29.8, 24.4, 20.7, 19.2, 18.9, 14.4; HRMS (ASAP): Found MH^+ 221.1898, $C_{15}H_{25}O$ requires 221.1900.

***trans*-3,4,-Dimethyl-6-phenyl-3-cyclohexene-1-carbaldehyde (10s)**

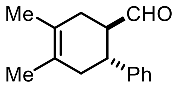

Following **GP1**, 2,3-dimethyl-1,3-butadiene (0.56 mL, 5.0 mmol, 1.0 equiv.) and *trans*-cinnamaldehyde (0.95 mL, 7.5 mmol, 1.5 equiv.) gave **10s** (700 mg, 74%) as an oil. ^{1}H NMR (500 MHz, $CDCl_3$) δ 9.48 (1H, d, *J* = 2.9 Hz), 7.34–7.30 (2H, m), 7.24–7.21 (3H, m), 3.10 (1H, ddd, *J* = 10.3, 8.8, 6.4 Hz), 2.82 (1H, tdd, *J* = 10.3, 5.6, 2.9 Hz), 2.35–2.22 (3H, m), 2.09 (1H, dd, *J* = 17.4, 5.2 Hz), 1.72 (3H, s), 1.68 (3H, s); ^{13}C NMR (126 MHz, $CDCl_3$) δ 204.5, 143.6, 128.8, 127.5, 126.8, 125.7, 123.5, 52.1, 41.5, 39.6, 31.1, 18.9 (2 x C). Data in accordance with the literature.[1]

***trans*-4'-Methoxy-4,5-dimethyl-1,2,3,6-tetrahydro-[1,1'-biphenyl]-2-carbaldehyde (12s)**

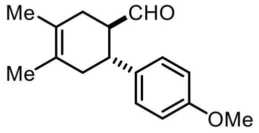

Following **GP1**, 2,3-dimethyl-1,3-butadiene (0.56 mL, 5.0 mmol, 1.0 equiv.) and *trans*-*p*-methoxycinnamaldehyde (1.2 g, 7.5 mmol, 1.5 equiv.) gave **12s** (410 mg, 34%) as an oil. R_f 0.29 [petrol–EtOAc (8:1)]; ^{1}H NMR (400 MHz, $CDCl_3$) δ 9.45 (1H, d, *J* = 3.1 Hz), 7.12 (2H, d, *J* = 8.7 Hz), 6.85 (2H, d, *J* = 8.7 Hz), 3.78 (3H, s), 3.03 (1H, ddd, *J* = 10.5, 9.0, 6.4 Hz), 2.80–2.68 (1H, m), 2.35–2.16 (3H, m), 2.06 (1H, dd, *J* = 17.2, 4.9 Hz), 1.69 (3H, s), 1.65 (3H, s); ^{13}C NMR (101 MHz, $CDCl_3$) δ 204.8, 158.4, 135.6, 128.5, 125.8, 123.4, 114.2, 55.3, 52.4, 40.8, 39.9, 31.3, 18.9 (2 x C); HRMS (ESI): Found MNa^+ 267.1347, $C_{16}H_{20}O_2Na$ requires 267.1356.

cis-6-(2-Furyl)-3,4-dimethyl-3-cyclohexene-1-carbaldehyde (13s)

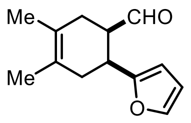

Following **GP1**, 2,3-dimethyl-1,3-butadiene (0.56 mL, 5.0 mmol, 1.0 equiv.) and *cis*-3-(2-furyl)acrolein (916 mg, 7.5 mmol, 1.5 equiv.) gave **13s** (180 mg, 18%) as an oil. R_f 0.45 [petrol–EtOAc (20:1)]; ^{1}H NMR (400 MHz, $CDCl_3$) δ 9.57 (1H, d, *J* = 2.7 Hz), 7.31 (1H, dd, *J* = 1.8, 0.7 Hz), 6.28 (1H, dd, *J* = 3.2, 1.8 Hz), 6.03 (1H, dt, *J* = 3.2, 0.7 Hz), 3.24 (1H, dt, *J* = 9.8, 7.3 Hz), 2.75 (1H, tdd, *J* = 8.7, 5.9, 2.7 Hz), 2.32–2.21 (3H, m), 2.10–1.99 (1H, m), 1.66 (3H, s), 1.65 (3H, s); ^{13}C NMR (101 MHz, $CDCl_3$) δ 204.0, 156.8, 141.5, 124.8, 123.3, 110.3, 105.4, 50.7, 35.7, 34.4, 30.5, 19.0, 18.9; HRMS (ASAP): Found MH^+ 205.1216, $C_{13}H_{17}O_2$ requires 205.1223.

Ethyl *trans*-6-formyl-3,4-dimethyl-3-cyclohexene-1-carboxylate (14s)

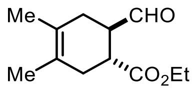

Following **GP1**, 2,3-dimethylbutadiene (1.14 mL, 10.0 mmol, 1.0 equiv.), ethyl *trans*-4-oxo-2-butenoate (1.8 mL, 15.0 mmol, 1.5 equiv.), gave **14s** (1.11 g, 53%) as an oil. ^{1}H NMR (400 MHz, $CDCl_3$) δ 9.70 (1H, d, *J* = 1.4 Hz), 4.16 (2H, q, *J* = 7.1 Hz), 2.94–2.76 (2H, m), 2.30–2.17 (3H, m), 2.11–2.01 (1H, m), 1.65 (3H, s), 1.63 (3H, s), 1.26 (3H, t, *J* = 7.1 Hz). Data in accordance with literature.[2]

Preparation of 16s

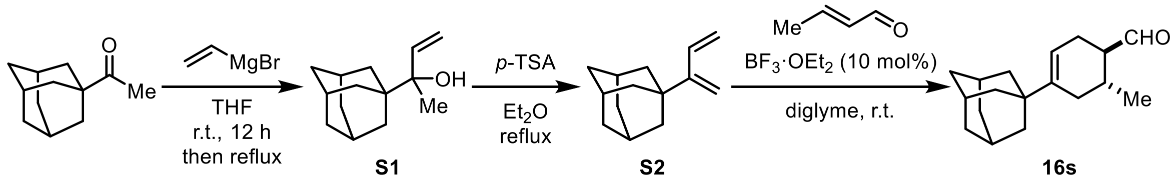

2-(Adamantan-1-yl)but-3-en-2-ol (S1)

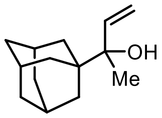

To a flame-dried RBF charged with N_2, 1-(adamantan-1-yl)ethan-1-one (1.78 g, 10.0 mmol, 1.0 equiv.) and THF (10 mL, 1M) were added. The reaction was cooled to 0 ºC and vinyl magnesium bromide in THF (1M, 20 mL, 20.0 mmol, 2.0 equiv.) was added dropwise. The solution was then warmed to r.t and stirred for 12 h overnight. The reaction was then refluxed for 3 h. The reaction was quenched with saturated aqueous NH_4Cl (20 mL) and the aqueous phase was extracted with Et_2O (40 mL). The organic layer was washed with brine (20 mL) and dried over $MgSO_4$. The solvent was removed *in vacuo* to give **S1** (1.94 g, 94%) as an oil that required no further purification. ^{1}H NMR (400 MHz, $CDCl_3$) δ 6.03 (1H, dd, *J* = 17.3, 10.9 Hz), 5.18 (1H, dd, *J* = 17.3, 1.6 Hz), 5.09 (1H, dd, *J* = 10.9, 1.6 Hz), 1.99 (3H, br s), 1.73–1.59 (12H, m), 1.34 (1H, br s), 1.19 (3H, s). Data in accordance with literature.[3]

1-(Buta-1,3-dien-2-yl)adamantine (S2)

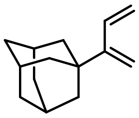

S1 (1.94 g, 9.41 mmol, 1.0 equiv.) and *p*-toluenesulfonic acid monohydrate (1.90 g, 10.0 mmol, 1.1 equiv.) were added to a flame-dried RBF fitted with a condenser and purged with N_2. Et_2O (40 mL, 0.24 M) was added, and the reaction was heated under reflux for 12 h. The reaction was neutralised with sat. $NaHCO_3$ solution (30 mL) and the organic phase was separated and dried over $MgSO_4$. The solvent was removed *in vacuo* and the residue was purified via column chromatography on silica gel eluting with pentane to give **S2** (0.71 g, 40%) as a solid. ^{1}H NMR (400 MHz, $CDCl_3$) δ 6.45 (1H, dd, *J* = 15.6, 10.7 Hz), 5.38 (1H, dd, *J* = 17.0, 2.3 Hz), 5.08 (1H, dd, *J* = 1.7, 1.0 Hz), 5.00 (1H, dd, *J* = 10.7, 2.3 Hz), 4.72 (1H, d, *J* = 1.7 Hz), 2.01 (3H, br s), 1.78–1.61 (12H, m). Data in accordance with literature.[3]

4-Adamantan-1-yl-*trans*-6-methyl-3-cyclohexene-1-carbaldehyde (16s)

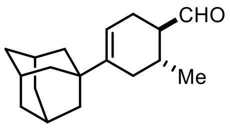

Following **GP1**, **S2** (0.38 g, 2.0 mmol, 1.0 equiv.), *trans*-crotonaldehyde (0.25 mL, 3.0 mmol, 1.5 equiv.), $BF_3 \cdot Et_2O$ (0.025 mL, 10 mol%) gave **16s** (0.27 g, 53%) as a solid. R_f 0.21 [pentane–CH_2Cl_2 (7:3)]; ^{1}H NMR (400 MHz, $CDCl_3$) δ 9.60 (1H, d, *J* = 3.1 Hz), 5.37 (1H, br s), 2.34–2.20 (1H, m), 2.19–2.08 (3H, m), 1.98 (4H, s), 1.74–1.55 (13H, m), 1.01 (3H, d, *J* = 6.6 Hz); ^{13}C NMR (101 MHz, $CDCl_3$) δ 205.6, 145.7, 114.5, 52.9, 40.9, 37.2, 37.0, 30.7, 28.9, 28.8, 24.5, 19.9; HRMS (ESI): Found MNa^+ 281.1868, $C_{18}H_{26}ONa$ requires 281.1881.

Preparation of 17s

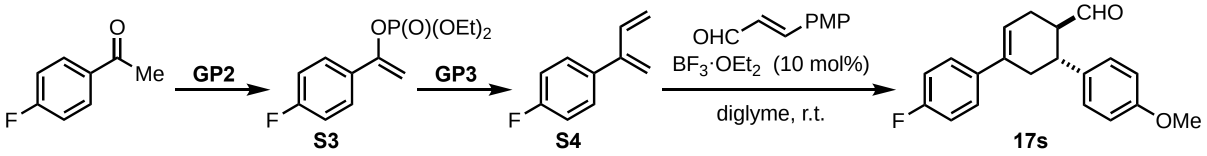

Diethyl (1-(4-fluorophenyl)vinyl) phosphate (S3)

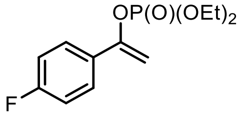

Following **GP2**, 4-fluoroacetopheneone (1.82 mL, 15.0 mmol, 1.0 equiv.), LiHMDS (1M, 20 mL, 20.0 mmol, 1.3 equiv.) and $(EtO)_2P(O)Cl$ (3.26 mL, 22.5 mmol, 1.5 equiv.) gave **S3** (3.55 g, 86%) as an oil. ^{1}H NMR (500 MHz, $CDCl_3$) δ 7.61–7.46 (2H, m), 7.04 (2H, t, *J* = 8.7 Hz), 5.23–5.19 (2H, m), 4.25–4.16 (4H, m), 1.35 (6H, td, *J* = 7.0, 1.1 Hz); ^{19}F NMR (376 MHz, $CDCl_3$) δ –112.19 (1F, tt, *J* = 8.5, 5.3 Hz); ^{31}P NMR (162 MHz, $CDCl_3$) δ –6.15 – –6.44 (1P, m). Data in accordance with literature.[4]

1-(Buta-1,3-dien-2-yl)-4-fluorobenzene (S4)

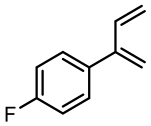

Following **GP3**, **S3** (2.74 g, 10.0 mmol, 1.0 equiv.), $Ni(dppe)Cl_2$ (132 mg, 2.5 mol%) and vinyl magnesium bromide in THF (1 M, 10.5 mL, 10.5 mmol, 1.05 equiv.) gave **S4** (0.87 g, 59%) as an oil. ^{1}H NMR (400 MHz, $CDCl_3$) δ 7.30 (2H, dt, *J* = 6.4, 2.9 Hz), 7.05 (2H, t, *J* = 8.7 Hz), 6.62 (1H, dd, *J* = 17.4, 10.7 Hz), 5.37–5.06 (4H, m); ^{19}F NMR (376 MHz, $CDCl_3$) δ –115.14 (tt, *J* = 8.7, 5.5 Hz). Data in accordance with literature.[5]

4-(4-Fluorophenyl)-*trans*-6-(4-methoxyphenyl)-3-cyclohexene-1-carbaldehyde (17s)

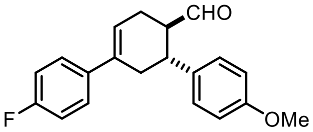

Following **GP1**, **S4** (0.30 g, 2.0 mmol, 1.0 equiv.), *trans*-4-methoxycinnamaldehyde (0.49 g, 3.0 mmol, 1.5 equiv.), $BF_3{\cdot}Et_2O$ (0.025 mL, 10 mol%) gave **17s** (0.23 g, 37%) as an oil. R_f 0.30 [EtOAc–Pentane (15:85)]; ^{1}H NMR (400 MHz, $CDCl_3$) δ 9.53 (1H, d, *J* = 2.8 Hz), 7.34 (2H, dd, *J* = 8.7, 5.5 Hz), 7.19 (2H, d, *J* = 8.6 Hz), 7.00 (2H, t, *J* = 8.7 Hz), 6.88 (2H, d, *J* = 8.7 Hz), 6.16–6.09 (1H, m), 3.80 (3H, s), 3.29–3.15 (1H, m), 2.90–2.79 (1H, m), 2.77–2.68

(1H, m), 2.65–2.47 (2H, m), 2.47–2.37 (1H, m); ^{13}C NMR (101 MHz, $CDCl_3$) δ 204.4, 162.2 (d, J_{C-F} = 246.1 Hz), 158.7, 137.3 (d, J_{C-F} = 3.2 Hz), 135.8, 135.1, 128.6, 126.7 (d, J_{C-F} = 7.8 Hz), 121.6, 115.3 (d, J_{C-F} = 21.4 Hz), 114.5, 55.4, 51.2, 40.6, 35.8, 25.8; ^{19}F NMR (376 MHz, $CDCl_3$) δ –115.6 (tt, *J* = 8.5, 5.3 Hz); HRMS (ESI): Found MNa^+ 333.1246, $C_{20}H_{19}O_2FNa$ requires 333.1267.

Preparation of 18s

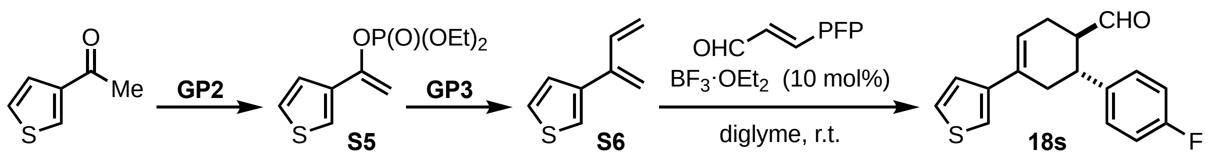

Diethyl (1-(thiophen-3-yl)vinyl) phosphate (S5)

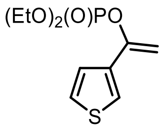

Following **GP2**, 1-(thiophen-3-yl)ethan-1-one (2.53 g, 20.0 mmol, 1.0 equiv.), NaHMDS (2M, 13 mL, 26.0 mmol, 1.3 equiv.) and $(EtO)_2P(O)Cl$ (4.35 mL, 30.0 mmol, 3.0 equiv.) gave **S5** (3.26 g, 62%) as an oil. ^{1}H NMR (400 MHz, $CDCl_3$) δ 7.47 (1H, dd, *J* = 3.1, 1.3 Hz), 7.28 (1H, dd, *J* = 5.1, 3.1 Hz), 7.21 (1H, dd, *J* = 5.1, 1.3 Hz), 5.18–5.11 (2H, m), 4.30–4.12 (4H, m), 1.36 (6H, td, *J* = 7.1, 1.1); ^{31}P NMR (162 MHz, $CDCl_3$) δ –6.23 – –6.49 (1P, m). Data in accordance with literature.[6]

3-(Buta-1,3-dien-2-yl)thiophene (S6)

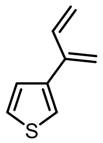

Following **GP3**, **S5** (3.27 g, 12.5 mmol, 1.0 equiv.), Ni(dppe)Cl_2 (186 mg, 2.8 mol%) and vinyl magnesium bromide in THF (1 M, 14.8 mL, 14.8 mmol, 1.2 equiv.) gave **S6** (1.33 g, 68%) as a solid. ^{1}H NMR (400 MHz, $CDCl_3$) δ 7.30 (1H, dd, *J* = 5.0, 3.0 Hz), 7.24 (1H, dd, *J* = 3.0, 1.3 Hz), 7.14 (1H, dd, *J* = 5.0, 1.3 Hz), 6.61 (1H, dd, *J* = 17.4, 10.7 Hz), 5.42–5.33 (1H, m), 5.30–5.26 (2H, m), 5.25–5.20 (1H, m). Data in accordance with literature.[7]

4-(Thiophen-3-yl)-*trans*-6-(4-fluorophenyl)-3-cyclohexene-1-carbaldehyde (18s)

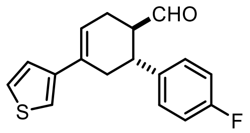

Following **GP1**, **S6** (0.27 g, 2.0 mmol, 1.0 equiv.), *trans*-4-fluorocinnamaldehyde (0.45 g, 3.0 mmol, 1.5 equiv.), $BF_3{\cdot}Et_2O$ (0.025 mL, 10 mol%) gave **18s** (31.1 mg, 5%) as a solid. R_f 0.30 [EtOAc–Pentane (5:95)]; ^{1}H NMR (400 MHz, $CDCl_3$) δ 9.53 (1H, d, *J* = 2.7 Hz), 7.29 (1H, dd, *J* = 5.1, 2.9 Hz), 7.26–7.22 (3H, m), 7.10 (1H, dd, *J* = 2.9, 1.4 Hz), 7.03 (2H, t, *J* = 8.7 Hz), 6.27–6.21 (1H, m), 3.27 (1H, td, *J* = 9.6, 5.7 Hz), 2.89–2.74 (2H, m), 2.68–2.47 (2H, m),

2.46–2.35 (1H, m); ^{13}C NMR (101 MHz, $CDCl_3$) δ 203.9, 161.9 (d, J_{C-F} = 245.5 Hz), 142.4, 139.0 (d, J_{C-F} = 3.2 Hz), 131.9, 129.1 (d, J_{C-F} = 7.9 Hz), 125.9, 124.7, 120.7, 119.1, 115.9 (d, J_{C-F} = 21.4 Hz), 51.3, 40.3, 35.0, 25.3; ^{19}F NMR (376 MHz, $CDCl_3$) δ –115.73 (tt, J = 8.6, 5.3 Hz); HRMS (ASAP): Found MH^+ 287.0888, $C_{17}H_{16}OFS$ requires 287.0906.

Preparation of 19s

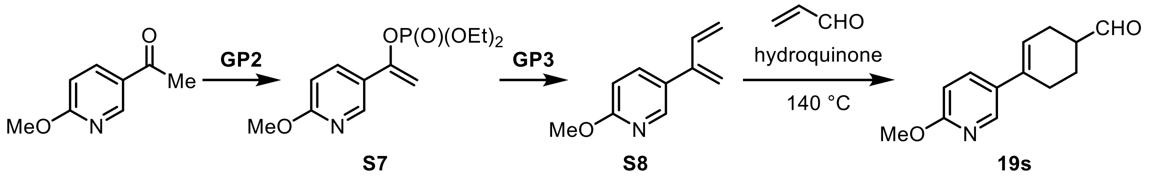

Diethyl (1-(6-methoxypyridin-3-yl)vinyl) phosphate (S7)

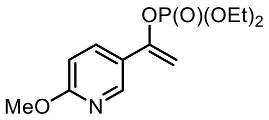

Following **GP2**, 1-(6-methoxypyridin-3-yl)ethan-1-one (3.02 g, 20.0 mmol, 1.0 equiv.), NaHMDS (2M, 13 mL, 26.0 mmol, 1.3 equiv.) and $(EtO)_2P(O)Cl$ (4.35 mL, 30.0 mmol, 3.0 equiv.) gave **S7** (4.60 g, 80%) as an oil. ^{1}H NMR (400 MHz, $CDCl_3$) δ 8.39 (1H, d, *J* = 2.5 Hz), 7.75 (1H, dd, *J* = 8.8, 2.6 Hz), 6.73 (1H, d, *J* = 8.7 Hz), 5.21–5.14 (2H, m), 4.28–4.14 (4H, m), 3.95 (3H, s), 1.35 (6H, t, *J* = 7.1 Hz); ^{31}P NMR ($CDCl_3$, 162 MHz) δ –6.22 – –6.47 (m). Data in accordance with literature.[8]

5-(Buta-1,3-dien-2-yl)-2-methoxypyridine (S8)

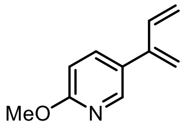

Following **GP3**, **S7** (4.60 g, 16.0 mmol), Ni(dppe)Cl_2 (232 mg, 2.5 mol%) and vinyl magnesium bromide in THF (1 M, 16.8 mL, 16.8 mmol, 1.05 equiv.) gave **S8** (1.97 g, 76%) as a solid. ^{1}H NMR (400 MHz, $CDCl_3$) δ 8.11 (1H, d, *J* = 2.4 Hz), 7.53 (1H, dd, *J* = 8.4, 2.4 Hz), 6.73 (1H, d, *J* = 8.5 Hz), 6.61 (1H, dd, *J* = 17.4, 10.7 Hz), 5.40–5.05 (4H, m), 3.95 (3H, s). Data in accordance with literature.[8]

4-(6-Methoxypyridin-3-yl)-3-cyclohexene-1-carbaldehyde (19s)

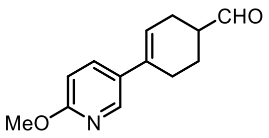

To a microwave vial equipped with a stir bar hydroquinone (2 mg, 0.22 mmol, 0.1 equiv.) and **S8** (0.32 g, 2.0 mmol, 1.0 equiv.) were added. The vial was capped, evacuated and refilled with N_2 thrice. Acrolein (0.3 mL, 8.0 mmol, 4.0 equiv.) was added and the reaction vessel was heated to 140 ºC for 5 h. The reaction was then allowed to cool to room temperature. The contents of the vial were dissolved in Et_2O, and the volatiles were removed

in vacuo to give a crude oil which was purified using column chromatography on silica gel eluting with EtOAc-pentane (2:8) to give **19s** (0.26 g, 61%) as a solid. R_f 0.34 [EtOAc–Pentane (2:8)]; ^{1}H NMR (400 MHz, $CDCl_3$) δ 9.73 (1H, s), 8.12 (1H, d, *J* = 2.5 Hz), 7.55 (1H, dd, *J* = 8.7, 2.6 Hz), 6.66 (1H, d, *J* = 8.7 Hz), 6.05–5.97 (1H, m), 3.90 (3H, s), 2.60–2.51 (1H, m), 2.50–2.35 (4H, m), 2.19–2.08 (1H, m), 1.87–1.73 (1H, m); ^{13}C NMR (101 MHz, $CDCl_3$) δ 204.1, 163.4, 143.2, 135.6, 133.8, 130.5, 121.7, 110.4, 53.5, 45.6, 25.9, 25.0, 22.5; HRMS (ESI): Found MH^+ 218.1174, $C_{13}H_{16}O_2N$ requires 218.1181.

Preparation of 24

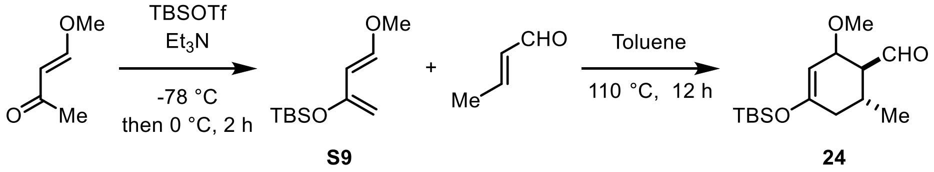

(*E*)-*tert*-Butyl((4-methoxybuta-1,3-dien-2-yl)oxy)dimethylsilane (S9)

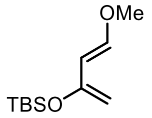

(*E*)-4-Methoxybut-3-en-2-one (1.6 mL, 15.8 mmol, 1.0 equiv.) was dissolved in Et_2O (32 mL, 0.5 M) and Et_3N (6 mL, 43.0 mmol, 2.7 equiv.) was added. The reaction was cooled to –78 ºC and then TBSOTf (3.2 mL, 17.7 mmol, 1.1 equiv.) was added dropwise. The reaction was warmed to 0 ºC and stirred for 2 h. The reaction was diluted with pentane (30 mL) and washed with ice-cold sat. $NaHCO_3$ solution (30 mL) and brine (30 mL). The layers were separated, and the organic layer was dried ($MgSO_4$), filtered through an alumina plug and evaporated to give **S9** (2.33 g, 69%) as an oil. ^{1}H NMR (400 MHz, $CDCl_3$) δ 6.88 (1H, d, *J* = 12.3 Hz), 5.35 (1H, d, *J* = 12.4 Hz), 4.07 (2H, d, *J* = 13.0 Hz), 3.58 (3H, s), 0.96 (9H, s), 0.19 (6H, s). Data in accordance with literature.[9]

4-((*tert*-Butyldimethylsilyl)oxy)-2-methoxy-*trans*-6-methyl-3-cyclohexene-1-carbaldehyde (24)

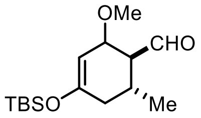

To a capped microwave vial equipped with a stir bar and purged with N_2, **S9** (0.57 g, 2.0 mmol, 1.0 equiv.), *trans*-crotonaldehyde (0.33 mL, 4.0 mmol, 2.0 equiv.) and toluene (2 mL, 1 M) were added. The reaction mixture was heated to 110 ºC for 12 h. The solution was then evaporated and purified via column chromatography on silica gel eluting with EtOAc-pentane (9:1) to give **24s** (0.34 g, 60%) as an oil. R_f 0.50 [EtOAc–pentane (9:1)]; ^{1}H NMR (400 MHz, $CDCl_3$) δ 9.75 (1H, d, *J* = 3.9 Hz), 5.00–4.97 (1H, m), 4.38–4.31 (1H, m), 3.28 (3H, s), 2.28 (1H, ddd, *J* = 10.6, 8.4, 3.8 Hz), 2.17–2.01 (2H, m), 1.94–1.81 (1H, m), 1.01 (3H, d, *J* = 6.3 Hz), 0.92 (9H, s), 0.16 (3H, s), 0.16 (3H, s); ^{13}C NMR (101 MHz, $CDCl_3$) δ 204.6, 152.8, 102.7, 75.9, 58.7, 55.3, 37.9, 28.7, 25.7, 19.4, 18.1, –4.2, –4.4; HRMS (ESI): Found MNa^+ 307.1687, $C_{15}H_{28}O_3SiNa$ requires 307.1705.

Preparation of 26s

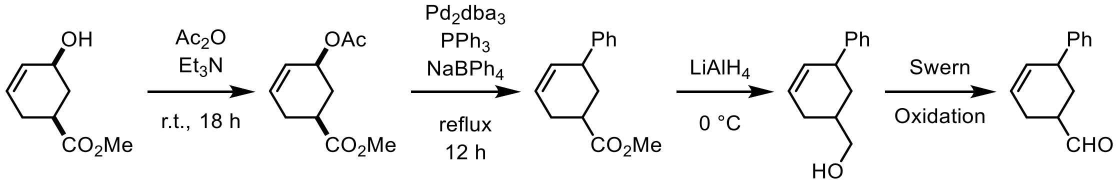

Methyl *cis*-5-acetoxy-3-cyclohexene-1-carboxylate (S11)

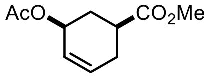

To a flame-dried flask purged with N_2, **S10** [prepared according to a literature procedure][10] (2.34 g, 15.0 mmol, 1.0 equiv.), CH_2Cl_2 (45 mL, 0.33M) and Et_3N (6.27 mL, 45.0 mmol, 3.0 equiv.) were added. The solution was cooled to 0 ºC and AcCl (2.24 mL, 31.5 mmol, 2.1 equiv.) was added dropwise. The solution was allowed to warm to room temperature and stirred for 18 h. The reaction was quenched with sat. NH_4Cl solution (30 mL) and layers were separated. The aqueous layer was extracted with CH_2Cl_2 (2 x 20 mL). The combined organic layer was dried ($MgSO_4$), evaporated and the crude was purified via column chromatography on silica gel eluting with EtOAc-pentane (1:9) to give **S11** as an oil. ^{1}H NMR (500 MHz, $CDCl_3$) δ 5.91–5.85 (1H, m), 5.66–5.61 (1H, m), 5.42–5.36 (1H, m), 3.70 (3H, s), 2.76–2.68 (1H, m), 2.41–2.33 (1H, m), 2.33–2.28 (2H, m), 2.05 (3H, s), 1.76 (1H, td, *J* = 12.3, 9.2 Hz). Data in accordance with literature.[10]

Methyl 3-phenyl-3-cyclohexene-1-carboxylate (S12)

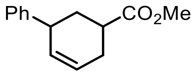

To a flame-dried RBF equipped with a reflux condenser and purged with N_2, $Pd_2(dba)_3$ (46 mg, 1 mol%), PPh_3 (52 mg, 4 mol%), $NaBPh_4$ (0.94 g, 2.75 mmol, 0.55 equiv.) and THF (5 mL, 1M) were added followed by **S11** (0.99g, 5.0 mmol, 1.0 equiv.). The reaction was heated under reflux for 12 h and then diluted with pentane (10 mL). The solution was filtered through celite, and the solvent was removed *in vacuo*. The resulting residue was purified via column chromatography on silica gel eluting with EtOAc-pentane (1:19) to give **S12** (432 mg, 40) as an oil. *anti*:*syn* = 3:1.

Data for *anti*-**S12** (major): ^{1}H NMR (400 MHz, $CDCl_3$) δ 7.35–7.27 (2H, m), 7.24–7.18 (3H, m), 6.01–5.93 (1H, m), 5.82–5.75 (1H, m), 3.65 (3H, s), 3.61–3.53 (1H, m), 2.67–2.55 (1H, m), 2.44–2.28 (2H, m), 2.16 (1H, ddd, *J* = 13.2, 10.6, 6.1 Hz), 2.01–1.94 (1H, m).

Data for *syn*-**S12** (minor): ^{1}H NMR (400 MHz, $CDCl_3$) δ 7.35–7.27 (2H, m), 7.24–7.18 (3H, m), 5.90–5.83 (1H, m), 5.75–5.70 (1H, m), 3.67 (3H, s), 3.53–3.43 (1H, m), 2.84–2.71 (1H, m), 2.44–2.28 (3H, m), 1.64 (1H, td, *J* = 12.9, 11.5 Hz).

Data in accordance with literature.[11],[12]

(1,2,3,4-Tetrahydro-[1,1'-biphenyl]-3-yl)methanol (S13)

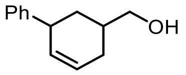

Following **GP4**, **S12** (377 mg, 2.0 mmol, 1.0 equiv.), $LiAlH_4$ (91 mg, 2.4 mmol, 1.2 equiv.) gave **S13** (347 mg, 92%) as an inseparable mixture of diasteromers, as an oil. dr = 3:1. R_f 0.16 [EtOAc–pentane (1:5)]; ^{1}H NMR (500 MHz, $CDCl_3$, diastereomers) δ 7.33–7.28 (2H, m), 7.25–7.18 (3H, m), 5.98–5.93 (0.7H, m), 5.90–5.84 (0.3H, m), 5.79–5.75 (0.7H, m), 5.75–5.71 (0.3H, m), 3.59–3.53 (0.7H, m), 3.52 (2H, d, *J* = 5.6 Hz), 3.49–3.43 (0.3H, m), 2.31–2.23 (0.7H, m), 2.23–2.16 (0.3H, m), 2.15–2.07 (0.3H, m), 2.06–1.96 (0.3H, m), 1.91–1.74 (3.3H, m), 1.37 (1H, br s), 1.29–1.18 (0.3H, m); ^{13}C NMR (126 MHz, $CDCl_3$, diastereomers) δ 146.6, 145.7, 131.1, 129.0, 128.6, 128.4, 128.4, 127.7, 127.5, 127.1, 126.3, 126.2, 68.2, 67.5, 43.1, 39.7, 37.4, 36.5, 33.9, 32.0, 28.4, 28.2; HRMS (ESI): Found 211.1097, $C_{13}H_{16}ONa$ requires 211.1099.

5-Phenyl-3-cyclohexene-1-carbaldehyde (26s)

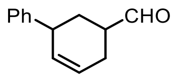

Following **GP5**, **S13** (347 mg, 1.84 mmol, 1.0 equiv.), DMSO (0.39 mL, 5.52 mmol, 3 equiv.), oxalyl chloride (0.24 mL, 2.76 mmol, 1.5 equiv.), Et_3N (1.56 mL, 11.0 mmol, 6.0 equiv.) gave **26s** (285 mg, 83%) as an inseparable mixture of diasteromers, as an oil. dr = 7:3. R_f 0.46 [EtOAc–pentane (1:8)]; ^{1}H NMR (400 MHz, $CDCl_3$, diastereomers) δ 9.90–9.53 (1H, m), 7.35–7.29 (2H, m), 7.26–7.18 (3H, m), 6.00–5.94 (0.7H, m), 5.93–5.87 (0.3H, m), 5.81–5.74 (1H, m), 3.60–3.47 (1H, m), 2.77–2.69 (0.3H, m), 2.62–2.53 (0.7H, m), 2.43–2.22 (2.3 H, m), 2.13 (0.7H, ddd, *J* = 13.2, 9.1, 6.0 Hz), 1.96 (0.7H, ddd, *J* = 13.2, 5.7, 3.5 Hz), 1.47 (0.3H, td, *J* = 12.8, 11.2 Hz); ^{13}C NMR (101 MHz, $CDCl_3$, diastereomers) δ 204.4, 203.6, 145.4, 144.8, 131.1, 129.6, 128.8, 128.6, 128.2, 127.5, 126.7, 126.6, 126.5, 125.9, 47.4, 43.1, 42.5, 39.3, 32.7, 31.2, 24.5, 24.3; HRMS (ESI): Found 209.0942, $C_{13}H_{14}ONa$ requires 209.0942.

Preparation of 27s

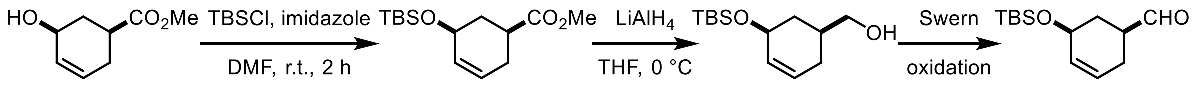

***cis*-Methyl 5-((*tert*-butyldimethylsilyl)oxy)-3-cyclohexene-1-carboxylate (S14)**

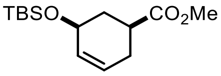

TBSCl (2.26 g, 15.0 mmol, 1.5 equiv.) was added in three portions to a stirred solution of **S10** (1.56 g, 10.0 mmol, 1.0 equiv.), imidazole (2.04 g, 30.0 mmol, 3.0 equiv.) in dry DMF (25 mL, 0.4 M) at 0 °C, and the reaction mixture was stirred at room temperature for 2 hours. The reaction was diluted with saturated aqueous NH_4Cl (20 mL), extracted with Et_2O (3 x 100 mL). The combined organic layers were washed with H_2O (20 mL) and brine (20 mL), dried (Na_2SO_4), filtered and evaporated. The crude was purified by column chromatography on silica gel eluting with petrol–EtOAc (20:1) to give **S14** (2.16 g, 80%) as an oil. ^{1}H NMR (400 MHz, $CDCl_3$) δ 5.73–5.64 (1H, m), 5.64–5.55 (1H, m), 4.39–4.27 (1H, m), 3.68 (3H, s), 2.63 (1H, dtd, *J* = 12.9, 8.0, 2.7 Hz), 2.28–2.17 (3H, m), 1.64 (1H, td, *J* = 12.7, 9.6 Hz), 0.89 (9H, s), 0.07 (3H, s), 0.06 (3H, s); ^{13}C NMR (101 MHz, $CDCl_3$) δ 175.3, 132.1, 126.4, 67.9, 51.9, 38.7, 35.0, 27.5, 26.0, 18.3, –4.4, –4.6. Data in accordance with the literature.[13]

***cis*-5-((*tert*-Butyldimethylsilyl)oxy)cyclohex-3-en-1-yl)methanol (S15)**

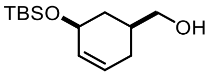

Following **GP4**, **S14** (1.35 g, 5.0 mmol, 1.0 equiv.) and $LiAlH_4$ (323 mg, 6.0 mmol, 1.2 equiv.) gave **S15** (1.03 g, 85%) as an oil. ^{1}H NMR (400 MHz, $CDCl_3$) δ 5.78–5.68 (1H, m), 5.67–5.58 (1H, m), 4.36–4.26 (1H, m), 3.63–3.50 (2H, m), 2.15–2.05 (1H, m), 2.02–1.95 (1H, m), 1.95–1.87 (2H, m), 1.86–1.72 (1H, m), 1.43 (1H, ddd, *J* = 12.4, 10.0, 7.7 Hz), 0.90 (9H, s), 0.09 (6H, s); ^{13}C NMR (101 MHz, $CDCl_3$) δ 131.0, 128.4, 67.3, 67.0, 35.3, 35.2, 28.1, 25.8, 18.1, –3.5. Data in accordance with the literature.[14]

***cis*-Methyl 5-((*tert*-butyldimethylsilyl)oxy)-3-cyclohexene-1-carboxylate (27s)**

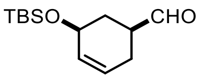

Following **GP5**, **S15** (800 mg, 3.3 mmol, 1.0 equiv.) gave **27s** (638 mg, 80%) as an oil. ^{1}H NMR (400 MHz, $CDCl_3$) δ 9.68 (1H, d, *J* = 1.2 Hz), 5.77–5.68 (1H, m), 5.68–5.58 (1H, m), 4.34–4.25 (1H, m), 2.60–2.48 (1H, m), 2.38–2.26 (1H, m), 2.22–2.07 (2H, m), 1.72 (1H, ddd,

J = 12.9, 10.0, 7.3 Hz), 0.87 (9H, s), 0.06 (3H, s), 0.05 (3H, s); ^{13}C NMR (101 MHz, $CDCl_3$) δ 202.8, 131.5, 126.5, 66.2, 45.1, 32.3, 25.9, 24.0, 18.2, –4.5, –4.6. Data in accordance with the literature.[13]

Preparation of 28s

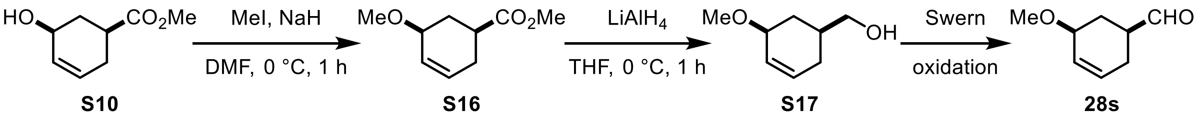

***cis*-Methyl 5-methoxy-3-cyclohexene-1-carboxylate (S16)**

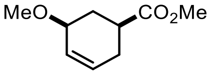

MeI (3.1 mL, 50.0 mmol, 10.0 equiv.) and NaH (60% in mineral oil, 400 mg, 10.0 mmol, 2.0 equiv.) were added to a solution of the **S10** (781 mg, 5.0 mmol, 1.0 equiv.) in dry DMF (10 mL, 0.5 M) at 0 °C and the reaction mixture was stirred at the room temperature for 1 h. The reaction was quenched with saturated aqueous NH_4Cl (5 mL). The aqueous layer was extracted with EtOAc (3 × 10 mL). The combined organic layers were washed with brine (10 mL), dried (Na_2SO_4), filtered and evaporated. The crude was purified by column chromatography on silica gel eluting with petrol–EtOAc (4:1) to give **S16** (700 mg, 82%) as an oil. ^{1}H NMR (400 MHz, $CDCl_3$) δ 5.81–5.74 (1H, m), 5.74–5.67 (1H, m), 3.96–3.85 (1H, m), 3.67 (3H, s), 3.35 (3H, s), 2.67–2.55 (1H, m), 2.39–2.30 (1H, m), 2.29–2.20 (2H, m), 1.60 (1H, dt, *J* = 12.3, 9.5 Hz); ^{13}C NMR (101 MHz, $CDCl_3$) δ 175.2, 128.5, 127.8, 75.4, 55.8, 51.9, 38.2, 30.7, 27.7. Data in accordance with the literature.[15]

***cis*-(5-Methoxycyclohex-3-en-1-yl)methanol (S17)**

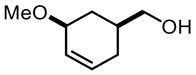

Following **GP4**, **S16** (681 mg, 4.0 mmol, 1.0 equiv.) and $LiAlH_4$ (259 mg, 4.8 mmol, 1.2 equiv.) gave **S17** (555 mg, 98%) as an oil. ^{1}H NMR (400 MHz, $CDCl_3$) δ 5.84–5.77 (1H, m), 5.77–5.71 (1H, m), 3.93–3.83 (1H, m), 3.56 (1H, dd, *J* = 9.6, 4.9 Hz), 3.52 (1H, dd, *J* = 9.6, 5.4 Hz), 3.37 (3H, s), 2.18–2.02 (3H, m), 1.94–1.83 (1H, m), 1.83–1.73 (1H, m), 1.29 (1H, ddd, *J* = 12.4, 11.0, 8.7 Hz); ^{13}C NMR (101 MHz, $CDCl_3$) δ 129.0, 128.2, 75.5, 67.3, 55.8, 35.0, 31.2, 28.3. Data in accordance with the literature.[14]

***cis*-5-Methoxy-3-cyclohexene-1-carbaldehyde (28s)**

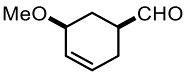

Following **GP5**, **S17** (540 mg, 3.8 mmol, 1.0 equiv.) gave **28s** (530 mg, 98%) as an oil. R_f 0.37 [petrol–EtOAc (2:1)]; ^{1}H NMR (400 MHz, $CDCl_3$) δ 9.66 (1H, s), 5.87–5.80 (1H, m), 5.80–5.74 (1H, m), 3.92–3.83 (1H, m), 3.34 (3H, s), 2.60–2.48 (1H, m), 2.38–2.24 (2H, m),

2.23–2.14 (1H, m), 1.70 (1H, ddd, J = 13.0, 10.2, 7.6 Hz); ^{13}C NMR (101 MHz, $CDCl_3$) δ 202.9, 128.4, 128.0, 74.1, 55.9, 44.9, 28.1, 24.4; HRMS (ESI): Found MNa^+ 163.0724, $C_8H_{12}O_2Na$ requires 163.0730.

Preparation of 29s

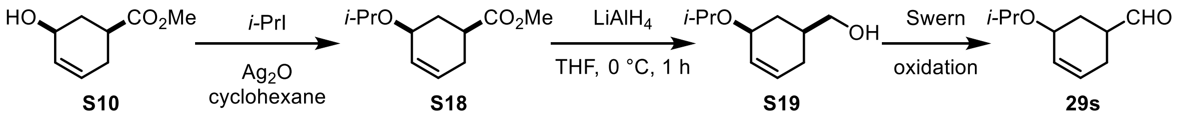

cis-Methyl 5-(*iso*-Propoxy)-3-cyclohexene-1-carboxylate (S18)

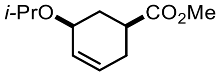

S10 (781 mg, 5.0 mmol, 1.0 equiv.) was dissolved in dry cyclohexane (10 mL, 0.2 M) and treated with Ag_2O (1.24 g, 10.0 mmol, 2.0 equiv.) and *i*-Pr–I (1.5 mL, 15.0 mmol, 3.0 equiv.). The reaction was stirred for 48 h, diluted with CH_2Cl_2 (20 mL), filtered on celite and evaporated. The crude was purified by column chromatography on silica gel eluting with petrol–EtOAc (20:1) to give **S18** (780 mg, 78%) as an oil. ^1H NMR (400 MHz, $CDCl_3$) δ 5.79–5.70 (1H, m), 5.70–5.62 (1H, m), 4.11–4.01 (1H, m), 3.74 (1H, hept, *J* = 6.1 Hz), 3.68 (3H, s), 2.69–2.56 (1H, m), 2.37–2.28 (1H, m), 2.27–2.18 (2H, m), 1.60 (1H, td, *J* = 12.5, 9.6 Hz), 1.15 (3H, d, *J* = 6.1 Hz), 1.14 (3H, d, *J* = 6.1 Hz); ^{13}C NMR (101 MHz, $CDCl_3$) δ 175.3, 130.0, 127.3, 71.6, 69.4, 51.9, 38.6, 32.3, 27.7, 23.2, 22.7. Data in accordance with the literature.[14]

cis-(5-*iso*-Propoxycyclohex-3-en-1-yl)methanol (S19)

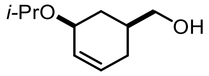

Following **GP4**, **S18** (594 mg, 3.0 mmol, 1.0 equiv.) and $LiAlH_4$ (194 mg, 3.6 mmol, 1.2 equiv.) gave **S19** (468 mg, 92%) as an oil. ^1H NMR (500 MHz, $CDCl_3$) δ 5.78–5.71 (1H, m), 5.69–5.63 (1H, m), 4.03–3.96 (1H, m), 3.74 (1H, hept, *J* = 6.1 Hz), 3.55–3.44 (2H, m), 2.92 (1H, s), 2.12–2.00 (2H, m), 1.93–1.80 (1H, m), 1.80–1.69 (1H, m), 1.35–1.25 (1H, m), 1.14 (3H, d, *J* = 6.1 Hz), 1.13 (3H, d, *J* = 6.1 Hz); ^{13}C NMR (126 MHz, $CDCl_3$) δ 129.2, 128.5, 71.1, 69.2, 67.0, 34.9, 32.5, 28.2, 22.9, 22.7. Data in accordance with the literature.[14]

5-(*iso*-Propoxy)-3-cyclohexene-1-carbaldehyde (29s)

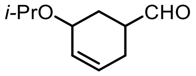

Following **GP5**, **S19** (460 mg, 2.7 mmol, 1.0 equiv.) gave **29s** (415 mg, 91%) as an oil. R_f 0.28 [petrol–EtOAc (8:1)]; d.r. = 10:1; data for the major isomer: ^1H NMR (400 MHz, $CDCl_3$) δ 9.66 (1H, s), 5.83–5.74 (1H, m), 5.74–5.66 (1H, m), 4.07–3.97 (1H, m), 3.72 (1H, hept, *J* = 6.1 Hz), 2.59–2.47 (1H, m), 2.37–2.26 (1H, m), 2.26–2.11 (2H, m), 1.69 (1H, ddd, *J*

= 12.8, 10.1, 7.5 Hz), 1.13 (3H, d, *J* = 6.1 Hz), 1.12 (3H, d, *J* = 6.1 Hz); ^{13}C NMR (101 MHz, $CDCl_3$) δ 203.0, 129.5, 127.5, 69.8, 69.2, 45.0, 29.5, 24.2, 22.9, 22.6; HRMS (ESI): Found MNa^+ 191.1048, $C_{10}H_{16}O_2Na$ requires 191.1043.

Preparation of 30s

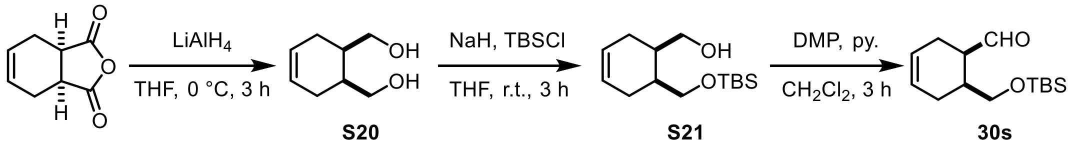

***cis*-4-Cyclohexene-l,2-dimethanol (S20)**

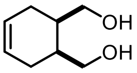

Following **GP4**, *cis*-4-Cyclohexene-1,2-dicarboxylic anhydride (7.61 g, 50.0 mmol, 1.0 equiv.) and $LiAlH_4$ (6.73 g, 125.0 mmol, 2.5 equiv.) gave **S20** as an oil (7.04 g, 99%). ^{1}H NMR (400 MHz, $CDCl_3$) δ 5.77–5.47 (2H, m), 3.73 (2H, dd, *J* = 11.0, 6.6 Hz), 3.60 (2H, dd, *J* = 11.0, 3.5 Hz), 2.81 (2H, br s), 2.25–1.94 (6H, m). Data in accordance with the literature.[16]

***cis*-6-(((*tert*-Butyldimethylsilyl)oxy)methyl)-3-cyclohexene-1-methanol (S21)**

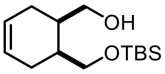

A solution of the crude **S20** (7.11 g, 50.0 mmol, 1.0 equiv.) in dry THF (20 mL) was added slowly to a suspension of NaH (2.00 g, 50.0 mmol, 1.0 equiv., 60% in mineral oil) in dry THF (180 mL) at 0 °C. The mixture was stirred at room temperature for 1.5 h. TBSCl (7.54 g, 50.0 mmol, 1.0 equiv.) was added and the mixture was stirred at room temperature for 3 h. The reaction was quenched with saturated aqueous NH_4Cl solution (20 mL) and extracted with EtOAc (3 × 50 mL). The combined organic layers were washed with brine (20 mL), dried (Na_2SO_4), filtered and evaporated. The crude was purified by column chromatography on silica gel eluting with petrol–EtOAc (8:1) to give **S21** (1.21 g, 94%) as an oil. ^{1}H NMR (400 MHz, $CDCl_3$) δ 5.68–5.47 (2H, m), 3.71 (1H, dd, *J* = 10.3, 6.9 Hz), 3.66–3.57 (1H, m), 3.54 (1H, dd, *J* = 10.3, 3.6 Hz), 3.51–3.45 (2H, m), 2.21–1.85 (6H, m), 0.88 (9H, s), 0.05 (6H, s); ^{13}C NMR (101 MHz, $CDCl_3$) δ 125.8, 125.4, 64.9, 64.2, 38.3, 37.5, 27.3, 26.8, 25.9, 18.3, –5.4 (2 x C). Data in accordance with the literature.[17]

***cis*-6-(((*tert*-Butyldimethylsilyl)oxy)methyl)-3-cyclohexene-1-carbaldehyde (30s)**

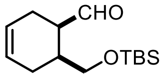

Following **GP6**, **S21** (513 mg, 2.0 mmol, 1.0 equiv.), pyridine (1.6 mL, 20.0 mmol, 10.0 equiv.) and DMP (2.54 g, 6.0 mmol, 3.0 equiv.) gave **30s** (420 mg, 83%) as an oil. R_f 0.32

[petrol–EtOAc (20:1)]; ^{1}H NMR (400 MHz, $CDCl_3$) δ 9.71 (1H, s), 5.70–5.63 (1H, m), 5.63–5.56 (1H, m), 3.59 (1H, dd, *J* = 10.0, 8.8 Hz), 3.52 (1H, dd, *J* = 10.0, 5.7 Hz), 2.57–2.46 (2H, m), 2.31–2.17 (3H, m), 1.99–1.87 (1H, m), 0.84 (9H, s), –0.01 (6H, d, *J* = 4.0 Hz); ^{13}C NMR (101 MHz, $CDCl_3$) δ 203.8, 125.7, 125.0, 63.5, 47.6, 36.5, 27.0, 25.9, 22.6, 18.3, –5.5; HRMS (ASAP): Found MH^+ 255.1766, $C_{14}H_{27}O_2Si$ requires 255.1775.

Preparation of 31s

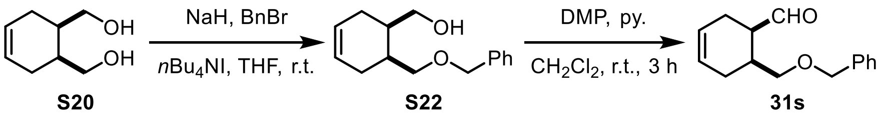

***cis*-6-((Benzyloxy)methyl)cyclohex-3-en-1-yl)methanol (S22)**

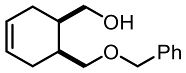

NaH (60% in mineral oil, 400 mg, 10.0 mmol, 2.0 equiv.) was added to a solution of **S20** (711 g, 5.0 mmol, 1.0 equiv.) in dry THF (25 mL, 0.2 M) at 0 °C. The reaction was stirred at room temperature for 15 min, then Bn–Br (0.62 mL, 5.25 mmol, 1.05 equiv.) and Bu_4NI (37 mg, 1.0 mmol, 0.2 equiv.) were added at 0 °C. The reaction mixture was stirred at room temperature overnight, then quenched with saturated aqueous NH_4Cl (10 mL) at 0 °C. H_2O (20 mL) was added and the layers were separated. The aqueous layer was extracted with EtOAc (3 x 15 mL). The combined organic layers were washed with brine (15 mL), dried (Na_2SO_4), filtered and evaporated. The crude was purified by column chromatography on silica gel eluting with petrol–EtOAc (4:1) to give **S22** (700 g, 60%) as an oil. ^{1}H NMR (500 MHz, $CDCl_3$) δ 7.39–7.26 (5H, m), 5.66–5.56 (2H, m), 4.52 (2H, s), 3.63 (1H, dd, *J* = 11.4, 6.7 Hz), 3.59 (1H, dd, *J* = 9.5, 7.7 Hz), 3.54 (1H, dd, *J* = 11.4, 4.8 Hz), 3.41 (1H, dd, *J* = 9.5, 4.9 Hz), 3.06 (1H, s), 2.29–2.22 (1H, m), 2.22–2.14 (1H, m), 2.14–2.04 (2H, m), 2.03–1.92 (2H, m); ^{13}C NMR (126 MHz, $CDCl_3$) δ 137.9, 128.5, 127.8 (2 x C), 125.8, 125.5, 73.4, 71.8, 64.3, 38.0, 34.9, 27.9, 26.3; HRMS (ASAP): Found MH^+ 233.1534, $C_{15}H_{21}O_2$ requires 233.1536.

***cis*-6-((Benzyloxy)methyl)-3-cyclohexene-1-carbaldehyde (31s)**

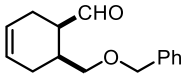

Following **GP6**, **S22** (558 mg, 2.4 mmol, 1.0 equiv.), pyridine (1.9 mL, 24.0 mmol, 10.0 equiv.) and DMP (3.05 g, 7.2 mmol, 3.0 equiv.) gave **32s** (450 mg, 81%) as an oil. R_f 0.53 [petrol–EtOAc (4:1)]; ^{1}H NMR (500 MHz, $CDCl_3$) δ 9.68 (1H, s), 7.29–7.24 (2H, m), 7.24–7.18 (3H, m), 5.66–5.60 (1H, m), 5.59–5.55 (1H, m), 4.39 (2H, s), 3.43 (1H, t, *J* = 9.0 Hz), 3.37 (1H, dd, *J* = 9.3, 5.8 Hz), 2.61–2.52 (2H, m), 2.26–2.18 (3H, m), 1.97–1.90 (1H, m); ^{13}C NMR (126 MHz, $CDCl_3$) δ 203.9, 138.1, 128.4, 127.7, 127.6, 125.7, 124.9, 73.3, 70.6, 47.5, 34.2, 27.2, 22.7; HRMS (ESI): Found MNa^+ 253.1198, $C_{15}H_{18}O_2Na$ requires 253.1199.

Preparation of 32s

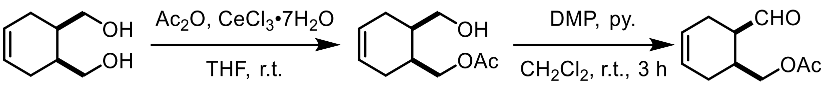

S20 **S23** **32s**

***cis*-6-(Hydroxymethyl)-3-cyclohexene-1-methyl acetate (S23)**

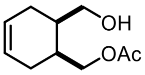

Ac_2O (4.7 mL, 50.0 mmol, 10.0 equiv.) was added to a solution of **S20** (711 mg, 5.0 mmol, 1.0 equiv.) and $CeCl_3 \cdot H_2O$ (186 mg, 0.5 mmol, 0.1 equiv.) in THF (20 mL, 0.25 M) at 0 °C. The mixture was stirred at room temperature overnight and then diluted with Et_2O (20 mL). The layers were separated and the organic layer was washed with saturated aqueous $NaHCO_3$ (2 x 20 mL) and brine (15 mL), dried (Na_2SO_4), filtered and evaporated. The crude was purified by column chromatography on silica gel eluting with petrol–EtOAc (2:1) to give **S23** (300 mg, 32%) as an oil. ^{1}H NMR (500 MHz, $CDCl_3$) δ 5.70–5.59 (2H, m), 4.20 (1H, dd, *J* = 11.0, 6.0 Hz), 3.96 (1H, dd, *J* = 11.0, 7.9 Hz), 3.68 (1H, dd, *J* = 10.9, 7.1 Hz), 3.60 (1H, dd, *J* = 10.9, 6.9 Hz), 2.29–2.21 (1H, m), 2.19–2.07 (3H, m), 2.06 (3H, s), 2.01–1.94 (1H, m), 1.92–1.83 (2H, m); ^{13}C NMR (126 MHz, $CDCl_3$) δ 171.6, 125.8, 125.3, 65.1, 63.9, 37.4, 33.3, 27.2, 26.1, 21.2. Data in accordance with the literature.[18]

***cis*-(6-Formylcyclohex-3-en-1-yl)methyl acetate (32s)**

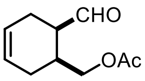

Following **GP6**, **S23** (295 mg, 1.6 mmol, 1.0 equiv.), pyridine (1.3 mL, 16.0 mmol, 10.0 equiv.) and DMP (2.04 g, 4.8 mmol, 3.0 equiv.) gave **32s** (170 mg, 58%) as an oil. ^{1}H NMR (500 MHz, $CDCl_3$) δ 9.72 (1H, s), 5.74–5.68 (1H, m), 5.68–5.63 (1H, m), 4.13–4.05 (2H, m), 2.67–2.57 (2H, m), 2.33–2.26 (3H, m), 2.06–2.01 (1H, m), 2.00 (3H, s); ^{13}C NMR (126 MHz, $CDCl_3$) δ 203.5, 170.9, 125.6, 124.9, 64.6, 47.4, 32.9, 27.0, 22.9, 20.9. Data in accordance with the literature.[18]

Preparation of 33s

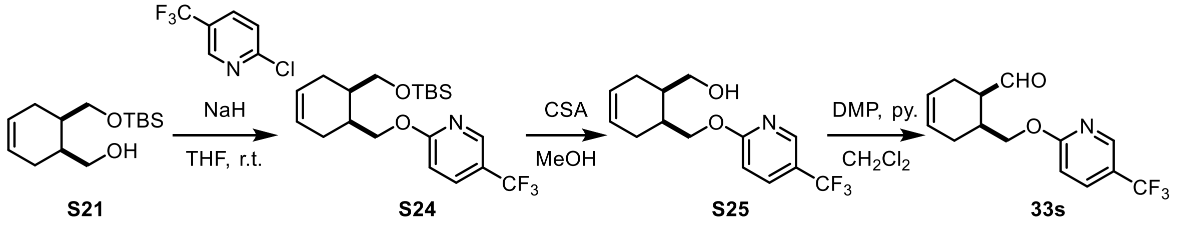

***cis*-2-((6-(((*tert*-Butyldimethylsilyl)oxy)methyl)cyclohex-3-en-1-yl)methoxy)-5-(trifluoromethyl)pyridine (S24)**

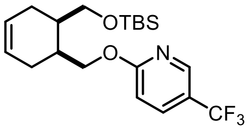

NaH (60% in mineral oil, 144 mg, 3.6 mmol, 1.2 equiv.) was added to a solution of **S21** (769 mg, 3.0 mmol, 1.0 equiv.) in dry THF (12 mL, 0.25M) at 0 °C and the reaction was stirred at room temperature for 30 min. Then 2-chloro-5-(trifluoromethyl)pyridine (653 mg, 3.6 mmol, 1.2 equiv.) was added and the mixture was stirred at room temperature overnight. The reaction was diluted with H_2O (5 mL) and EtOAc (15 mL) and the layers were separated. The aqueous layer was extracted with EtOAc (2 × 15 mL) and the combined organic layers were washed with brine (15 mL), dried (Na_2SO_4), filtered and evaporated. The crude was purified by column chromatography on silica gel eluting with petrol–EtOAc (30:1) to give **S24** (1.12 g, 93%) as an oil. ^{1}H NMR (400 MHz, $CDCl_3$) δ 8.41 (1H, d, *J* = 2.6 Hz), 7.74 (1H, dd, *J* = 8.7, 2.6 Hz), 6.79 (1H, d, *J* = 8.7 Hz), 5.72–5.58 (2H, m), 4.42 (1H, dd, *J* = 10.5, 5.9 Hz), 4.28 (1H, dd, *J* = 10.5, 8.5 Hz), 3.71 (1H, dd, *J* = 10.0, 6.4 Hz), 3.58 (1H, dd, *J* = 10.0, 7.0 Hz), 2.47–2.36 (1H, m), 2.27–2.02 (4H, m), 2.02–1.91 (1H, m), 0.88 (9H, s), 0.03 (6H, s); ^{13}C NMR (101 MHz, $CDCl_3$) δ 166.3, 145.1 (q, $J_{C\text{-}F}$ = 4.5 Hz), 135.6 (q, $J_{C\text{-}F}$ = 3.1 Hz), 126.1, 125.3, 124.2 (q, $J_{C\text{-}F}$ = 271.0 Hz), 119.8 (q, $J_{C\text{-}F}$ = 33.0 Hz), 111.4, 67.7, 64.0, 37.5, 34.1, 27.1, 26.6, 26.1, 18.4, –5.3; ^{19}F NMR (376 MHz, $CDCl_3$) δ –61.5; HRMS (ASAP): Found MH^+ 402.2068, $C_{20}H_{31}O_2NF_3Si$ requires 402.2071.

***cis*-6-(((5-(Trifluoromethyl)pyridin-2-yl)oxy)methyl)-3-cyclohexene-1-methanol (S25)**

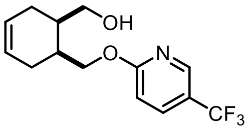

Following **GP8**, **S24** (1.00 g, 2.5 mmol, 1.0 equiv.) and CSA (58 mg, 0.25 mmol, 0.1 equiv.) gave **S25** (718 mg, quant.) as an oil. R_f 0.24 [petrol–EtOAc (4:1)]; ^{1}H NMR (400 MHz,

$CDCl_3$) δ 8.39 (1H, d, *J* = 2.6 Hz), 7.75 (1H, dd, *J* = 8.7, 2.6 Hz), 6.80 (1H, d, *J* = 8.7 Hz), 5.72–5.59 (2H, m), 4.53 (1H, dd, *J* = 10.8, 5.4 Hz), 4.18 (1H, dd, *J* = 10.8, 8.1 Hz), 3.73 (1H, ddd, *J* = 11.0, 7.6, 4.6 Hz), 3.65 (1H, dt, *J* = 11.0, 6.8 Hz), 2.86–2.74 (1H, m), 2.50–2.38 (1H, m), 2.27–2.02 (4H, m), 1.93–1.81 (1H, m); ^{13}C NMR (101 MHz, $CDCl_3$) δ 165.9, 144.9 (q, J_{C-F} = 4.5 Hz), 136.0 (q, J_{C-F} = 3.1 Hz), 125.8, 125.3, 124.0 (q, J_{C-F} = 271.0 Hz), 120.1 (q, J_{C-F} = 33.2 Hz), 111.7, 66.9, 64.1, 37.3, 32.9, 27.4, 25.9; ^{19}F NMR (376 MHz, $CDCl_3$) δ –61.6; HRMS (ASAP): Found MH^+ 288.1200, $C_{14}H_{17}O_2NF_3$ requires 288.1206.

***cis*-6-(((5-(Trifluoromethyl)pyridin-2-yl)oxy)methyl)-3-cyclohexene-1-carbaldehydel (33s)**

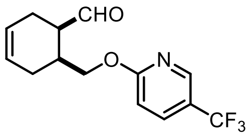

Following **GP6**, **S25** (632 mg, 2.2 mmol, 1.0 equiv.), pyridine (1.8 mL, 22.0 mmol, 10.0 equiv.) and DMP (2.80 g, 6.6 mmol, 3.0 equiv.) gave **33s** (527 mg, 84%) as an oil. R_f 0.42 [petrol–EtOAc (4:1)]; ^1H NMR (400 MHz, $CDCl_3$) δ 9.78 (1H, s), 8.38 (1H, d, *J* = 2.6 Hz), 7.73 (1H, dd, *J* = 8.7, 2.6 Hz), 6.74 (1H, d, *J* = 8.7 Hz), 5.76–5.63 (2H, m), 4.40 (1H, dd, *J* = 10.8, 8.6 Hz), 4.35 (1H, dd, *J* = 10.8, 6.4 Hz), 2.86–2.74 (1H, m), 2.70 (1H, td, *J* = 6.8, 3.0 Hz), 2.42–2.26 (3H, m), 2.18–2.07 (1H, m); ^{13}C NMR (101 MHz, $CDCl_3$) δ 203.6, 165.5, 145.0 (q, J_{C-F} = 4.5 Hz), 135.8 (q, J_{C-F} = 3.2 Hz), 125.7, 125.0, 124.1 (q, J_{C-F} = 271.2 Hz), 120.3 (q, J_{C-F} = 33.1 Hz), 111.3, 66.7, 47.5, 33.17, 27.0, 22.8; ^{19}F NMR (376 MHz, $CDCl_3$) δ –61.6; HRMS (ASAP): Found MH^+ 286.1037, $C_{14}H_{15}O_2NF_3$ requires 286.1049.

Preparation of 34s

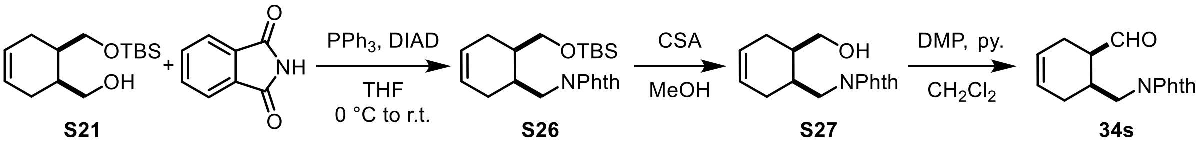

***cis*-2-((6-(((*tert*-Butyldimethylsilyl)oxy)methyl)cyclohex-3-en-1-yl)methyl)isoindoline-1,3-dione (S26)**

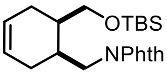

Following **GP7**, **S21** (1.03 g, 4.0 mmol, 1.0 equiv.), DIAD (0.95 mL, 4.8 mmol, 1.2 equiv.), PPh_3 (1.26 g, 4.8 mmol, 1.2 equiv.) and phthalimide (706 mg, 4.8 mmol, 1.2 equiv.) gave **S26** (1.50 g, 97%) as an oil. R_f 0.37 [petrol–EtOAc (8:1)]; ^{1}H NMR (400 MHz, $CDCl_3$) δ 7.82 (2H, dd, J = 5.5, 3.0 Hz), 7.69 (2H, dd, J = 5.5, 3.0 Hz), 5.68–5.61 (1H, m), 5.61–5.54 (1H, m), 3.78 (1H, dd, J = 13.6, 9.6 Hz), 3.75 (1H, dd, J = 10.0, 5.4 Hz), 3.67 (1H, dd, J = 13.6, 5.3 Hz), 3.57 (1H, dd, J = 10.0, 7.0 Hz), 2.42–2.30 (1H, m), 2.18–1.98 (4H, m), 1.94–1.82 (1H, m), 0.87 (9H, s), 0.05 (3H, s), 0.04 (3H, s); ^{13}C NMR (101 MHz, $CDCl_3$) δ 168.7, 133.9, 132.2, 125.9, 125.2, 123.2, 63.4, 39.9, 37.8, 34.4, 27.6, 26.7, 26.1, 18.4, –5.3 (2 x C); HRMS (ASAP): Found MH^+ 386.2149, $C_{22}H_{32}O_3NSi$ requires 386.2146.

***cis*-2-((6-(Hydroxymethyl)cyclohex-3-en-1-yl)methyl)isoindoline-1,3-dione (S27)**

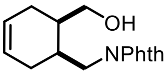

Following **GP8**, **S26** (1.45 g, 3.8 mmol, 1.0 equiv.) and CSA (88 mg, 0.38 mmol, 0.1 equiv.) gave **S27** (1.03 g, quant.) as a solid. R_f 0.26 [petrol–EtOAc (2:1)]; ^{1}H NMR (500 MHz, $CDCl_3$) δ 7.81–7.74 (2H, m), 7.70–7.63 (2H, m), 5.66–5.55 (2H, m), 3.81 (1H, dt, J = 11.6, 6.1 Hz), 3.72–3.62 (2H, m), 3.59 (1H, dt, J = 11.9, 6.8 Hz), 3.09–2.74 (1H, m), 2.31–2.21 (1H, m), 2.09–1.95 (3H, m), 1.95–1.85 (2H, m); ^{13}C NMR (126 MHz, $CDCl_3$) δ 168.9, 134.0, 132.0, 125.7, 125.2, 123.3, 63.0, 38.7, 37.2, 33.9, 27.5, 26.3; HRMS (ASAP): Found MH^+ 272.1278, $C_{16}H_{18}O_3N$ requires 272.1281.

***cis*-6-((1,3-Dioxoisoindolin-2-yl)methyl)-3-cyclohexene-1-carbaldehyde (34s)**

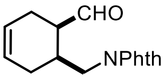

Following **GP6**, **S27** (814 mg, 3.0 mmol, 1.0 equiv.), pyridine (2.4 mL, 30.0 mmol, 10.0 equiv.) and DMP (3.82 g, 9.0 mmol, 3.0 equiv.) gave **34s** (650 mg, 80%) as a solid. R_f 0.29

[petrol–EtOAc (4:1)]; ^{1}H NMR (400 MHz, $CDCl_3$) δ 9.75 (1H, s), 7.86–7.77 (2H, m), 7.74–7.65 (2H, m), 5.76–5.69 (1H, m), 5.69–5.60 (1H, m), 3.78–3.76 (1H, m), 3.76–3.74 (1H, m), 2.70–2.58 (2H, m), 2.50–2.38 (1H, m), 2.29–2.16 (2H, m), 2.15–2.04 (1H, m); ^{13}C NMR (101 MHz, $CDCl_3$) δ 203.5, 168.5, 134.1, 132.0, 125.8, 124.9, 123.4, 47.4, 39.5, 33.2, 27.8, 23.6; HRMS (ASAP): Found MH^+ 270.1120, $C_{16}H_{16}O_3N$ requires 270.1125.

Preparation of 35s

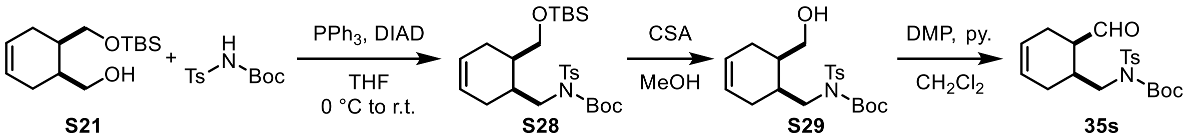

***cis*-*tert*-Butyl ((6-(((*tert*-butyldimethylsilyl)oxy)methyl)cyclohex-3-en-1-yl)methyl)(tosyl)carbamate (S28)**

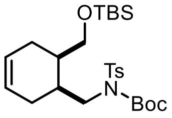

Following **GP7**, **S21** (1.28 g, 5.0 mmol, 1.0 equiv.), DIAD (1.2 mL, 6.0 mmol, 1.2 equiv.), PPh_3 (1.57 g, 6.0 mmol, 1.2 equiv.) and *tert*-butyl tosylcarbamate (1.63 g, 6.0 mmol, 1.2 equiv.) gave **S28** (2.19 g, 86%) as an oil. R_f 0.45 [petrol–EtOAc (8:1)]; ^{1}H NMR (500 MHz, $CDCl_3$) δ 7.75 (2H, d, *J* = 8.2 Hz), 7.28 (2H, d, *J* = 8.2 Hz), 5.69–5.61 (2H, m), 4.01 (1H, dd, *J* = 14.2, 10.0 Hz), 3.83 (1H, dd, *J* = 14.2, 4.3 Hz), 3.75 (1H, dd, *J* = 10.0, 5.6 Hz), 3.55 (1H, dd, *J* = 10.0, 7.6 Hz), 2.43 (3H, s), 2.38–2.29 (1H, m), 2.21–2.12 (2H, m), 2.10–1.92 (3H, m), 1.31 (9H, s), 0.90 (9H, s), 0.05 (6H, s); ^{13}C NMR (126 MHz, $CDCl_3$) δ 151.3, 144.1, 137.7, 129.3, 127.9, 125.8, 125.6, 84.1, 63.3, 49.4, 38.0, 36.3, 28.0, 27.2, 26.9, 26.1, 21.7, 18.4, –5.2, –5.3; HRMS (ESI): Found MNa^+ 532.2523, $C_{26}H_{43}O_5NSSiNa$ requires 532.2523.

***cis*-*tert*-Butyl ((6-(hydroxymethyl)cyclohex-3-en-1-yl)methyl)(tosyl)carbamate (S29)**

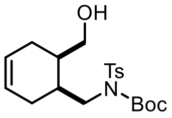

Following **GP8**, **S28** (1.02 g, 2.0 mmol, 1.0 equiv.) and CSA (47 mg, 0.2 mmol, 0.1 equiv.) gave **S29** (760 mg, 96%) as an oil. R_f 0.26 [petrol–EtOAc (2:1)]; ^{1}H NMR (400 MHz, $CDCl_3$) δ 7.73 (2H, d, *J* = 8.2 Hz), 7.28 (2H, d, *J* = 8.2 Hz), 5.72–5.59 (2H, m), 3.92 (1H, dd, *J* = 14.3, 9.0 Hz), 3.79 (1H, dd, *J* = 14.3, 3.9 Hz), 3.76–3.68 (1H, m), 3.63–3.52 (1H, m), 2.63–2.55 (1H, m), 2.42 (3H, s), 2.40–2.34 (1H, m), 2.14–1.99 (4H, m), 1.90–1.77 (1H, m), 1.28 (9H, s); ^{13}C NMR (101 MHz, $CDCl_3$) δ 151.6, 144.3, 137.4, 129.4, 127.8, 125.7 (2 x C), 84.8, 63.4, 46.7, 37.9, 34.2, 27.9, 27.4, 25.9, 21.7; HRMS (ASAP): Found MH^+ 396.1841, $C_{20}H_{30}O_5NS$ requires 396.1839.

***cis*-*tert*-Butyl ((6-formylcyclohex-3-en-1-yl)methyl)(tosyl)carbamate (35s)**

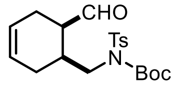

Following **GP6**, **S29** (751 mg, 1.9 mmol, 1.0 equiv.), pyridine (1.5 mL, 19.0 mmol, 10.0 equiv.) and DMP (2.42 g, 5.7 mmol, 3.0 equiv.) gave **35s** (600 mg, 80%) as an oil. R_f 0.32 [petrol–EtOAc (4:1)]; ^{1}H NMR (400 MHz, $CDCl_3$) δ 9.79 (1H, s), 7.72 (2H, d, *J* = 8.2 Hz), 7.28 (2H, d, *J* = 8.2 Hz), 5.75–5.63 (2H, m), 3.98 (1H, dd, *J* = 14.4, 7.9 Hz), 3.88 (1H, dd, *J* = 14.4, 6.1 Hz), 2.78–2.70 (1H, m), 2.65–2.54 (1H, m), 2.45–2.35 (1H, m), 2.41 (3H, s), 2.32–2.14 (3H, m), 1.29 (9H, s); ^{13}C NMR (101 MHz, $CDCl_3$) δ 203.9, 151.2, 144.4, 137.2, 129.3, 127.8, 126.4, 124.6, 84.6, 48.8, 47.1, 35.0, 27.9, 27.1, 24.1, 21.7; HRMS (ASAP): Found M-H^+ 392.1538, $C_{20}H_{26}O_5NS$ requires 392.1537.

Preparation of 36s

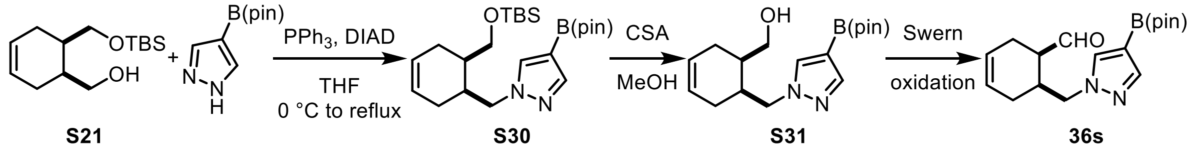

***cis*-1-((6-(((*tert*-Butyldimethylsilyl)oxy)methyl)cyclohex-3-en-1-yl)methyl)-4-(4,4,5,5-tetramethyl-1,3,2-dioxaborolan-2-yl)-1*H*-pyrazole (S30)**

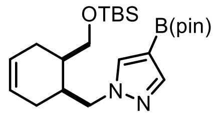

Following **GP7**, **S21** (769 mg, 3.0 mmol, 1.0 equiv.), DIAD (0.71 mL, 3.6 mmol, 1.2 equiv.), PPh_3 (944 mg, 3.6 mmol, 1.2 equiv.) and 4-pyrazoleboronic acid pinacol ester (698 mg, 3.6 mmol, 1.2 equiv.) gave **S30** (1.00 g, 77%) as an oil. R_f 0.26 [petrol–EtOAc (8:1)]; ^{1}H NMR (500 MHz, $CDCl_3$) δ 7.77 (1H, s), 7.65 (1H, s), 5.67–5.61 (1H, m), 5.60–5.53 (1H, m), 4.23 (1H, dd, *J* = 13.6, 5.2 Hz), 4.07 (1H, dd, *J* = 13.6, 10.0 Hz), 3.68 (1H, dd, *J* = 10.2, 6.9 Hz), 3.50 (1H, dd, *J* = 10.2, 6.9 Hz), 2.56–2.45 (1H, m), 2.20–2.10 (1H, m), 2.08–2.00 (1H, m), 1.99–1.87 (2H, m), 1.79–1.69 (1H, m), 1.30 (12H, s), 0.88 (9H, s), 0.04 (6H, s); ^{13}C NMR (126 MHz, $CDCl_3$) δ 145.5, 136.8, 126.0, 125.0, 83.3, 64.2, 53.2, 37.7, 36.2, 27.1, 26.8, 26.1, 25.0, 24.9, 18.4, –5.3 (2 x C); ^{11}B NMR (128 MHz, $CDCl_3$) δ 29.4; HRMS (ESI): Found MNa^+ 455.2857, $C_{23}H_{41}O_3N_2BNaSi$ requires 455.2872.

***cis*-6-((4-(4,4,5,5-Tetramethyl-1,3,2-dioxaborolan-2-yl)-1*H*-pyrazol-1-yl)methyl)cyclohex-3-en-1-yl)methanol (S31)**

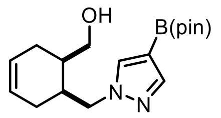

Following **GP8**, **S30** (994 mg, 2.3 mmol, 1.0 equiv.) and CSA (53 mg, 0.23 mmol, 0.1 equiv.) gave **S31** (540 mg, 74%) as an oil. R_f 0.32 [petrol–EtOAc (1:1)]; ^{1}H NMR (400 MHz, $CDCl_3$) δ 7.77 (1H, s), 7.66 (1H, s), 5.70–5.63 (1H, m), 5.63–5.57 (1H, m), 4.30 (1H, dd, *J* = 13.7, 6.7 Hz), 3.99 (1H, dd, *J* = 13.7, 8.2 Hz), 3.67–3.49 (3H, m), 2.59–2.49 (1H, m), 2.14–1.96 (3H, m), 1.90–1.77 (2H, m), 1.30 (12H, s); ^{13}C NMR (101 MHz, $CDCl_3$) δ 145.6, 137.0, 126.0, 125.1, 83.4, 63.5, 52.5, 37.2, 35.2, 27.6, 26.5, 24.9 (2 x C); ^{11}B NMR (128 MHz, $CDCl_3$) δ 29.3; HRMS (ESI): Found MNa^+ 341.1995, $C_{17}H_{27}O_3N_2BNa$ requires 341.2007.

***cis*-6-((4-(4,4,5,5-Tetramethyl-1,3,2-dioxaborolan-2-yl)-1*H*-pyrazol-1-yl)methyl)-3-cyclohexene-1-carbaldehyde (36s)**

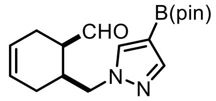

Following **GP5**, **S31** (318 mg, 1.0 mmol, 1.0 equiv.) gave **36s** (200 mg, 63%) as an oil. R_f 0.32 [petrol–EtOAc (2:1)]; ^{1}H NMR (400 MHz, $CDCl_3$) δ 9.62 (1H, s), 7.78 (1H, s), 7.67 (1H, s), 5.79–5.71 (1H, m), 5.71–5.65 (1H, m), 4.27 (1H, dd, *J* = 13.6, 7.6 Hz), 4.16 (1H, dd, *J* = 13.6, 7.9 Hz), 2.82–2.70 (1H, m), 2.52–2.44 (1H, m), 2.43–2.25 (2H, m), 2.16–2.05 (1H, m), 2.05–1.92 (1H, m), 1.30 (12H, s); ^{13}C NMR (101 MHz, $CDCl_3$) δ 204.1, 146.0, 137.2, 126.5, 124.9, 83.4, 53.2, 46.4, 35.3, 27.0, 25.0, 24.9, 24.3; ^{11}B NMR (128 MHz, $CDCl_3$) δ 29.3; HRMS (ESI): Found MNa^+ 339.1839, $C_{17}H_{25}O_3N_2BNa$ requires 339.1850.

Preparation of 37s

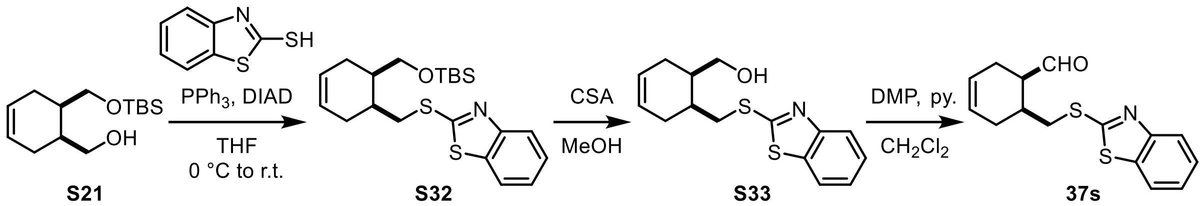

***cis*-2-(((6-(((*tert*-Butyldimethylsilyl)oxy)methyl)cyclohex-3-en-1-yl)methyl)thio)benzo[*d*]thiazolee (S32)**

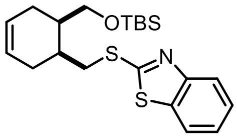

Following **GP7**, **S21** (769 mg, 3.0 mmol, 1.0 equiv.), DIAD (0.71 mL, 3.6 mmol, 1.2 equiv.), PPh_3 (944 mg, 3.6 mmol, 1.2 equiv.) and 2-mercaptobenzothiazole (602 mg, 3.6 mmol, 1.2 equiv.) gave **S32** (1.16 g, 95%) as an oil. R_f 0.21 [petrol–EtOAc (4:1)]; ^{1}H NMR (400 MHz, $CDCl_3$) δ 7.81 (1H, dd, *J* = 8.2, 1.2 Hz), 7.69 (1H, dd, *J* = 8.0, 1.2 Hz), 7.36 (1H, ddd, *J* = 8.2, 7.2, 1.2 Hz), 7.23 (1H, ddd, *J* = 8.0, 7.2, 1.2 Hz), 5.67–5.60 (1H, m), 5.60–5.54 (1H, m), 3.71 (1H, dd, *J* = 10.2, 6.8 Hz), 3.58 (1H, dd, *J* = 10.2, 6.8 Hz), 3.51 (1H, dd, *J* = 12.8, 4.9 Hz), 3.23 (1H, dd, *J* = 12.8, 9.4 Hz), 2.34–2.18 (2H, m), 2.18–2.01 (3H, m), 1.99–1.87 (1H, m), 0.86 (9H, s), 0.03 (6H, s); ^{13}C NMR (101 MHz, $CDCl_3$) δ 167.7, 153.5, 135.3, 126.1, 126.0, 125.2, 124.2, 121.6, 121.0, 64.0, 38.8, 35.1, 34.9, 29.1, 26.6, 26.1, 18.4, –5.2; HRMS (ASAP): Found MH^+ 406.1695, $C_{21}H_{32}ONS_2Si$ requires 406.1689.

***cis*-(6-((Benzo[*d*]thiazol-2-ylthio)methyl)-3-cyclohexene-1-methanol (S33)**

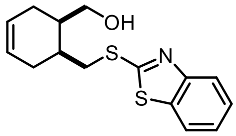

Following **GP8**, **S32** (974 mg, 2.4 mmol, 1.0 equiv.) and CSA (56 mg, 0.24 mmol, 0.1 equiv.) gave **S33** (650 mg, 93%) as a solid. R_f 0.30 [petrol–EtOAc (2:1)]; ^{1}H NMR (400 MHz, $CDCl_3$) δ 7.86 (1H, dd, *J* = 8.2, 1.4 Hz), 7.69 (1H, dd, *J* = 8.1, 1.3 Hz), 7.38 (1H, ddd, *J* = 8.2, 7.2, 1.4 Hz), 7.26 (1H, ddd, *J* = 8.1, 7.2, 1.3 Hz), 5.75–5.65 (1H, m), 5.65–5.57 (1H, m), 4.93 (1H, dd, *J* = 9.8, 4.2 Hz), 3.84–3.67 (3H, m), 2.65 (1H, dd, *J* = 13.2, 10.9 Hz), 2.61–2.54 (1H, m), 2.25–2.20 (2H, m), 2.19–2.11 (1H, m), 2.02–1.90 (1H, m), 1.76–1.61 (1H, m); ^{13}C NMR (101 MHz, $CDCl_3$) δ 168.7, 152.5, 134.5, 126.3, 126.0, 124.9, 124.5, 121.0 (2 x C), 64.0, 38.5, 32.1, 31.1, 29.0, 24.3; HRMS (ASAP): Found MH^+ 292.0817, $C_{15}H_{18}ONS_2$ requires 292.0824.

***cis*-6-((Benzo[*d*]thiazol-2-ylthio)methyl)-3-cyclohexene-1-carbaldehyde (37s)**

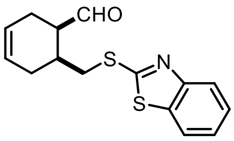

Following **GP6**, **S33** (582 mg, 2.0 mmol, 1.0 equiv.), pyridine (1.6 mL, 20.0 mmol, 10.0 equiv.) and DMP (2.54 g, 6.0 mmol, 3.0 equiv.) gave **37s** (440 mg, 76%) as an oil. R_f 0.32 [petrol–EtOAc (8:1)]; ^{1}H NMR (500 MHz, $CDCl_3$) δ 9.85 (1H, s), 7.85 (1H, dd, *J* = 8.2, 1.4 Hz), 7.74 (1H, dd, *J* = 8.1,1.2 Hz), 7.40 (1H, ddd, *J* = 8.2, 7.2, 1.4 Hz), 7.28 (1H, ddd, *J* = 8.1, 7.2, 1.2 Hz), 5.78–5.72 (1H, m), 5.72–5.67 (1H, m), 3.52 (1H, dd, *J* = 13.6, 6.2 Hz), 3.34 (1H, dd, *J* = 13.6, 8.3 Hz), 2.81 (1H, td, *J* = 6.1, 3.1 Hz), 2.71–2.61 (1H, m), 2.44–2.28 (3H, m), 2.28–2.20 (1H, m); ^{13}C NMR (126 MHz, $CDCl_3$) δ 204.1, 166.4, 153.2, 135.3, 126.1 (2 x C), 124.9, 124.4, 121.6, 121.1, 48.4, 35.0, 34.1, 29.1, 23.7; HRMS (ASAP): Found MH^+ 290.0664, $C_{15}H_{16}ONS_2$ requires 290.0668.

Preparation of 39

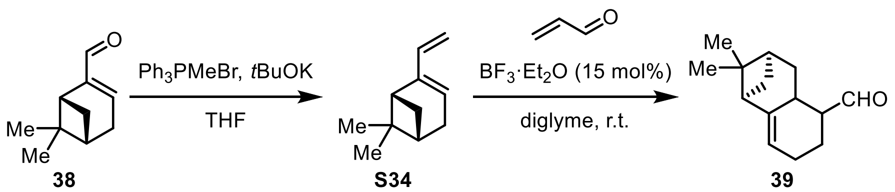

(-)-(1*R*,5*S*)-Nopadiene (S34)

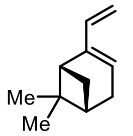

A suspension of methyltriphenylphosphonium bromide (7.74 g, 21.6 mmol, 1.1 equiv.) in anhydrous THF (50 mL) was treated with *t*-BuOK (2.42 g, 21.6 mmol, 1.1 equiv.) and stirred at r.t for 30 mins. Then (–)-myrtenal **38** (3.0 mL, 19.72 mmol, 1.0 equiv) was added and the reaction mixture was stirred at room temperature for 24 h. The reaction was diluted with pentane (50 mL) and filtered through a plug of celite, eluting with more pentane. The filtrate was evaporated, and the crude was purified by column chromatography on silica gel eluting with pentane to give **S34** (2.50 g, 86%) as an oil. ^{1}H NMR (400 MHz, $CDCl_3$) δ 6.36 (1H, dd, *J* = 17.4, 10.7 Hz), 5.60–5.51 (1H, m), 5.05 (1H, dd, *J* = 17.4, 0.4 Hz), 4.89 (1H, d, *J* = 10.7 Hz), 2.57 (1H, td, *J* = 5.7, 1.5 Hz), 2.46–2.27 (3H, m), 2.16–2.09 (1H, m), 1.33 (3H, s), 1.14 (1H, d, *J* = 8.8 Hz), 0.80 (3H, s); ^{13}C NMR (101 MHz, $CDCl_3$) δ 146.9, 138.0, 124.6, 109.7, 41.2, 40.4, 37.8, 32.0, 31.4, 26.5, 20.9. Data in accordance with the literature.[19]

(1*R*,3*S*)-2,2-dimethyl-1,2,3,4,4a,5,6,7-octahydro-1,3-methanonaphthalene-5-carbaldehyde (39)

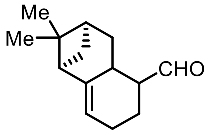

Following **GP1**, (–)-(1*R*,5*S*)-nopadiene **S34** (297 mg, 2.0 mmol, 1.0 equiv.) and acrolein (0.2 mL, 3.0 mmol, 1.5 equiv.) gave **39** (163 mg, 40%) as an inseparable mixture of diastereomers as an oil. d.r. = 9:1.6:1. R_f 0.50 [petrol–EtOAc (20:1)]; ^{1}H NMR (400 MHz, $CDCl_3$, diastereomers) δ 9.92 (0.8H, d, *J* = 2.8 Hz), 9.69 (0.1H, d, *J* = 3.4 Hz), 9.64 (0.1H, d, *J* = 1.7 Hz), 5.26–5.16 (1H, m), 3.08–2.76 (1H, m), 2.56–2.42 (3H, m), 2.25–2.04 (5H, m), 2.02–1.82 (2H, m), 1.25 (3H, s), 1.01–0.94 (1H, m) 0.98 (3H, s); ^{13}C NMR (101 MHz, $CDCl_3$, diastereomers) δ 206.7, 205.3, 203.9, 147.1, 144.0, 143.5, 118.3, 117.4, 115.2, 56.4, 52.3, 52.0 (2 x C), 48.9, 48.1, 42.2, 42.1, 42.0, 40.3, 39.7, 39.6, 35.9, 35.5, 34.9, 33.3, 33.0, 32.8,

32.2, 32.0, 31.7, 30.0, 27.2 (2 x C), 25.4, 24.0 (2 x C), 23.8 (2 x C), 23.1, 23.0, 22.9, 21.7; HRMS (ESI): Found MNa^+ 227.1400, $C_{14}H_{20}ONa$ requires 227.1406.

Preparation of 42

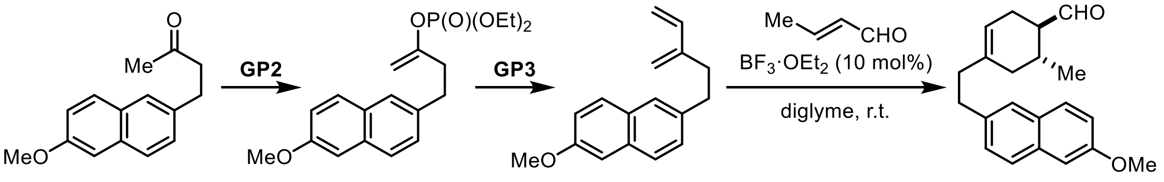

41 **S35** **S36** **42**

Diethyl (4-(6-methoxynaphthalen-2-yl)but-1-en-2-yl) phosphate (S35)

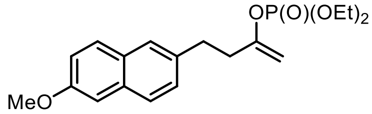

Following **GP2** using LDA instead of NaHMDS, nabumetone **41** (3.42 g, 15.0 mmol, 1.0 equiv.), LDA (2M, 8.25 mL, 16.5 mmol, 1.1 equiv.) and $(EtO)_2P(O)Cl$ (3.26 mL, 22.5 mmol, 1.5 equiv.) gave **S35** (3.19 g, 58%) as an oil. R_f 0.2 [EtOAc–pentane (2:3)]; ^{1}H NMR (400 MHz, $CDCl_3$) δ 7.68 (1H, s), 7.66 (1H, s), 7.57 (1H, br s), 7.30 (1H, dd, *J* = 8.4, 1.8 Hz), 7.15–7.08 (2H, m), 4.85 (1H, t, *J* = 2.1 Hz), 4.52 (1H, tt, *J* = 2.0, 0.9 Hz), 4.22–4.13 (4H, m), 3.91 (s, 3H), 3.00–2.92 (2H, m), 2.62–2.54 (2H, m), 1.36 (6H, td, *J* = 7.1, 1.0 Hz); ^{13}C NMR (101 MHz, $CDCl_3$) δ 157.41, 155.07, 136.12, 133.25, 129.18, 129.08, 127.76, 126.97, 126.53, 118.91, 105.77, 97.61 (d, *J* = 4.2 Hz), 64.45 (d, *J* = 6.0 Hz), 55.44, 36.57 (d, *J* = 5.5 Hz), 32.95, 16.26 (d, *J* = 6.8 Hz); ^{31}P NMR (162 MHz, $CDCl_3$) δ -6.46–-6.74 (1P, m); HRMS (ASAP): Found MH^+ 365.1503, $C_{19}H_{25}O_5P$ requires 365.1518.

2-Methoxy-6-(3-methylenepent-4-en-1-yl)naphthalene (S36)

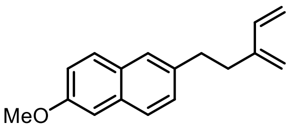

Following **GP3**, **S35** (3.18 g, 8.75 mmol, 1.0 equiv.), $Ni(dppe)Cl_2$ (116 mg, 2.5 mol%) and vinyl magnesium bromide in THF (1 M, 9.2 mL, 9.2 mmol, 1.05 equiv.) gave **S36** (1.33 g, 64%) as a solid. R_f 0.17 [CH_2Cl_2–petrol (1:9)]; ^{1}H NMR (500 MHz, $CDCl_3$) δ 7.69 (1H, s), 7.67 (1H, s), 7.57 (1H, s), 7.32 (1H, dd, *J* = 8.4, 1.8 Hz), 7.16–7.09 (2H, m), 6.43 (1H, dd, *J* = 17.9, 10.6 Hz), 5.32 (1H, d, *J* = 17.7 Hz), 5.11 (1H, d, *J* = 11.3 Hz), 5.05 (1H, s), 5.03 (1H, s), 3.92 (3H, s), 2.98–2.88 (2H, m), 2.63–2.56 (2H, m); ^{13}C NMR (126 MHz, $CDCl_3$) δ 157.3, 145.9, 139.0, 137.5, 133.2, 129.2, 129.1, 127.9, 126.9, 126.4, 118.8, 116.3, 113.5, 105.8, 55.4, 34.7, 33.5; HRMS (ASAP): Found MH^+ 239.1419, $C_{17}H_{19}O$ requires 239.1436.

4-(2-(6-Methoxynaphthalen-2-yl)ethyl)-*trans*-6-methyl-3-cyclohexene-1-carbaldehyde (42)

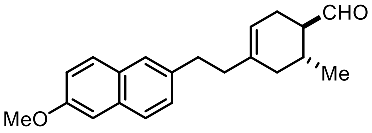

Following **GP1**, **S36** (0.48 g, 2 mmol, 1 equiv.), crotonaldehyde (0.25 mL, 3 mmol, 1.5 equiv.), $BF_3 \cdot Et_2O$ (0.025 mL, 10 mol%) gave **42** (0.30 g, 49%) as a solid. R_f 0.29 [EtOAc–Pentane (5:95)]; ^1H NMR (500 MHz, $CDCl_3$) δ 9.62 (1H, d, *J* = 2.9 Hz), 7.67 (1H, d, *J* = 4.4 Hz), 7.65 (1H, d, *J* = 4.4 Hz), 7.52 (1H, s), 7.31–7.26 (1H, m), 7.15–7.09 (2H, m), 5.46–5.33 (1H, m), 3.91 (3H, s), 2.86–2.78 (2H, m), 2.37–2.28 (2H, m), 2.28–2.20 (1H, m), 2.20–2.06 (4H, m), 1.82–1.72 (1H, m), 1.04 (3H, d, *J* = 6.4 Hz); ^{13}C NMR (126 MHz, $CDCl_3$) δ 205.5, 157.3, 137.4, 136.8, 133.1, 129.2, 129.0, 127.9, 126.8, 126.3, 118.8, 118.3, 105.8, 55.4, 52.6, 39.5, 35.5, 34.4, 28.5, 24.1, 19.9; HRMS (ESI): Found MNa^+ 331.1654, $C_{21}H_{24}O_2Na$ requires 331.1674.

Preparation of 45

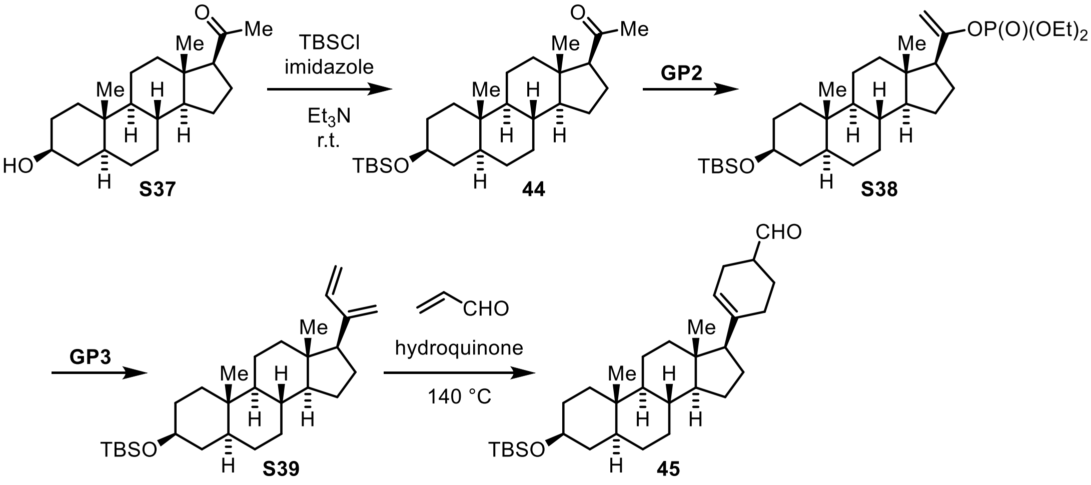

3β-*tert*-Butyldimethylsilyloxy-5α,17β-pregnan-20-one (44)

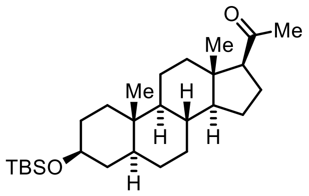

A dry flask was charged with imidazole (1.53 g, 15.0 mmol, 1.0 equiv.), allopregnanolone **S37** (4.77 g, 15.0 mmol, 1.0 equiv.) and TBSCl (2.81 g, 18.8 mmol, 1.25 equiv.). The flask was evacuated and refilled with N_2 twice and DMF (135 mL) was added. The reaction was stirred at r.t for 10 h and H_2O (30 mL) was added. The layers were separated, and the aqueous layer was extracted with Et_2O (3 x 30 mL). The combined organic layers were washed with saturated aqueous $NaHCO_3$ (30 mL), H_2O (5 x 20 mL), brine (30 mL) and dried ($MgSO_4$). The solvent was evaporated to give **44** (5.24 g, 81%) as a solid. ^{1}H NMR (400 MHz, $CDCl_3$) δ 3.60–3.49 (1H, m), 2.51 (1H, t, *J* = 9.0 Hz), 2.21–2.12 (1H, m), 2.11 (3H, s), 1.99 (1H, dt, *J* = 11.4, 3.2 Hz), 1.75–1.01 (17H, m), 1.01–0.89 (2H, m), 0.89 (9H, s), 0.79 (3H, s), 0.69–0.62 (1H, m), 0.59 (3H, s), 0.05 (6H, s). Data in accordance with literature.[20]

1-((3*S*,5*S*,8*R*,9*S*,10*S*,13*S*,14*S*,17*S*)-3-((*tert*-Butyldimethylsilyl)oxy)-10,13-dimethylhexadecahydro-1*H*-cyclopenta[*a*]phenanthren-17-yl)vinyl diethyl phosphate (S38)

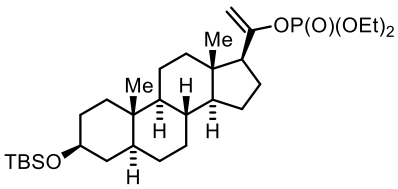

Following **GP2** using LDA instead of NaHMDS, **44** (5.24 g, 12.1 mmol, 1.0 equiv.), LDA (2M, 6.66 mL, 13.3 mmol, 1.1 equiv.) and $(EtO)_2P(O)Cl$ (3.26 mL, 22.5 mmol, 1.5 equiv.) gave **S38** (5.01 g, 73%) as a solid. R_f 0.25 [acetone–petrol (1:9)]; ^{1}H NMR (500 MHz, $CDCl_3$) δ 4.90 (1H, t, *J* = 2.1 Hz), 4.49–4.44 (1H, m), 4.16–4.05 (4H, m), 3.50 (1H, tt, *J* = 10.9, 4.6 Hz), 2.16 (1H, t, *J* = 9.4 Hz), 1.89 (1H, dt, *J* = 12.5, 3.2 Hz), 1.80–1.67 (1H, m), 1.67–1.57 (5H, m), 1.49 (1H, dq, *J* = 13.2, 3.7 Hz), 1.45–1.32 (2H, m), 1.30 (7H, td, *J* = 7.0, 1.1 Hz), 1.27–1.07 (6H, m), 1.05–0.97 (2H, m), 0.93–0.81 (2H, m), 0.85 (9H, s), 0.75 (3H, s), 0.60 (3H, s), -0.00 (6H, s); ^{13}C NMR (126 MHz, $CDCl_3$) δ 156.6 (d, *J* = 9.5 Hz), 96.8 (d, *J* = 3.4 Hz), 72.2, 64.3–63.9 (m), 55.8, 54.9 (d, *J* = 6.3 Hz), 54.6, 45.1, 43.5, 38.7, 38.5, 37.2, 35.8, 35.6, 32.1, 32.0, 28.8, 26.0, 24.6, 24.2, 21.2, 18.3, 16.2 (d, *J* = 6.8 Hz), 12.9, 12.4, -4.5; ^{31}P NMR (162 MHz, $CDCl_3$) δ -6.99 (p, *J* = 8.4 Hz); HRMS (ESI): Found MNa^+ 591.3590, $C_{31}H_{57}O_5PSiNa$ requires 591.3611.

(((3*S*,5*S*,8*R*,9*S*,10*S*,13*S*,14*S*,17*R*)-17-(Buta-1,3-dien-2-yl)-10,13-dimethylhexadecahydro-1*H*-cyclopenta[*a*]phenanthren-3-yl)oxy)(*tert*-butyl)dimethylsilane (S39)

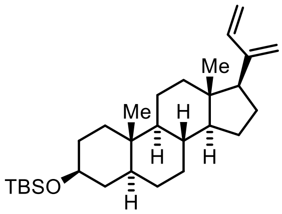

Following **GP3**, **S38** (5.01 g, 8.80 mmol), $Ni(dppe)Cl_2$ (116 mg, 2.5 mol%) and vinyl magnesium bromide in THF (1 M, 9.63 mL, 9.63 mmol, 1.05 equiv.) gave **S39** (1.91 g, 49%) as a solid. R_f 0.81 [CH_2Cl_2–pentane (1:1)]; ^{1}H NMR (400 MHz, $CDCl_3$) δ 6.34 (1H, dd, *J* = 17.1, 11.4 Hz), 5.35–5.26 (1H, m), 5.21 (1H, s), 5.03–4.90 (2H, m), 3.54 (1H, tt, *J* = 11.0, 4.7 Hz), 2.43 (1H, t, *J* = 9.4 Hz), 1.83–1.71 (2H, m), 1.74–1.59 (6H, m), 1.50 (1H, dd, *J* = 13.3, 3.6 Hz), 1.47–1.40 (2H, m), 1.39–1.29 (1H, m), 1.29–1.22 (3H, m), 1.17 (2H, td, *J* = 10.8, 4.9 Hz), 1.13–0.99 (2H, m), 0.99–0.87 (2H, m), 0.88 (9H, s), 0.79 (3H, s), 0.69–0.60 (1H, m), 0.54 (3H, s), 0.05 (6H, s); ^{13}C NMR (101 MHz, $CDCl_3$) δ 146.4, 141.2, 114.6, 112.8, 72.3,

56.8, 54.8, 51.6, 45.3, 43.6, 39.3, 38.9, 37.4, 36.3, 35.7, 32.3, 32.1, 28.9, 26.5, 26.1, 24.5, 21.4, 18.4, 13.2, 12.6, -4.4; HRMS (ASAP): Found MH^+ 443.3693, $C_{29}H_{51}OSi$ requires 443.3709.

4-((3*S*,5*S*,8*R*,9*S*,10*S*,13*S*,14*S*,17*R*)-3-((*tert*-Butyldimethylsilyl)oxy)-10,13-dimethylhexadecahydro-1*H*-cyclopenta[*a*]phenanthren-17-yl)-3-cyclohexene-1-carbaldehyde (45)

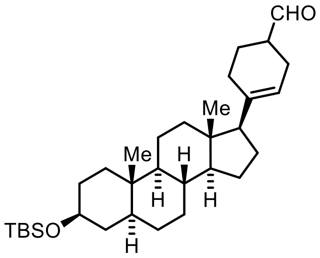

To a microwave vial equipped with a stir bar hydroquinone (2 mg, 0.22 mmol, 0.1 equiv.) and diene **S39** (0.54 g, 2.0 mmol, 1.0 equiv.) were added. The vial was capped and evacuated and refilled with N_2 twice. Acrolein (0.13 mL, 4.0 mmol, 2.0 equiv.) was added and the reaction vessel was heated to 50 ºC for 12 h. The reaction was then removed from the heating block and allowed to cool to room temperature. The contents of the vial were transferred to an RBF and the volatiles were removed *in vacuo* to give the product (0.465 g, 47%), a mixture of regioisomers each with two diastereomers, as a solid. The major regisomer was separated via column chromatography on silica gel eluting with Et_2O-pentane (5:95) to give **45** (0.167 g, 17%, d.r. 1:1), an inseparable mixture of diastereomers, as a solid. R_f 0.21 [Et_2O–Pentane (5:95)]; ^1H NMR (500 MHz, $CDCl_3$, diastereomers) δ 9.68 (0.5H, d, J = 3.1 Hz), 9.67 (0.5H, d, J = 3.4 Hz), 5.47–5.40 (2H, m), 3.53 (2H, tt, J = 11.0, 4.6 Hz), 2.50–2.39 (1H, m), 2.36–2.19 (2H, m), 2.17–2.07 (1H, m), 2.00–1.84 (3H, m), 1.81–1.48 (9H, m), 1.47–1.37 (2H, m), 1.37–1.19 (5H, m), 1.16–1.00 (4H, m), 0.96–0.83 (2H, m), 0.87 (9H, s), 0.78 (1.5H, s), 0.78 (1.5H, s), 0.65–0.57 (1H, m), 0.50 (1.5H, s), 0.49 (1.5H, s), 0.04 (6H, s); ^{13}C NMR (126 MHz, $CDCl_3$, diastereomers) δ 204.9, 204.8, 137.9, 137.8, 119.2, 119.2, 72.3, 57.7, 57.4, 56.3, 56.2, 54.8, 46.7, 45.9, 45.2, 43.8, 43.7, 39.2, 39.1, 38.8, 37.3, 36.0, 35.9, 35.7, 32.2, 32.1, 29.4, 28.9, 28.5, 26.1, 24.9, 24.9, 24.7, 24.4, 24.3, 24.2, 23.0, 22.8, 21.4, 18.4, 13.3, 13.1, 12.5, -4.4; HRMS (ESI): Found $[M-H]^+$ 497.3802, $C_{32}H_{53}O_2Si$ requires 497.3820.

(*E*)-3-(Cyclohex-3-en-1-yl)acrylaldehyde (49)

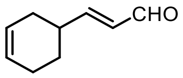

A solution of (triphenylphosphoranylidene)-acetaldehyde (1.83 g, 6.0 mmol, 1.2 equiv.) and 3-cyclohexene-1-carbaldehyde **1** (0.59 mL, 5.0 mmol, 1.0 equiv.) in $CHCl_3$ (16 mL, 0.3 M) was refluxed for 24 hours. The solvent was evaporated, and the crude was purified by flash column chromatography on silica gel eluting with petrol–EtOAc (20:1) to give **49** (249 mg, 37%) as a pale-yellow oil. ^{1}H NMR (400 MHz, $CDCl_3$) δ 9.51 (1H, d, *J* = 7.8 Hz), 6.84 (1H, dd, *J* = 15.7, 6.8 Hz), 6.11 (1H, ddd, *J* = 15.7, 7.8, 1.4 Hz), 5.76–5.63 (2H, m), 2.63–2.52 (1H, m), 2.26–2.15 (1H, m), 2.15–2.06 (2H, m), 2.01–1.83 (2H, m), 1.56–1.43 (1H, m); ^{13}C NMR (101 MHz, $CDCl_3$) δ 194.5, 162.8, 131.2, 127.2, 125.1, 36.9, 30.0, 27.4, 24.4. Data in accordance with the literature.[21]

(*Z*)-4-(Cyclohex-3-en-1-ylidenemethyl)morpholine (3)

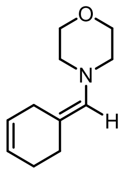

3-cyclohexene-1-carbaldehyde **1** (2.34 mL, 20.0 mmol, 1.0 equiv.) was added to a flame-dried RBF flushed with N_2 and equipped with a Dean-Stark apparatus. Morpholine (1.9 mL, 22 mmol, 1.1 equiv.) and toluene (50 mL) were added, and the reaction was heated under reflux for 4 h. The solvent was removed *in vacuo* and the impurities were distilled from the product (20 mbar at 80 ^{o}C) to give **3** (2.89 g, 81%) as a solid. The relative configuration was assigned by NOESY experiment. ^{1}H NMR (400 MHz, C_6D_6) δ 5.76–5.61 (2H, m), 5.31 (1H, s), 3.58–3.54 (4H, m), 2.89–2.84 (2H, m), 2.44–2.36 (4H, m), 2.14 (2H, t, *J* = 6.3 Hz), 2.08–1.99 (2H, m); ^{13}C NMR (101 MHz, C_6D_6) δ 134.2, 127.1, 126.34, 126.2, 66.9, 53.1, 29.6, 28.0, 27.4; HRMS (ESI): Found MH^+ 180.1385, $C_{11}H_{18}NO$ requires 180.1388.

3 Reaction Optimizations

General Procedure for the Reaction Optimization – GP9

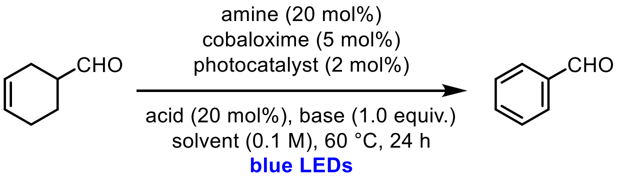

A dry tube equipped with a stirring bar was charged with the photocatalyst (2 μmol, 2 mol%), the cobaloxime (5 μmol, 5 mol%), the amine (if solid, 0.02 mmol, 20 mol%), the base (if solid, 0.10 mmol, 1.0 equiv.) and acid (if solid, 0.02 mmol, 20 mol%). The tube was capped with a Supelco aluminium crimp seal with septum (PTFE/butyl), then evacuated under high vacuum and backfilled with N_2 (x 3). Degassed solvent (1.0 mL, 0.1 M), **1** (12 μL, 0.10 mmol, 1.0 equiv.), the amine (if liquid, 0.02 mmol, 20 mol%), the base (if liquid, 0.10 mmol, 1.0 equiv.) and the acid (if liquid, 0.02 mmol, 20 mol%) were sequentially added. The vial was purged with a stream of N_2, and the lid sealed with parafilm and placed approximately 4 cm from blue LEDs. The blue LEDs were switched on and the mixture was stirred under irradiation without fan for 24 hours. The tube was opened, and the mixture was quenched with H_2O (0.2 mL). 1,3,5-Trimethoxybenzene (1.0 mL, 1.0 equiv., 0.1 M solution in $CDCl_3$) was added. A 1.0 mL aliquot of the resulting mixture was passed through a short pad of $MgSO_4$ (anhydrous) directly into an NMR tube for analysis by ^1H NMR spectroscopy.

Table S1 details all the experiments performed.

Table S1

Entry	Photocatalyst	Cobaloxime	Amine	Base	Acid	Solvent	Yield (%)
**1**	Ir(ppy)$_3$	Co(dmgH)$_2$PyCl$_2$	morpholine	DABCO	AcOH	CH$_3$CN	14
**2**	Ir(Fppy)$_3$	Co(dmgH)$_2$PyCl$_2$	morpholine	DABCO	AcOH	CH$_3$CN	11
**3**	Ru(bpy)$_3$Cl$_2$	Co(dmgH)$_2$PyCl$_2$	morpholine	DABCO	AcOH	CH$_3$CN	9
**4**	4CzIPN	Co(dmgH)$_2$PyCl$_2$	morpholine	DABCO	AcOH	CH$_3$CN	35
**5**	Mes-Acr·BF$_4$	Co(dmgH)$_2$PyCl$_2$	morpholine	DABCO	AcOH	CH$_3$CN	4
**6**	(Ir[dF(CF$_3$)ppy]$_2$(dtbpy))PF$_6$	Co(dmgH)$_2$PyCl$_2$	morpholine	DABCO	AcOH	CH$_3$CN	22
**7**	Ir(dtbbpy)(ppy)$_2$]PF$_6$	Co(dmgH)$_2$PyCl$_2$	morpholine	DABCO	AcOH	CH$_3$CN	74
**8**	Ir(dtbbpy)(ppy)$_2$]PF$_6$	Co(dmgH)$_2$(DMAP)Cl	morpholine	DABCO	AcOH	CH$_3$CN	66
**9**	Ir(dtbbpy)(ppy)$_2$]PF$_6$	Co(dmgH)$_2$(4-CN-py)Cl	morpholine	DABCO	AcOH	CH$_3$CN	67
**10**	Ir(dtbbpy)(ppy)$_2$]PF$_6$	Co(dmgH)(dmgH$_2$)Cl$_2$	morpholine	DABCO	AcOH	CH$_3$CN	70
**11**	Ir(dtbbpy)(ppy)$_2$]PF$_6$	Co(dmgH)$_2$PyCl$_2$	piperidine	DABCO	AcOH	CH$_3$CN	59
**12**	Ir(dtbbpy)(ppy)$_2$]PF$_6$	Co(dmgH)$_2$PyCl$_2$	pyrrolidine	DABCO	AcOH	CH$_3$CN	24
**13**	Ir(dtbbpy)(ppy)$_2$]PF$_6$	Co(dmgH)$_2$PyCl$_2$	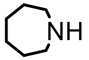	DABCO	AcOH	CH$_3$CN	39
**14**	Ir(dtbbpy)(ppy)$_2$]PF$_6$	Co(dmgH)$_2$PyCl$_2$	L-proline	DABCO	AcOH	CH$_3$CN	30
**15**	Ir(dtbbpy)(ppy)$_2$]PF$_6$	Co(dmgH)$_2$PyCl$_2$	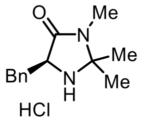	DABCO	AcOH	CH$_3$CN	3
**16**	Ir(dtbbpy)(ppy)$_2$]PF$_6$	Co(dmgH)$_2$PyCl$_2$	Cy$_2$NH	DABCO	AcOH	CH$_3$CN	26
**17**	Ir(dtbbpy)(ppy)$_2$]PF$_6$	Co(dmgH)$_2$PyCl$_2$	*i*Pr$_2$NH	DABCO	AcOH	CH$_3$CN	17

Entry	Photocatalyst	Cobaloxime	Amine	Base	Acid	Solvent	Yield (%)
**18**	Ir(dtbbpy)(ppy)$_2$]PF$_6$	Co(dmgH)$_2$PyCl$_2$	Et$_2$NH	DABCO	AcOH	CH_3CN	39
**19**	Ir(dtbbpy)(ppy)$_2$]PF$_6$	Co(dmgH)$_2$PyCl$_2$	BnNH$_2$	DABCO	AcOH	CH_3CN	27
**20**	Ir(dtbbpy)(ppy)$_2$]PF$_6$	Co(dmgH)$_2$PyCl$_2$	PhNH$_2$	DABCO	AcOH	CH_3CN	9
**21**	Ir(dtbbpy)(ppy)$_2$]PF$_6$	Co(dmgH)$_2$PyCl$_2$	morpholine	quinuclidine	AcOH	CH_3CN	47
**22**	Ir(dtbbpy)(ppy)$_2$]PF$_6$	Co(dmgH)$_2$PyCl$_2$	morpholine	2,6-lutidine	AcOH	CH_3CN	40
**23**	Ir(dtbbpy)(ppy)$_2$]PF$_6$	Co(dmgH)$_2$PyCl$_2$	morpholine	NaOAc	AcOH	CH_3CN	52
**24**	Ir(dtbbpy)(ppy)$_2$]PF$_6$	Co(dmgH)$_2$PyCl$_2$	morpholine	K_3PO_4	AcOH	CH_3CN	25
**25**	Ir(dtbbpy)(ppy)$_2$]PF$_6$	Co(dmgH)$_2$PyCl$_2$	morpholine	K_2HPO_4	AcOH	CH_3CN	56
**26**	Ir(dtbbpy)(ppy)$_2$]PF$_6$	Co(dmgH)$_2$PyCl$_2$	morpholine	K_2CO_3	AcOH	CH_3CN	3
**27**	Ir(dtbbpy)(ppy)$_2$]PF$_6$	Co(dmgH)$_2$PyCl$_2$	morpholine	$NaHCO_3$	AcOH	CH_3CN	19
**28**	Ir(dtbbpy)(ppy)$_2$]PF$_6$	Co(dmgH)$_2$PyCl$_2$	morpholine	DABCO	PTSA	CH_3CN	71
**29**	Ir(dtbbpy)(ppy)$_2$]PF$_6$	Co(dmgH)$_2$PyCl$_2$	morpholine	DABCO	TFA	CH_3CN	67
**30**	Ir(dtbbpy)(ppy)$_2$]PF$_6$	Co(dmgH)$_2$PyCl$_2$	morpholine	DABCO	HCO_2H	CH_3CN	18
**31**	Ir(dtbbpy)(ppy)$_2$]PF$_6$	Co(dmgH)$_2$PyCl$_2$	morpholine	DABCO	H_3PO_4	CH_3CN	52
**32**	Ir(dtbbpy)(ppy)$_2$]PF$_6$	Co(dmgH)$_2$PyCl$_2$	morpholine	DABCO	$HClO_4$	CH_3CN	70
**33**	Ir(dtbbpy)(ppy)$_2$]PF$_6$	Co(dmgH)$_2$PyCl$_2$	morpholine	DABCO	$PhCO_2H$	CH_3CN	72
**34**	Ir(dtbbpy)(ppy)$_2$]PF$_6$	Co(dmgH)$_2$PyCl$_2$	morpholine	DABCO	AcOH	DMSO	1
**35**	Ir(dtbbpy)(ppy)$_2$]PF$_6$	Co(dmgH)$_2$PyCl$_2$	morpholine	DABCO	AcOH	DMF	64
**36**	Ir(dtbbpy)(ppy)$_2$]PF$_6$	Co(dmgH)$_2$PyCl$_2$	morpholine	DABCO	AcOH	DMPU	6
**37**	Ir(dtbbpy)(ppy)$_2$]PF$_6$	Co(dmgH)$_2$PyCl$_2$	morpholine	DABCO	AcOH	MeOH	10

Entry	Photocatalyst	Cobaloxime	Amine	Base	Acid	Solvent	Yield (%)
**38**	Ir(dtbbpy)(ppy)$_2$]PF$_6$	Co(dmgH)$_2$PyCl$_2$	morpholine	DABCO	AcOH	THF	27
**39**	Ir(dtbbpy)(ppy)$_2$]PF$_6$	Co(dmgH)$_2$PyCl$_2$	morpholine	DABCO	AcOH	toluene	21
**40**	Ir(dtbbpy)(ppy)$_2$]PF$_6$	Co(dmgH)$_2$PyCl$_2$	morpholine	DABCO	AcOH	1,4-dioxane	20
**41**	Ir(dtbbpy)(ppy)$_2$]PF$_6$	Co(dmgH)$_2$PyCl$_2$	morpholine	DABCO	AcOH	EtOAc	27
**42**[a]	Ir(dtbbpy)(ppy)$_2$]PF$_6$	Co(dmgH)$_2$PyCl$_2$	morpholine	DABCO	AcOH	CH_3CN	69
**43**[b]	Ir(dtbbpy)(ppy)$_2$]PF$_6$	Co(dmgH)$_2$PyCl$_2$	morpholine	DABCO	AcOH	CH_3CN	59
			Control Experiments				
**44**	–	Co(dmgH)$_2$PyCl$_2$	morpholine	DABCO	AcOH	CH_3CN	2
**45**	Ir(dtbbpy)(ppy)$_2$]PF$_6$	–	morpholine	DABCO	AcOH	CH_3CN	–
**46**	Ir(dtbbpy)(ppy)$_2$]PF$_6$	Co(dmgH)$_2$PyCl$_2$	–	DABCO	AcOH	CH_3CN	5
**47**	Ir(dtbbpy)(ppy)$_2$]PF$_6$	Co(dmgH)$_2$PyCl$_2$	morpholine	–	AcOH	CH_3CN	33
**48**	Ir(dtbbpy)(ppy)$_2$]PF$_6$	Co(dmgH)$_2$PyCl$_2$	morpholine	DABCO	–	CH_3CN	70
**49**[c]	Ir(dtbbpy)(ppy)$_2$]PF$_6$	Co(dmgH)$_2$PyCl$_2$	morpholine	DABCO	AcOH	CH_3CN	–

(a) 4Å MS 100 mg; (b) H_2O 10 equiv.; (c) reaction run in the dark.

4 Picture of Reaction Set-Up

Pictures of set-up for 0.2 mmol scale reactions. Once the LEDs were turned on the entire reaction set up was wrapped in aluminium foil. In this way the reactions temperature remained uniform at around 60 ºC.

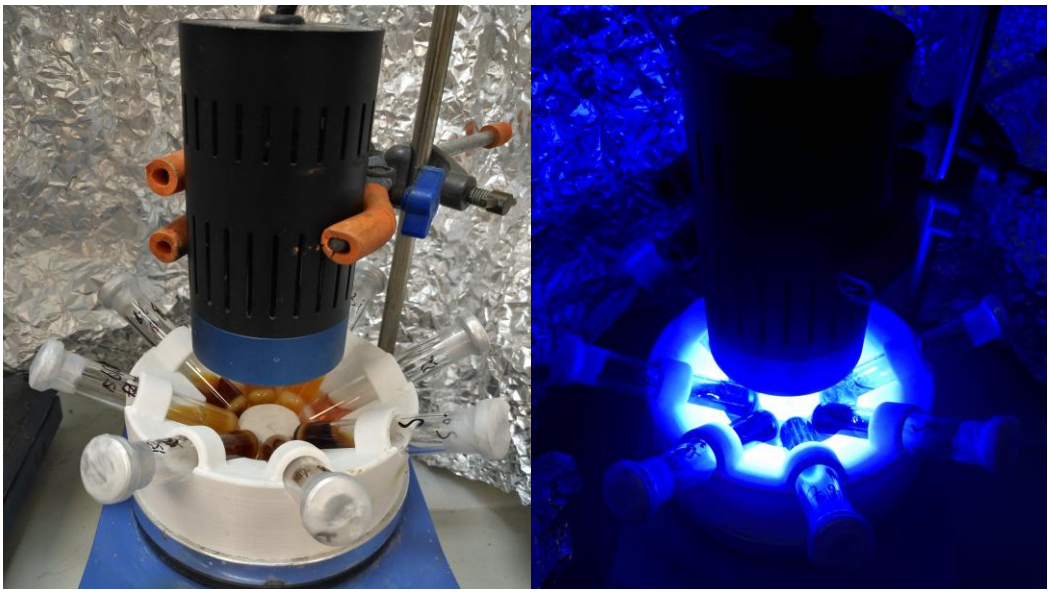

Figure S1

Picture of set-up for gram-scale scale reaction.

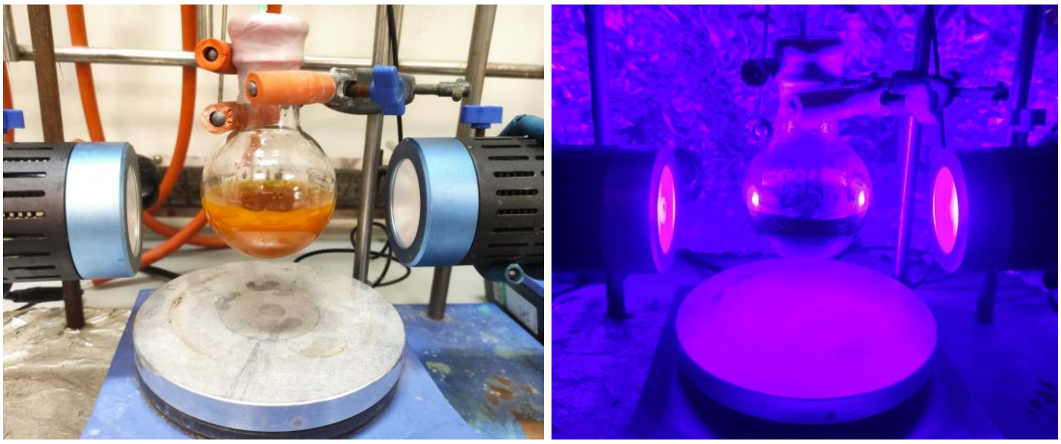

Figure S2

5 Reaction Performed at AstraZeneca

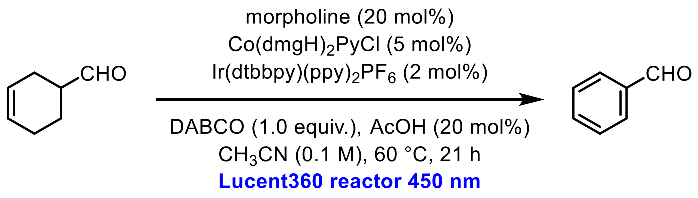

A 2 mL clear vial equipped with a stirring bar was charged with Co(dmgH)$_2$PyCl (2.02 mg, 5.0 µmol, 5 mol%) then taken into a glovebox. A stock solution was prepared within the glovebox containing the internal standard *m*-terphenyl (11.5 mg, 25 µmol, 0.5 equiv.), Ir(dtbbpy)(ppy)$_2$PF$_6$ (1.85 mg, 2.0 µmol, 2 mol%), DABCO (11.2 mg, 100 µmol, 1.0 equiv.), morpholine (1.74 µL, 20.0 µmol, 20 mol%), AcOH (1.15 µL, 20.0 µmol, 20 mol%) and 3-cyclohexene-1-carboxaldehyde (11.0 mg, 100 µmol, 1.0 equiv.) in degassed CH$_3$CN (1.0 mL, 0.1 M) and was added to the vial to give a light orange solution. Note the Co(dmgH)$_2$PyCl is not fully soluble at r.t. The vial was sealed with a PTFE lined screw cap and removed from the glovebox. Multiple reaction vials were arranged around a 2 mL x 24 reaction holder within a Lucent360 reactor with the internal coolant at 60 ± 5 °C. The vials were irradiated with 450 nm light for the given time before being removed from the reactor and sampled directly (50 µL into 1.5 mL 1:1 MeCN/Water) and analysed via UHPLC-MS at 254 nm. Yields were calculated vs *m*-terphenyl internal standard and corrected for UV response factor using authentic benzaldehyde product (99% w/w).

Entry	Time (h)	Yield (%)
**1**	2.3	51
**2**	3.5	60
**3**	21	68
**4**	21	71
**5**	21	70
**6**	21	74

Average yield at 21 h = 71%

6 Substrate Scope

General Procedure for the Desaturation Reaction– GP10

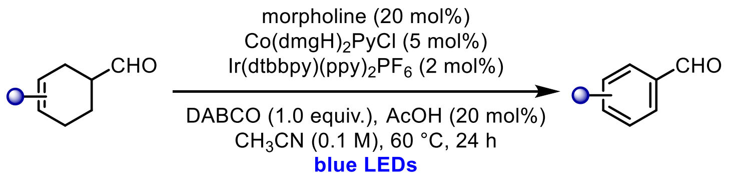

A dry tube equipped with a stirring bar was charged with Ir(dtbbpy)(ppy)$_2$PF$_6$ (4 mg, 4 µmol, 2 mol%), Co(dmgH)$_2$PyCl (4 mg, 0.01 mmol, 5 mol%), DABCO (22 mg, 0.20 mmol, 1.0 equiv.). and the aldehyde (if solid, 0.20 mmol, 1.0 equiv.). The tube was capped with a Supelco aluminium crimp seal with septum (PTFE/butyl), then evacuated under high vacuum and backfilled with N_2 (x 3). Degassed CH_3CN (2.0 mL, 0.1 M), the carbaldehyde (if liquid, 0.20 mmol, 1.0 equiv.) and morpholine (3.5 µL, 0.04 mmol, 20 mol%) and AcOH (2.5 µL, 0.04 mmol, 20 mol%) were sequentially added. The vial was purged with a stream of N_2, and the lid sealed with parafilm and placed approximately 4 cm from blue LEDs. The blue LEDs were switched on and the mixture was stirred under irradiation without fan for 24 hours. The tube was opened, and the mixture was quenched with H_2O (0.5 mL) then the solvent was evaporated under reduced pressure. The crude was purified by flash column chromatography on silica gel with hexane–EtOAc.

Benzaldehyde (2)

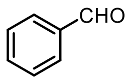

Following **GP10**, 3-cyclohexene-1-carboxaldehyde **1** (23 µL, 0.2 mmol) gave **2** (74%, NMR yield). ^{1}H NMR (500 MHz, $CDCl_3$) δ 10.01 (1H, s), 7.91–7.81 (2H, m), 7.66–7.58 (1H, m), 7.52-7.49 (2H, m); ^{13}C NMR (126 MHz, $CDCl_3$) δ 192.5, 136.4, 134.5, 129.8, 129.1. Data in accordance with the literature.[22]

• Following **GP10** but using **4** (24 µL, 0.20 mmol) instead of **1** and stirring the reaction for 30 h, morpholine (21 µL, 0.24 mmol, 1.2 equiv.) and DABCO (67 mg, 0.6 mmol, 3.0 equiv.) gave **2** (27%, NMR yield).

• Following **GP10** but using **5** (23 µL, 0.20 mmol) instead of **1** and stirring the reaction for 30 h, morpholine (21 µL, 0.24 mmol, 1.2 equiv.) gave **2** (42%, NMR yield).

• Following **GP10** but using **3** (36 mg, 0.2 mmol) instead of **1** and without morpholine and stirring the reaction at r.t., gave **2** (43%, NMR yield).

2,4,5-Trimethylbenzaldehyde (6)

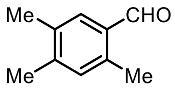

Following **GP10** but stirring the reaction for 30 h, *trans*-3,4,6-trimethyl-3-cyclohexene-1-carbaldehyde **6s** (31 mg, 0.2 mmol) gave **6** (27 mg, 90%) as an oil. ^{1}H NMR (400 MHz, $CDCl_3$) δ 10.18 (1H, s), 7.54 (1H, s), 7.02 (1H, s), 2.59 (3H, s), 2.29 (6H, s); ^{13}C NMR (101 MHz, $CDCl_3$) δ 192.7, 143.5, 138.2, 134.7, 133.3, 133.2, 132.2, 20.1, 19.2, 19.0. Data in accordance with the literature.[23]

2-Ethyl-4,5-dimethylbenzaldehyde (7)

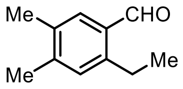

Following **GP10** but stirring the reaction for 30 h, *trans*-6-ethyl-3,4-dimethyl-3-cyclohexene-1-carbaldehyde **7s** (33 mg, 0.2 mmol) gave **7** (27 mg, 83%) as an oil. R_f 0.37 [petrol–EtOAc (8:1)]; ^{1}H NMR (400 MHz, $CDCl_3$) δ 10.20 (1H, s), 7.58 (1H, s), 7.05 (1H, s), 2.99 (2H, q, *J* = 7.5 Hz), 2.30 (3H, s), 2.29 (3H, s), 1.24 (3H, t, *J* = 7.5 Hz); ^{13}C NMR (101 MHz, $CDCl_3$) δ 192.2, 144.8, 143.8, 134.8, 132.8, 131.8, 131.5, 25.3, 20.2, 19.2, 16.7; HRMS (ASAP): Found MH^+ 163.1112, $C_{11}H_{15}O$ requires 163.1117.

4,5-Dimethyl-2-propylbenzaldehyde (8)

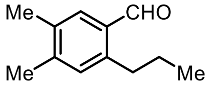

Following **GP10** but stirring the reaction for 30 h, *trans*-3,4-dimethyl-6-propyl-3-cyclohexene-1-carbaldehyde **8s** (36 mg, 0.2 mmol) gave **8** (29 mg, 83%) as an oil. R_f 0.37 [petrol–EtOAc (8:1)]; ^{1}H NMR (400 MHz, $CDCl_3$) δ 10.21 (1H, s), 7.59 (1H, s), 7.03 (1H, s), 2.93 (t, *J* = 7.8 Hz), 2.30 (3H, s), 2.28 (3H, s), 1.65–1.59 (2H, m), 0.97 (3H, t, *J* = 7.3 Hz); ^{13}C NMR (101 MHz, $CDCl_3$) δ 192.1, 143.6, 143.3, 134.9, 132.5, 132.4, 131.8, 34.0, 25.8, 20.2, 19.2, 14.1; HRMS (ASAP): Found MH^+ 177.1271, $C_{12}H_{17}O$ requires 177.1274.

(*Z*)-2-(Hex-3-en-1-yl)-4,5-dimethylbenzaldehyde (9)

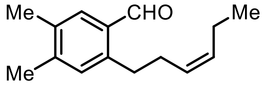

Following **GP10** but stirring the reaction for 30 h, *trans*-6-((*Z*)-hex-3-en-1-yl)-3,4-dimethyl-3-cyclohexene-1-carbaldehyde **9s** (44 mg, 0.2 mmol) gave **9** (33 mg, 77%) as an oil. R_f 0.37 [petrol–EtOAc (8:1)]; ^{1}H NMR (400 MHz, $CDCl_3$) δ 10.20 (1H, s), 7.58 (1H, s), 7.03 (1H, s),

5.45–5.32 (2H, m), 3.01 (2H, t, *J* = 7.5 Hz), 2.36–2.31 (2H, m), 2.30 (3H, s), 2.29 (3H, s), 2.02–1.89 (2H, m), 0.88 (3H, t, *J* = 7.5 Hz); ^{13}C NMR (101 MHz, $CDCl_3$) δ 192.2, 143.6, 142.6, 135.0, 132.9, 132.7, 132.6, 131.8, 127.6, 32.1, 29.8, 20.6, 20.2, 19.3, 14.3; HRMS (ASAP): Found MH^+ 217.1579, $C_{15}H_{21}O$ requires 217.1587.

4,5-Dimethyl-[1,1'-biphenyl]-2-carbaldehyde (10)

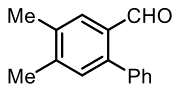

Following **GP10** but stirring the reaction for 30 h, *trans*-3,4,-dimethyl-6-phenyl-3-cyclohexene-1-carbaldehyde **10s** (43 mg, 0.2 mmol) gave **10** (29 mg, 70%) as an oil. ^{1}H NMR (400 MHz, $CDCl_3$) δ 9.94 (1H, s), 7.82 (1H, s), 7.49–7.41 (3H, m), 7.39–7.34 (2H, m), 7.22 (1H, s), 2.38 (3H, s), 2.37 (3H, s); ^{13}C NMR (101 MHz, $CDCl_3$) δ 192.5, 144.0, 143.6, 138.0, 136.7, 132.1, 131.7, 130.2, 128.5, 128.4, 127.9, 20.3, 19.5. Data in accordance with the literature.[24]

4'-Methoxy-4,5-dimethyl-[1,1'-biphenyl]-2-carbaldehyde (12)

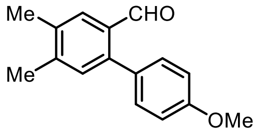

Following **GP10** but stirring the reaction for 30 h, *trans*-4'-methoxy-4,5-dimethyl-1,2,3,6-tetrahydro-[1,1'-biphenyl]-2-carbaldehyde **12s** (49 mg, 0.2 mmol) gave **12** (42 mg, 88%) as an oil. R_f 0.29 [petrol–EtOAc (8:1)]; ^{1}H NMR (400 MHz, $CDCl_3$) δ 9.94 (1H, s), 7.78 (1H, s), 7.28 (2H, d, *J* = 8.7 Hz), 7.20 (1H, s), 6.98 (2H, d, *J* = 8.7 Hz), 3.87 (3H, s), 2.36 (3H, s), 2.35 (3H, s); ^{13}C NMR (101 MHz, $CDCl_3$) δ 192.7, 159.6, 143.7, 143.5, 136.3, 132.1, 131.7, 131.4, 130.3, 128.5, 113.9, 55.5, 20.3, 19.5; HRMS (ESI): Found MNa^+ 263.1033, $C_{16}H_{16}O_2Na$ requires 263.1043.

2-(2-Furyl)-4,5-dimethylbenzaldehyde (13)

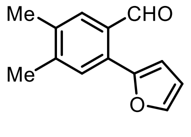

Following **GP10** but stirring the reaction for 30 h, *cis*-6-(2-furyl)-3,4-dimethyl-3-cyclohexene-1-carbaldehyde **13s** (41 mg, 0.2 mmol) gave **13** (32 mg, 73%) as an oil. R_f 0.37 [petrol–EtOAc (8:1)]; ^{1}H NMR (500 MHz, $CDCl_3$) δ 10.32 (1H, s), 7.76 (1H, s), 7.58 (1H, dd, *J* = 1.8, 0.8 Hz), 7.44 (1H, s), 6.57 (1H, dd, *J* = 3.3, 0.8 Hz), 6.54 (1H, dd, *J* = 3.3, 1.8 Hz),

2.35 (3H, s), 2.33 (3H, s); ^{13}C NMR (126 MHz, $CDCl_3$) δ 192.4, 151.4, 143.7, 143.6, 137.2, 131.5, 131.2, 129.7, 128.9, 111.9, 110.8, 20.3, 19.6; HRMS (ESI): Found MNa^+ 223.0724, $C_{13}H_{12}O_2Na$ requires 223.0730.

Ethyl 2-formyl-4,5-dimethylbenzoate (14)

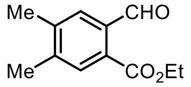

Following **GP10**, ethyl *trans*-6-formyl-3,4-dimethyl-3-cyclohexene-1-carboxylate **14s** (42 mg, 0.2 mmol) gave **14** (12 mg, 30%) as a solid. ^{1}H NMR (400 MHz, $CDCl_3$) δ 10.60 (1H, s), 7.73 (1H, s), 7.68 (1H, s), 4.42 (2H, q, *J* = 7.2 Hz), 2.36 (3H, s), 2.35 (3H, s), 1.41 (3H, t, *J* = 7.2 Hz); ^{13}C NMR (101 MHz, $CDCl_3$) δ 192.5, 166.7, 142.8, 141.8, 134.9, 131.8, 130.2, 129.5, 61.8, 20.1, 19.9, 14.5. Data in accordance with literature.[25]

2,4-Dimethylbenzaldehyde (15)

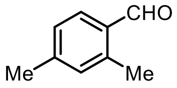

Following **GP10** but stirring the reaction for 30 h, commercially available substrate 2,4-dimethyl-3-cyclohexene-1-carboxaldehyde [CAS: 68039-49-6] (30 µL, 0.2 mmol) gave **15** (17 mg, 63%) as an oil. ^{1}H NMR (500 MHz, $CDCl_3$) δ 10.20 (1H, s), 7.69 (1H, d, *J* = 7.8 Hz), 7.16 (1H, d, *J* = 7.8 Hz), 7.07 (1H, s), 2.63 (3H, s), 2.38 (3H, s); ^{13}C NMR (126 MHz, $CDCl_3$) δ 192.5, 144.7, 140.8, 132.7, 132.5, 132.1, 127.2, 21.8, 19.7. Data in accordance with the literature.[26]

4-Adamantan-1-yl-2-methylbenzaldehyde (16)

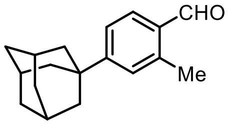

Following **GP10**, 4-adamantan-1-yl-*trans*-6-methyl-3cyclohex-ene-1-carbaldehyde **16s** (52 mg, 0.2 mmol) gave **16** (37 mg, 72%) as a solid. R_f 0.31 [EtOAc–Pentane (5:95)]; ^{1}H NMR (400 MHz, $CDCl_3$) δ 10.22 (1H, s), 7.75 (1H, d, *J* = 8.2 Hz), 7.36 (1H, d, *J* = 8.2 Hz), 7.24 (1H, s), 2.67 (3H, s), 2.19–2.06 (3H, m), 2.01–1.85 (6H, m), 1.85–1.73 (6H, m); ^{13}C NMR (101 MHz, $CDCl_3$) δ 192.7, 157.7, 140.5, 132.4, 132.1, 128.6, 123.1, 42.9, 36.9, 36.8, 28.9, 20.1; HRMS (ASAP): Found MH^+ 255.1736, $C_{18}H_{23}O$ requires 255.1749.

2-(4-Methoxyphenyl)-4-(4-fluorophenyl)benzaldehyde (17)

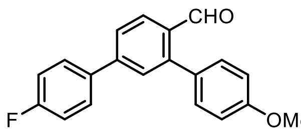

Following **GP10**, 4-(4-fluorophenyl)-*trans*-6-(4-methoxyphenyl)-3-cyclohexene-1-carbaldehyde **17s** (62 mg, 0.2 mmol) gave **17** (34 mg, 55%) as a solid. R_f 0.27 [EtOAc–Pentane (1:9)]; ^{1}H NMR (400 MHz, $CDCl_3$) δ 10.02 (1H, s), 8.08 (1H, d, *J* = 8.1 Hz), 7.69–7.57 (4H, m), 7.36 (2H, d, *J* = 8.4 Hz), 7.17 (2H, t, *J* = 8.6 Hz), 7.03 (2H, d, *J* = 8.4 Hz), 3.89 (3H, s); ^{13}C NMR (101 MHz, $CDCl_3$) δ 192.3, 163.3 (d, $J_{C\text{-}F}$ = 248.5 Hz), 160.0, 146.4, 145.3, 136.0 (d, $J_{C\text{-}F}$ = 3.3 Hz), 132.7, 131.4, 130.1, 129.4, 129.2 (d, $J_{C\text{-}F}$ = 8.1 Hz), 128.5, 126.1, 116.1 (d, $J_{C\text{-}F}$ = 21.6 Hz), 114.2, 55.6; ^{19}F NMR (376 MHz, $CDCl_3$) δ –113.61 (ddd, *J* = 14.0, 8.6, 5.3 Hz); HRMS (ASAP): Found MH^+ 307.1130, $C_{20}H_{16}FO_2$ requires 307.1134.

2-(4-Fluorophenyl)-4-(thiophen-3-yl)benzaldehyde (18)

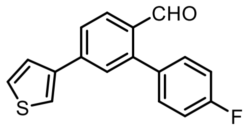

Following **GP10** on a 0.1 mmol scale, 4-(thiophen-3-yl)-*trans*-6-(4-fluorophenyl)-3-cyclohexene-1-carbaldehyde **18s** (29 mg, 0.1 mmol) gave **18** (8 mg, 30%) as a solid. R_f 0.30 [EtOAc–Pentane (5:95); ^{1}H NMR (500 MHz, $CDCl_3$) δ 9.95 (1H, s), 8.06 (1H, d, *J* = 8.2 Hz), 7.73 (1H, ddd, *J* = 8.2, 1.8, 0.8 Hz), 7.63 (1H, dd, *J* = 2.9, 1.5 Hz), 7.62 (1H, d, *J* = 1.4 Hz), 7.48–7.43 (2H, m), 7.40 (2H, dd, *J* = 8.7, 5.3 Hz), 7.19 (2H, t, *J* = 8.7 Hz); ^{13}C NMR (126 MHz, $CDCl_3$) δ 191.6, 163.0 (d, $J_{C\text{-}F}$ = 248.4 Hz), 145.7, 140.9 (2 x C), 134.0 (d, $J_{C\text{-}F}$ = 3.3 Hz), 132.5, 131.8 (d, $J_{C\text{-}F}$ = 8.3 Hz), 128.8, 128.7, 127.1, 126.3, 126.0, 122.8, 115.7 (d, $J_{C\text{-}F}$ = 21.6 Hz); ^{19}F NMR (376 MHz, $CDCl_3$) δ –113.57 (tt, *J* = 8.5, 5.2 Hz); HRMS (ESI): Found MH^+ 283.0579, $C_{17}H_{12}FOS$ requires 283.0593.

4-(6-Methoxypyridin-3-yl)benzaldehyde (19)

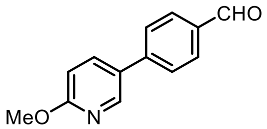

Following **GP10**, 4-(6-methoxypyridin-3-yl)-3-cyclohexene-1-carbaldehyde **19s** (44 mg, 0.2 mmol) gave **19** (32 mg, 76%) as a solid. R_f 0.25 [EtOAc–Pentane (1:4)]; ^{1}H NMR (400 MHz, $CDCl_3$) δ 10.05 (1H, s), 8.45 (1H, d, *J* = 2.6 Hz), 7.95 (2H, d, *J* = 8.4 Hz), 7.84 (1H, dd, *J* = 8.5, 2.6 Hz), 7.69 (2H, d, *J* = 8.4 Hz), 6.85 (1H, d, *J* = 8.5 Hz), 4.00 (3H, s); ^{13}C NMR (101

MHz, $CDCl_3$) δ 191.9, 164.5, 145.6, 144.1, 137.6, 135.3, 130.6, 128.8, 127.2, 111.3, 53.9; HRMS (ASAP): Found MH^+ 214.0865, $C_{13}H_{12}NO_2$ requires 214.0868.

4-(4-Hydroxy-4-methylpentyl)benzaldehyde and 3-(4-Hydroxy-4-methylpentyl)benzaldehyde (23)

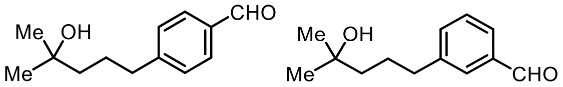

Following **GP10**, commercially available substrate 4-(4-hydroxy-4-methylpentyl)-3-cyclohexene-1-carboxaldehyde and 3-(4-hydroxy-4-methylpentyl)-3-cyclohexene-1-carbaldehyde [CAS: 31906-04-4, mixture of isomers] **22** (42 mg, 0.2 mmol) gave **23** (30 mg, 73%) as an oil. R_f 0.24 [petrol–EtOAc (2:1)]; ^{1}H NMR (400 MHz, $CDCl_3$) δ 9.99 (0.2H, s), 9.96 (0.8H, s), 7.79 (1.6H, d, *J* = 8.2 Hz), 7.72–7.70 (0.2H, m), 7.70–7.66 (0.2H, m), 7.48–7.41 (0.4H, m), 7.34 (1H, d, *J* = 8.2 Hz), 2.70 (2H, t, *J* = 7.7 Hz), 1.80–1.66 (2H, m), 1.55–1.46 (2H, m), 1.30 (1H, s), 1.20 (6H, s); ^{13}C NMR (101 MHz, $CDCl_3$) δ 192.8, 192.2, 150.1, 143.6, 136.7, 134.8, 134.6, 130.1, 129.4, 129.2, 129.1, 127.8, 71.0, 70.9, 43.4 (2 x C), 36.6, 36.1, 29.4 (2 x C), 26.2, 25.9; HRMS (ESI): Found MNa^+ 229.1196, $C_{13}H_{18}O_2Na$ requires 229.1199.

4-Hydroxy-2-methylbenzaldehyde (25)

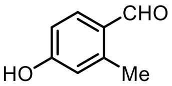

Following **GP10**, 4-((*tert*-butyldimethylsilyl)oxy)-2-methoxy-*trans*-6-methyl-3-cyclohexene-1-carbaldehyde **24** (57 mg, 0.2 mmol) gave **25** (13 mg, 46%) as a solid. ^{1}H NMR (400 MHz, $CDCl_3$) δ 10.10 (1H, s), 7.72 (1H, d, *J* = 8.4 Hz), 6.80 (1H, d, *J* = 9.3 Hz), 6.71 (1H, s), 6.13 (1H, br s) 2.63 (3H, s); ^{13}C NMR (126 MHz, $CDCl_3$) δ 191.4, 160.5, 144.0, 135.3, 128.1, 118.5, 113.5, 19.9. Data in accordance with literature.[27]

3-Phenylbenzaldehyde (26)

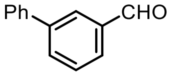

Following **GP10**, 1,2,3,4-tetrahydro-[1,1'-biphenyl]-3-carbaldehyde **26s** (37 mg, 0.2 mmol) gave **26** (23 mg, 62%) as a solid. ^{1}H NMR (500 MHz, $CDCl_3$) δ 10.10 (1H, s), 8.11 (1H, s), 7.87 (2H, dd, *J* = 7.7, 1.7 Hz), 7.65–7.60 (3H, m), 7.49 (2H, t, *J* = 7.7 Hz), 7.41 (1H, t, *J* = 7.4 Hz); ^{13}C NMR (126 MHz, $CDCl_3$) δ 192.5, 142.3, 139.8, 137.0, 133.2, 129.7, 129.2, 128.8, 128.4, 128.2, 127.3 Data in accordance with literature.[28]

3-Hydroxybenzaldehyde (27)

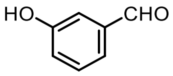

Following **GP10**, *cis*-5-((*tert*-butyldimethylsilyl)oxy)-3-cyclohexene-1-carbaldehyde **27s** (48 mg, 0.2 mmol) gave **27** (11 mg, 43%) as a solid.[a] ^{1}H NMR (500 MHz, $CDCl_3$) δ 9.96 (1H, s), 7.46 (1H, dt, *J* = 7.5, 1.4 Hz), 7.43 (1H, t, *J* = 7.6 Hz), 7.37 (1H, dd, *J* = 2.7, 1.4 Hz), 7.14 (1H, ddd, *J* = 7.6, 2.7, 1.5 Hz), 5.71 (1H, br s); ^{13}C NMR (126 MHz, $CDCl_3$) δ 192.5, 156.6, 138.0, 130.5, 123.6, 122.2, 114.9. Data in accordance with the literature.[29]

3-Methoxybenzaldehyde (28)

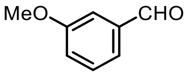

Following **GP10** but stirring the reaction for 30 h, *cis*-5-methoxy-3-cyclohexene-1-carbaldehyde **28s** (28 mg, 0.2 mmol) gave **28** (12 mg, 34%) as an oil. ^{1}H NMR (500 MHz, $CDCl_3$) δ 9.98 (1H, s), 7 7.48–7.43 (2H, m), 7.41–7.39 (1H, m), 7.18 (1H, dt, *J* = 6.6, 2.6 Hz), 3.87 (3H, s); ^{13}C NMR (126 MHz, $CDCl_3$) δ 192.3, 160.3, 137.9, 130.2, 123.7, 121.7, 112.2, 55.6. Data in accordance with the literature.[30]

3-(*iso*-Propoxy)benzaldehyde (29)

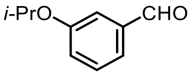

Following **GP10** but stirring the reaction for 30 h, 5-(*iso*-propoxy)-3-cyclohexene-1-carbaldehyde **29s** (34 mg, 0.2 mmol) gave **29** (10 mg, 30%) as an oil.[a] ^{1}H NMR (500 MHz, $CDCl_3$) δ 9.96 (1H, s), 7.47–7.40 (2H, m), 7.38 (1H, d, *J* = 2.6 Hz), 7.19–7.11 (1H, m), 4.63 (1H, hept, *J* = 6.0 Hz), 1.36 (6H, d, *J* = 6.0 Hz); ^{13}C NMR (126 MHz, $CDCl_3$) δ 192.4, 158.6, 137.9, 130.2, 123.3, 123.2, 114.2, 70.3, 22.0. Data in accordance with the literature.[31]

2-(((*tert*-Butyldimethylsilyl)oxy)methyl)benzaldehyde (30)

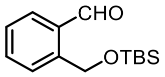

Following **GP10**, *cis*-6-(((*tert*-butyldimethylsilyl)oxy)methyl)-3-cyclohexene-1-carbaldehyde **30s** (51 mg, 0.2 mmol) gave **30** (35 mg, 70%) as an oil. ^{1}H NMR (400 MHz, $CDCl_3$) δ 10.17 (1H, s), 7.82 (1H, dd, *J* = 7.6, 1.4 Hz), 7.78 (1H, dd, *J* = 7.9, 1.2 Hz), 7.62 (1H, td, *J* = 7.6, 1.4 Hz), 7.49–7.40 (1H, td, *J* = 7.5, 1.2 Hz), 5.15 (2H, s), 0.96 (9H, s), 0.13 (6H, s); ^{13}C NMR

[a] In these examples we have observed isomerisation of the olefin to give the corresponding enals.

(101 MHz, $CDCl_3$) δ 193.5, 144.4, 134.1, 133.5, 132.7, 127.1, 126.8, 63.0, 26.1, 18.5, –5.2. Data in accordance with the literature.[32]

• Following **GP10** but stirring the reaction for 30 h, **30s** (1.02 g, 4.0 mmol) gave **30** (0.59 g) in 59% yield.

2-((Benzyloxy)methyl)benzaldehyde (31)

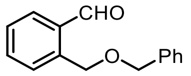

Following **GP10**, *cis*-6-((benzyloxy)methyl)-3-cyclohexene-1-carbaldehyde **31s** (46 mg, 0.2 mmol) gave **31** (32 mg, 71%) as an oil. R_f 0.50 [petrol–EtOAc (8:1)]; ^{1}H NMR (500 MHz, $CDCl_3$) δ 10.22 (1H, s), 7.87 (1H, dd, J = 7.6, 1.4 Hz), 7.70 (1H, dd, J = 7.7, 1.2 Hz), 7.61 (1H, td, J = 7.5, 1.4 Hz), 7.48 (1H, td, J = 7.5, 1.2 Hz), 7.41–7.35 (4H, m), 7.33–7.28 (1H, m), 4.99 (2H, s), 4.67 (2H, s); ^{13}C NMR (126 MHz, $CDCl_3$) δ 192.9, 141.0, 138.1, 134.0, 133.6, 132.6, 128.6, 128.4, 127.9 (3 x C), 73.0, 69.7; HRMS (ESI): Found MNa^+ 249.0884, $C_{15}H_{14}O_2Na$ requires 249.0886.

2-Formylbenzyl acetate (32)

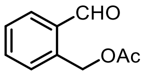

Following **GP10**, *cis*-(6-formylcyclohex-3-en-1-yl)methyl acetate **32s** (36 mg, 0.2 mmol) gave **32** (27 mg, 75%) as an oil. ^{1}H NMR (500 MHz, $CDCl_3$) δ 10.19 (1H, s), 7.88 (1H, dd, J = 7.9, 1.4 Hz), 7.61 (1H, td, J = 7.5, 1.5 Hz), 7.56–7.49 (2H, m), 5.55 (2H, s), 2.14 (3H, s); ^{13}C NMR (126 MHz, $CDCl_3$) δ 192.5, 170.7, 138.0, 134.1, 133.7, 133.4, 128.7, 128.6, 63.6, 21.0. Data in accordance with the literature.[33]

2-(((5-(Trifluoromethyl)pyridin-2-yl)oxy)methyl)benzaldehyde (33)

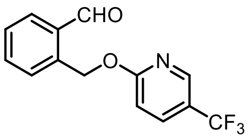

Following **GP10**, *cis*-6-(((5-(trifluoromethyl)pyridin-2-yl)oxy)methyl)-3-cyclohexene-1-carbaldehyde **33s** (57 mg, 0.2 mmol) gave **33** (39 mg, 70%) as an oil. R_f 0.35 [petrol–EtOAc (8:1)]; ^{1}H NMR (500 MHz, $CDCl_3$) δ 10.26 (1H, s), 8.44 (1H, d, J = 2.6 Hz), 7.91 (1H, dd, J = 7.6, 1.4 Hz), 7.81 (1H, dd, J = 8.7, 2.6 Hz), 7.64 (1H, dd, J = 7.7, 1.6 Hz), 7.61 (1H, td, J = 7.4, 1.4 Hz), 7.53 (1H, td, J = 7.4, 1.6 Hz), 6.92 (1H, d, J = 8.7 Hz), 5.88 (2H, s); ^{13}C NMR (126 MHz, $CDCl_3$) δ 192.8, 165.4, 145.2 (q, J_{C-F} = 4.4 Hz), 138.9, 136.1 (q, J_{C-F} = 3.1 Hz),

134.1, 133.7, 133.2, 128.7, 128.4, 124.1 (q, $J_{C\text{-}F}$ = 271.3 Hz), 120.6 (q, $J_{C\text{-}F}$ = 33.0 Hz), 111.4, 65.8; ^{19}F NMR (376 MHz, $CDCl_3$) δ –61.5; HRMS (ASAP): Found MH^+ 282.0735, $C_{14}H_{11}O_2NF_3$ requires 282.0736.

2-((1,3-Dioxoisoindolin-2-yl)methyl)benzaldehyde (34)

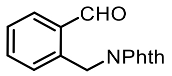

Following **GP10**, *cis*-6-((1,3-dioxoisoindolin-2-yl)methyl)-3-cyclohexene-1-carbaldehyde **34s** (54 mg, 0.2 mmol) gave **34** (37 mg, 69%) as an oil. R_f 0.32 [petrol–EtOAc (4:1)]; ^{1}H NMR (500 MHz, $CDCl_3$) δ 10.34 (1H, s), 7.89 (2H, dd, *J* = 5.4, 3.0 Hz), 7.87 (1H, dd, *J* = 7.3, 1.7 Hz), 7.75 (2H, dd, *J* = 5.4, 3.0 Hz), 7.51 (1H, td, *J* = 7.5, 1.7 Hz), 7.47 (1H, td, *J* = 7.4, 1.4 Hz), 7.26 (1H, dd, *J* = 7.5, 1.4 Hz), 5.36 (2H, s); ^{13}C NMR (126 MHz, $CDCl_3$) δ 193.1, 168.2, 138.0, 134.4, 134.1, 134.0, 133.6, 132.1, 128.1, 127.6, 123.7, 38.9; HRMS (ASAP): Found MH^+ 266.0821, $C_{16}H_{12}O_3N$ requires 266.0812. Data in accordance with the literature.[34]

***tert*-Butyl (2-formylbenzyl)(tosyl)carbamate (35)**

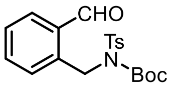

Following **GP10**, *cis*-*tert*-butyl ((6-formylcyclohex-3-en-1-yl)methyl)(tosyl)carbamate **35s** (79 mg, 0.2 mmol) gave **35** (55 mg, 70%) as an oil. R_f 0.32 [petrol–EtOAc (4:1)]; ^{1}H NMR (400 MHz, $CDCl_3$) δ 10.16 (1H, s), 7.87–7.79 (3H, m), 7.68–7.57 (2H, m), 7.49 (1H, ddd, *J* = 7.6, 6.5, 2.2 Hz), 7.32 (2H, d, *J* = 8.1 Hz), 5.53 (2H, s), 2.45 (3H, s), 1.29 (9H, s); ^{13}C NMR (101 MHz, $CDCl_3$) δ 193.8, 151.1, 144.6, 140.2, 137.0, 135.1, 134.3, 133.0, 129.4, 128.2, 127.5, 126.8, 84.7, 49.0, 27.9, 21.8; HRMS (ESI): Found MNa^+ 412.1187, $C_{20}H_{23}O_5NSNa$ requires 412.1189.

2-((4-(4,4,5,5-Tetramethyl-1,3,2-dioxaborolan-2-yl)-1*H*-pyrazol-1-yl)methyl)benzaldehyde (36)

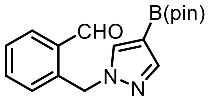

Following **GP10**, *cis*-6-((4-(4,4,5,5-tetramethyl-1,3,2-dioxaborolan-2-yl)-1*H*-pyrazol-1-yl)methyl)-3-cyclohexene-1-carbaldehyde **36s** (63 mg, 0.2 mmol) gave **36** (34 mg, 54%) as an oil. R_f 0.30 [petrol–EtOAc (2:1)]; ^{1}H NMR (400 MHz, $CDCl_3$) δ 10.15 (1H, s), 7.84 (1H,

s), 7.84–7.81 (1H, m), 7.78 (1H, s), 7.57–7.46 (2H, m), 6.97–6.89 (1H, m), 5.81 (2H, s), 1.31 (12H, s); ^{13}C NMR (101 MHz, $CDCl_3$) δ 193.4, 146.2, 138.5, 137.4, 135.0, 134.4, 133.2, 129.3, 128.5, 83.5, 53.0, 24.9; ^{11}B NMR (128 MHz, $CDCl_3$) δ 29.3; HRMS (ESI): Found MNa^+ 335.1529, $C_{17}H_{21}O_3N_2BNa$ requires 335.1537.

2-((Benzo[*d*]thiazol-2-ylthio)methyl)benzaldehyde (37)

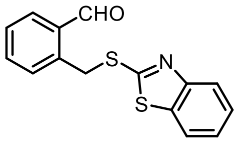

Following **GP10**, *cis*-6-((benzo[*d*]thiazol-2-ylthio)methyl)-3-cyclohexene-1-carbaldehyde **37s** (58 mg, 0.2 mmol) gave **37** (31 mg, 73%) as an oil. R_f 0.32 [petrol–EtOAc (8:1)]; ^{1}H NMR (500 MHz, $CDCl_3$) δ 10.26 (1H, s), 7.90 (1H, dd, *J* = 8.3, 1.2 Hz), 7.84 (1H, dd, *J* = 7.5, 1.7 Hz), 7.73 (1H, dd, *J* = 8.3, 1.2 Hz), 7.70 (1H, dd, *J* = 7.6, 1.4 Hz), 7.53 (1H, td, *J* = 7.5, 1.7 Hz), 7.48 (1H, td, *J* = 7.4, 1.4 Hz), 7.42 (1H, ddd, *J* = 8.3, 7.2, 1.2 Hz), 7.29 (1H, ddd, *J* = 8.3, 7.2, 1.2 Hz), 5.02 (2H, s); ^{13}C NMR (126 MHz, $CDCl_3$) δ 193.0, 166.5, 153.2, 139.0, 135.6, 134.5, 133.9, 133.7, 131.9, 128.5, 126.2, 124.4, 121.7, 121.2, 34.5; HRMS (ASAP): Found MH^+ 286.0349, $C_{15}H_{12}ONS_2$ requires 286.0355.

(1*R*,3*R*)-2,2-Dimethyl-1,2,3,4-tetrahydro-1,3-methanonaphthalene-5-carbaldehyde (40)

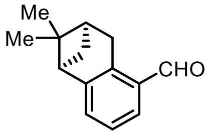

Following **GP10**, (1*R*,3*S*)-2,2-dimethyl-1,2,3,4,4a,5,6,7-octahydro-1,3-methanonaphthalene-5-carbaldehyde **39** (41 mg, 0.2 mmol) gave **40** (13 mg, 32%) as an oil. R_f 0.44 [petrol–EtOAc (8:1)]; ^{1}H NMR (500 MHz, $CDCl_3$) δ 10.23 (1H, s), 7.62 (1H, dd, *J* = 7.7, 1.4 Hz), 7.24 (1H, t, *J* = 7.5 Hz), 7.14 (1H, dd, *J* = 7.4, 1.4 Hz), 3.41 (1H, dd, *J* = 18.3, 3.2 Hz), 3.33 (1H, dd, *J* = 18.3, 2.8 Hz), 2.82 (1H, t, *J* = 5.6 Hz), 2.66–2.62 (1H, m), 2.37 (1H, tt, *J* = 6.0, 3.0 Hz), 2.17–2.08 (1H, m), 1.39 (3H, s), 0.62 (3H, s); ^{13}C NMR (126 MHz, $CDCl_3$) δ 193.3, 148.6, 138.0, 133.6, 131.6, 130.0, 125.9, 48.1, 40.3, 38.6, 32.3, 31.5, 26.1, 21.4; HRMS (ESI): Found MNa^+ 223.1088, $C_{14}H_{16}ONa$ requires 223.1093.

4-(2-(6-Methoxynaphthalen-2-yl)ethyl)-2-methylbenzaldehyde (43)

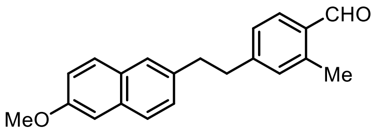

Following **GP10**, 4-(2-(6-methoxynaphthalen-2-yl)ethyl)-*trans*-6-methyl-3-cyclohexene-1-carbaldehyde **42** (0.2 mmol, 1 equiv.) gave **43** (31 mg, 51%) as a solid. R_f 0.34 [EtOAc–Pentane (1:9)]; ^{1}H NMR (400 MHz, $CDCl_3$) δ 10.22 (1H, s), 7.71 (1H, d, *J* = 7.8 Hz), 7.68 (1H, d, *J* = 4.8 Hz), 7.65 (1H, d, *J* = 5.5 Hz), 7.53 (1H, s), 7.30–7.24 (1H, m), 7.17 (1H, d, *J* = 7.8 Hz), 7.15–7.07 (3H, m), 3.92 (3H, s), 3.11–2.97 (4H, m), 2.64 (3H, s); ^{13}C NMR (126 MHz, $CDCl_3$) δ 192.6, 157.4, 148.4, 140.9, 136.4, 133.3, 132.6, 132.5, 132.2, 129.2, 129.1, 127.8, 127.0, 126.7, 126.6, 119.0, 105.8, 55.5, 38.1, 37.5, 19.8. HRMS (ASAP): Found MNa^+ 327.1349, $C_{21}H_{20}O_2Na$ requires 327.1361.

4-((3*S*,5*S*,8*R*,9*S*,10*S*,13*S*,14*S*,17*S*)-3-((*tert*-Butyldimethylsilyl)oxy)-10,13-dimethylhexadecahydro-1*H*-cyclopenta[*a*]phenanthren-17-yl)benzaldehyde (46)

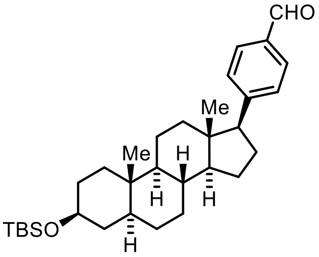

Following **GP10**, 4-((3*S*,5*S*,8*R*,9*S*,10*S*,13*S*,14*S*,17*R*)-3-((*tert*-butyldimethylsilyl)oxy)-10,13-dimethylhexadecahydro-1*H*-cyclopenta[*a*]phenanthren-17-yl)-3-cyclohexene-1-carbaldehyde **45** (100 mg, 0.2 mmol) gave **46** (64 mg, 65%) as a solid. R_f 0.41 [EtOAc–Pentane (5:95)]; ^{1}H NMR (400 MHz, $CDCl_3$) δ 9.98 (1H, s), 7.79 (2H, d, *J* = 8.0 Hz), 7.36 (2H, d, *J* = 8.0 Hz), 3.55 (1H, tt, *J* = 10.5, 4.8 Hz), 2.74 (1H, t, *J* = 9.7 Hz), 2.12 (1H, dtd, *J* = 14.0, 10.9, 3.3 Hz), 2.03–1.89 (1H, m), 1.81 (1H, qd, *J* = 10.4, 3.3 Hz), 1.76–1.62 (3H, m), 1.56–1.50 (2H, m), 1.49–1.16 (10H, m), 1.14–1.03 (1H, m), 1.00–0.90 (2H, m), 0.88 (9H, s), 0.79 (3H, s), 0.74–0.64 (1H, m), 0.45 (3H, s), 0.05 (6H, s); ^{13}C NMR (101 MHz, $CDCl_3$) δ 192.3, 149.3, 134.8, 129.4, 129.4, 72.3, 57.6, 56.6, 54.8, 45.3, 45.2, 38.8, 38.0, 37.4, 36.1, 35.8, 32.4, 32.1, 28.9, 26.2, 26.1, 24.7, 21.0, 18.4, 13.0, 12.6, –4.4; HRMS (ESI): Found MNa^+ 517.3465, $C_{32}H_{50}O_2SiNa$ requires 517.3478.

Cinnamaldehyde (50)

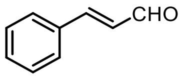

Following **GP10**, (*E*)-3-(cyclohex-3-en-1-yl)acrylaldehyde **49** (27 mg, 0.2 mmol) gave **50** (6 mg, 22%) as an oil. ^{1}H NMR (400 MHz, $CDCl_3$) δ 9.71 (1H, d, *J* = 7.7 Hz), 7.60–7.54 (2H, m), 7.48 (1H, d, *J* = 15.9 Hz), 7.46–7.38 (3H, m), 6.72 (1H, dd, *J* = 15.9, 7.7 Hz); ^{13}C NMR (101 MHz, $CDCl_3$) δ 193.9, 152.9, 134.1, 131.4, 129.2, 128.7, 128.6. Data in accordance with the literature.[35]

7 Mechanistic Considerations

7.1 Cyclic Voltammetry and Emission Quenching Studies

Cyclic Voltammetry General Experimental Details

Cyclic voltammetry was conducted on an EmStat (PalmSens) potentiostat using a 3-electrode cell configuration. A glassy carbon working electrode was employed alongside a platinum wire counter electrode and an Ag/AgCl reference electrode. All the solutions were degassed by bubbling N_2 prior to measurements. 5 mM solutions of the desired compounds were freshly prepared in dry acetonitrile along with 0.1 M of tetrabutylammonium hexafluorophosphate as supporting electrolyte and were examined at a scan rate of 0.1 V s^{-1}. Ferrocene ($E_{1/2}$ = +0.42 V vs SCE)[36] was added at the end of the measurements as an internal standard to determine the precise potential scale. Potential values are given versus the saturated calomel electrode (SCE). Irreversible waves were obtained in all cases; therefore, the potentials were estimated at half the maximum current, as previously described by Nicewicz.[37] Oxidation potentials higher than +1.5 V vs SCE was not determined.

Emission Quenching Studies General Experimental Details

Stern-Volmer experiments were carried out monitoring the emission intensity of argon-degassed solutions of $[Ir(ppy)_2(dtbbpy)]PF_6$ (2 x 10^{-5} M) containing variable amounts of the quencher in dry acetonitrile. The reported excited-state lifetime for $[Ir(ppy)_2(dtbbpy)]PF_6$ in MeCN (0.56 μs) was used for k_q calculations.[38]

Table S2.

$Ir(dtbbpy)(ppy)_2]PF_6$ redox properties			
E_{red} (V vs SCE)	*E_{ox}* (V vs SCE)	E_{red} (V vs SCE)	E_{ox} (V vs SCE)
+0.66	–0.96	–1.51	+1.21

Table S3.

	E_{ox} (V vs SCE)	E_{red} (V vs SCE)	k_{SV} (M^{-1} s^{-1})	k_q (M^{-1} s^{-1})
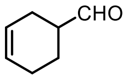	+2.10	-2.57	190	3.39 x 10^8
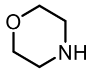	+1.10		141	2.52 x 10^8
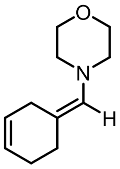	+0.55		1656	2.96 x 10^9
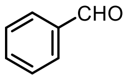	+2.55	-1.78	204	3.64 x 10^8
DABCO	+0.69		2560	4.57 x 10^9
$Co(dmgH)_2PyCl$	+0.85	-0.32, -1.02	5305	9.47 x 10^9

According to the cyclic voltammetry studies (Table S2 and S3 and Figure S3):

- *Ir(III) can only oxidize the enamine **A** and DABCO
- *Ir(III) can only reduce Co(III)
- Ir(II) can reduce Co(III) and Co(II)
- Ir(IV) can oxidized the enamine, DABCO, morpholine

According to the emission quenching experiments (Table S2 and S3 and Figure S4):

- *Ir(III) is efficiently quenched by the enamine **A**, DABCO and cobaloxime

DABCO can be replaced by other bases, such as NaOAc and lutidine, which do not quench the excited state of the photocatalyst. Therefore, we do not expect it to have a significant role in the redox events.

Considering the larger *k*SV of Co(dmgH)$_2$PyCl vs enamine **A**, an oxidative quenching is the most likely scenario between a visible-light excited [Ir(III)] photocatalyst and a [Co(III)] cobaloxime.

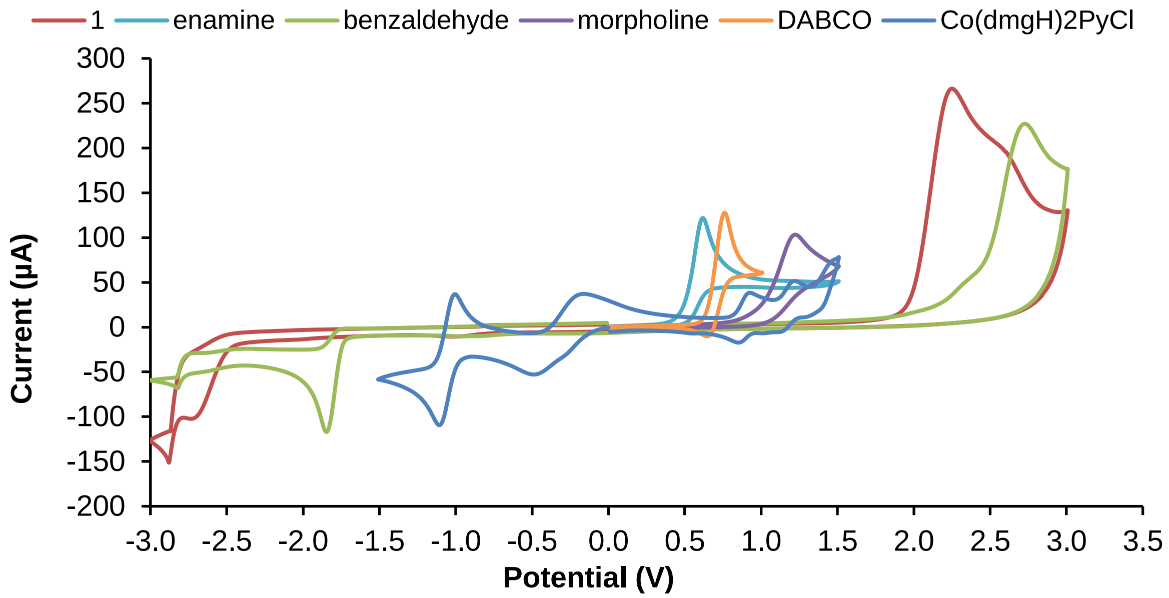

Figure S3.

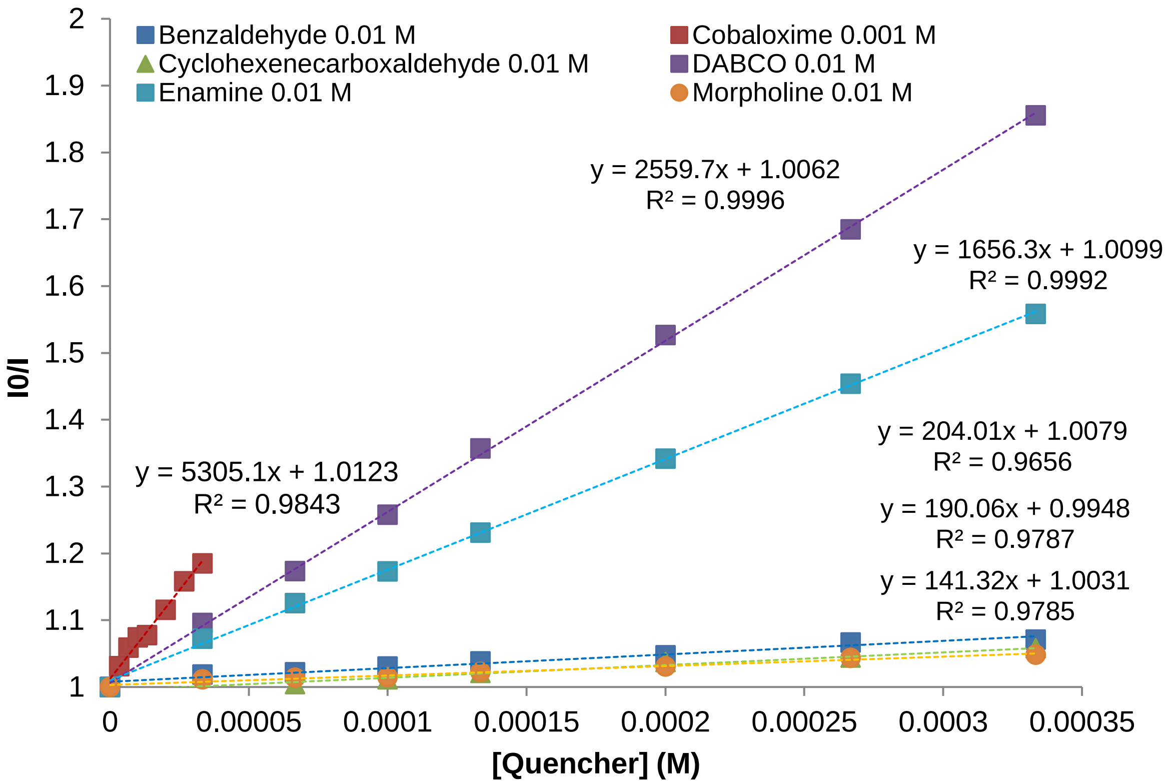

Figure S4.

7.2 Reaction on Preformed Enamine

To demonstrate the enamine was a competent starting material in the process, we have submitted the preformed enamine to the reaction conditions as detailed in Scheme S1.

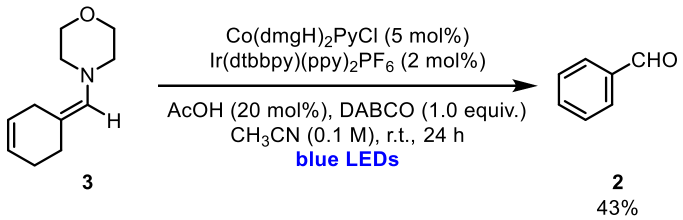

Scheme S1.

7.3 Detection of H_2

The reaction depicted in Scheme S2 and Scheme S3 were run in an J. Young tube NMR and immediately analysed by ^{1}H NMR spectroscopy which revealed the formation of H_2 (Figure S5 and Figure S6).

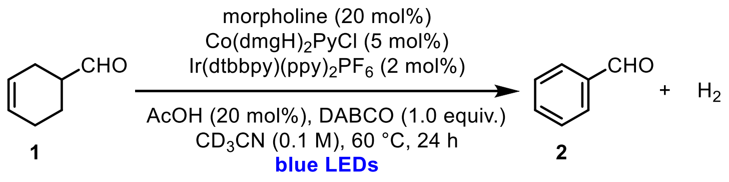

Scheme S2.

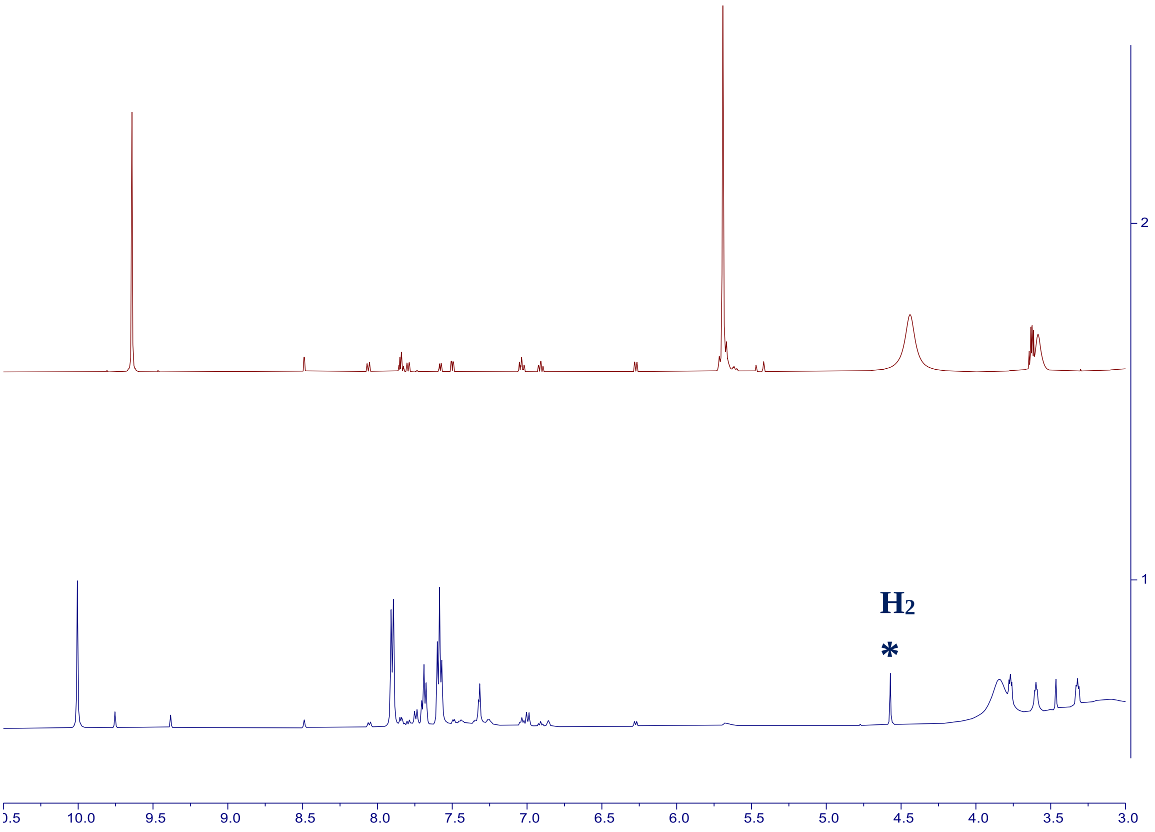

Figure S5.

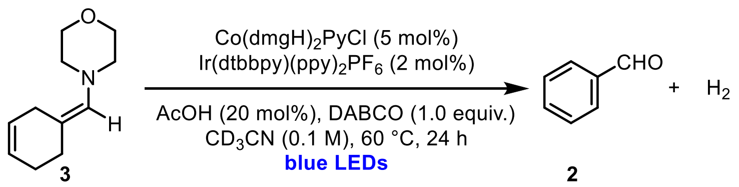

Scheme S3.

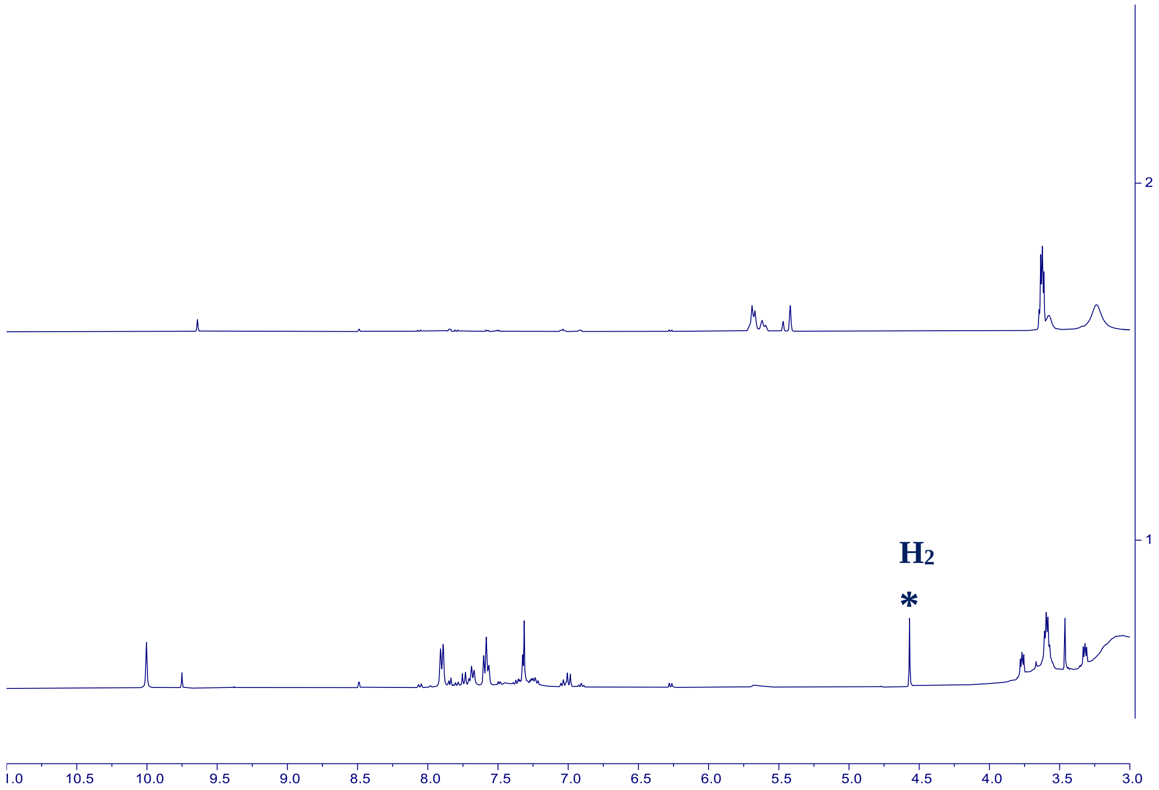

Figure S6.

7.4 Quantum Yields (Φ) Determination

The quantum yield of the photochemical reaction depicted in Scheme S4 was determined at 298 K following procedures described in literature.[39] Degassed reaction tubes were irradiated using as the light source blue LEDs plates (λ_{max} = 444 nm) for 30-60 min. The yield of products was determined by ^{1}H NMR spectroscopy; in all cases the conversion was lower than 20%. The photon flux of the blue LEDs used was determined by standard ferrioxalate actinometry.

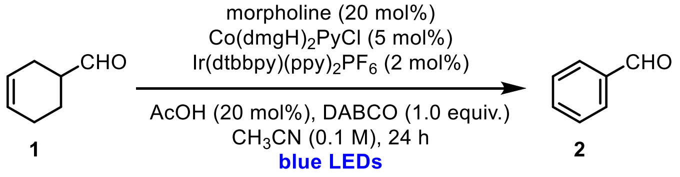

Φ = 0.0015

Scheme S4.

These results demonstrate that efficient radical chain propagations are not operating under these reaction conditions.

8 NMR Spectra

7s – ^{1}H NMR (400 MHz, $CDCl_3$)

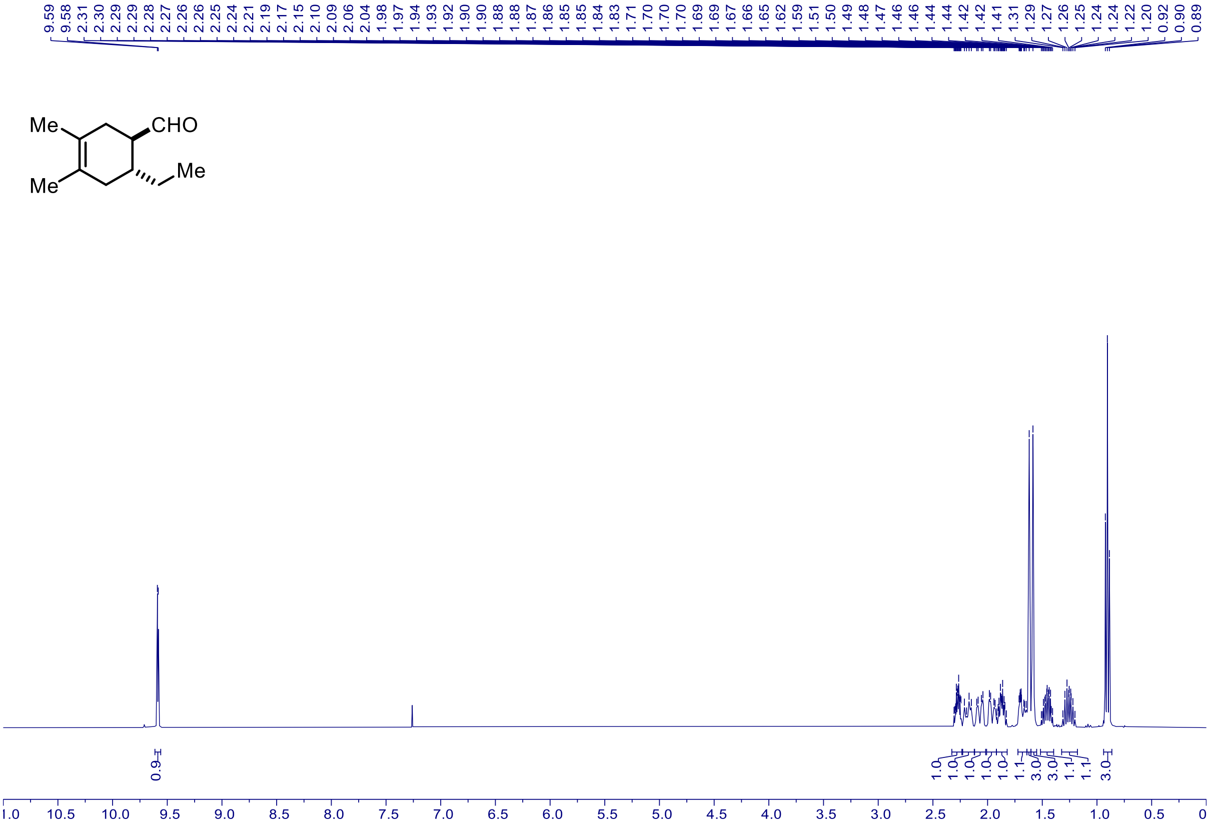

7s – ^{13}C NMR (101 MHz, $CDCl_3$)

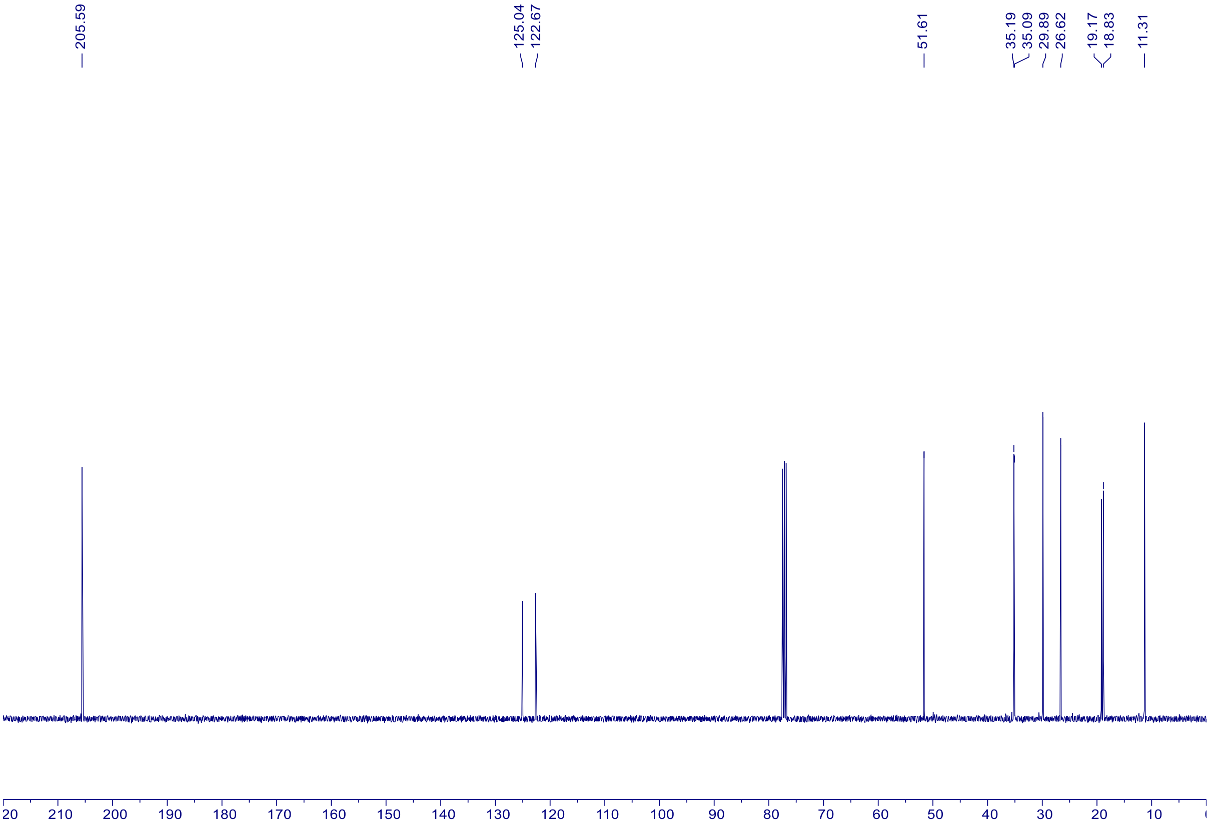

8s – ^{1}H NMR (400 MHz, CDCl$_3$)

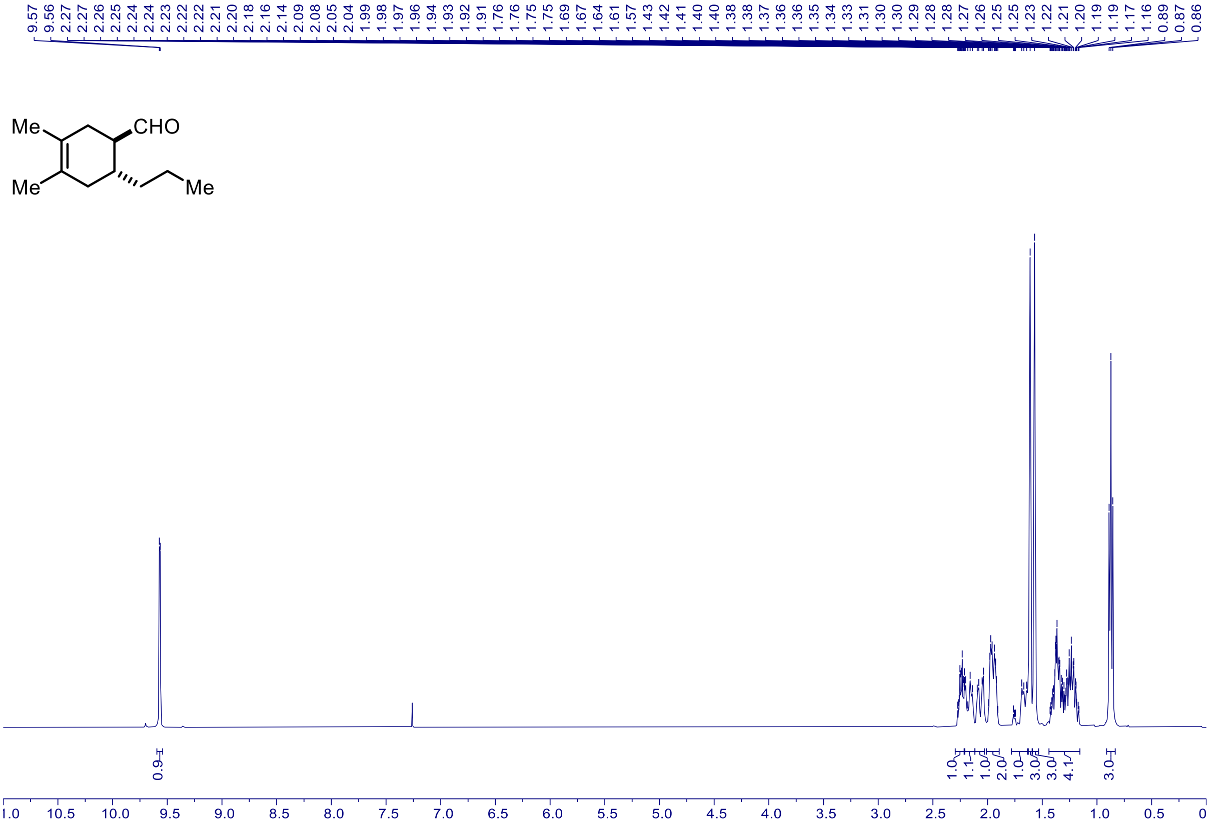

8s – ^{13}C NMR (101 MHz, CDCl$_3$)

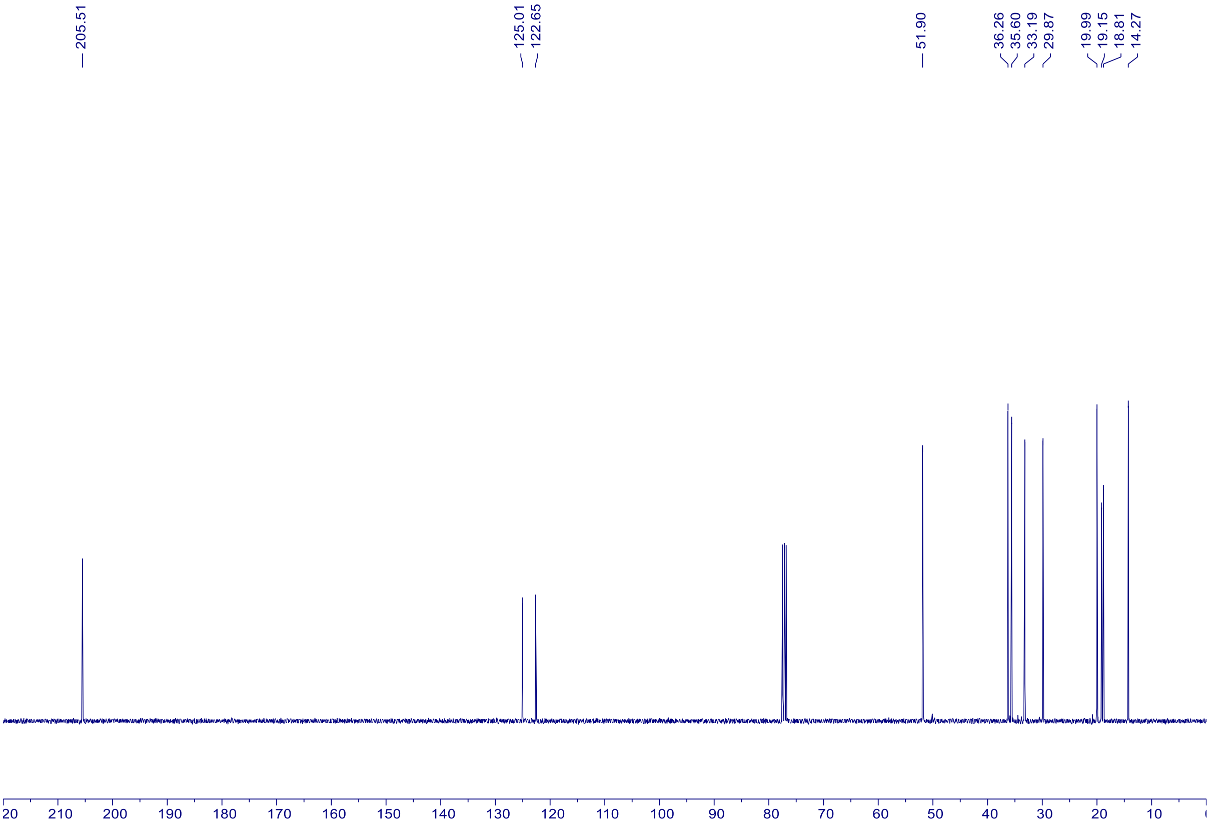

9s – ^{1}H NMR (400 MHz, $CDCl_3$)

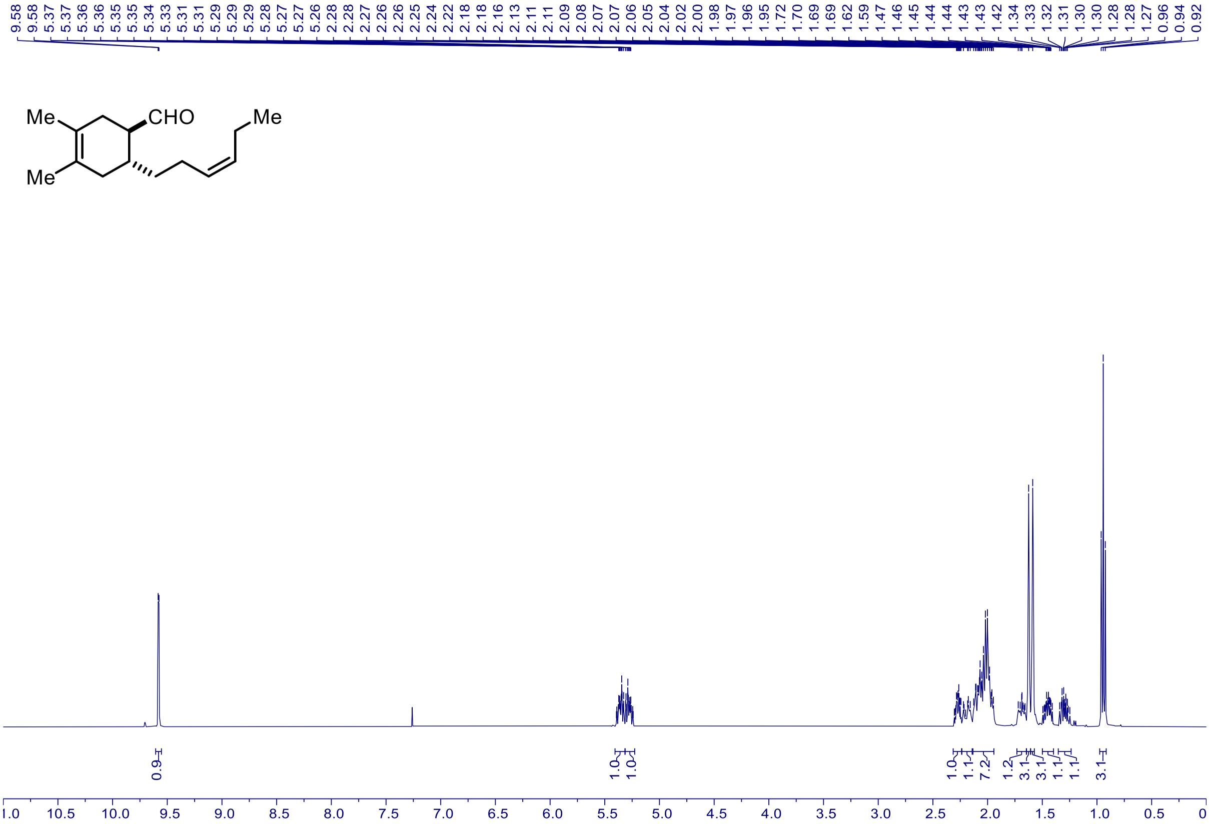

9s – ^{13}C NMR (101 MHz, $CDCl_3$)

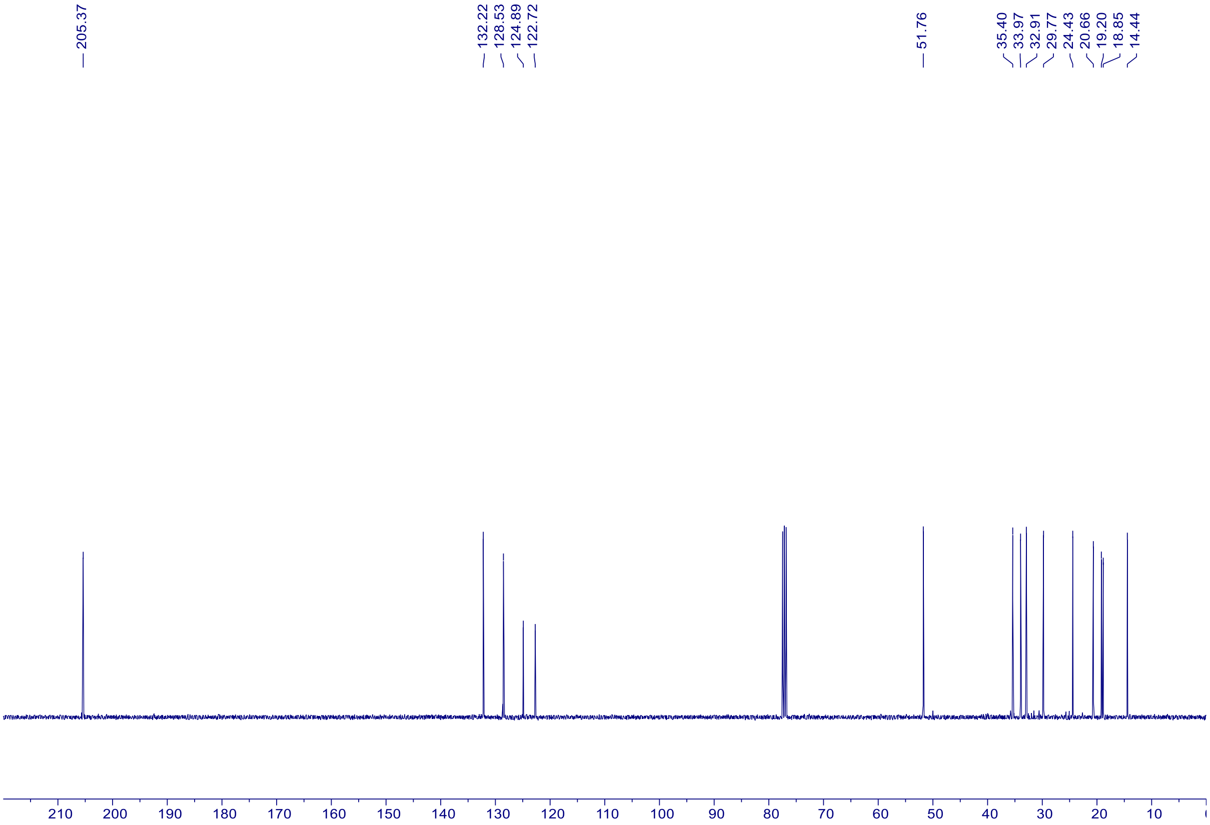

12s – ^{1}H NMR (400 MHz, $CDCl_3$)

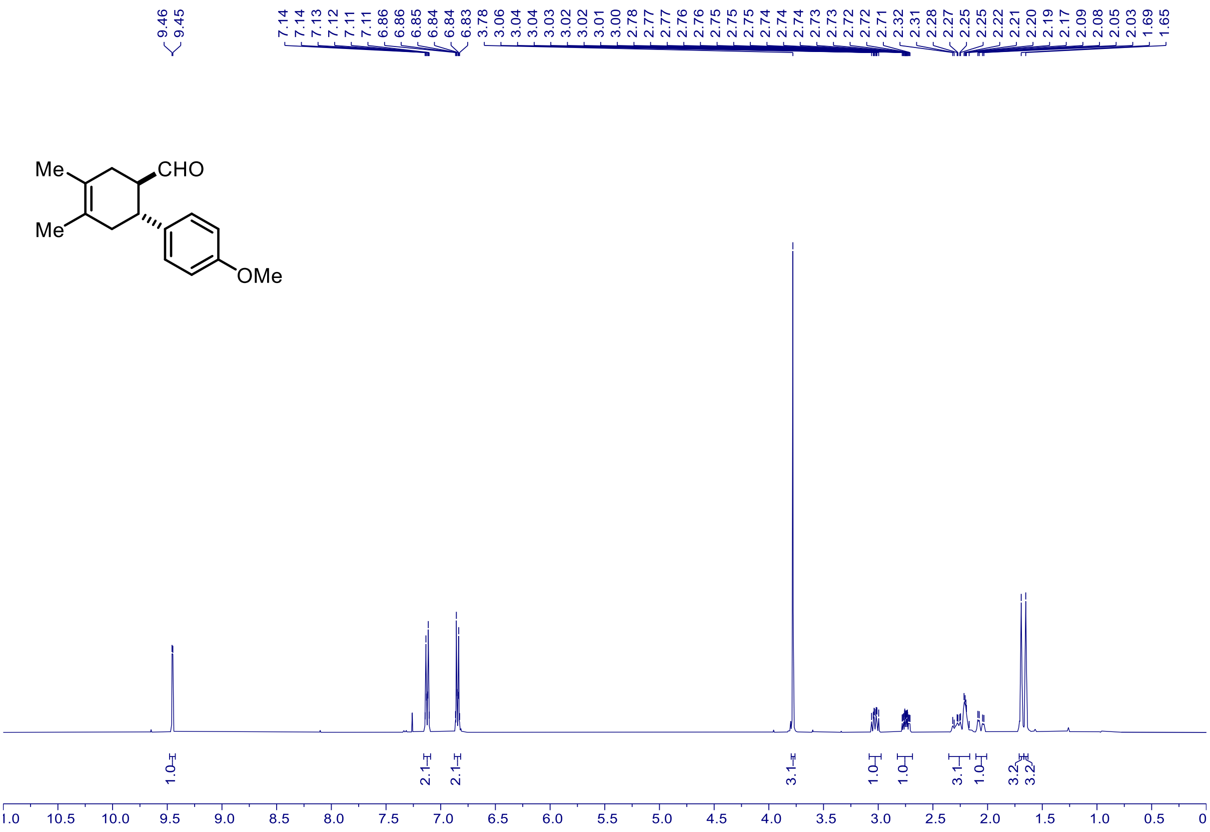

12s – ^{13}C NMR (101 MHz, $CDCl_3$)

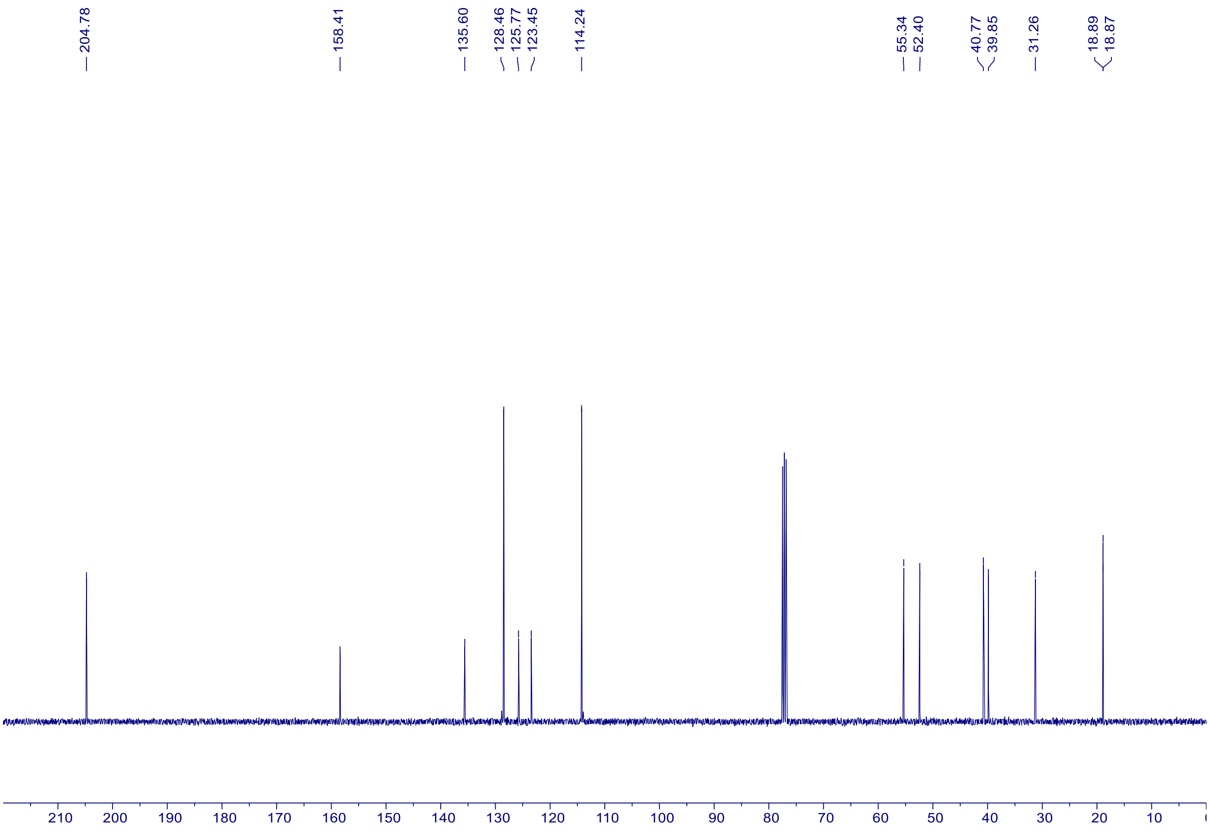

13s – ^{1}H NMR (400 MHz, CDCl$_3$)

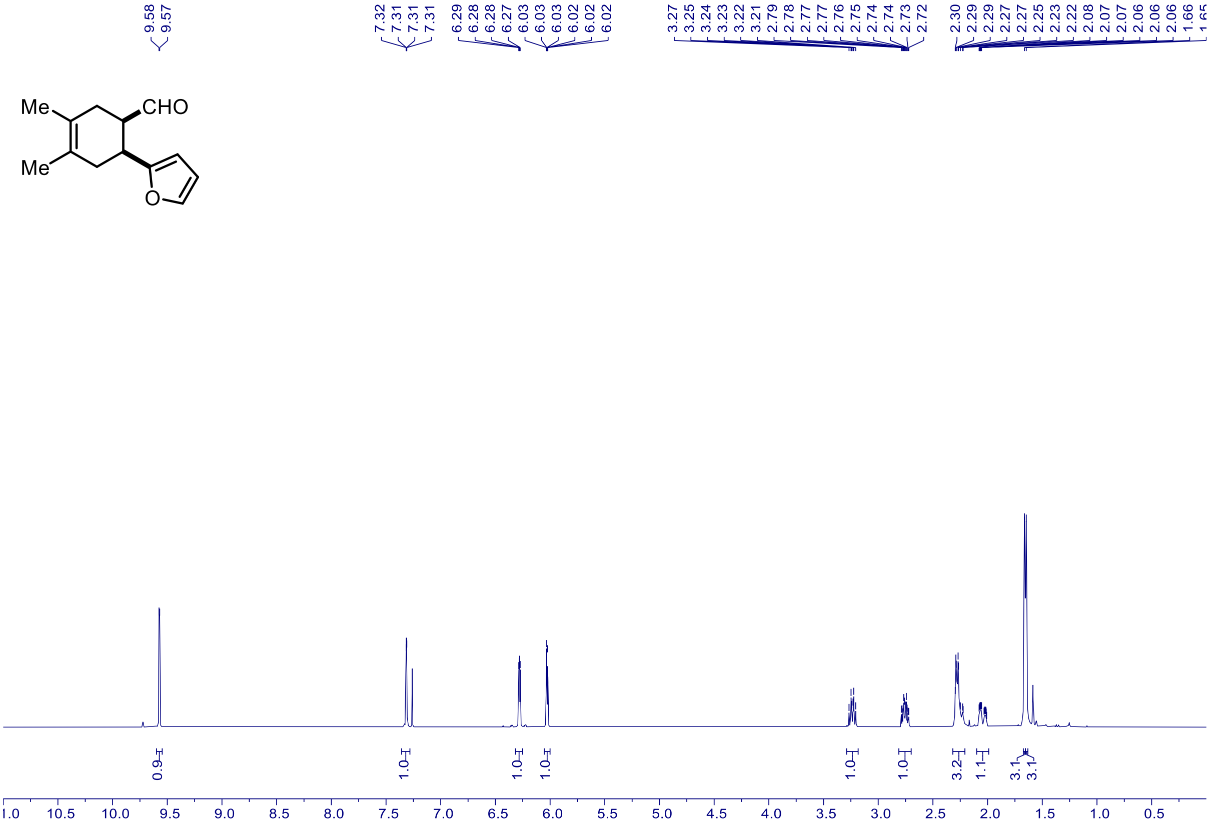

13s – ^{13}C NMR (101 MHz, CDCl$_3$)

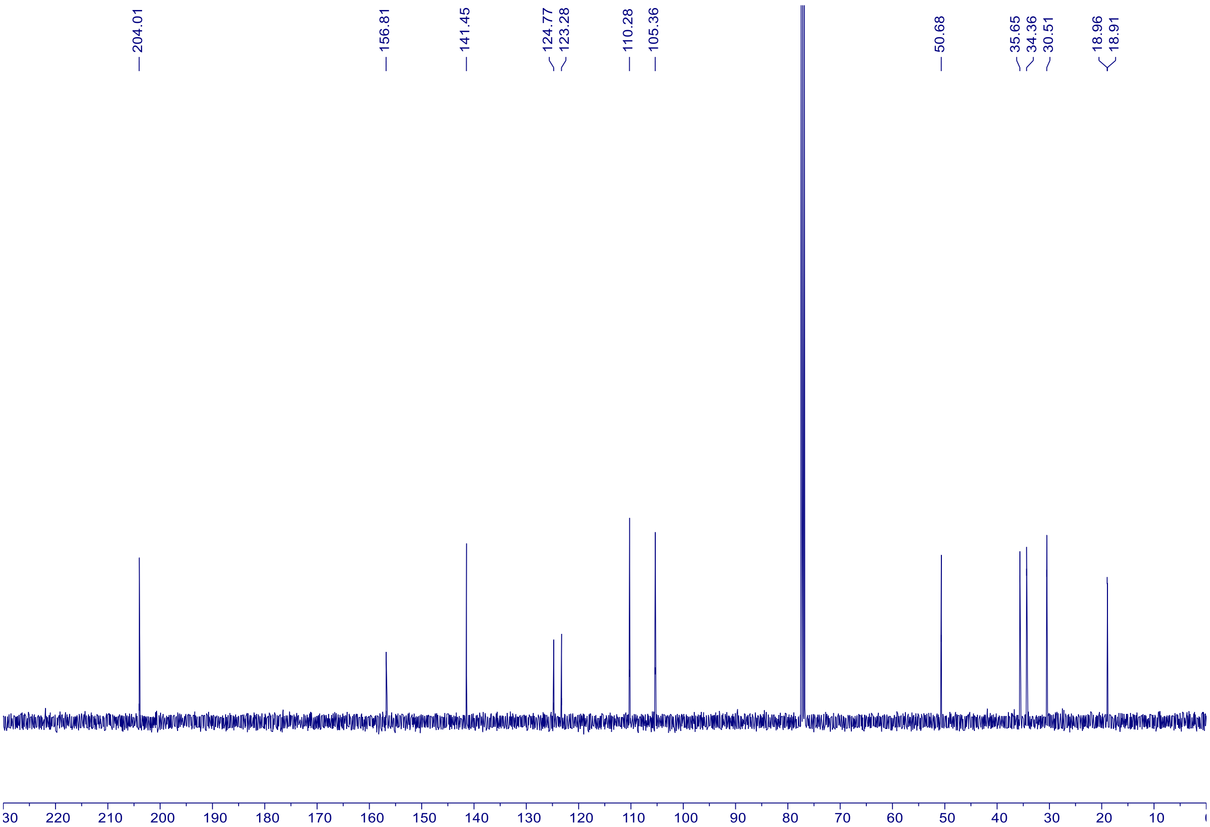

16s – ^{1}H NMR (400 MHz, $CDCl_3$)

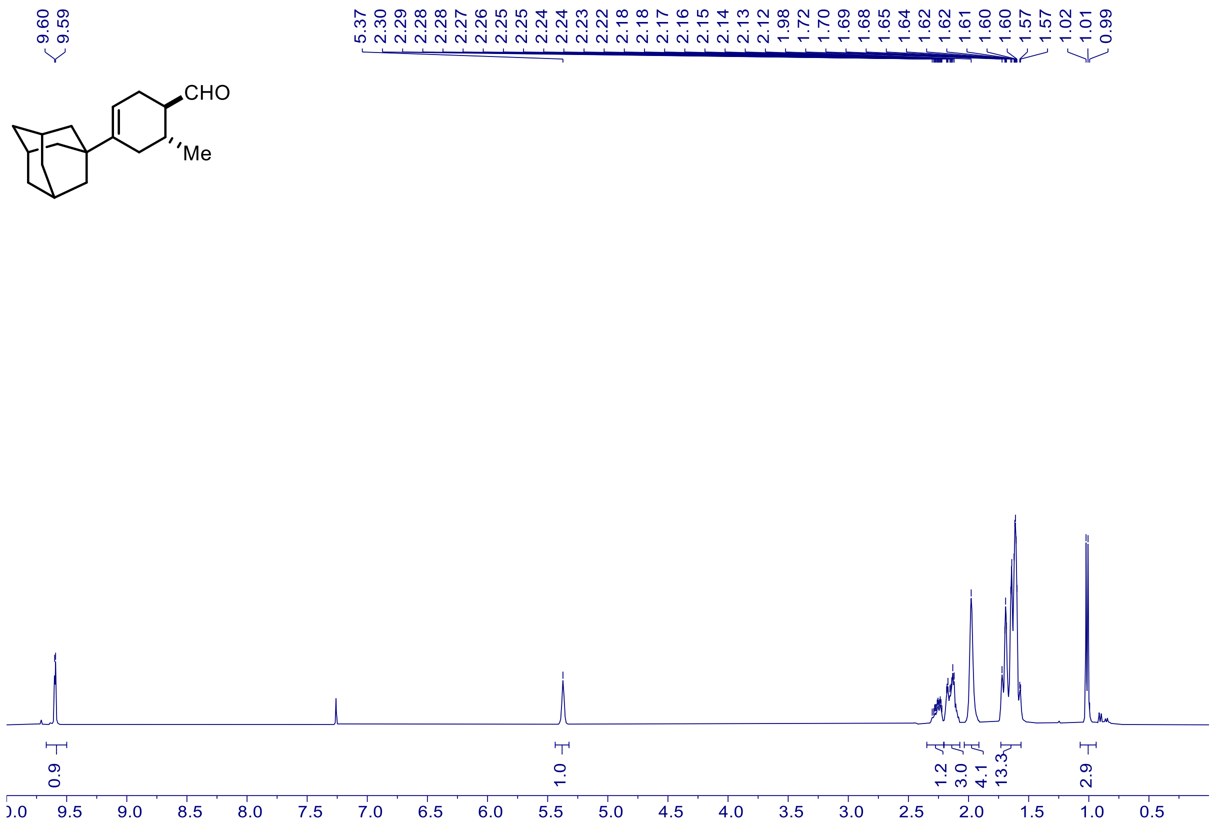

16s – ^{13}C NMR (101 MHz, $CDCl_3$)

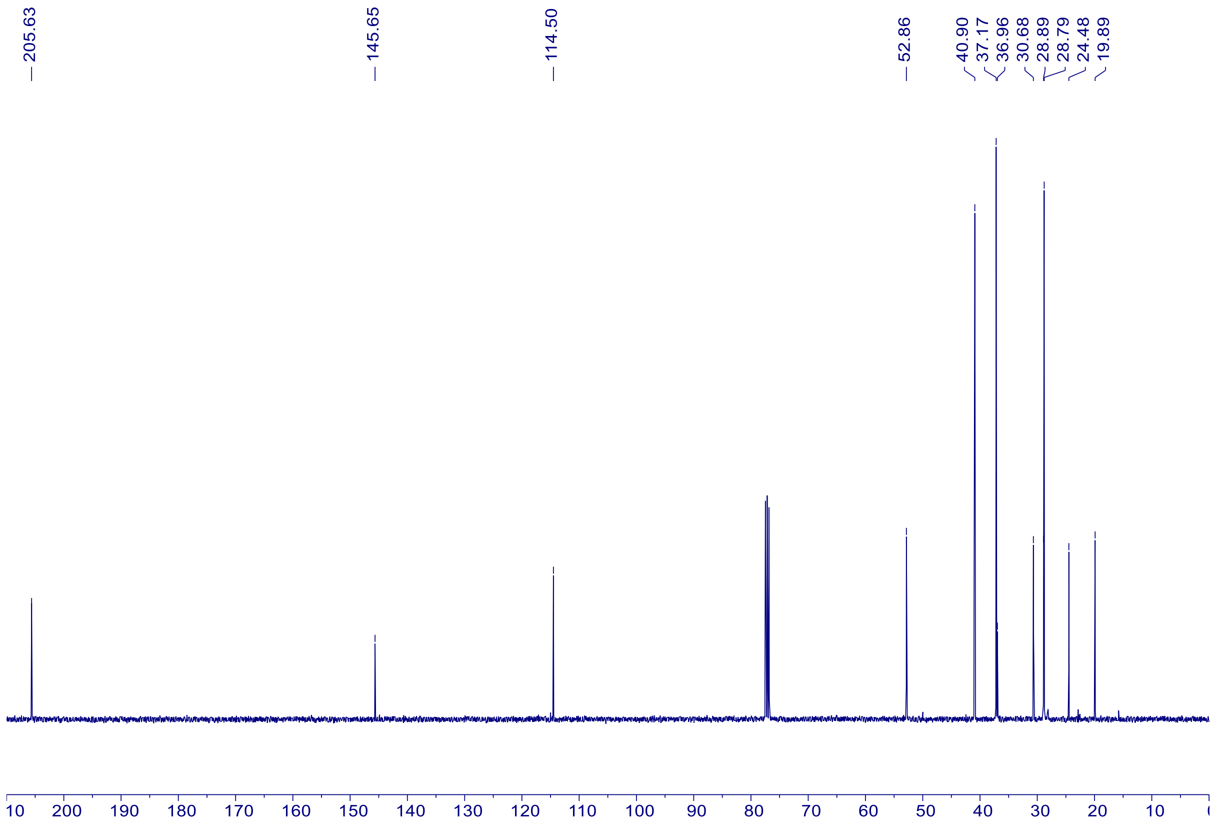

17s – ^{1}H NMR (400 MHz, $CDCl_3$)

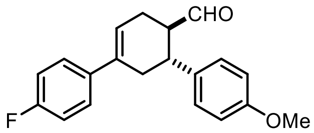

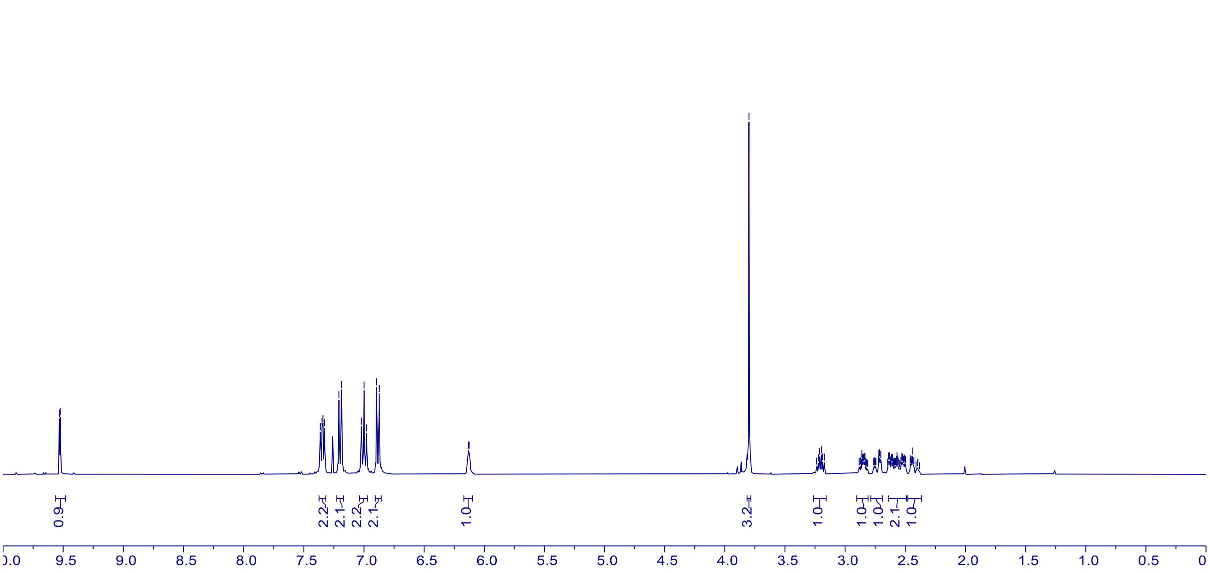

17s – ^{13}C NMR (101 MHz, $CDCl_3$)

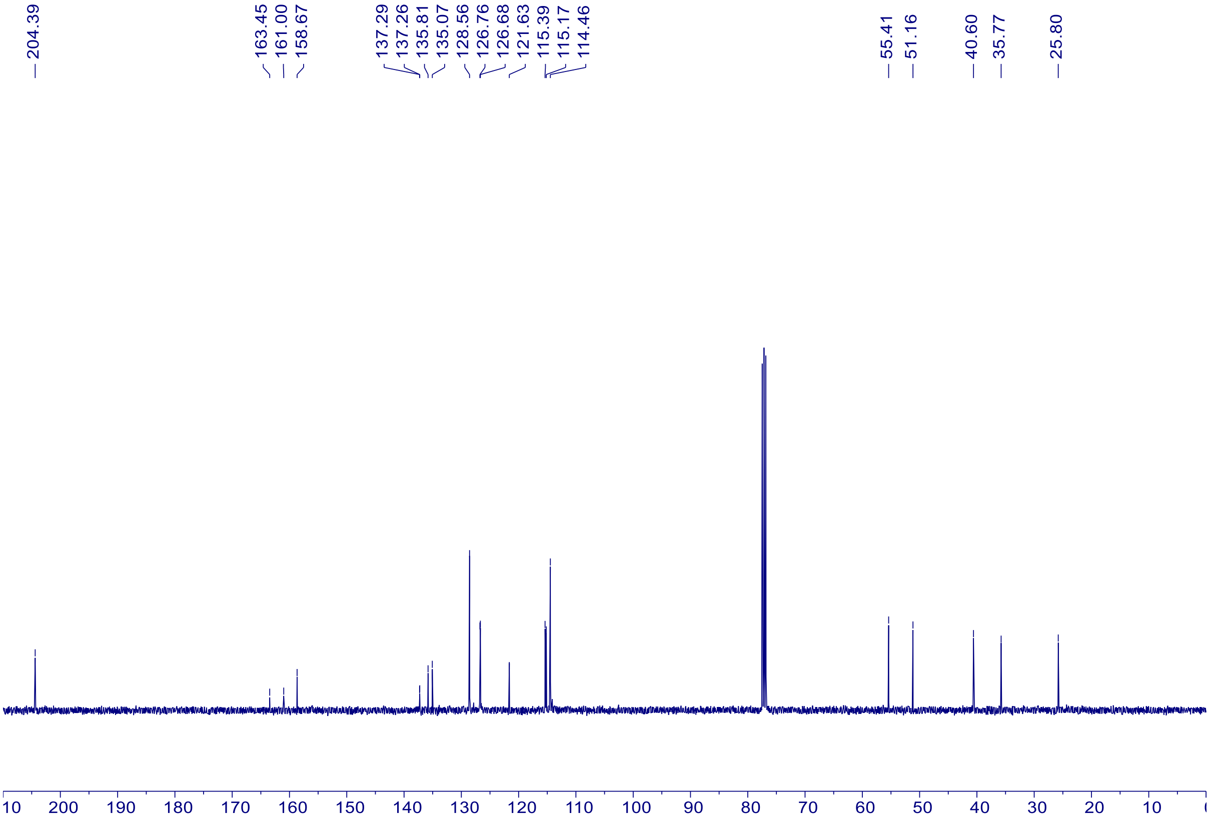

17s – ^{19}F NMR (376 MHz, $CDCl_3$)

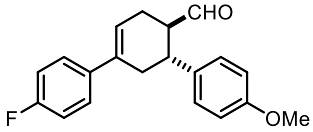

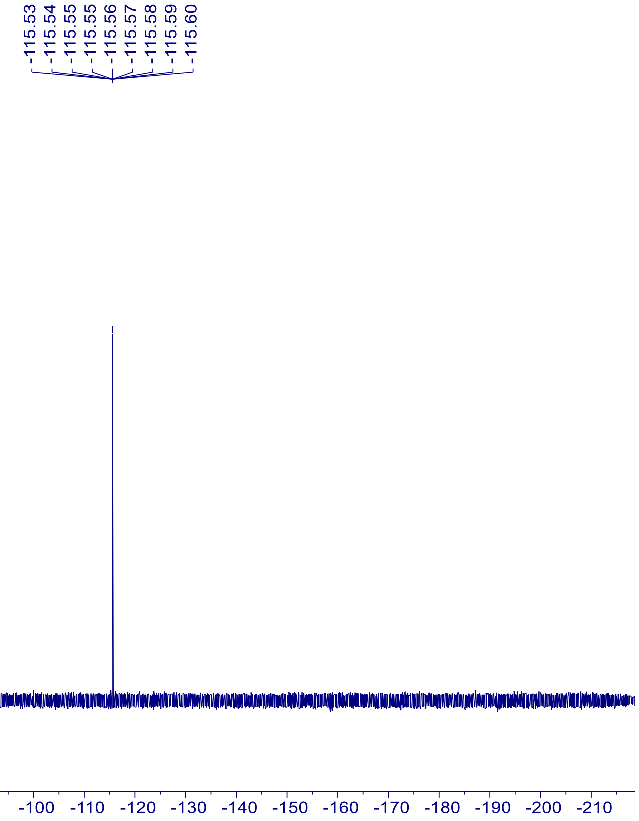

18s – ^{1}H NMR (400 MHz, $CDCl_3$)

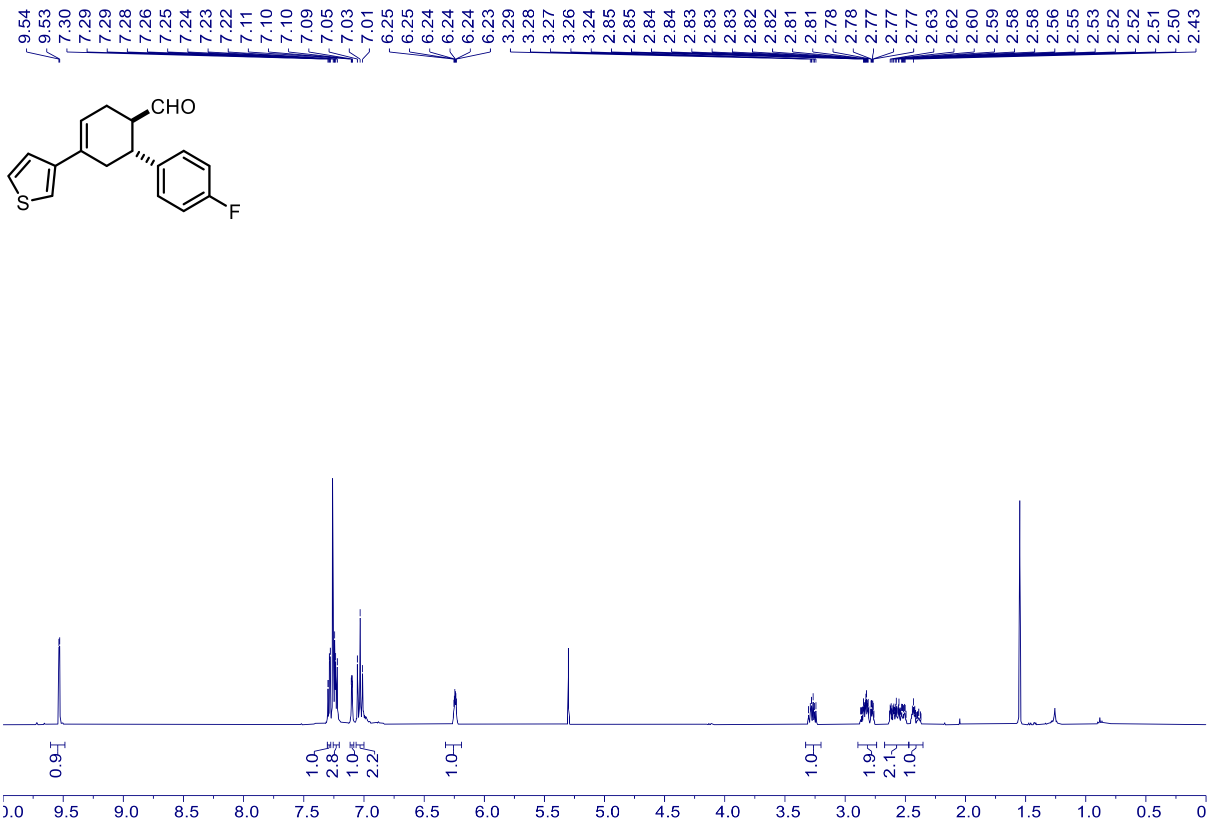

18s – ^{13}C NMR (101 MHz, $CDCl_3$)

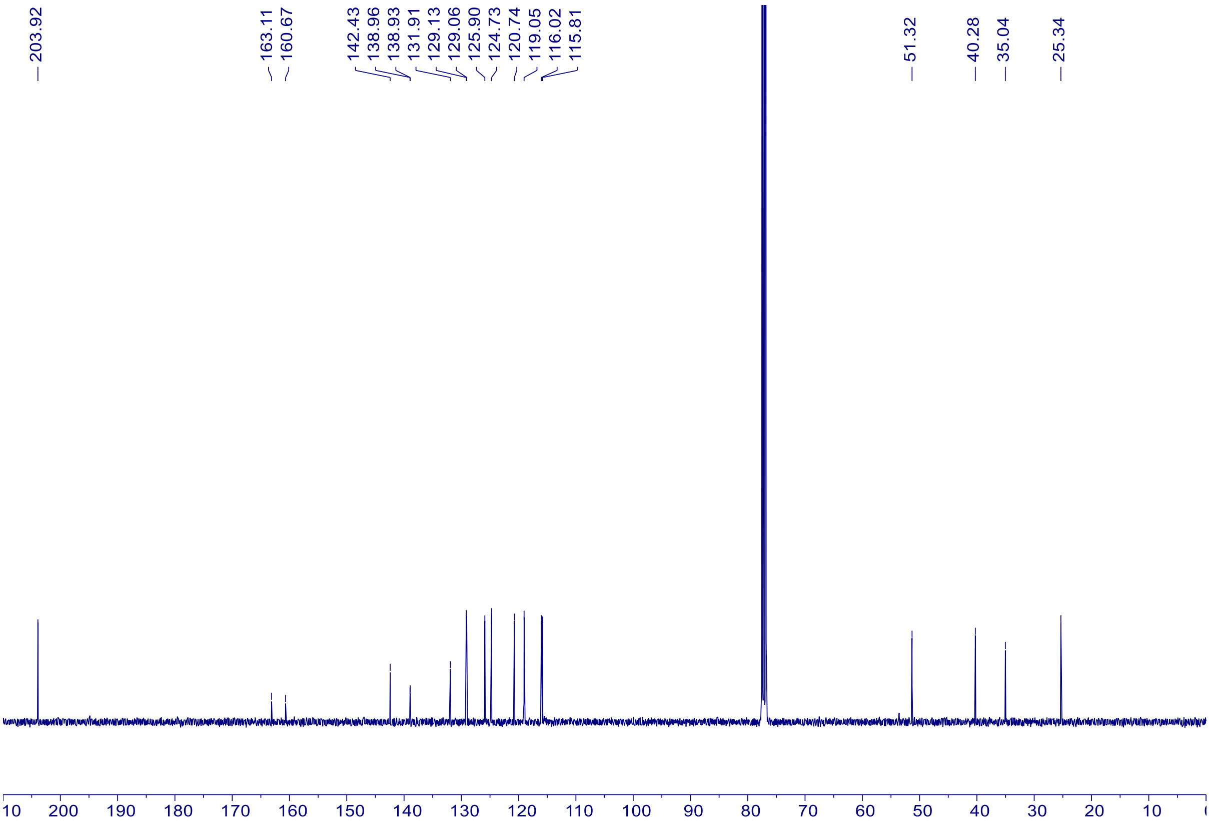

18s – ^{19}F NMR (376 MHz, $CDCl_3$)

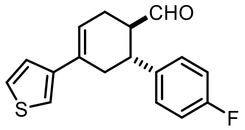

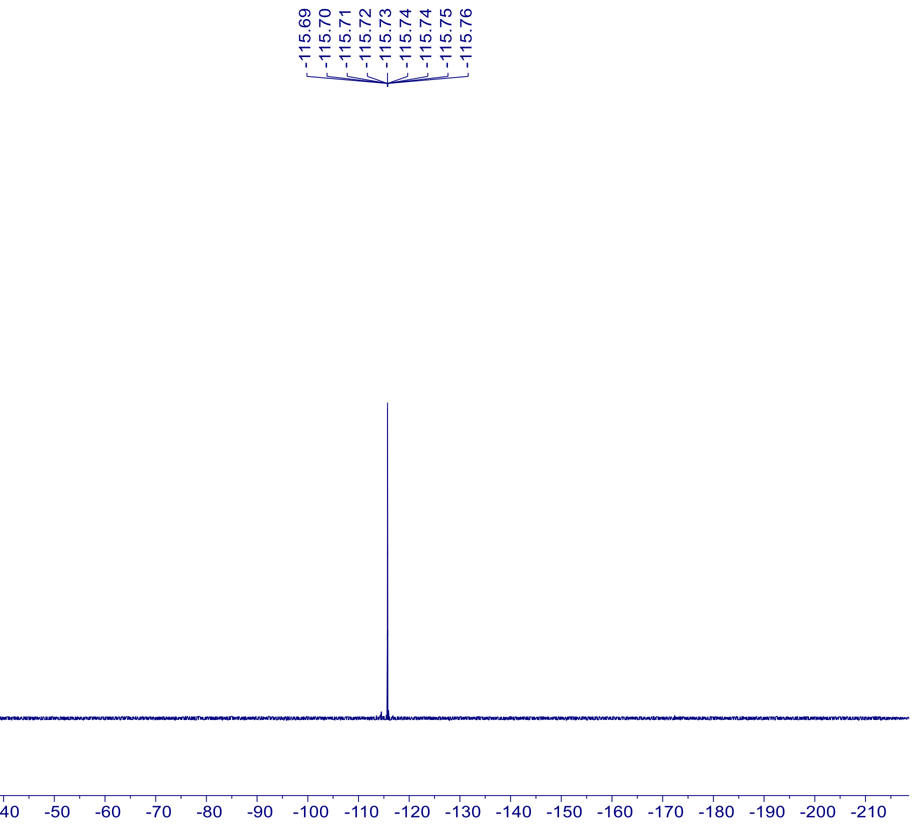

19s – ^{1}H NMR (400 MHz, $CDCl_3$)

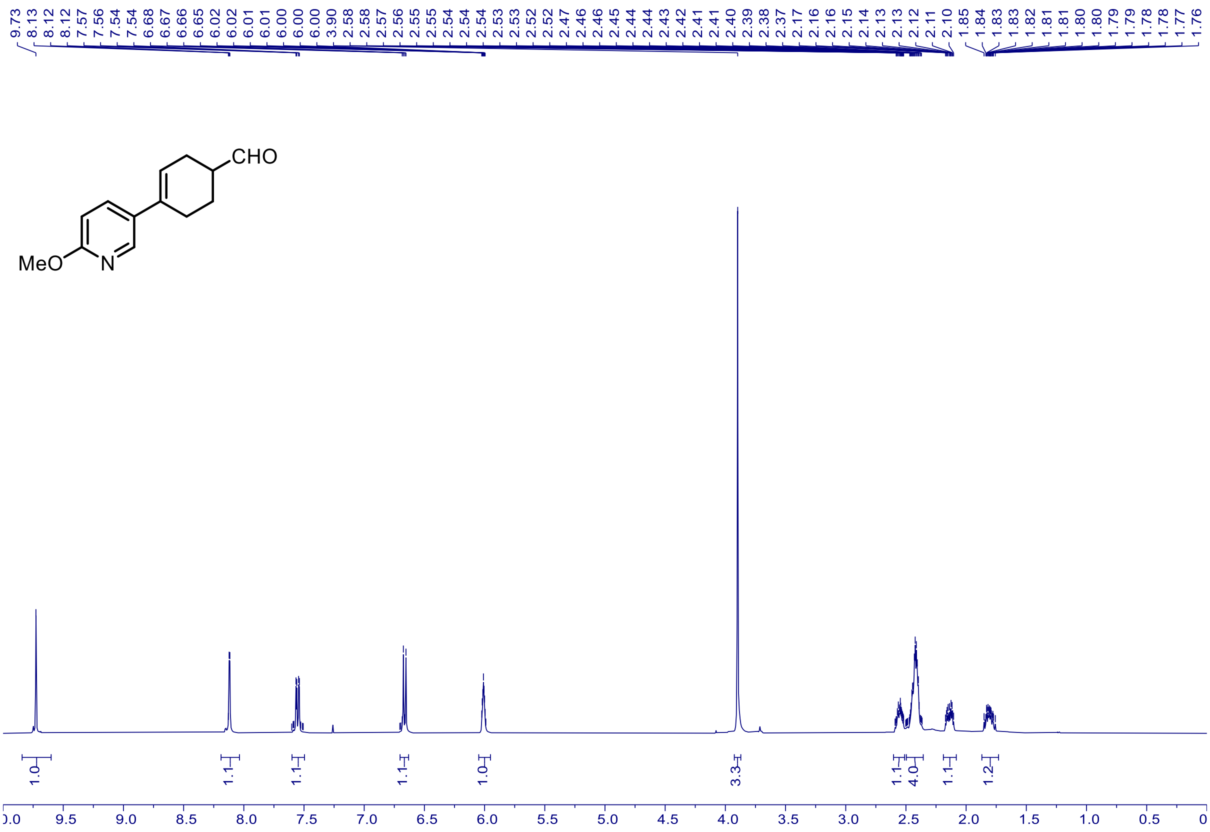

19s – ^{13}C NMR (101 MHz, $CDCl_3$)

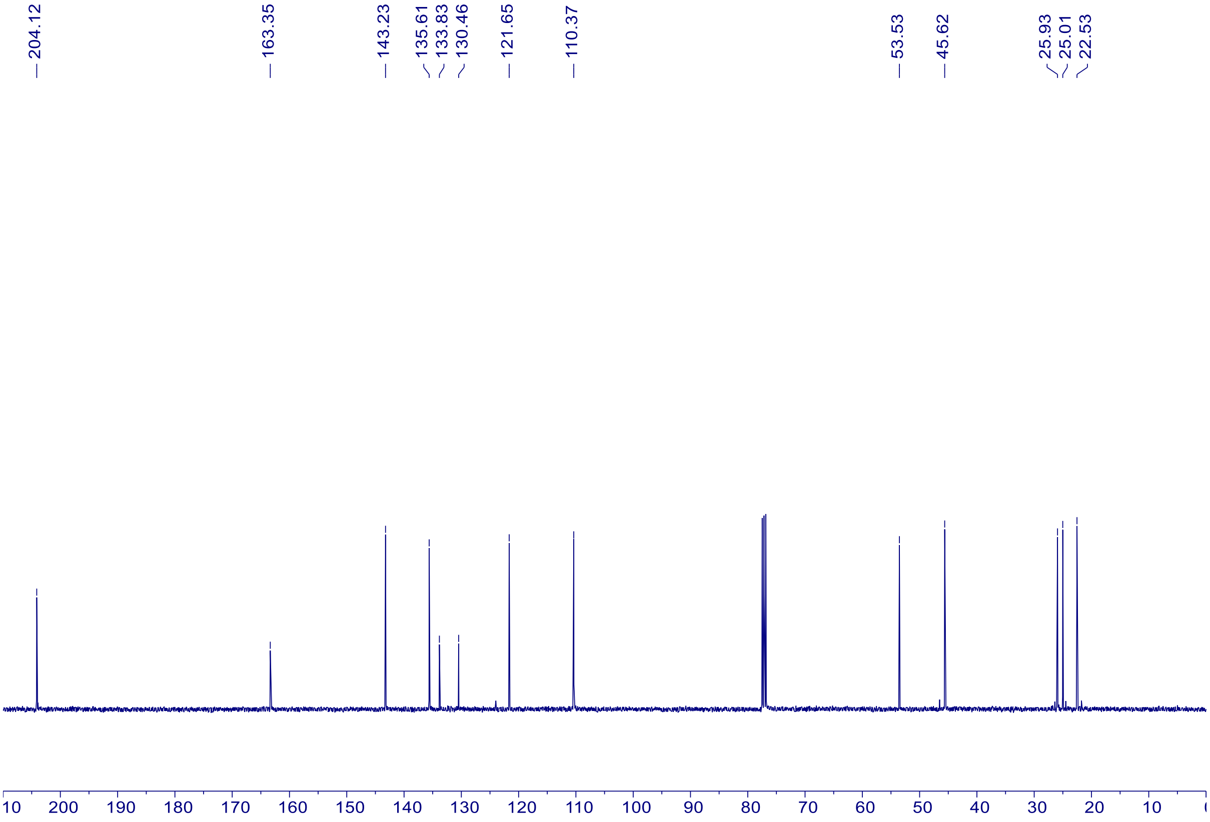

24 – ^{1}H NMR (400 MHz, $CDCl_3$)

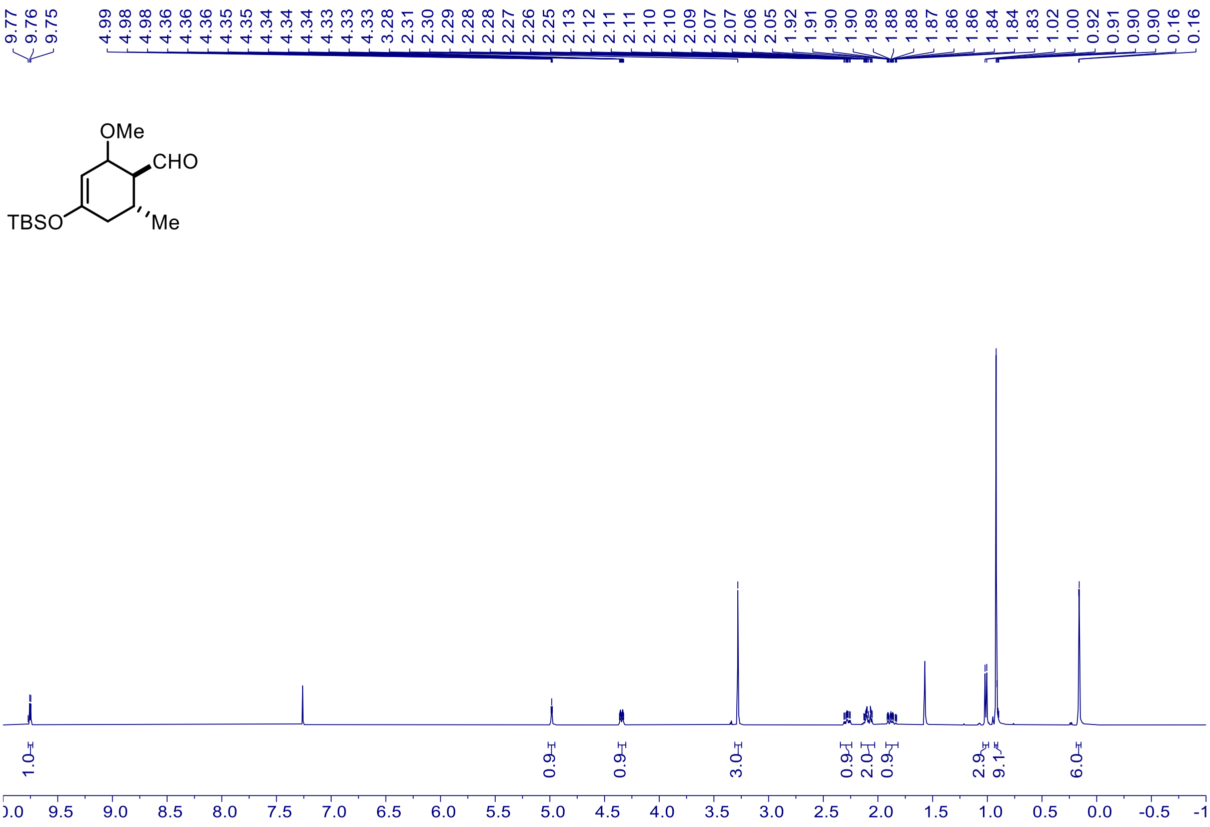

24 – ^{13}C NMR (101 MHz, $CDCl_3$)

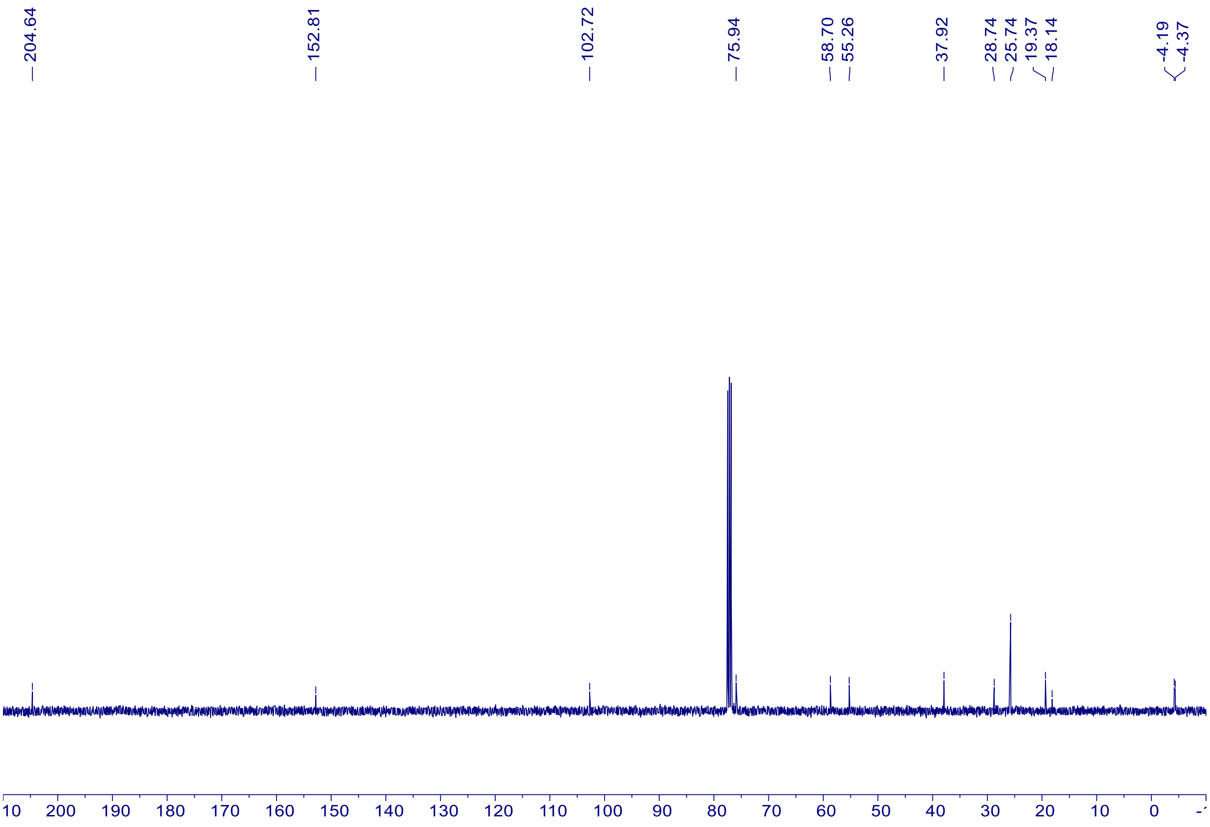

S13 – ^{1}H NMR (500 MHz, $CDCl_3$)

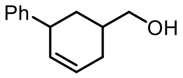

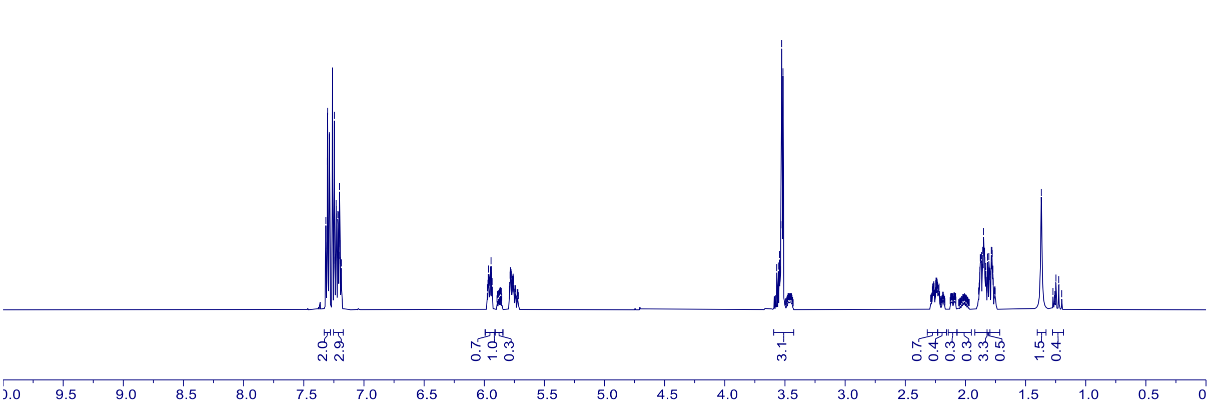

S13 – ^{13}C NMR (126 MHz, $CDCl_3$)

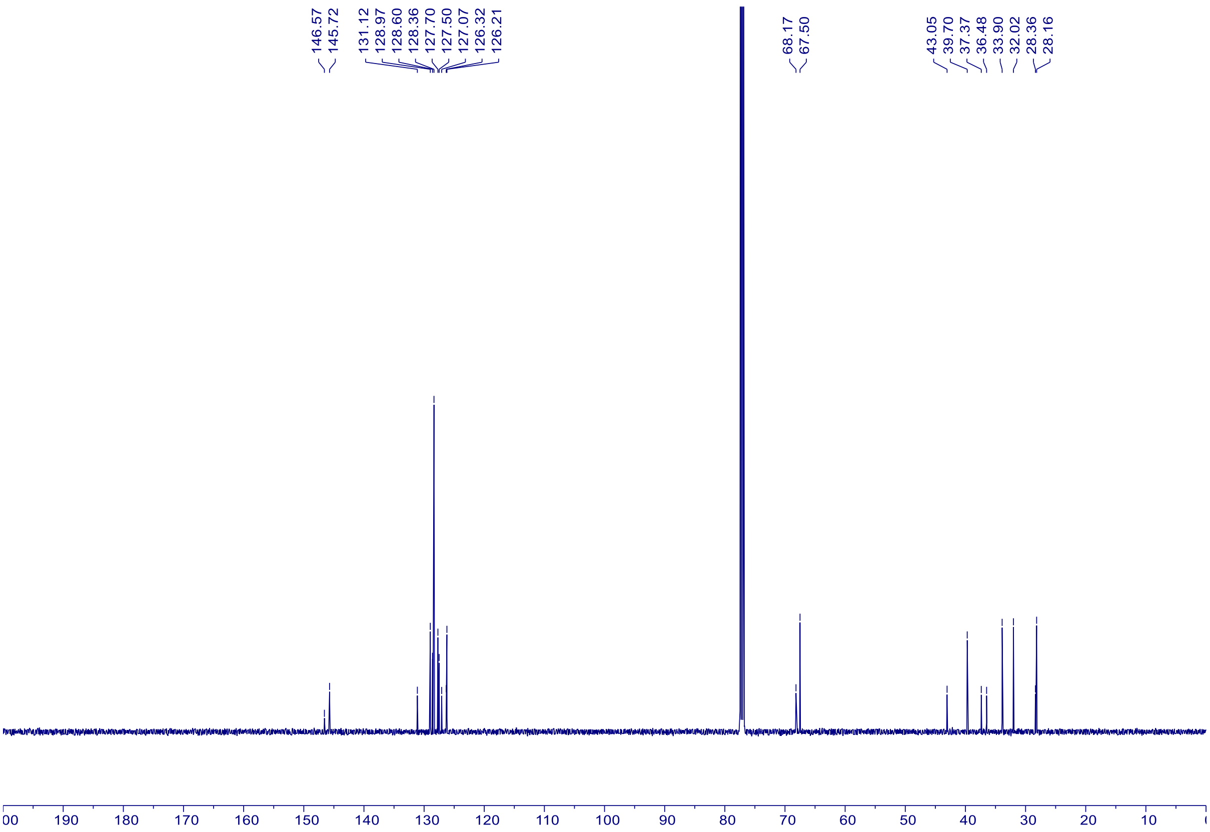

26s – ^{1}H NMR (400 MHz, $CDCl_3$)

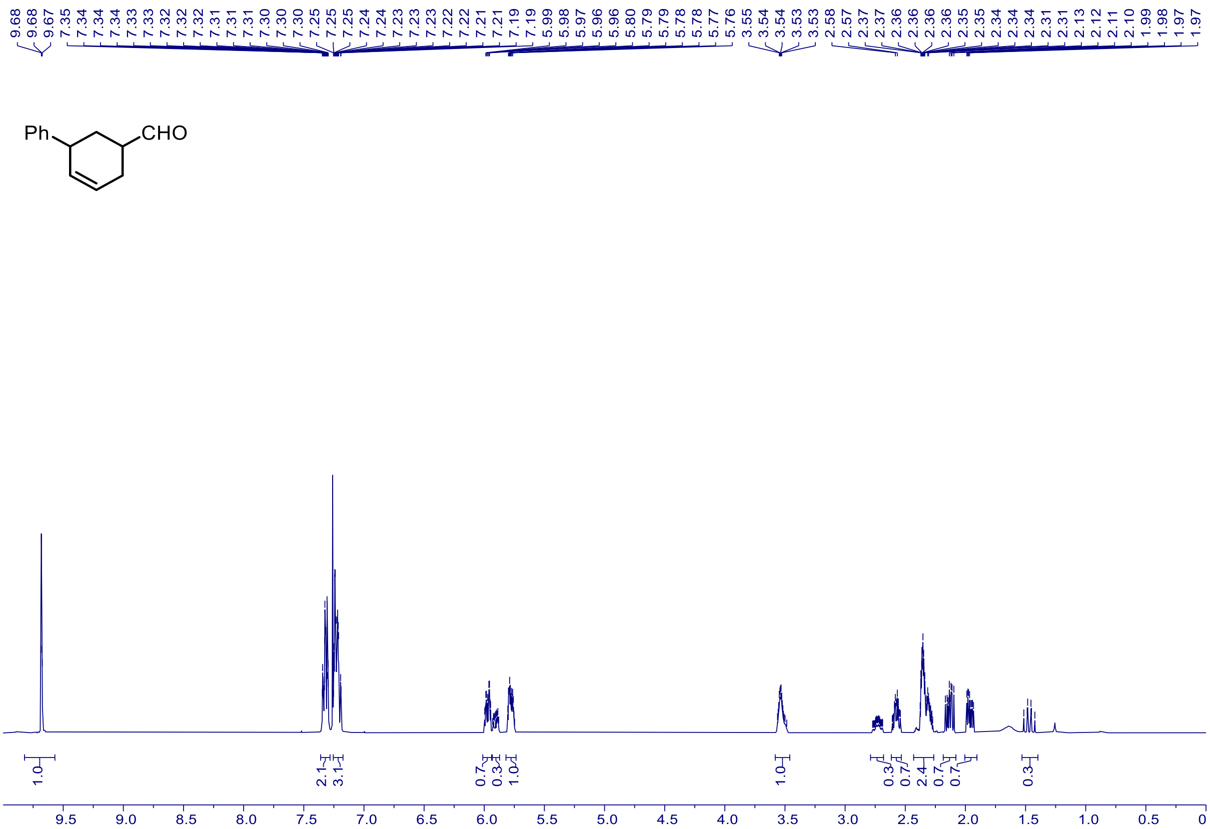

26s – ^{13}C NMR (101 MHz, $CDCl_3$)

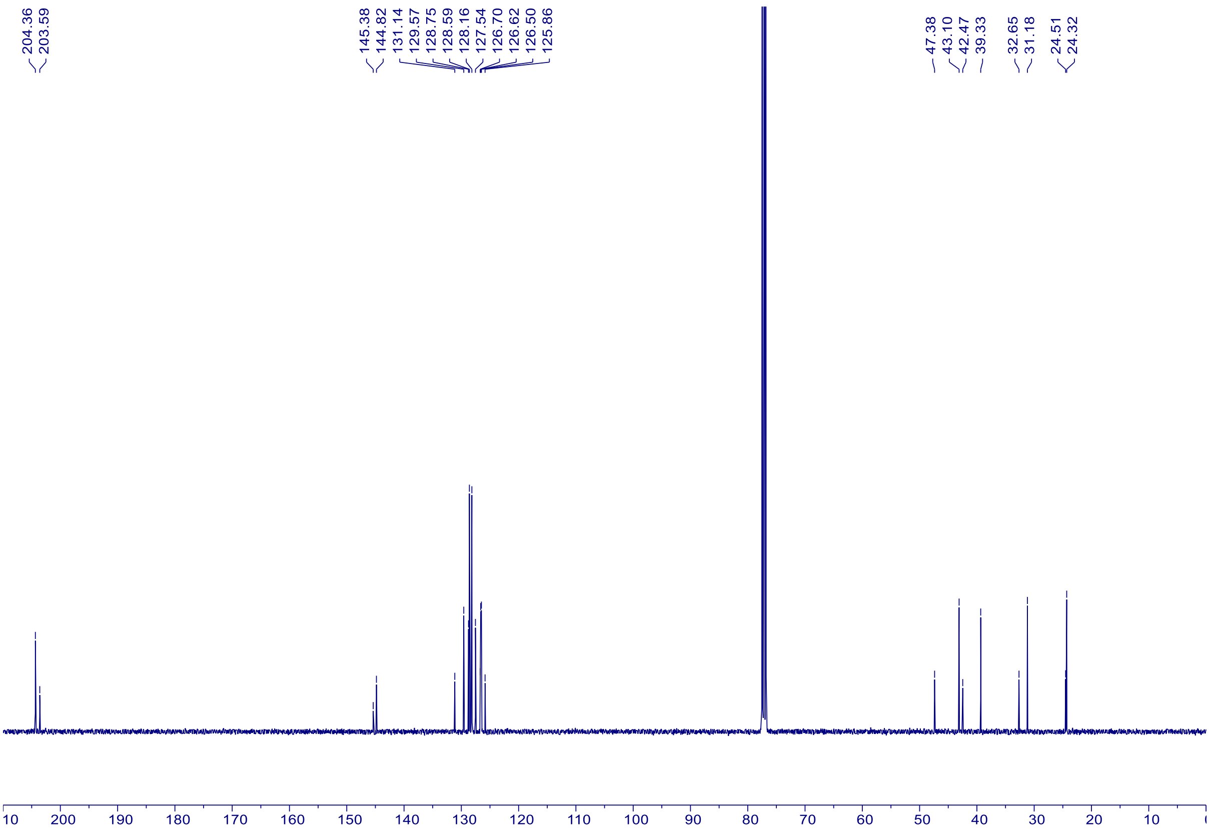

28s – ^{1}H NMR (400 MHz, $CDCl_3$)

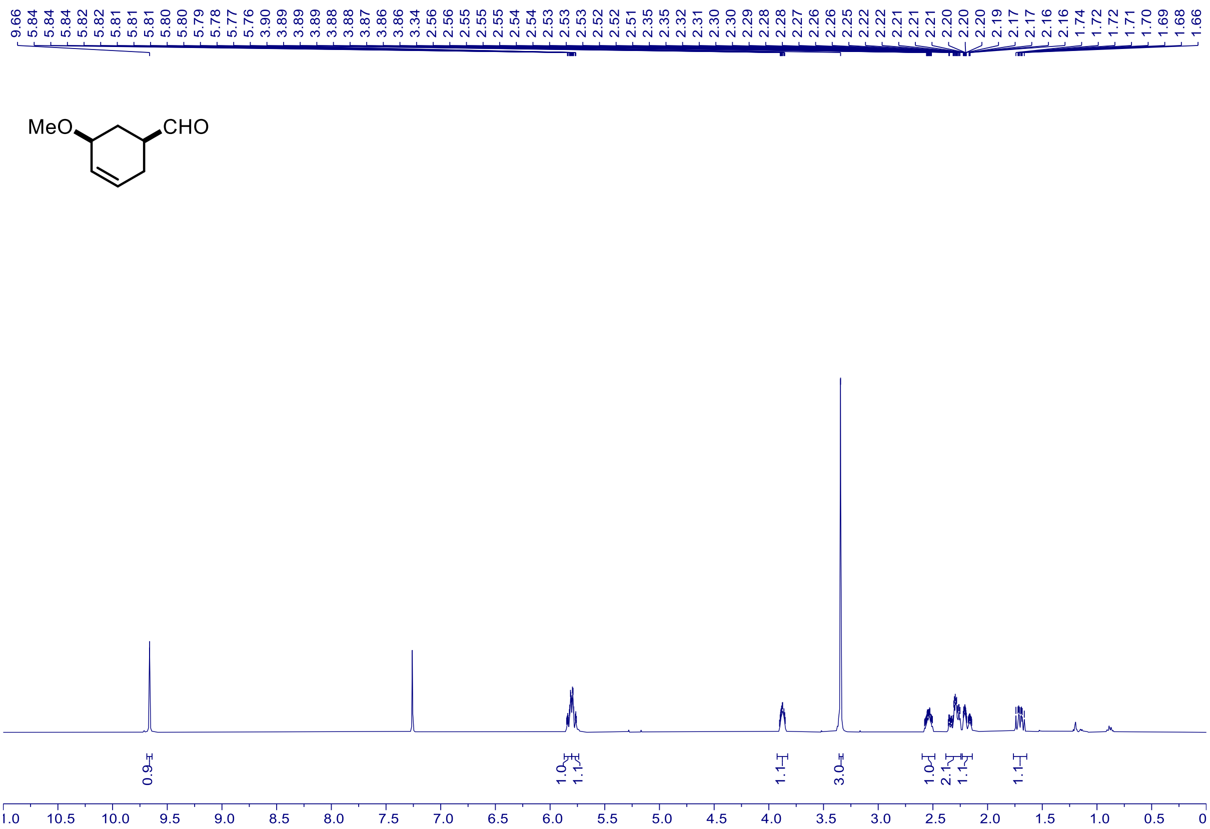

28s – ^{13}C NMR (101 MHz, $CDCl_3$)

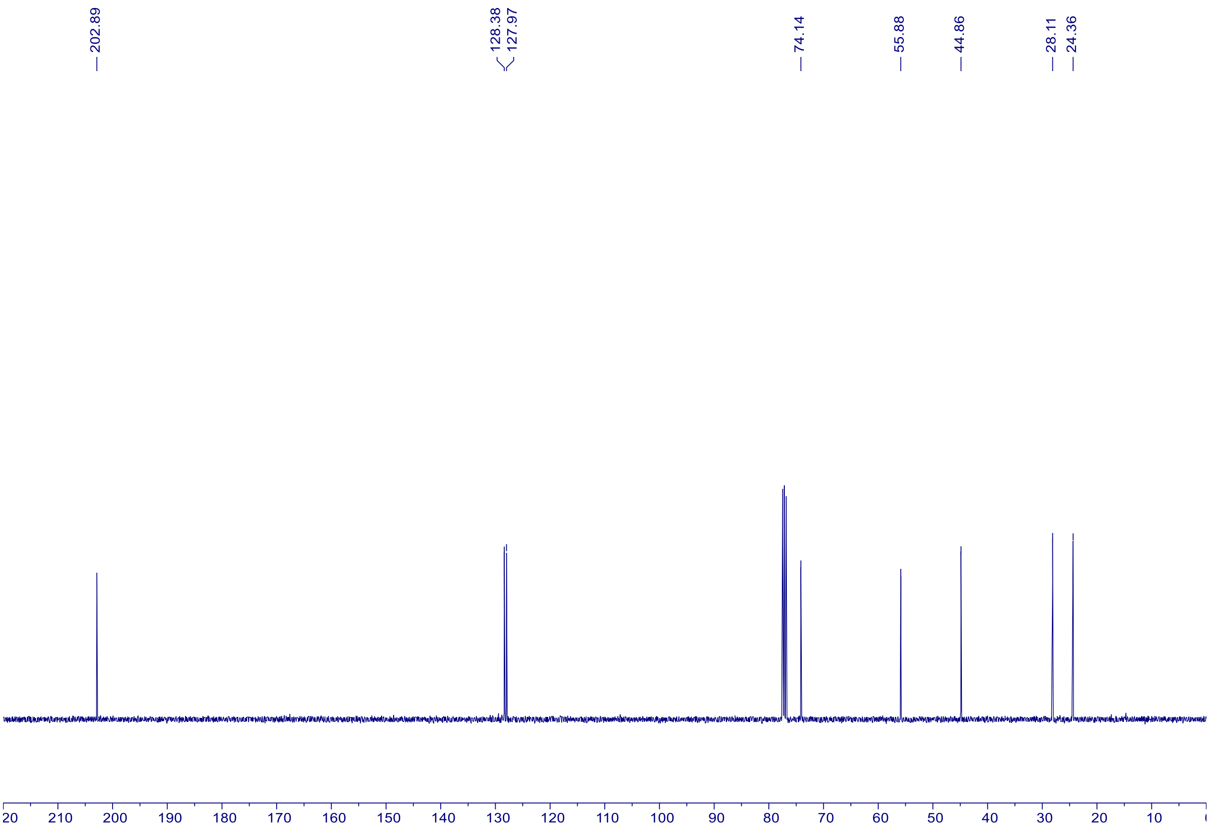

29s – ^{1}H NMR (400 MHz, $CDCl_3$)

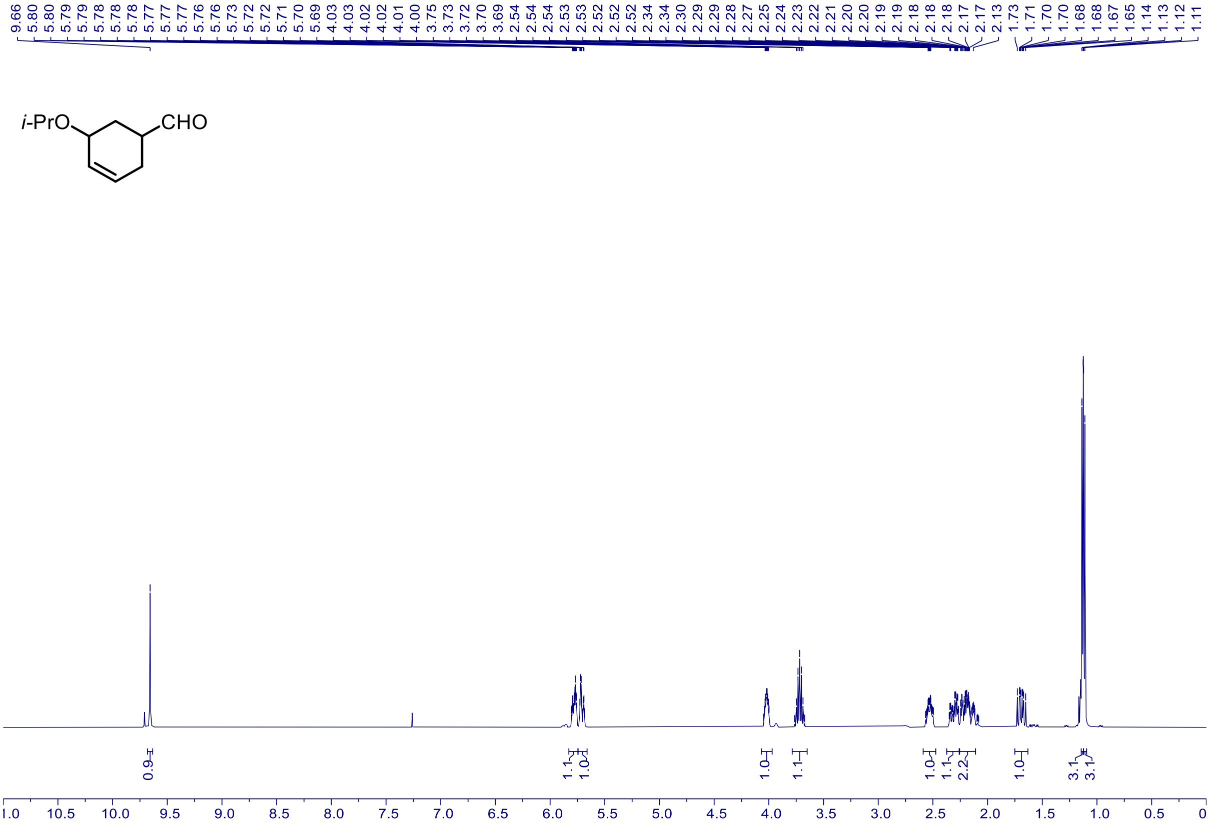

29s – ^{13}C NMR (101 MHz, $CDCl_3$)

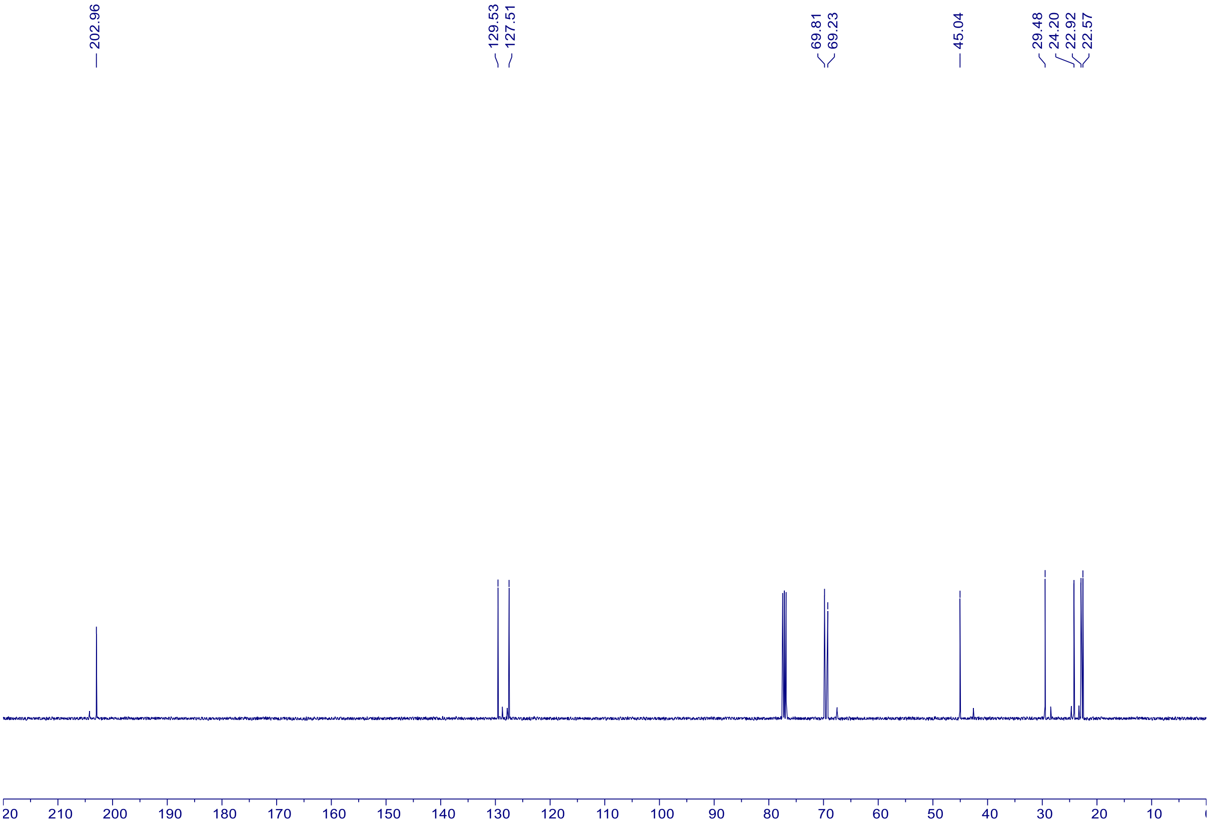

30s – ^{1}H NMR (400 MHz, $CDCl_3$)

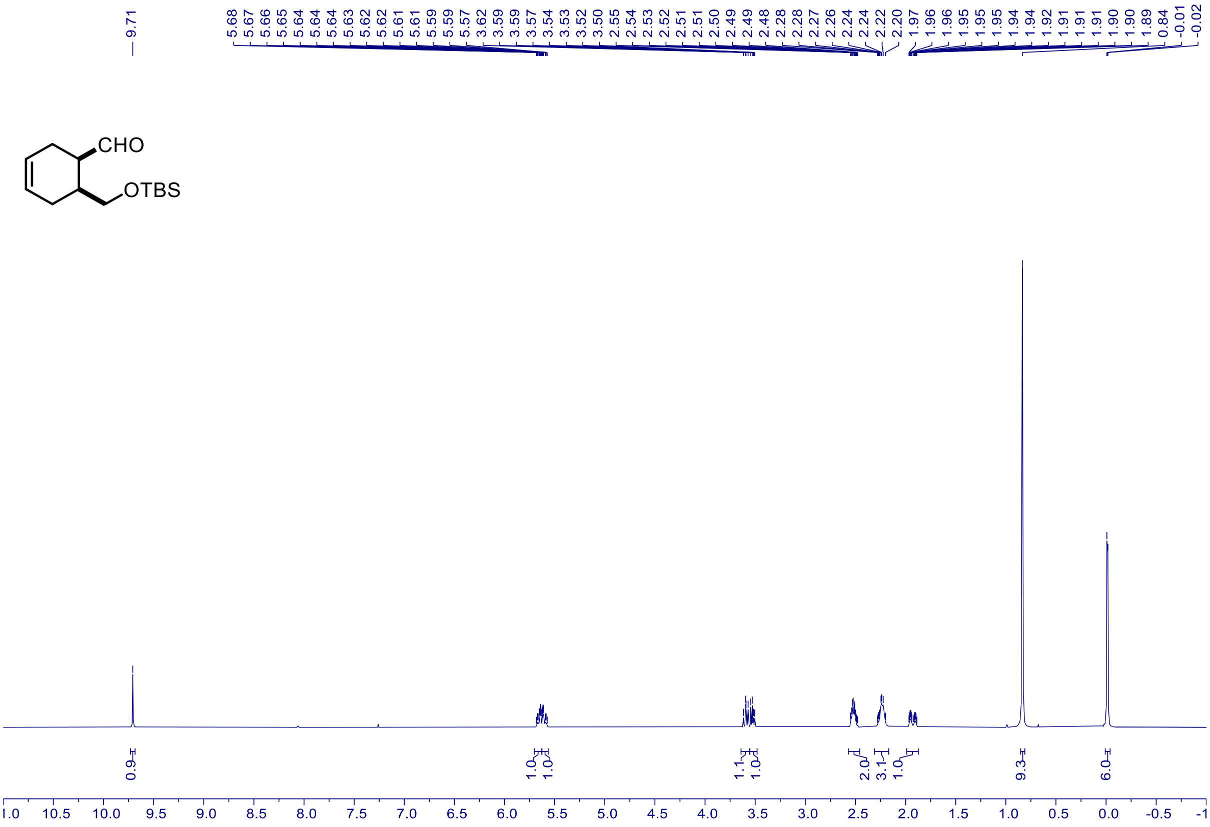

30s – ^{13}C NMR (101 MHz, $CDCl_3$)

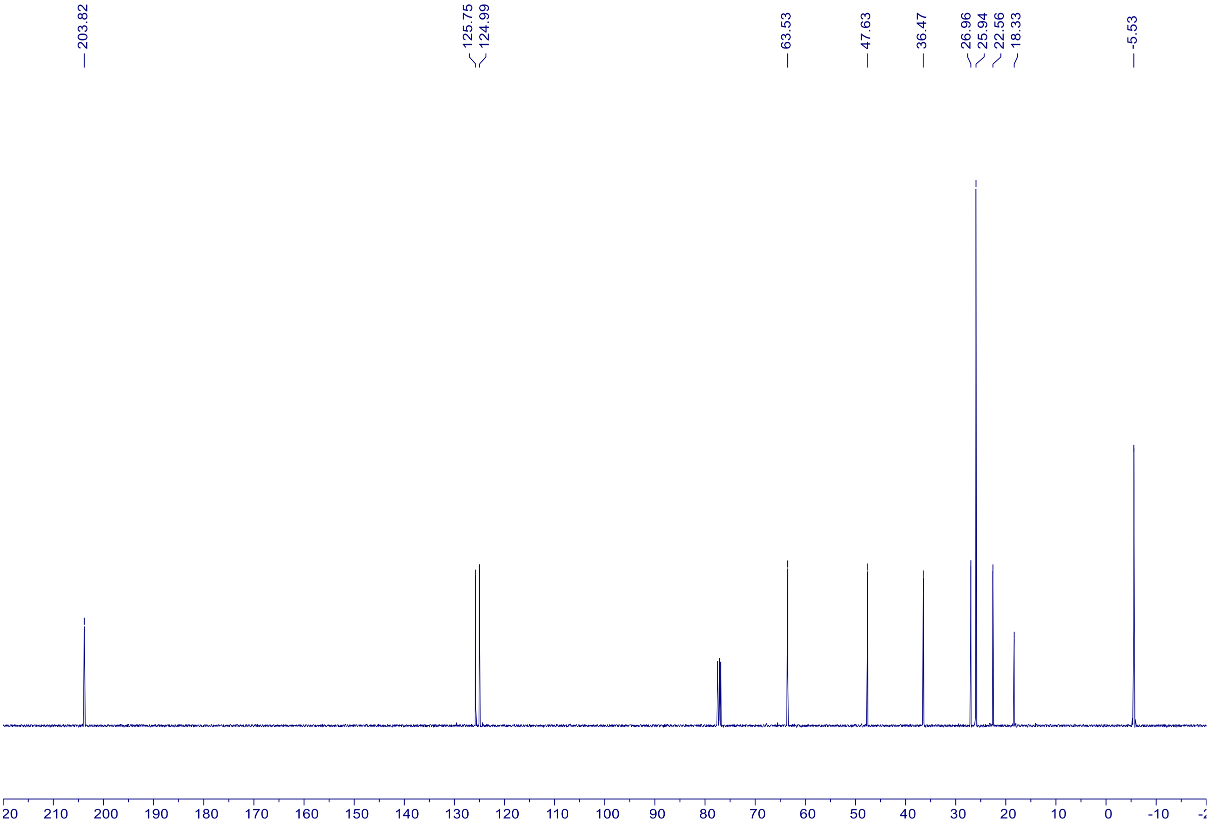

S22 – ^{1}H NMR (500 MHz, CDCl3)

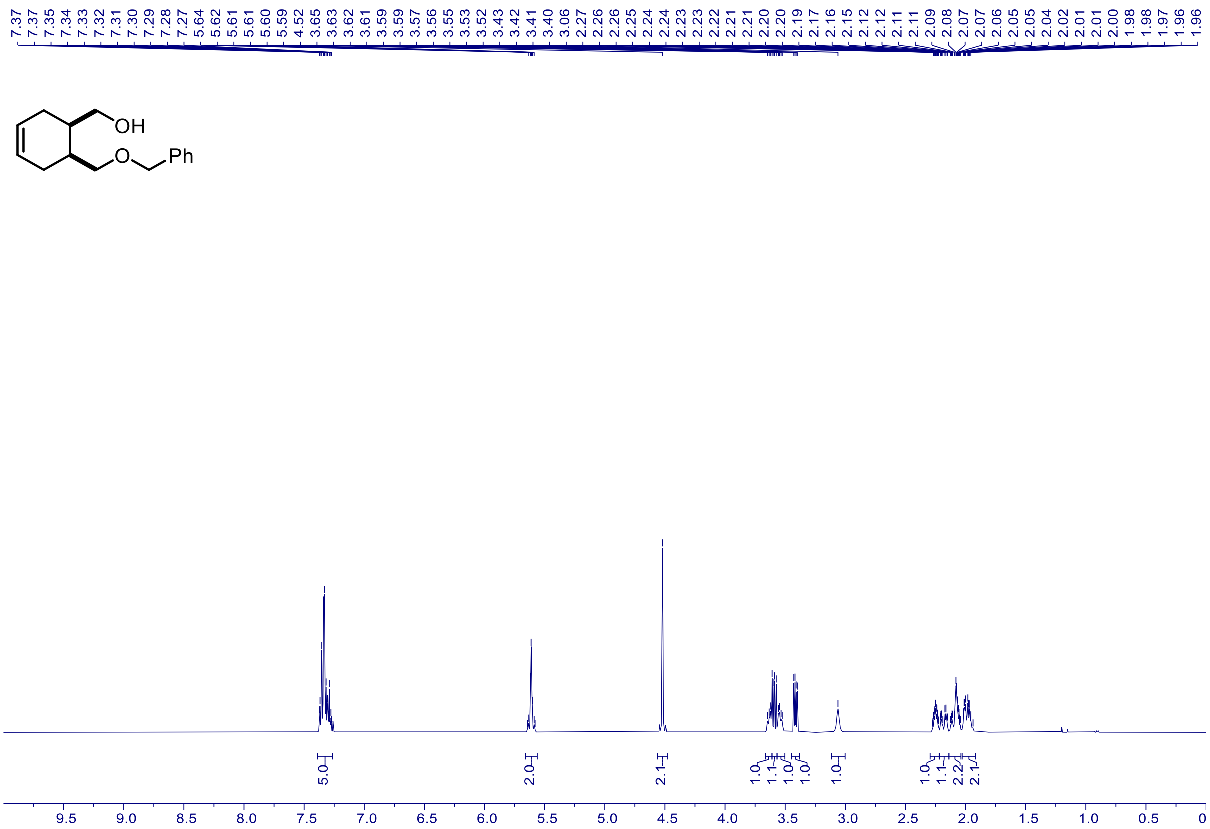

S22 – ^{13}C NMR (126 MHz, CDCl3)

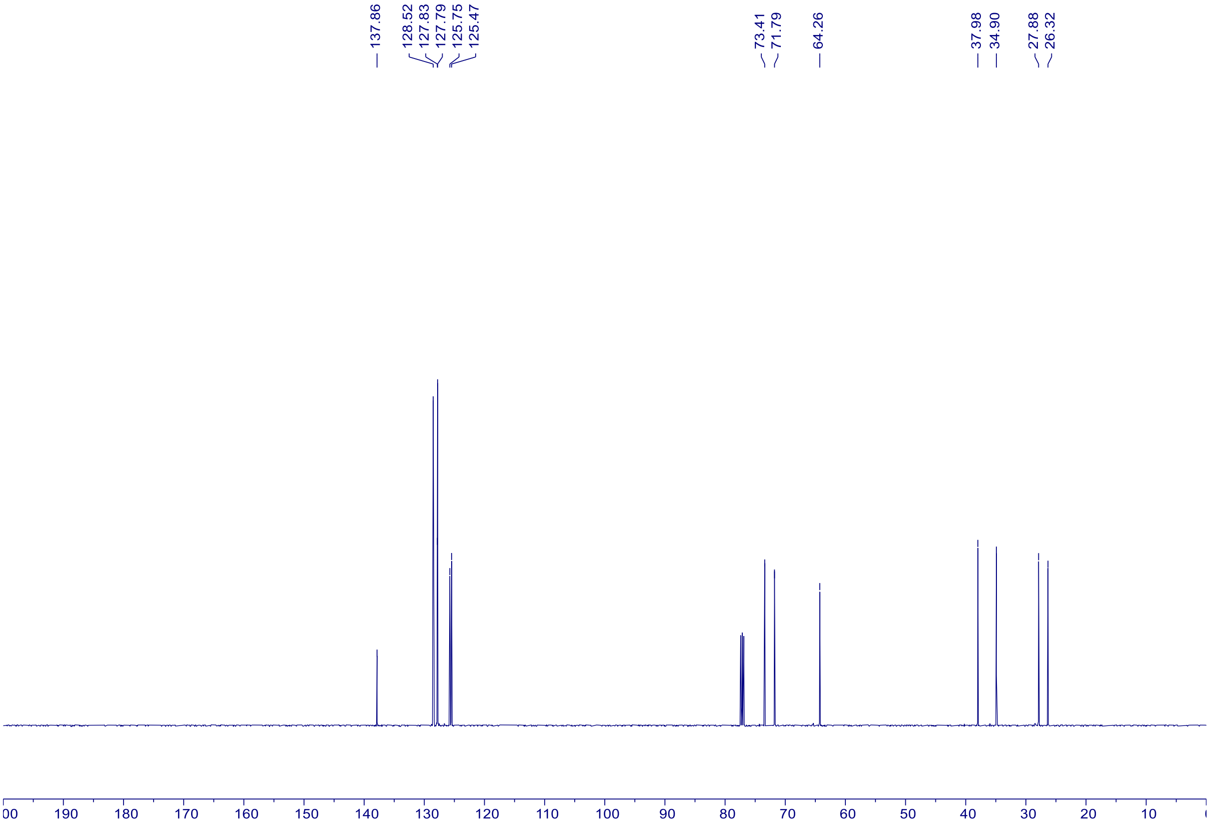

31s – ^{1}H NMR (500 MHz, $CDCl_3$)

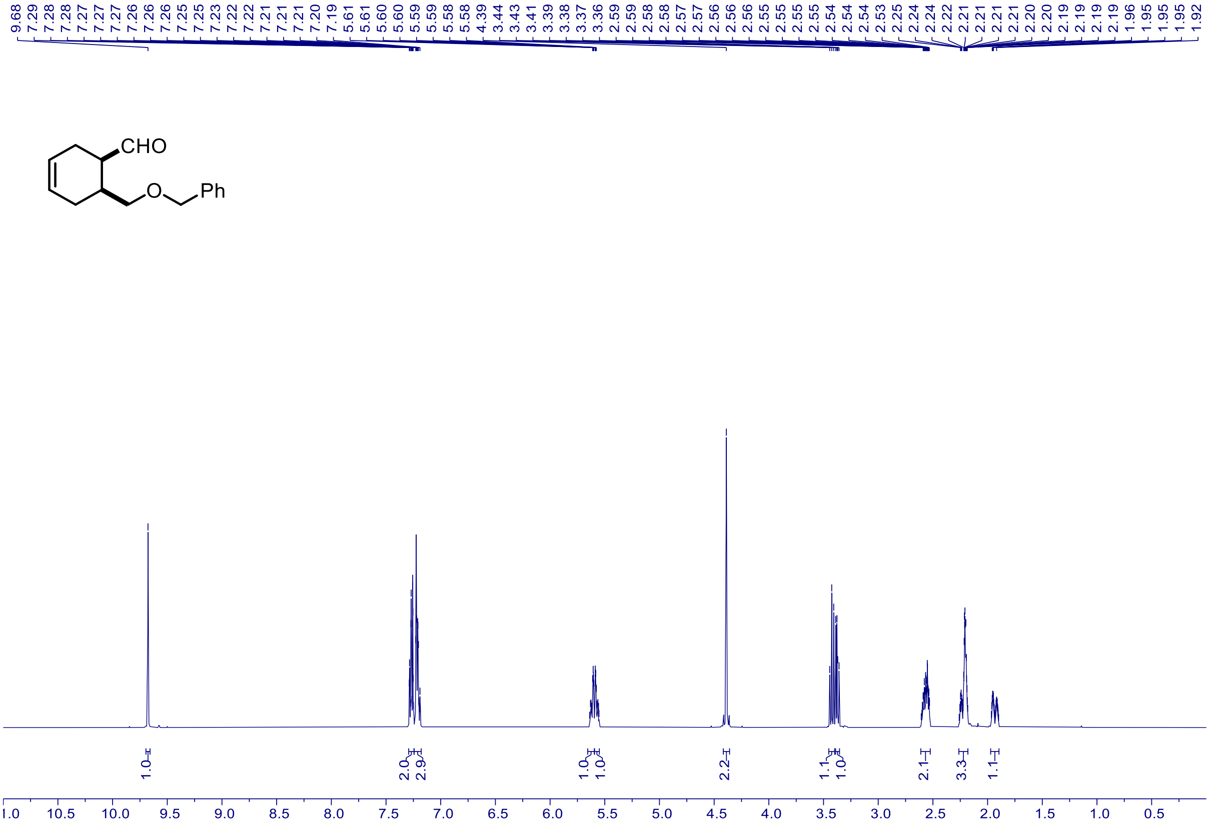

31s – ^{13}C NMR (126 MHz, $CDCl_3$)

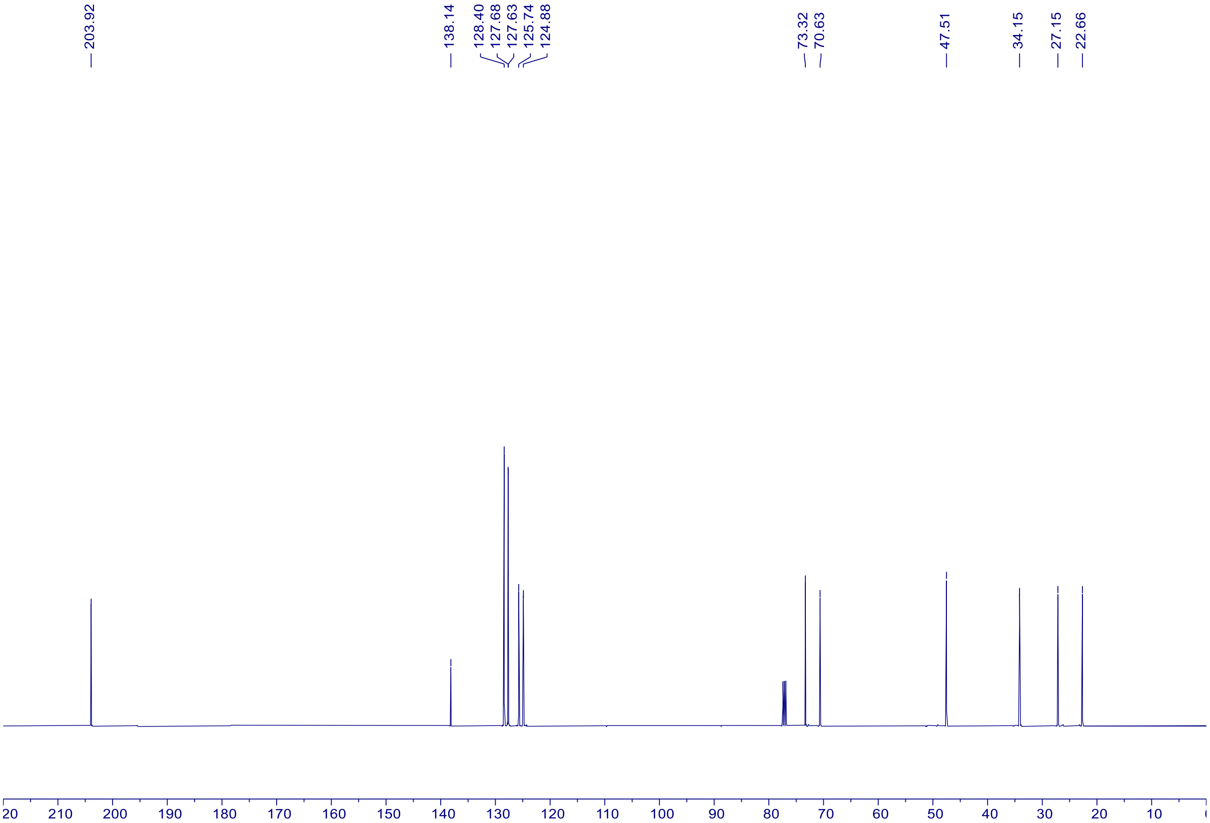

S24 – ^{1}H NMR (400 MHz, CDCl3)

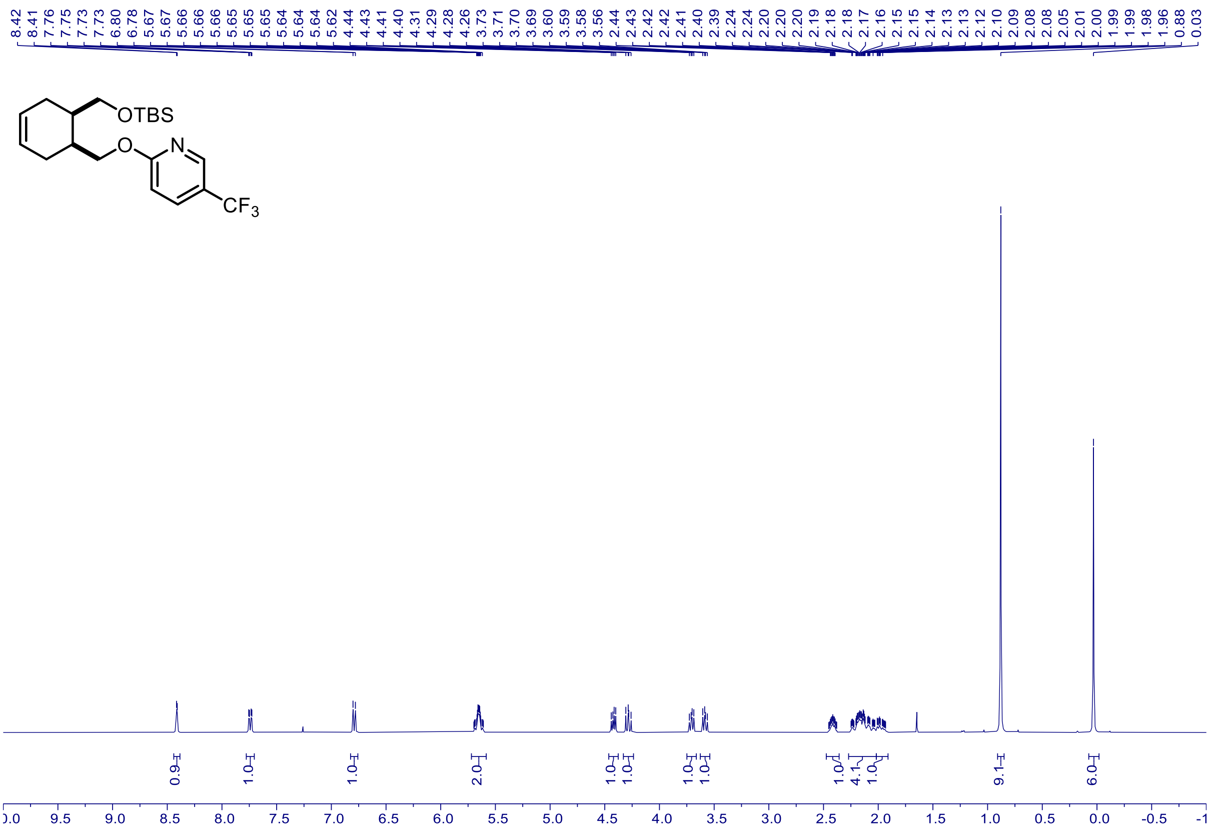

S24 – ^{13}C NMR (101 MHz, CDCl3)

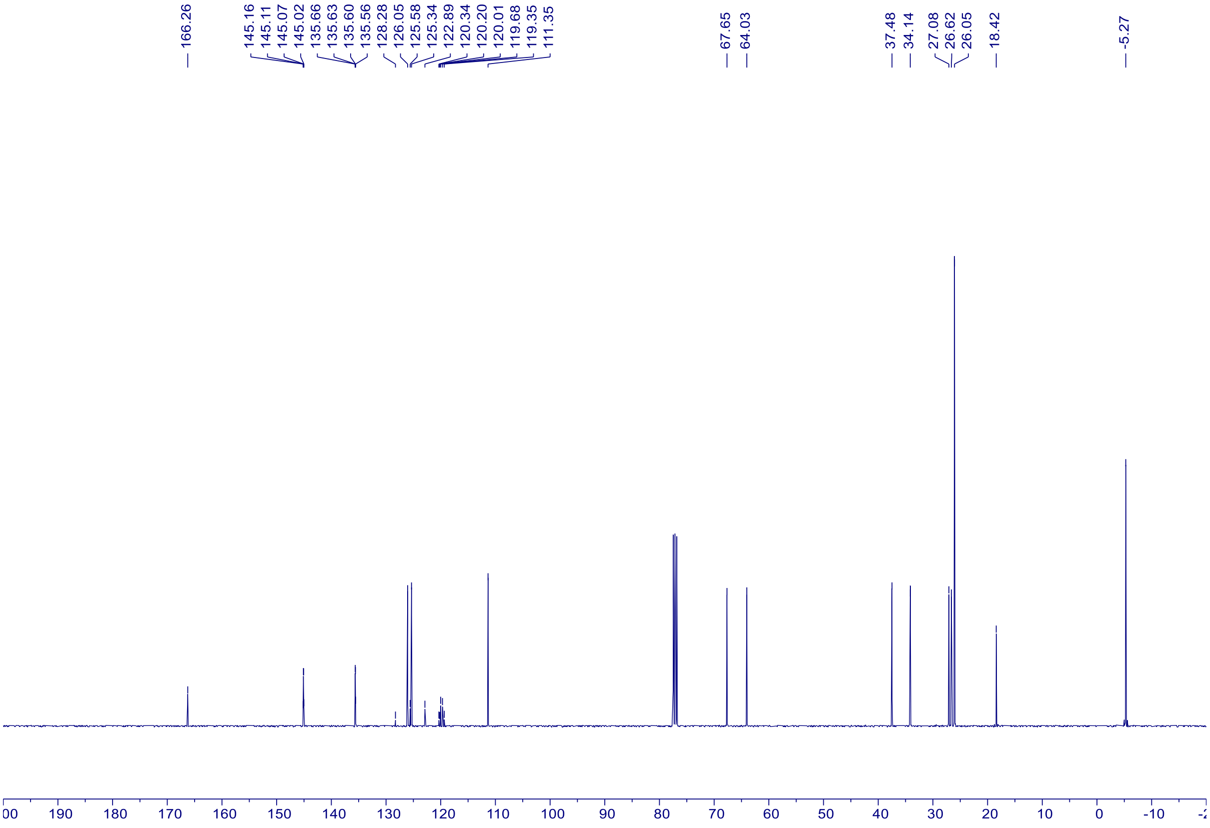

S24 – ^{19}F NMR (376 MHz, $CDCl_3$)

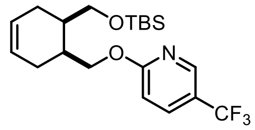

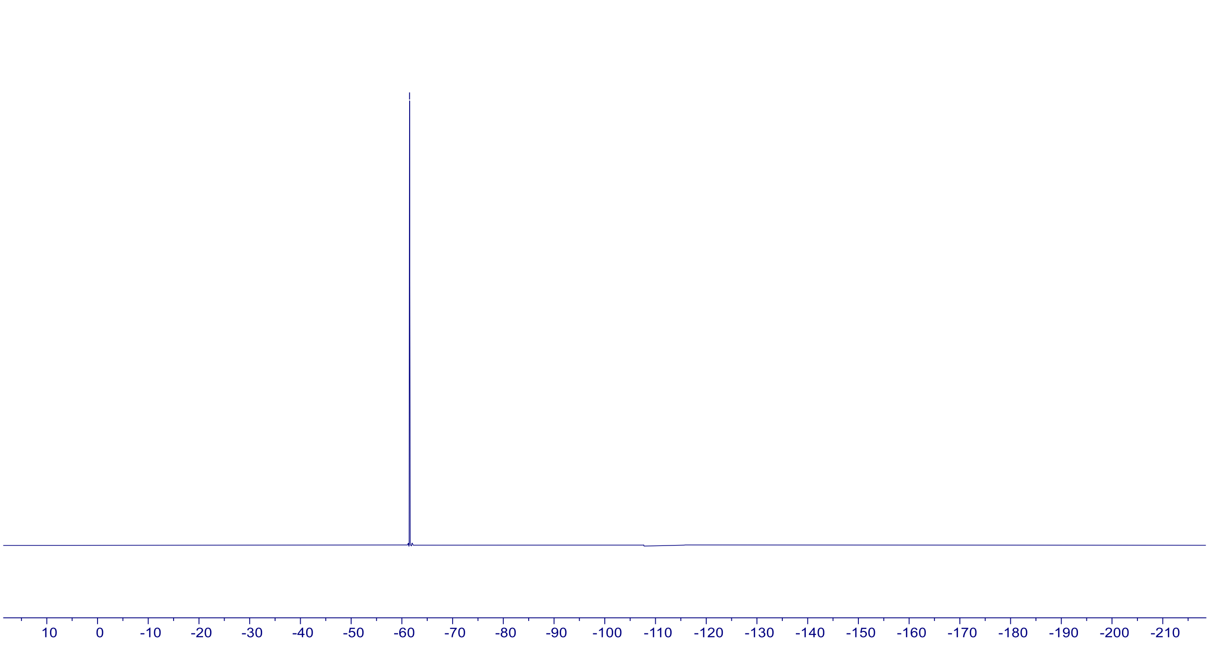

S25 – ^{1}H NMR (400 MHz, $CDCl_3$)

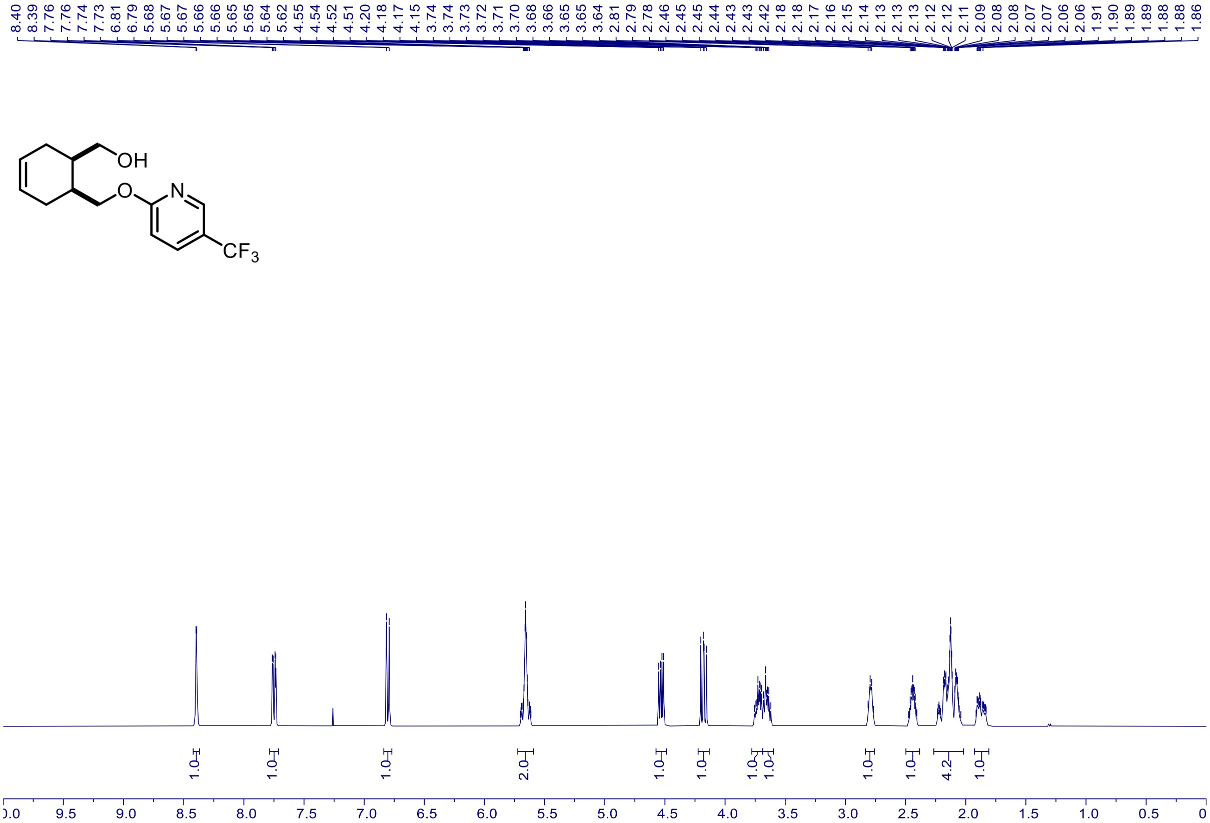

S25 – ^{13}C NMR (101 MHz, $CDCl_3$)

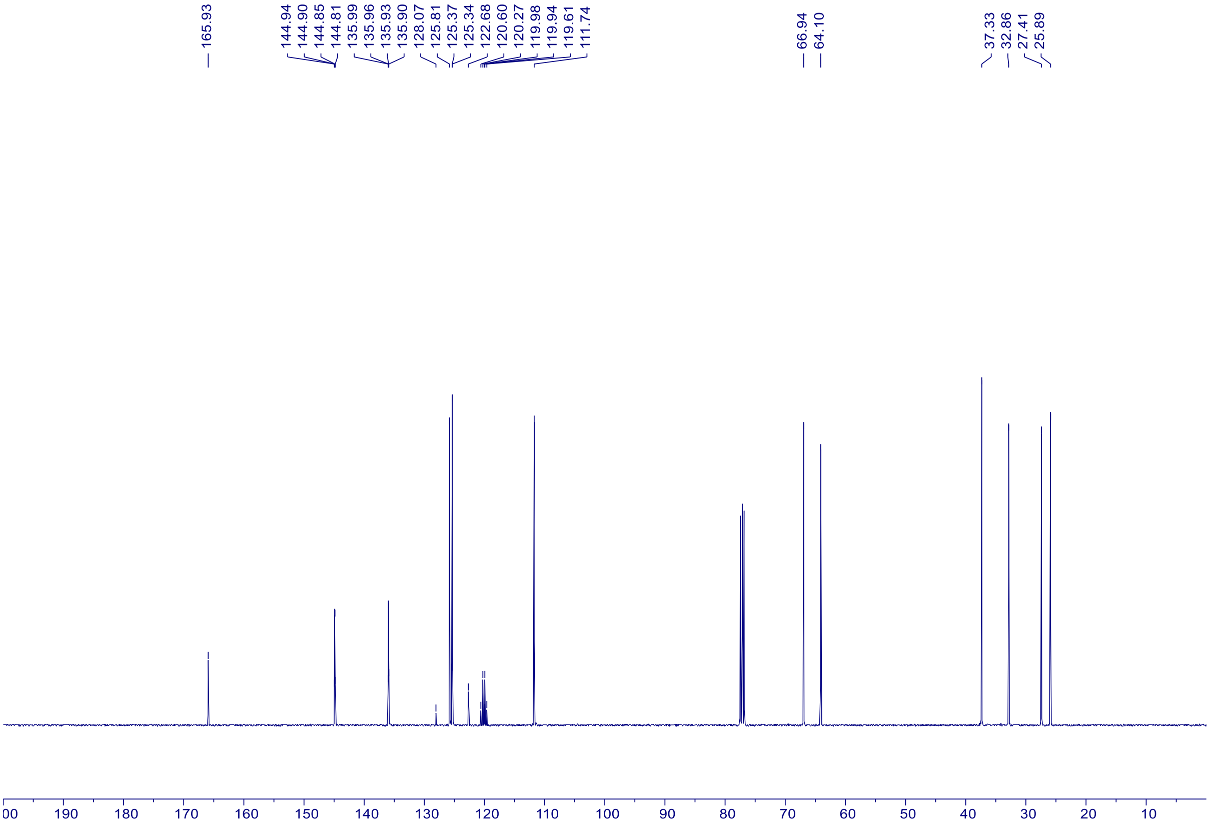

S25 – ^{19}F NMR (376 MHz, $CDCl_3$)

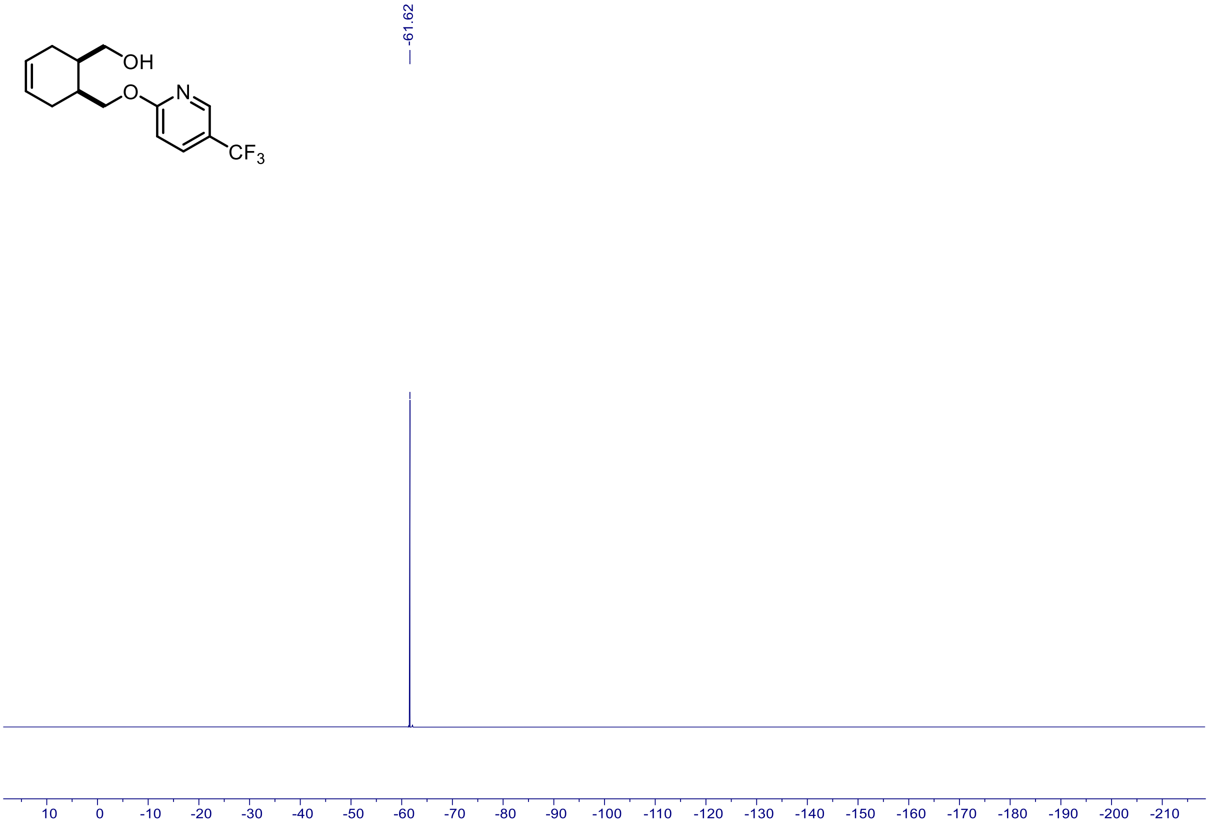

33s – ^{1}H NMR (400 MHz, CDCl$_3$)

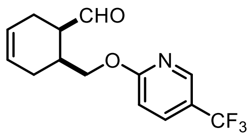

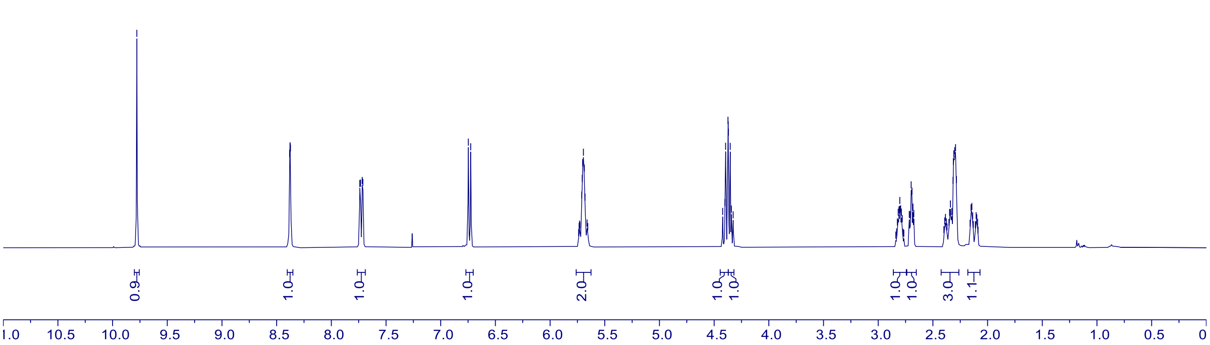

33s – ^{13}C NMR (101 MHz, CDCl$_3$)

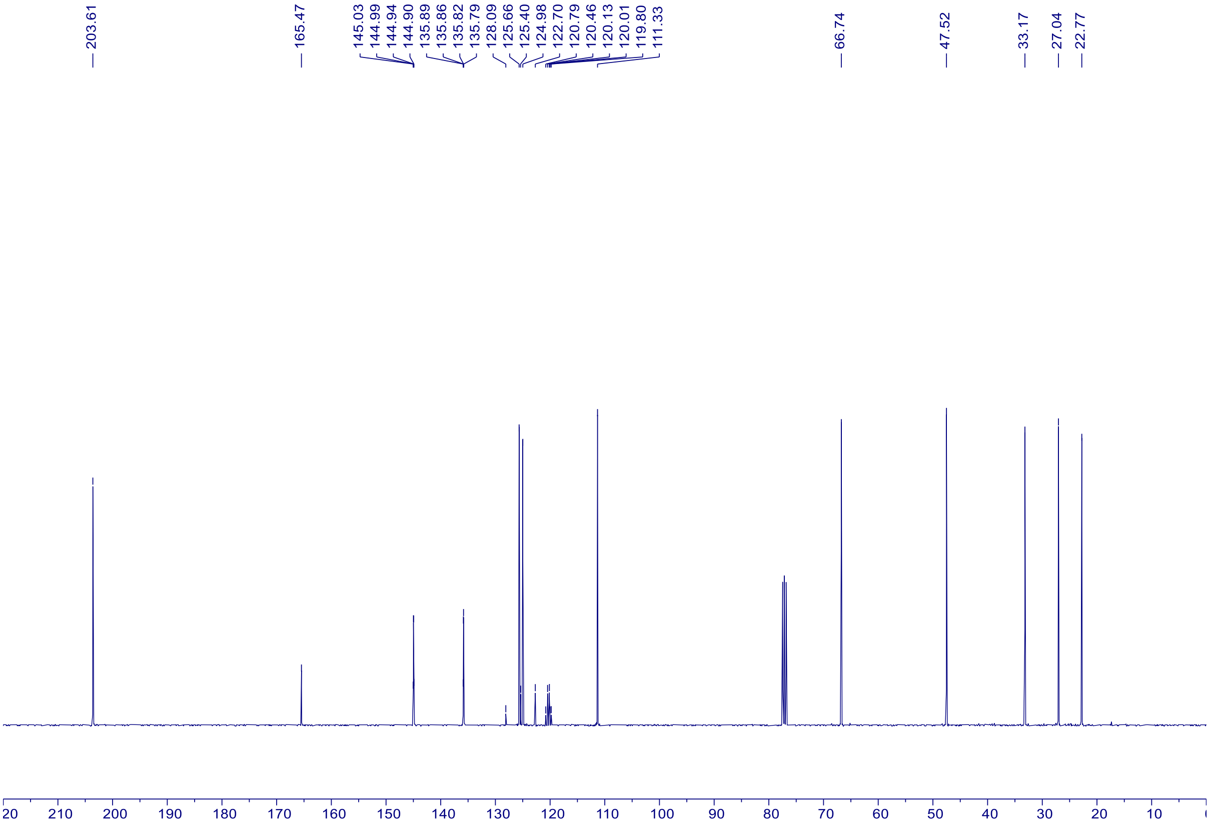

33s – ^{19}F NMR (376 MHz, $CDCl_3$)

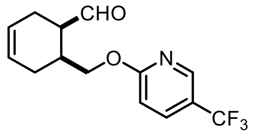

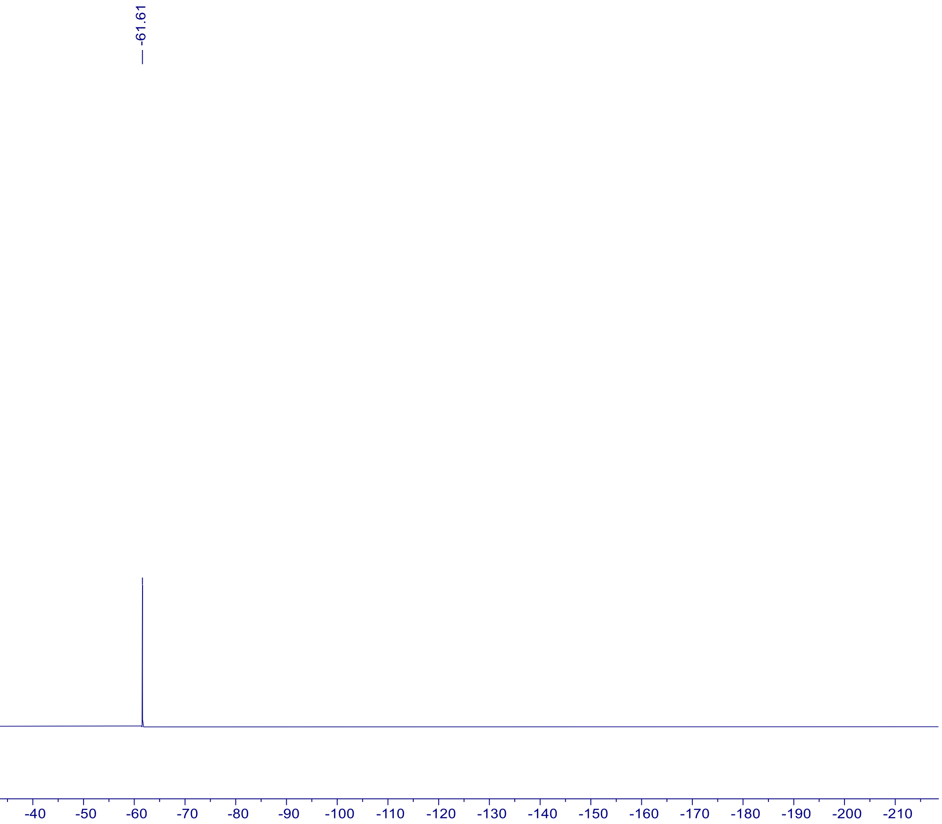

S26 – ^{1}H NMR (400 MHz, $CDCl_3$)

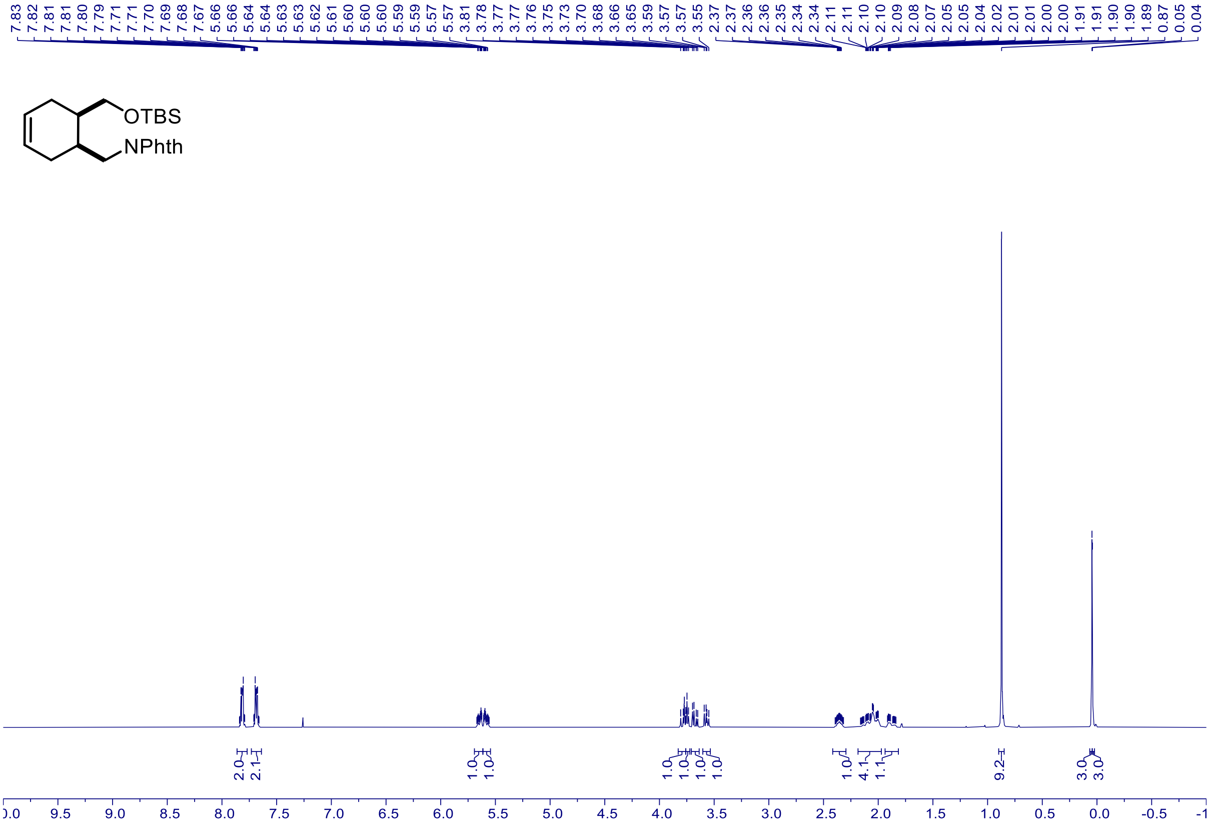

S26 – ^{13}C NMR (101 MHz, $CDCl_3$)

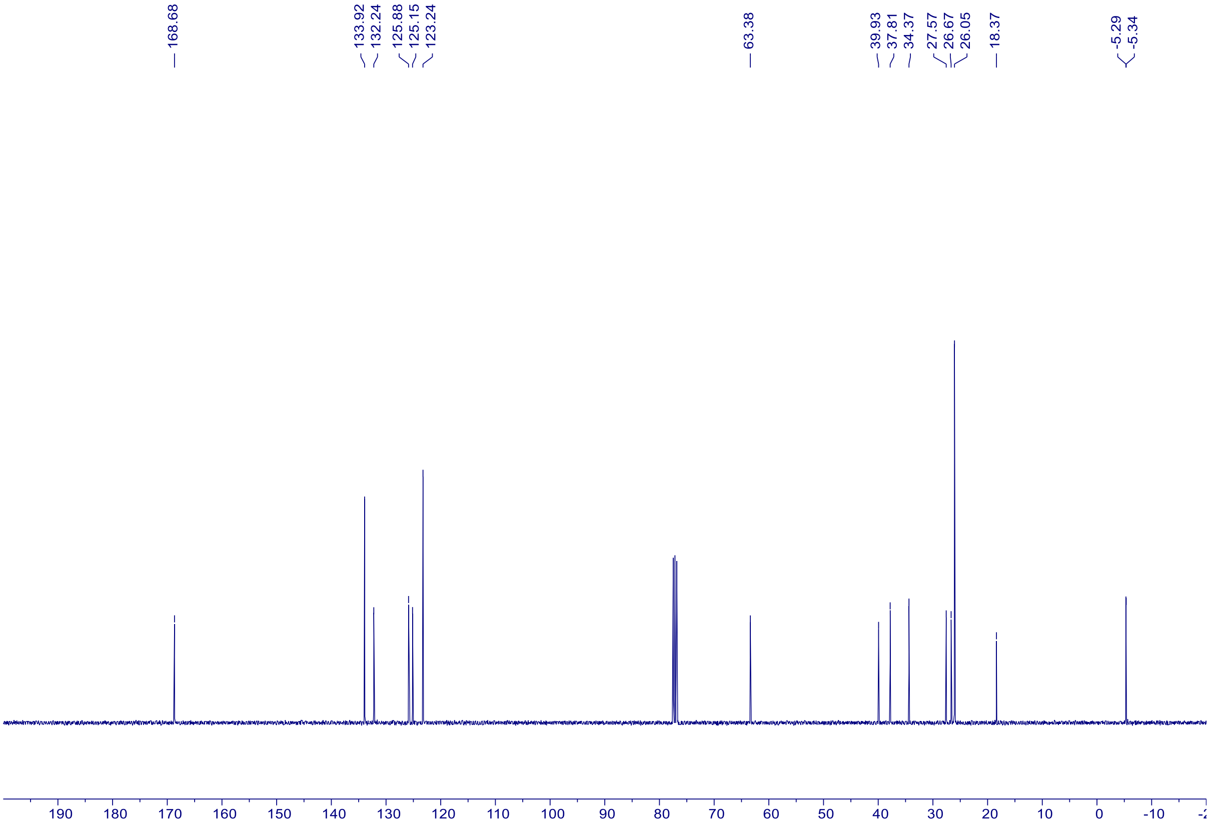

S27 – ^{1}H NMR (500 MHz, $CDCl_3$)

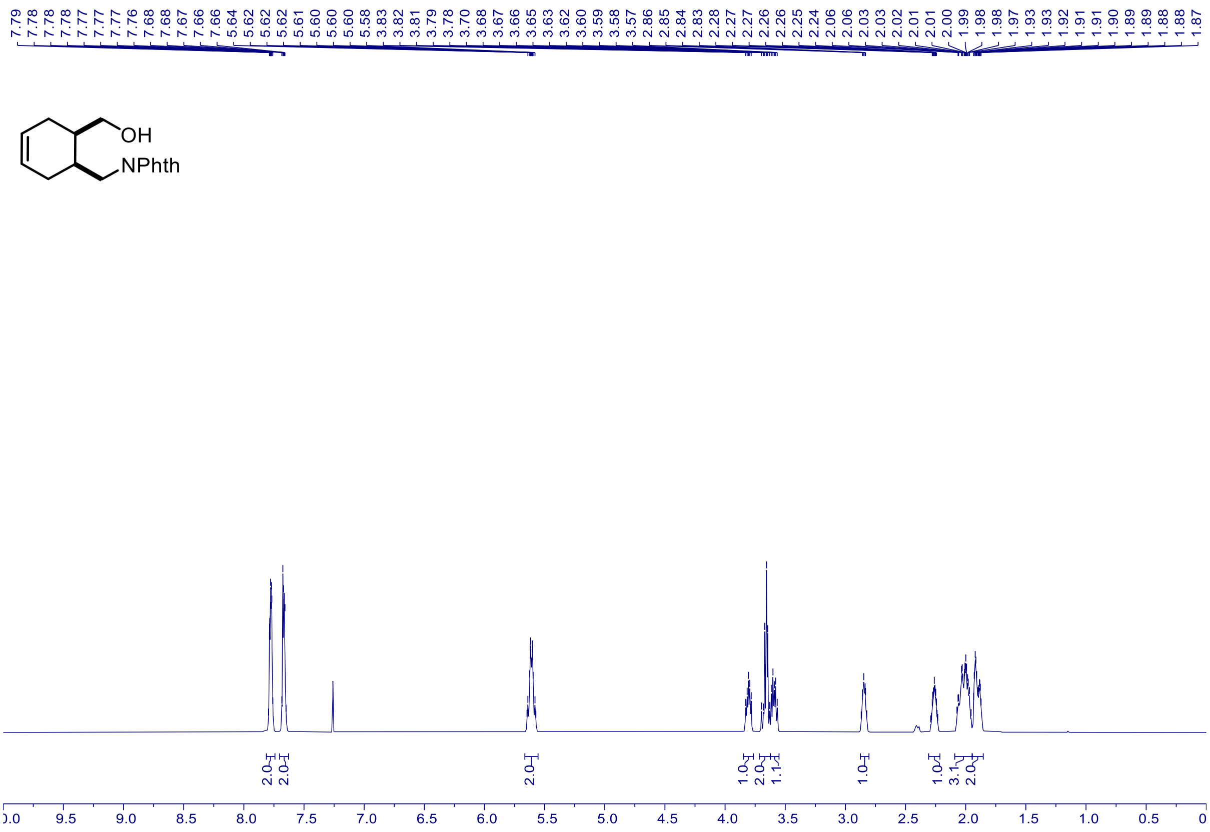

S27 – ^{13}C NMR (126 MHz, $CDCl_3$)

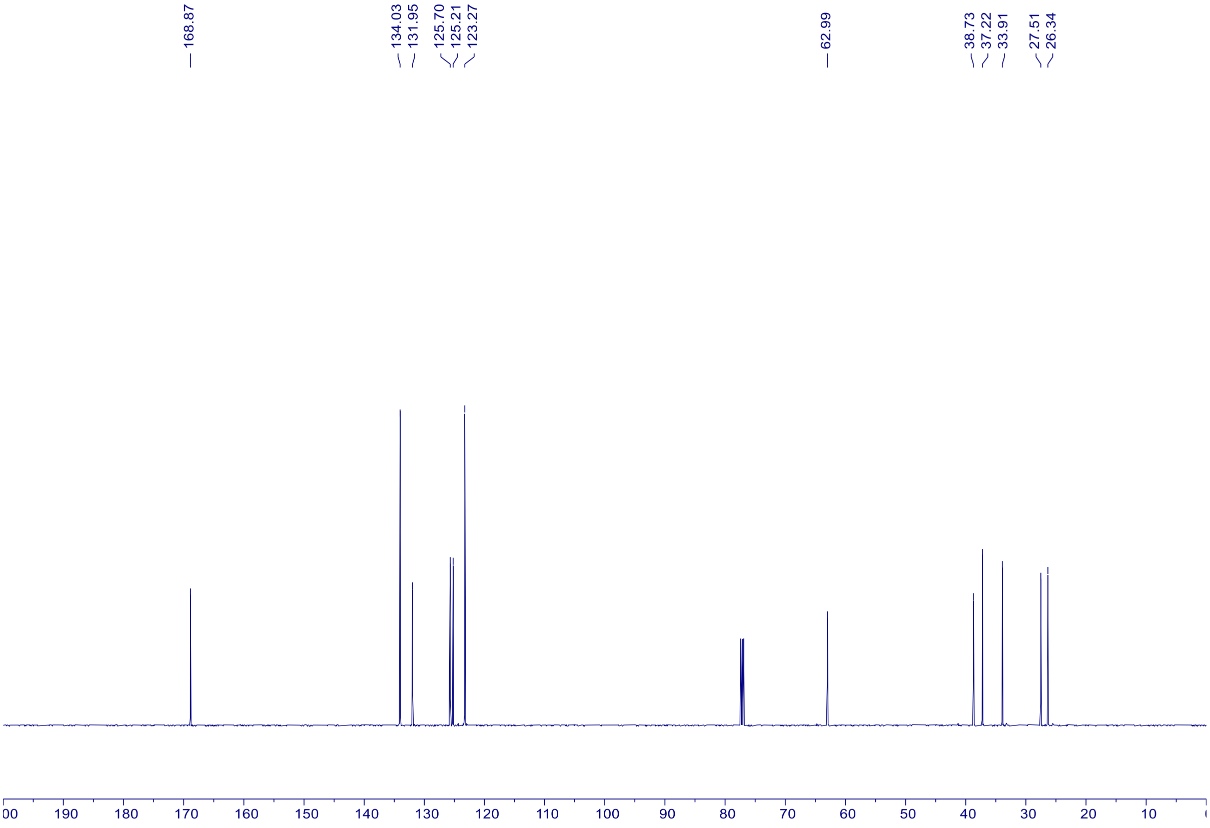

34s – ^{1}H NMR (400 MHz, $CDCl_3$)

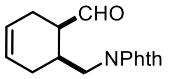

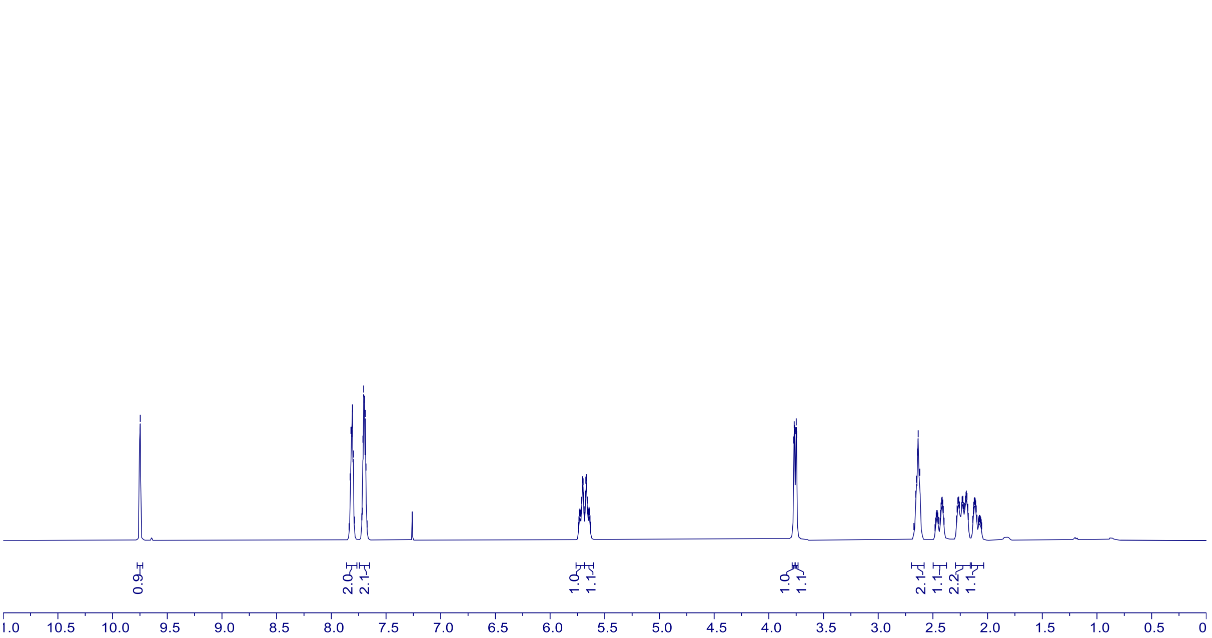

34s – ^{13}C NMR (101 MHz, $CDCl_3$)

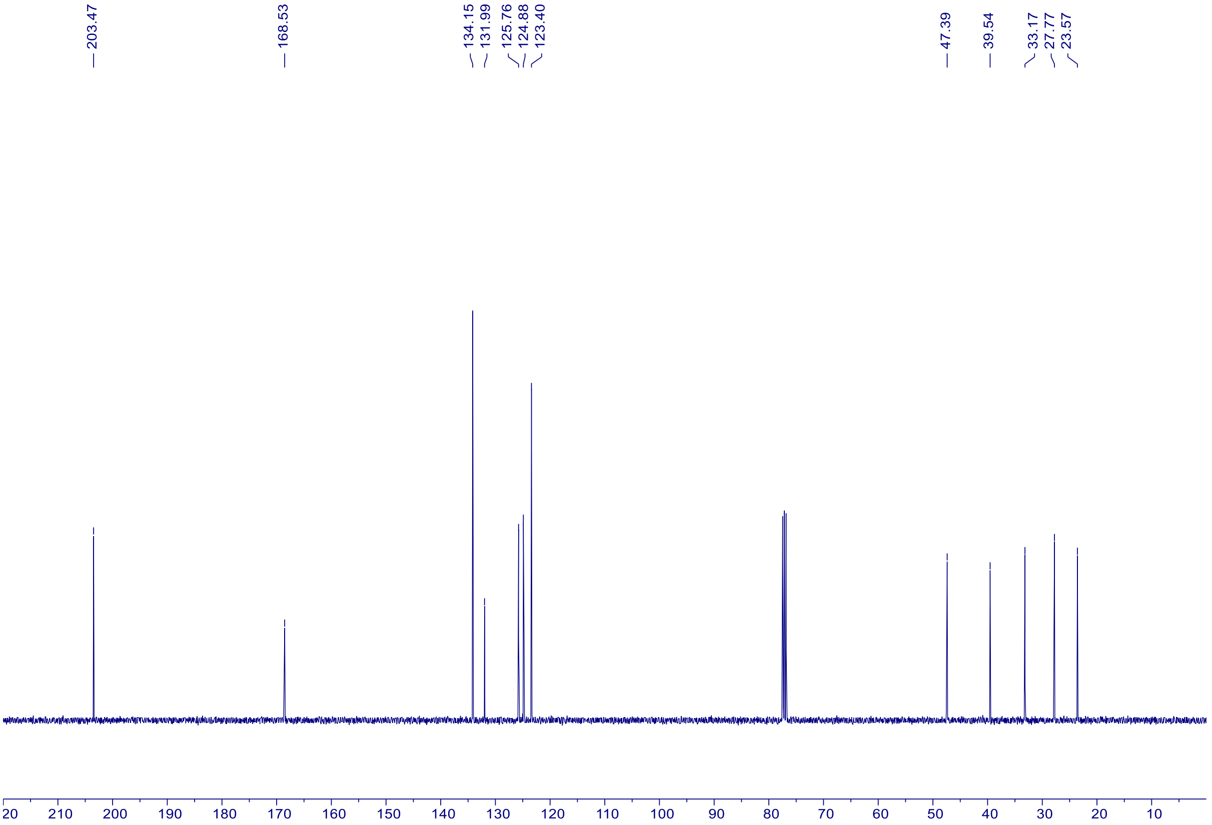

S28 – ^{1}H NMR (500 MHz, $CDCl_3$)

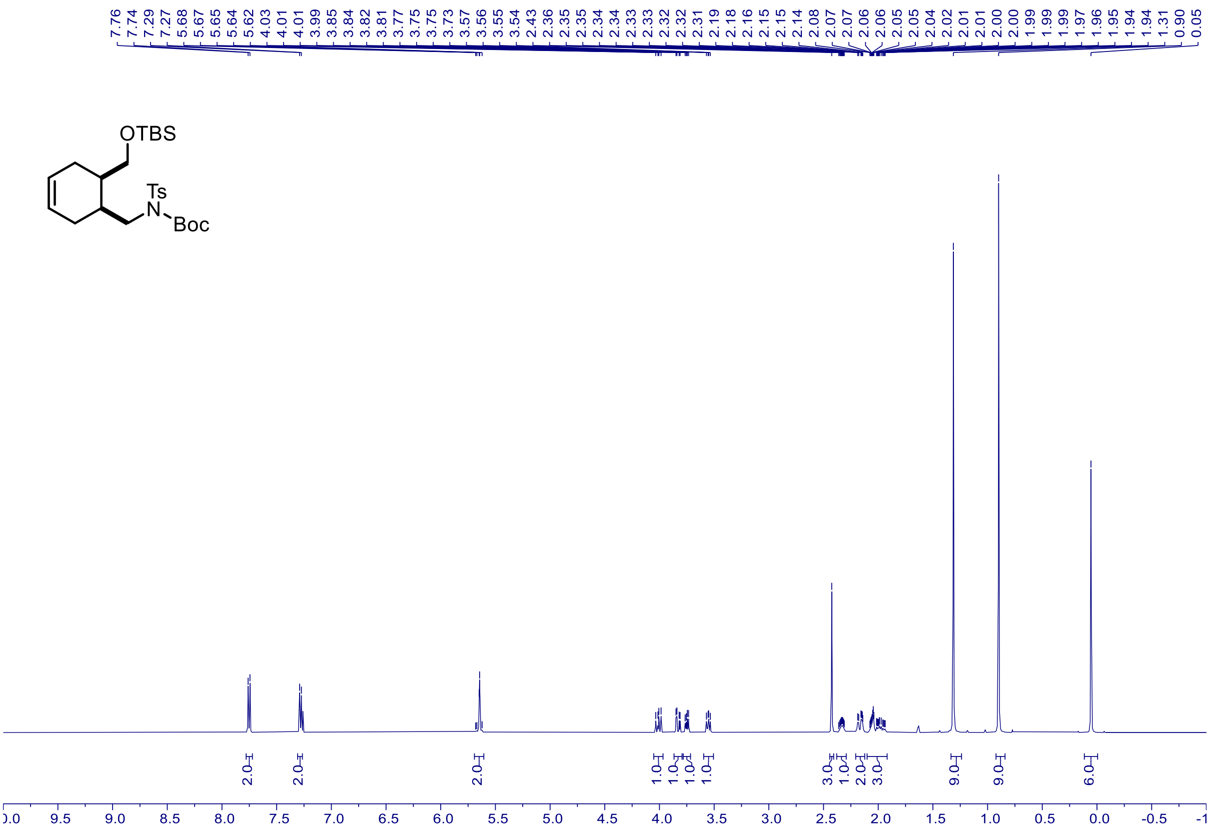

S28 – ^{13}C NMR (126 MHz, $CDCl_3$)

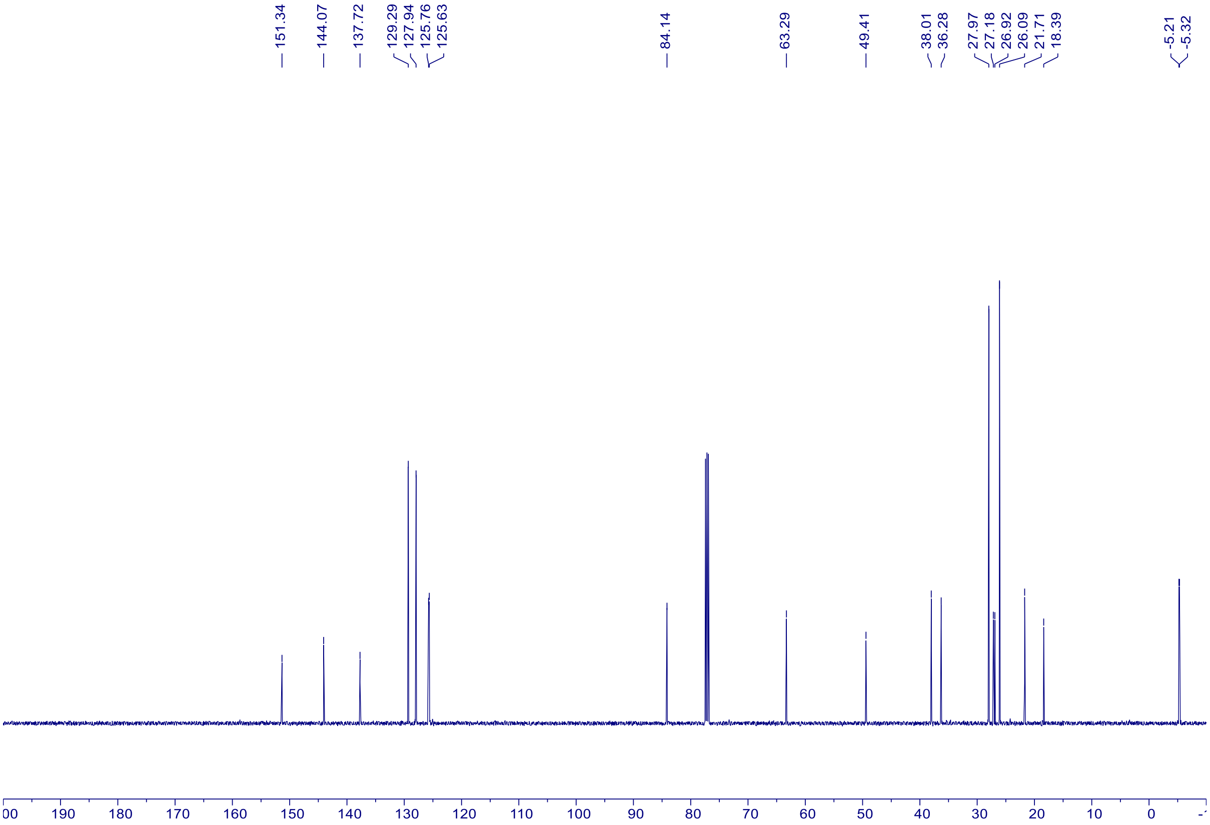

S29 – ^{1}H NMR (400 MHz, CDCl$_3$)

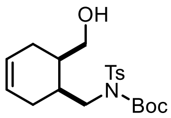

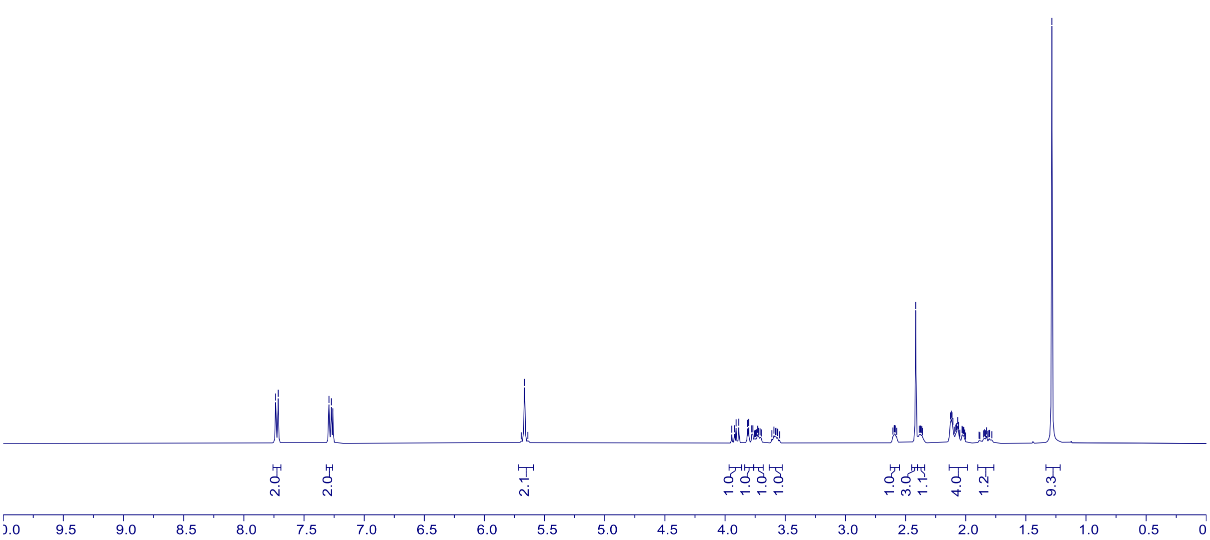

S29 – ^{13}C NMR (101 MHz, CDCl$_3$)

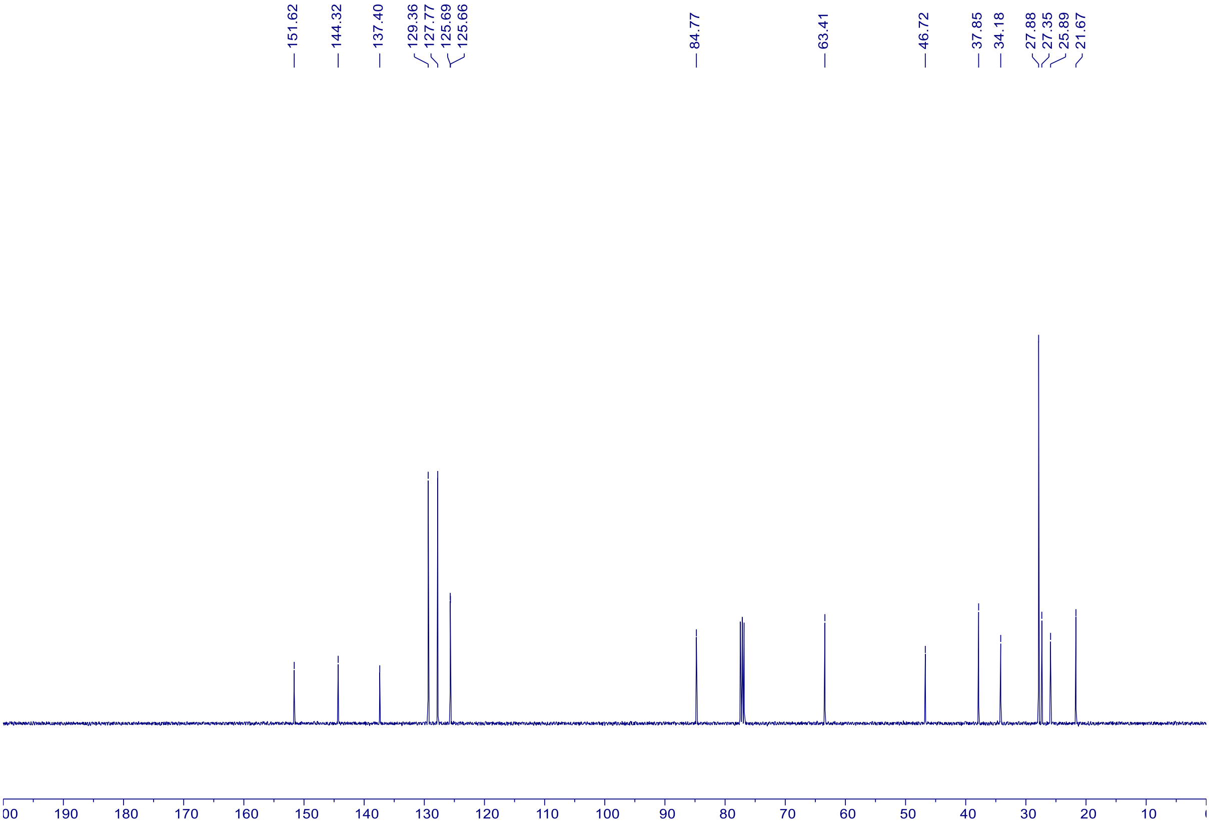

35s – ^{1}H NMR (400 MHz, $CDCl_3$)

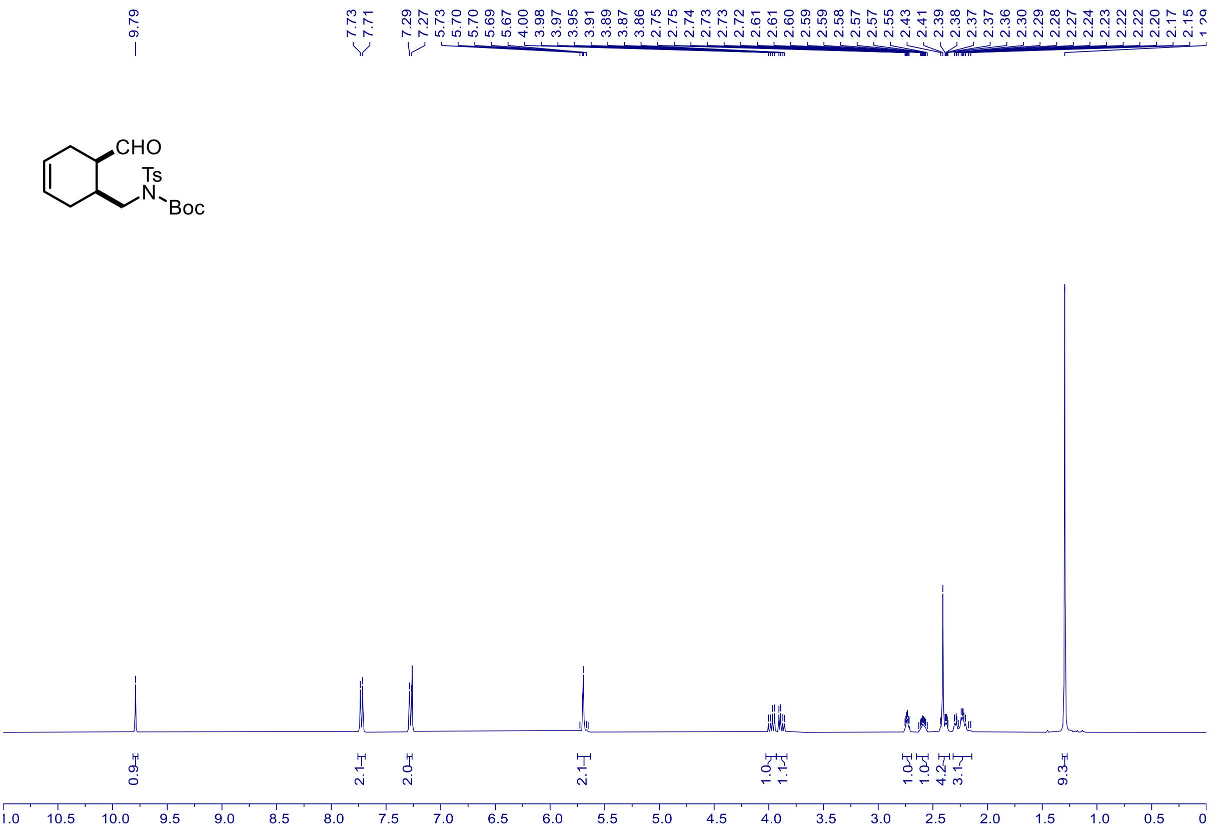

35s – ^{13}C NMR (101 MHz, $CDCl_3$)

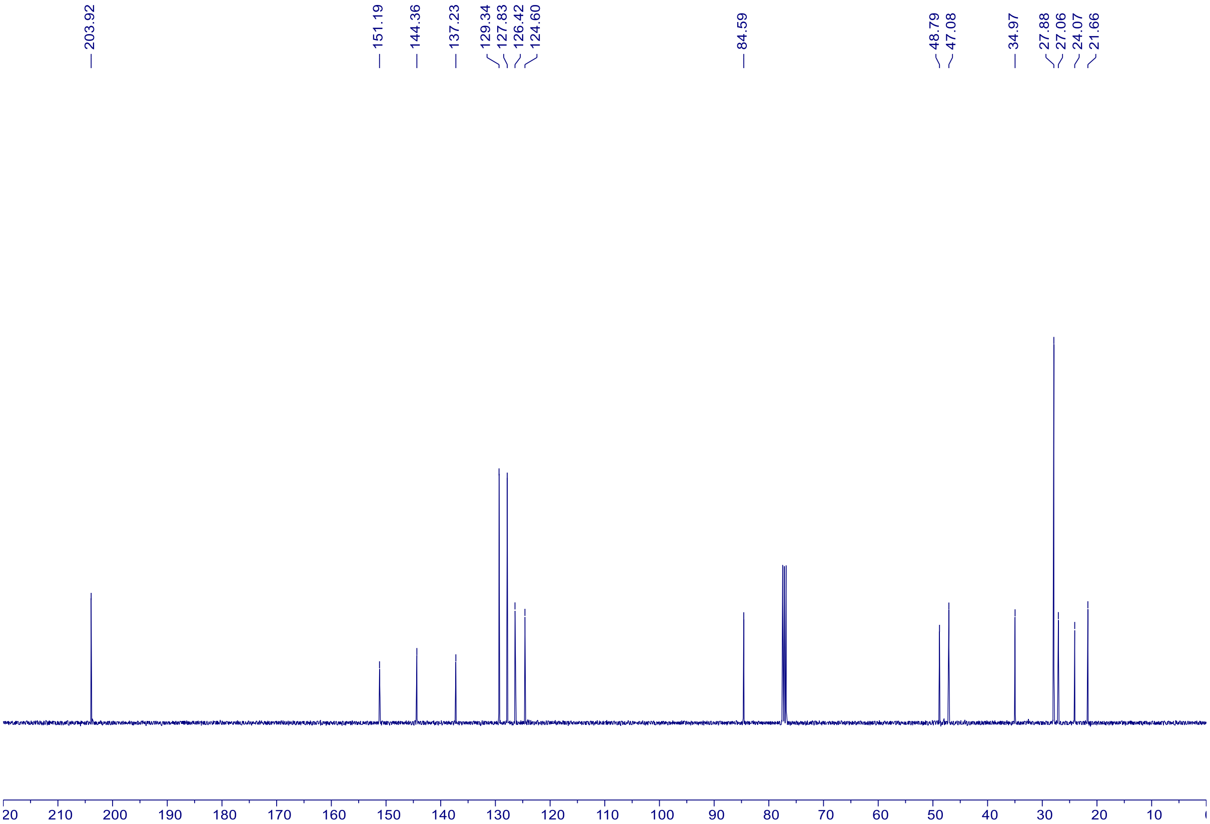

S30 – ^{1}H NMR (500 MHz, $CDCl_3$)

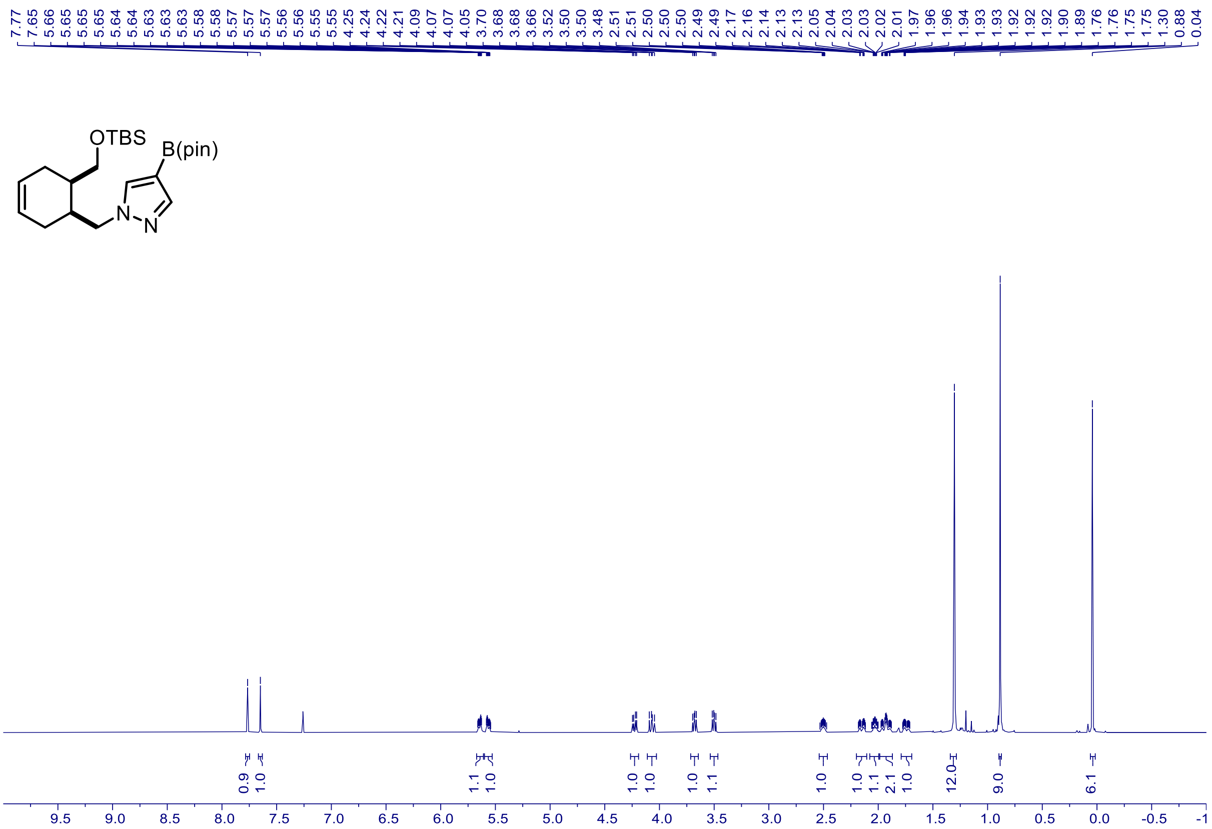

S30 – ^{13}C NMR (126 MHz, $CDCl_3$)

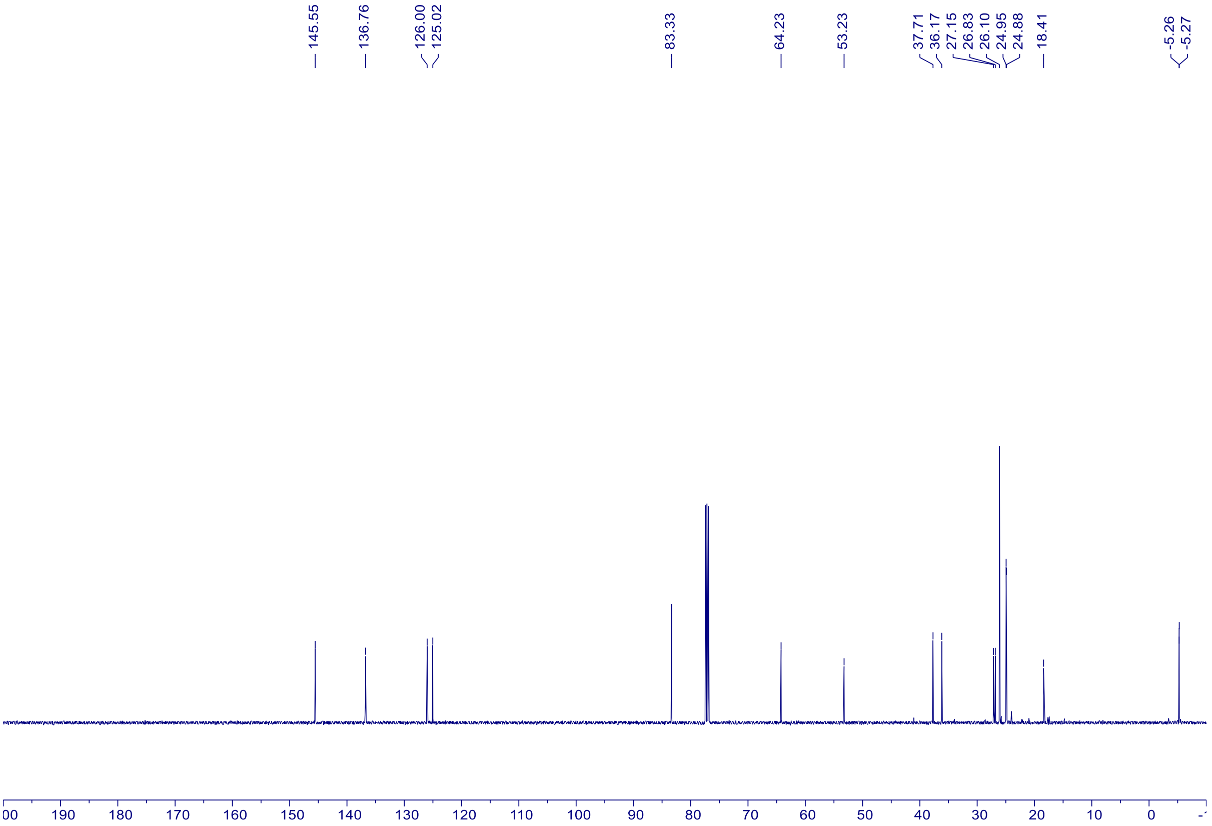

S30 – ^{11}B NMR (128 MHz, $CDCl_3$)

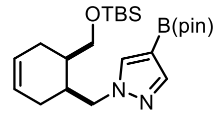

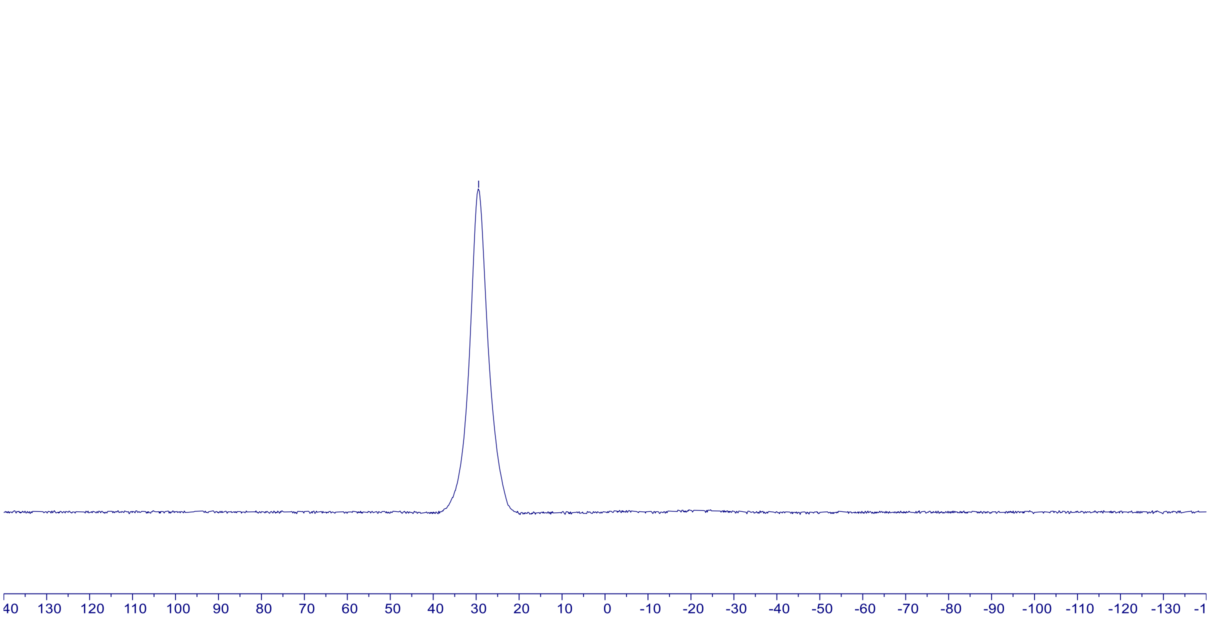

S31 – ^{1}H NMR (400 MHz, $CDCl_3$)

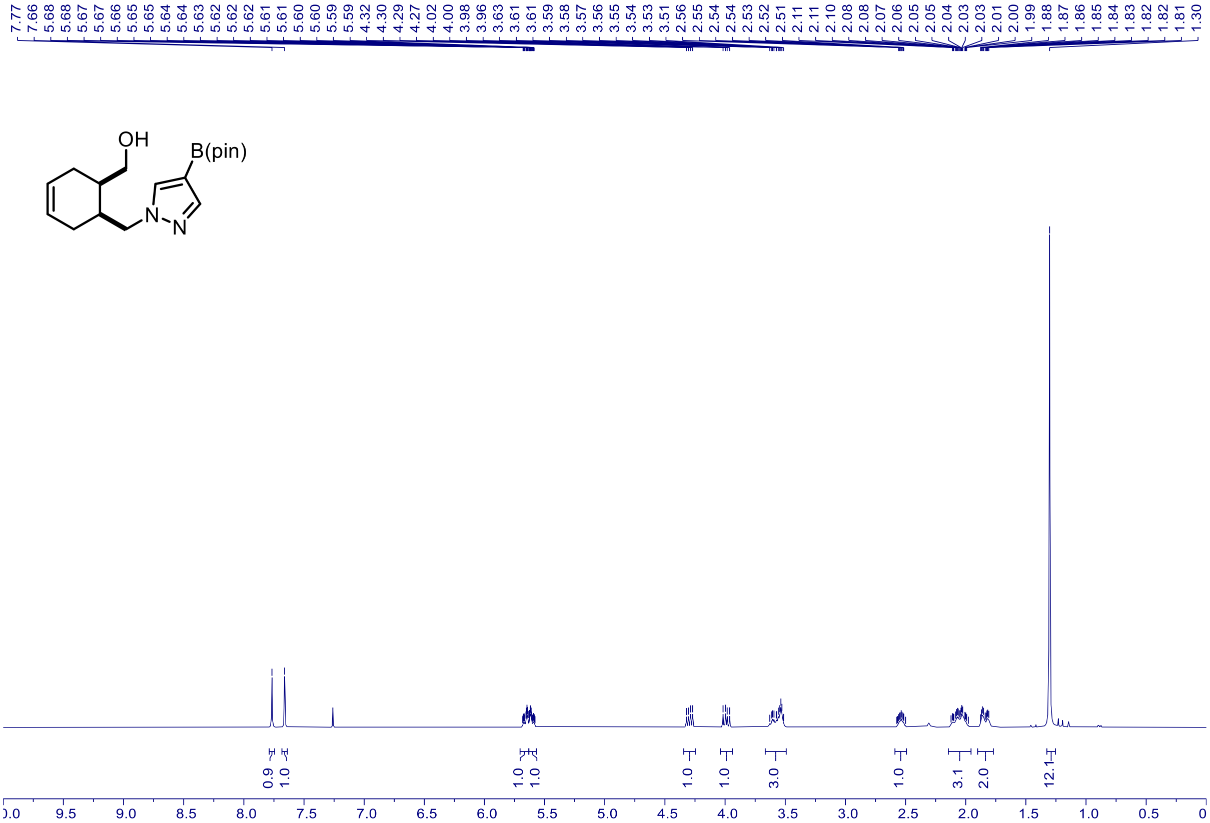

S31 – ^{13}C NMR (101 MHz, $CDCl_3$)

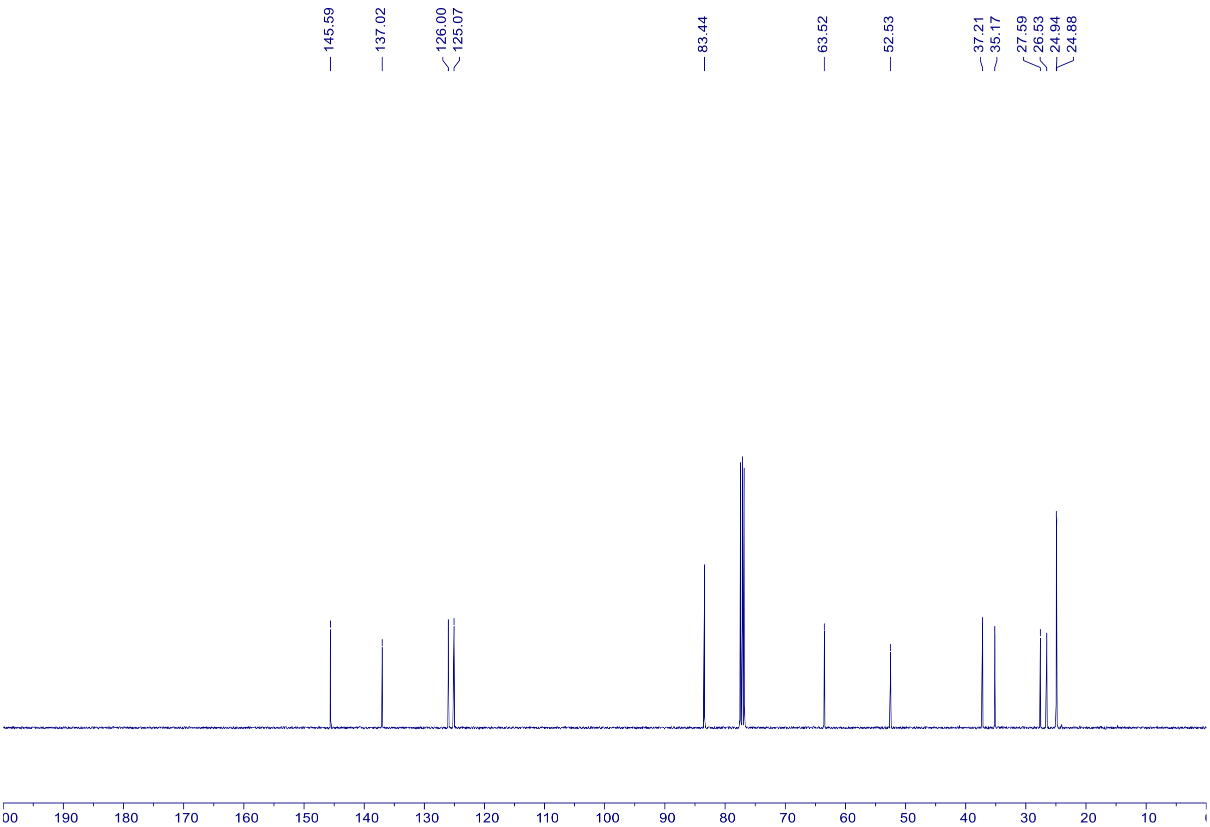

S31 – ^{11}B NMR (128 MHz, $CDCl_3$)

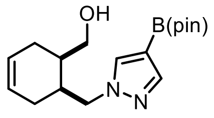

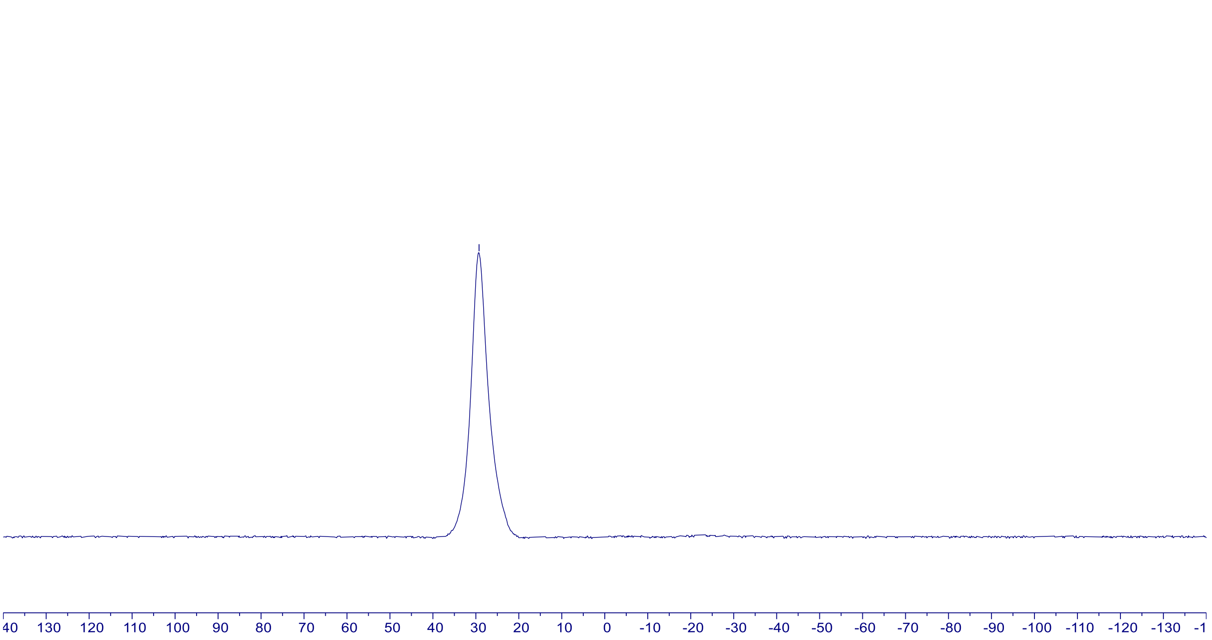

36s – ^{1}H NMR (400 MHz, $CDCl_3$)

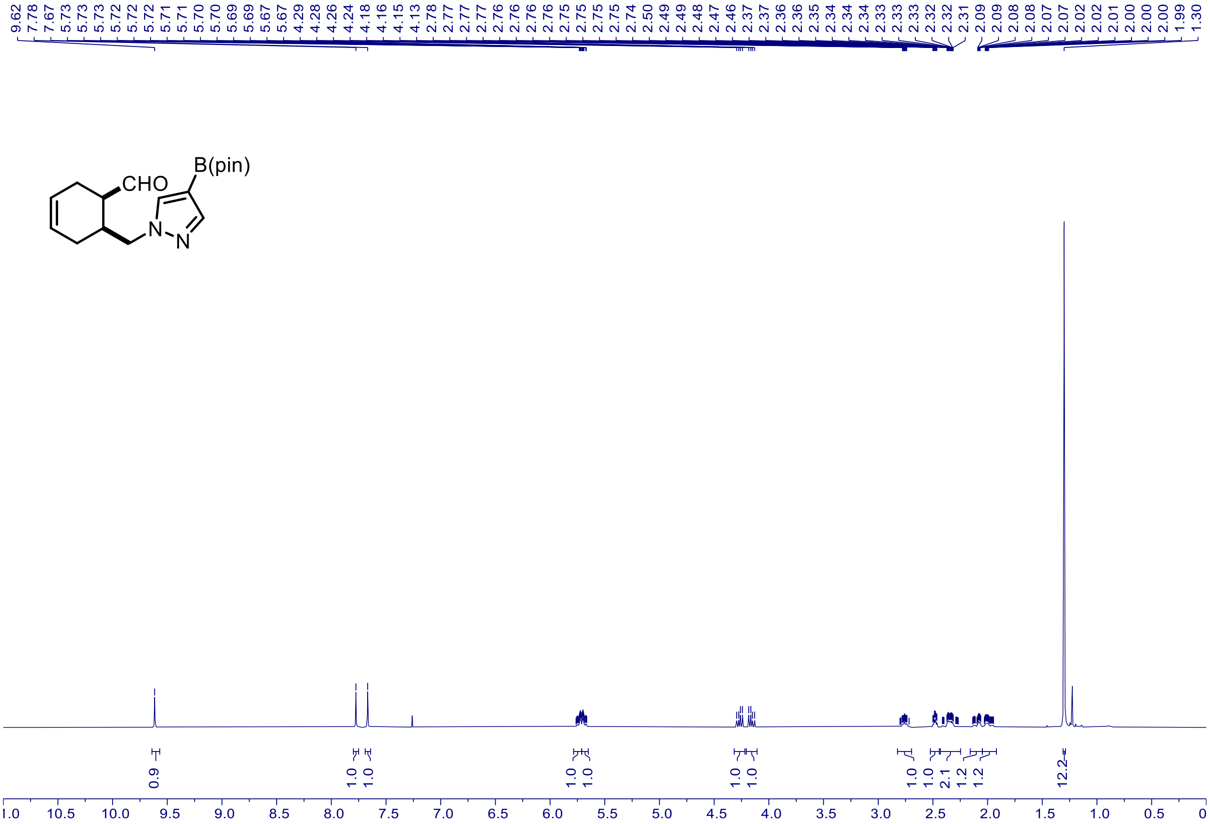

36s – ^{13}C NMR (101 MHz, $CDCl_3$)

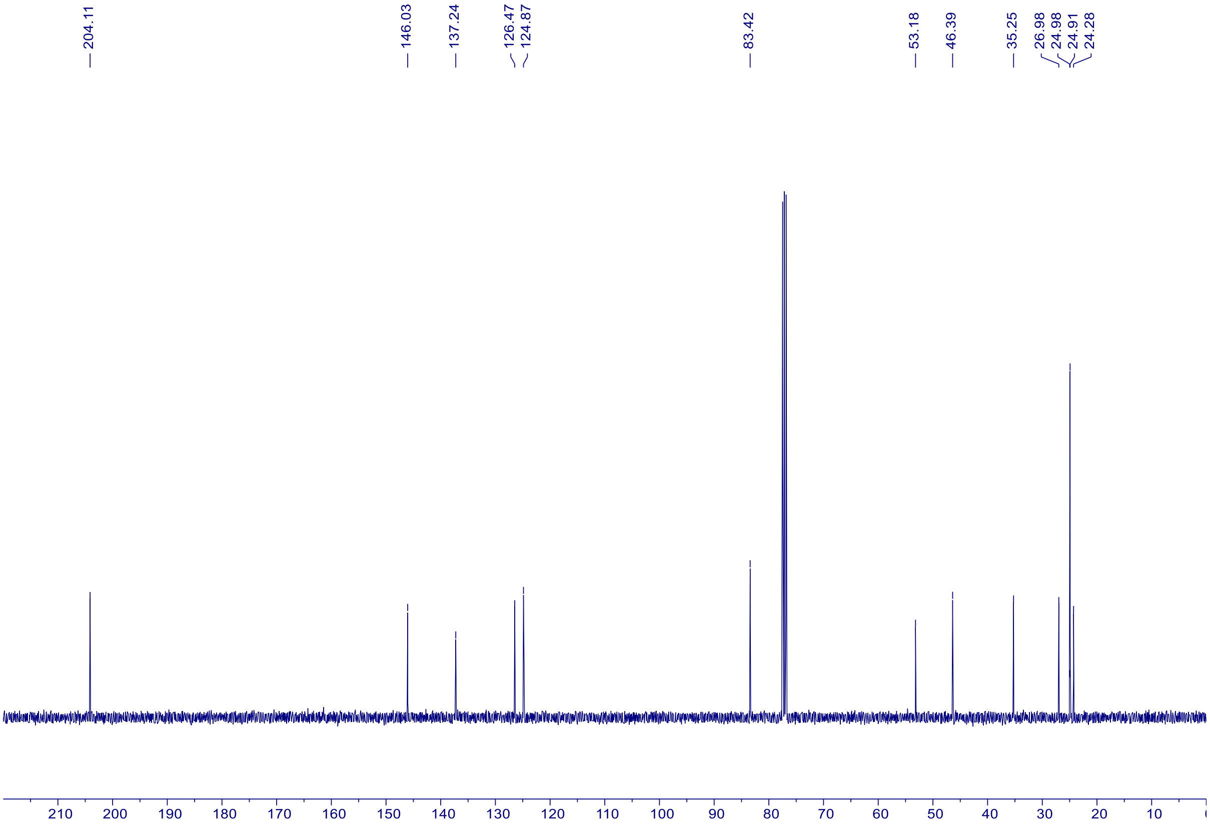

36s – ^{11}B NMR (128 MHz, $CDCl_3$)

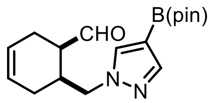

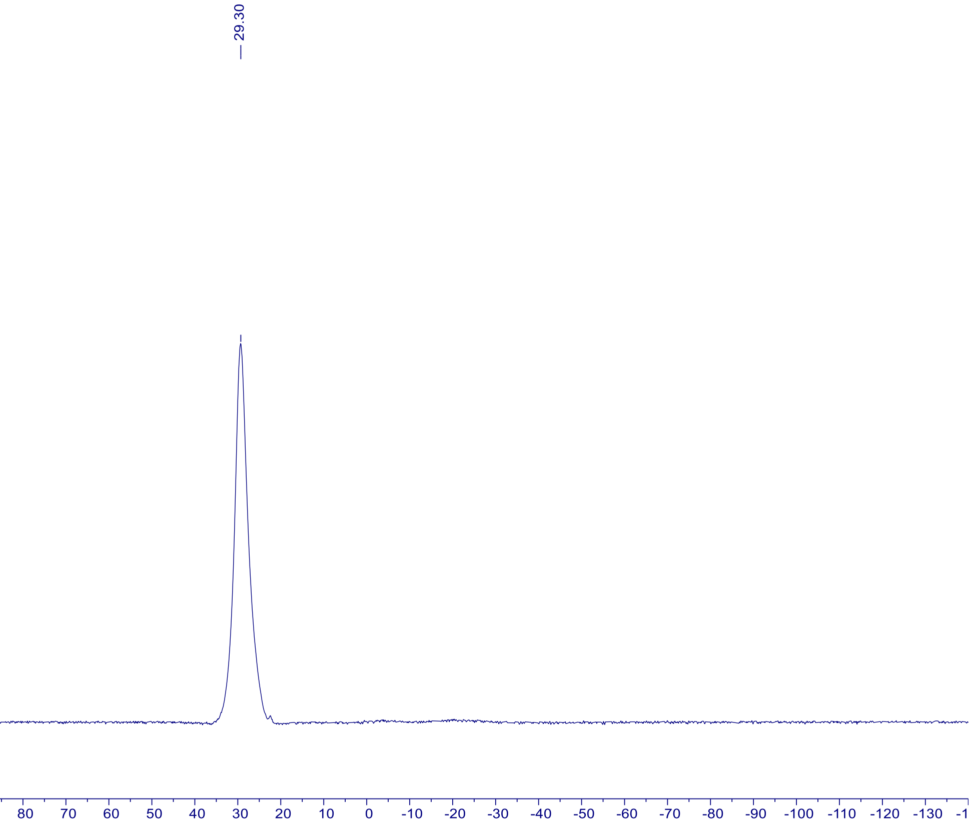

S32 – ^{1}H NMR (400 MHz, $CDCl_3$)

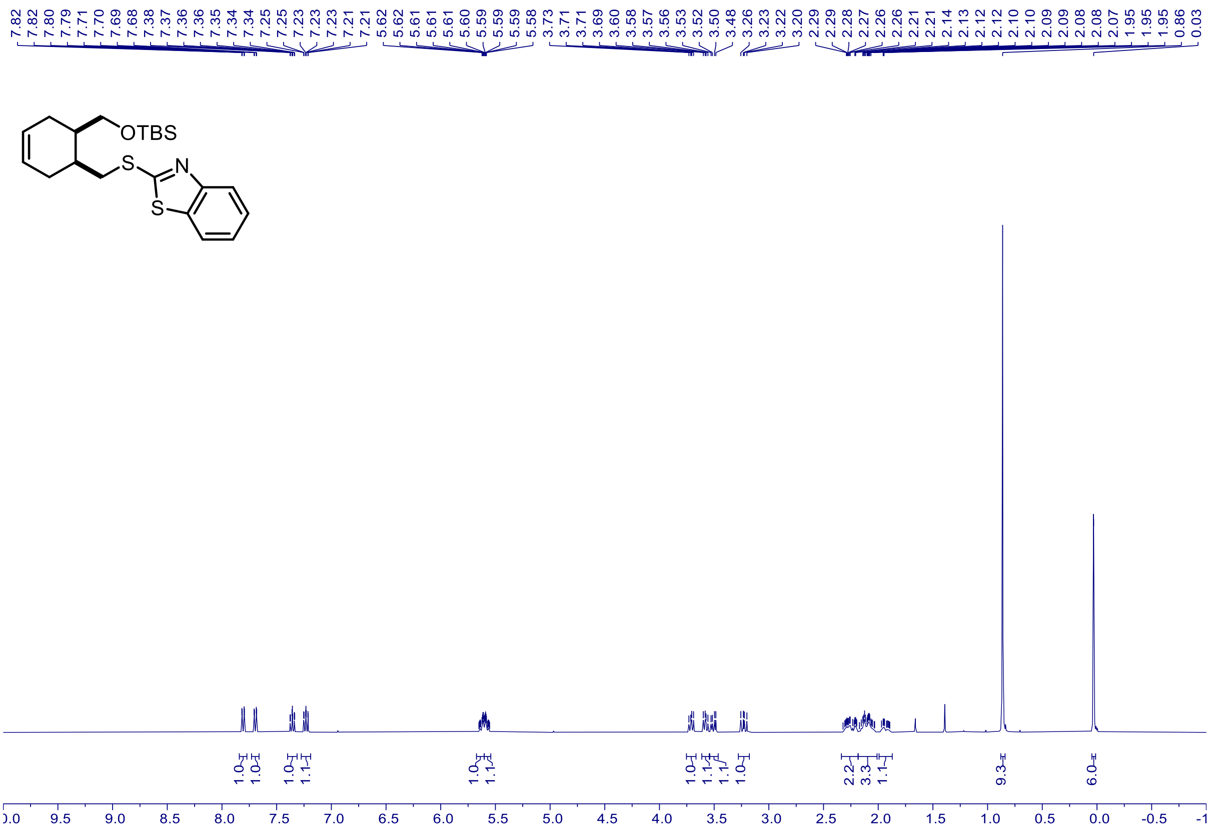

S32 – ^{13}C NMR (101 MHz, $CDCl_3$)

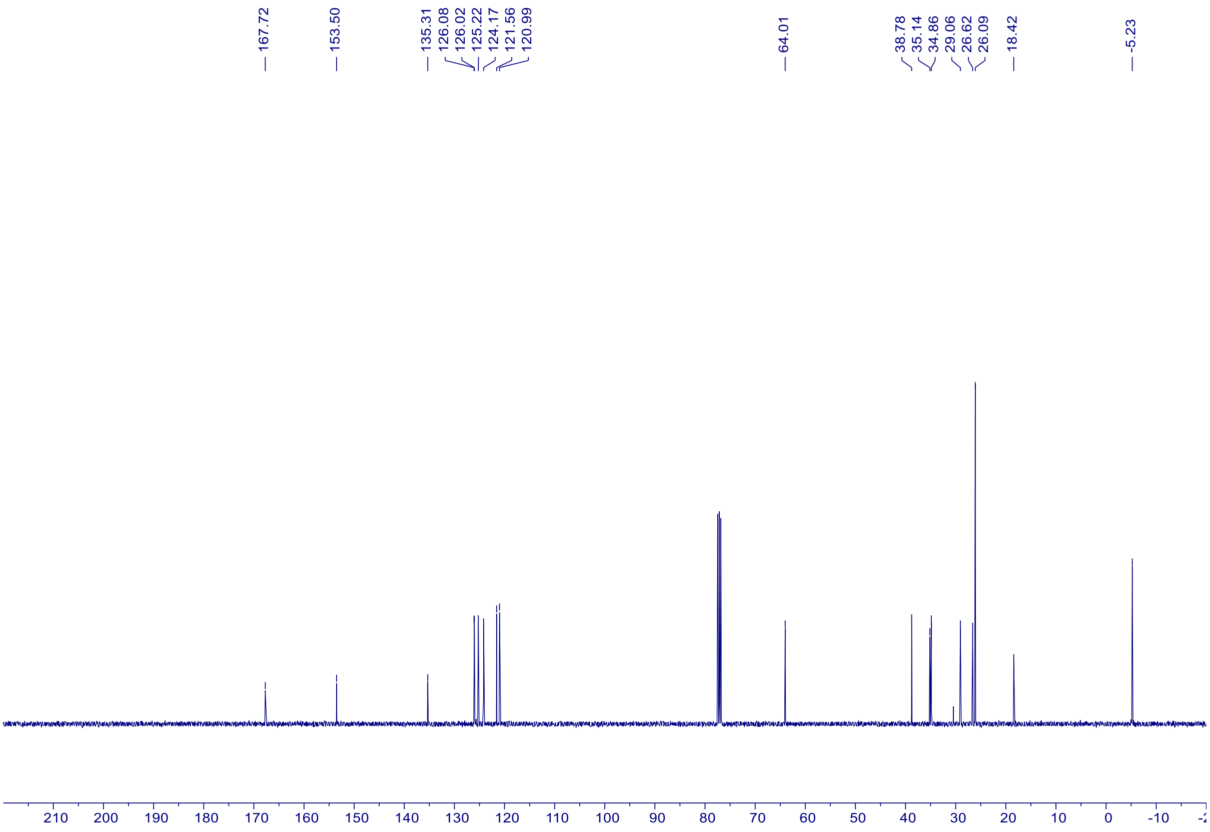

S33 – ^{1}H NMR (400 MHz, $CDCl_3$)

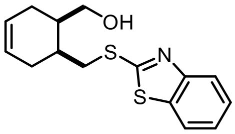

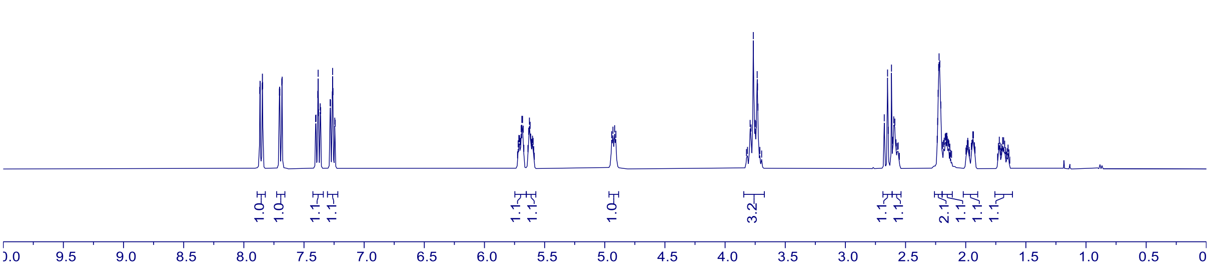

S33 – ^{13}C NMR (101 MHz, $CDCl_3$)

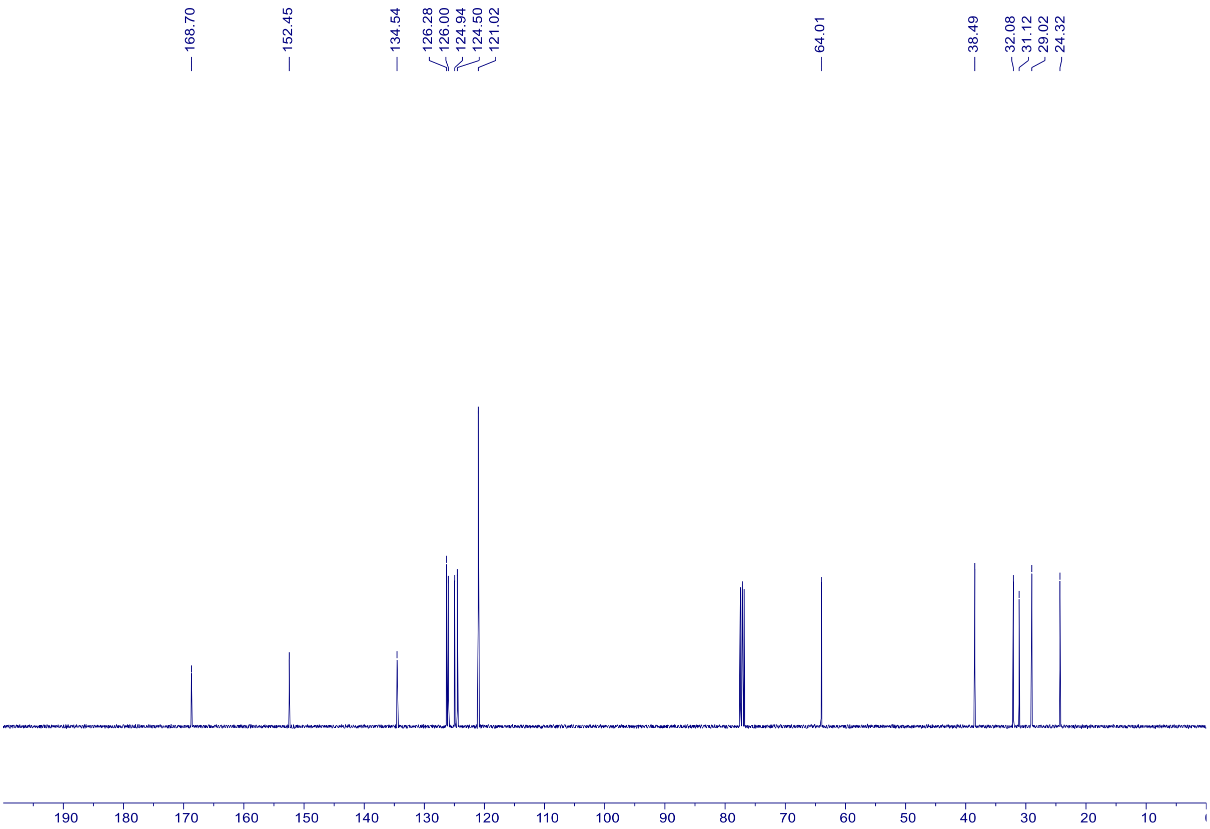

37s – ^{1}H NMR (500 MHz, CDCl$_3$)

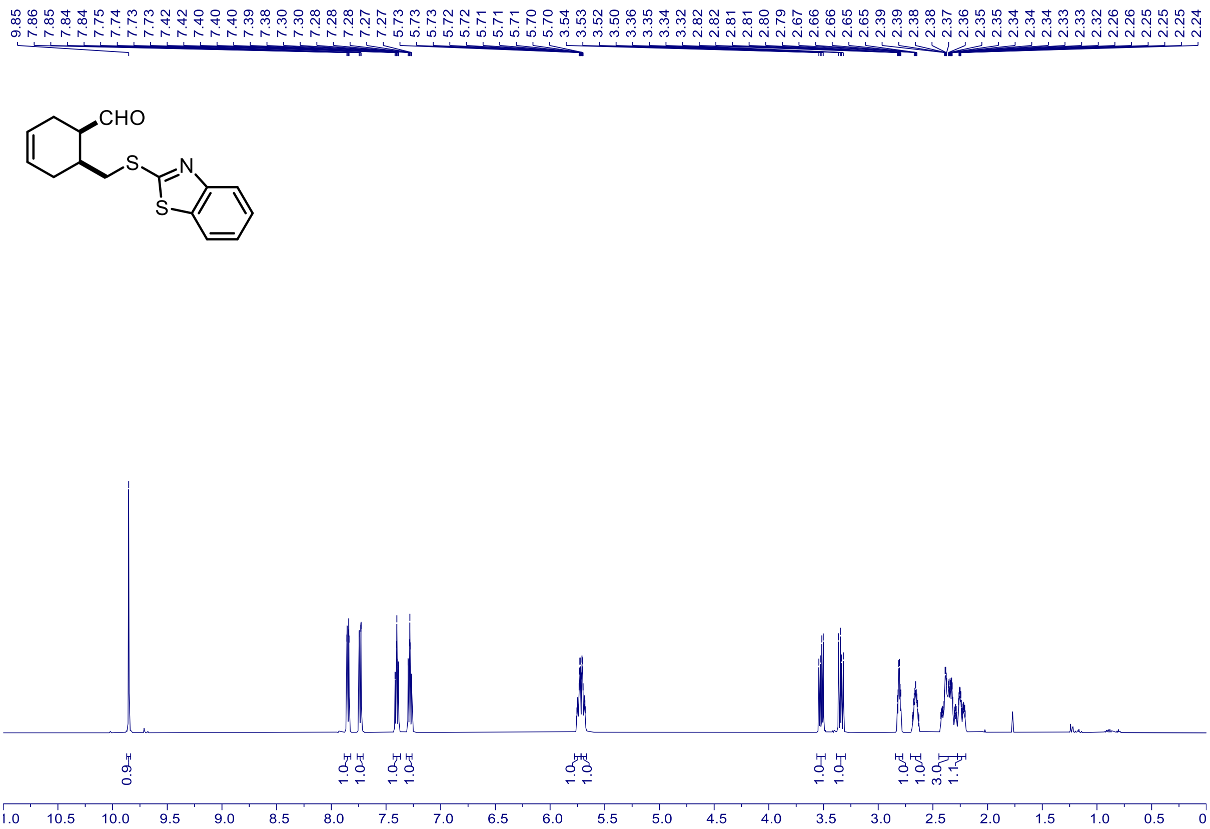

37s – ^{13}C NMR (126 MHz, CDCl$_3$)

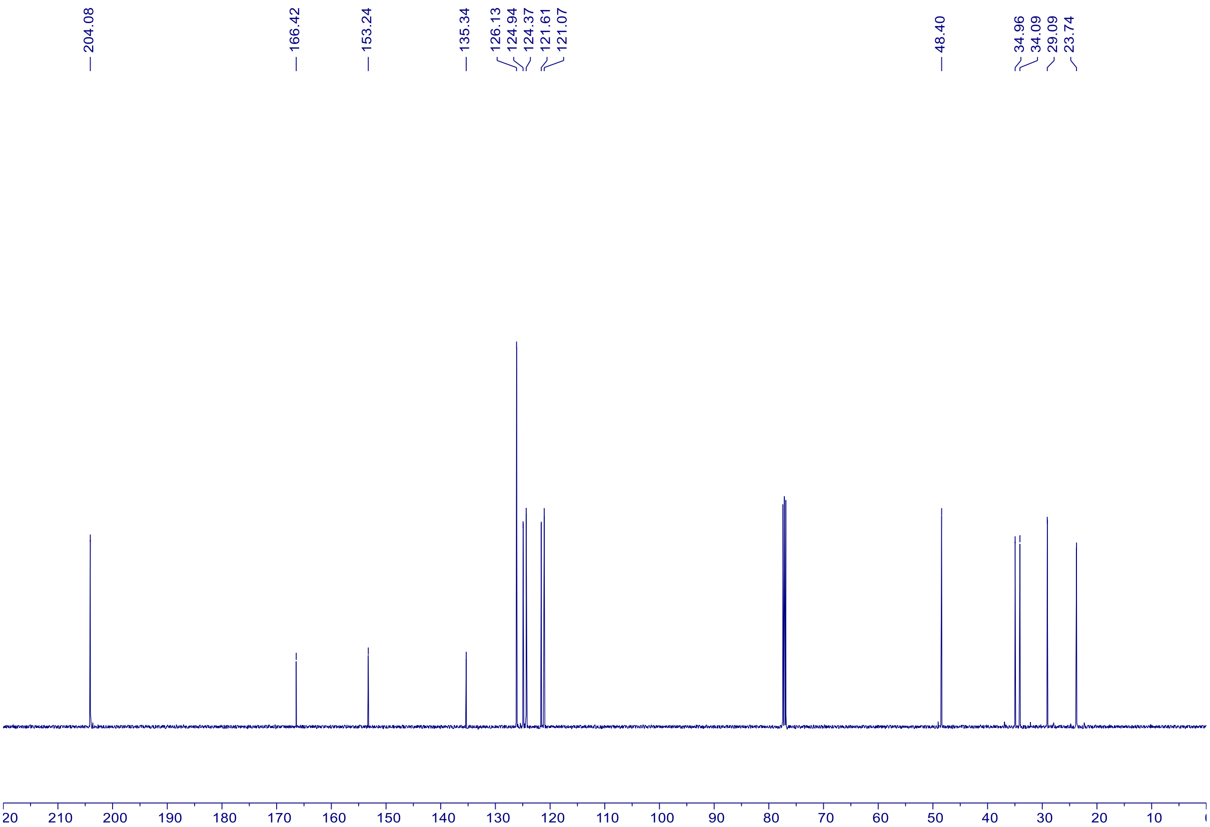

39 – ¹H NMR (400 MHz, $CDCl_3$)

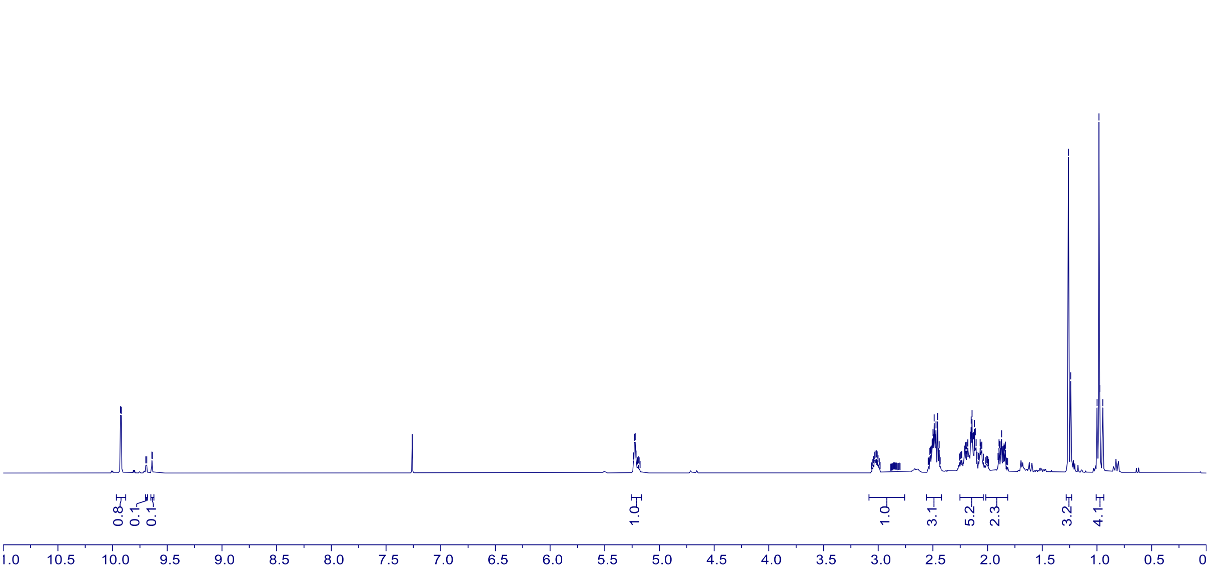

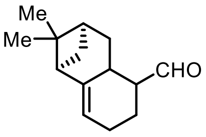

39 – ¹³C NMR (101 MHz, $CDCl_3$)

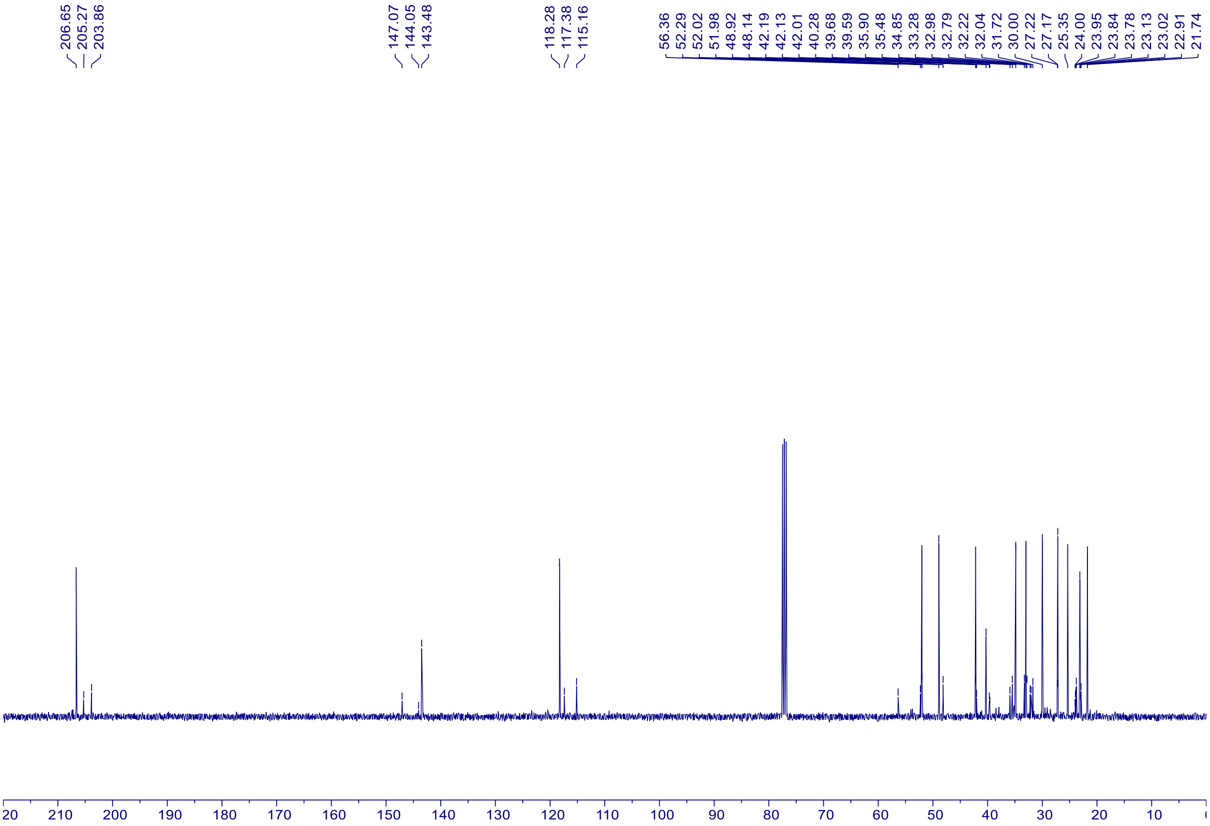

S35 – ^{1}H NMR (400 MHz, $CDCl_3$)

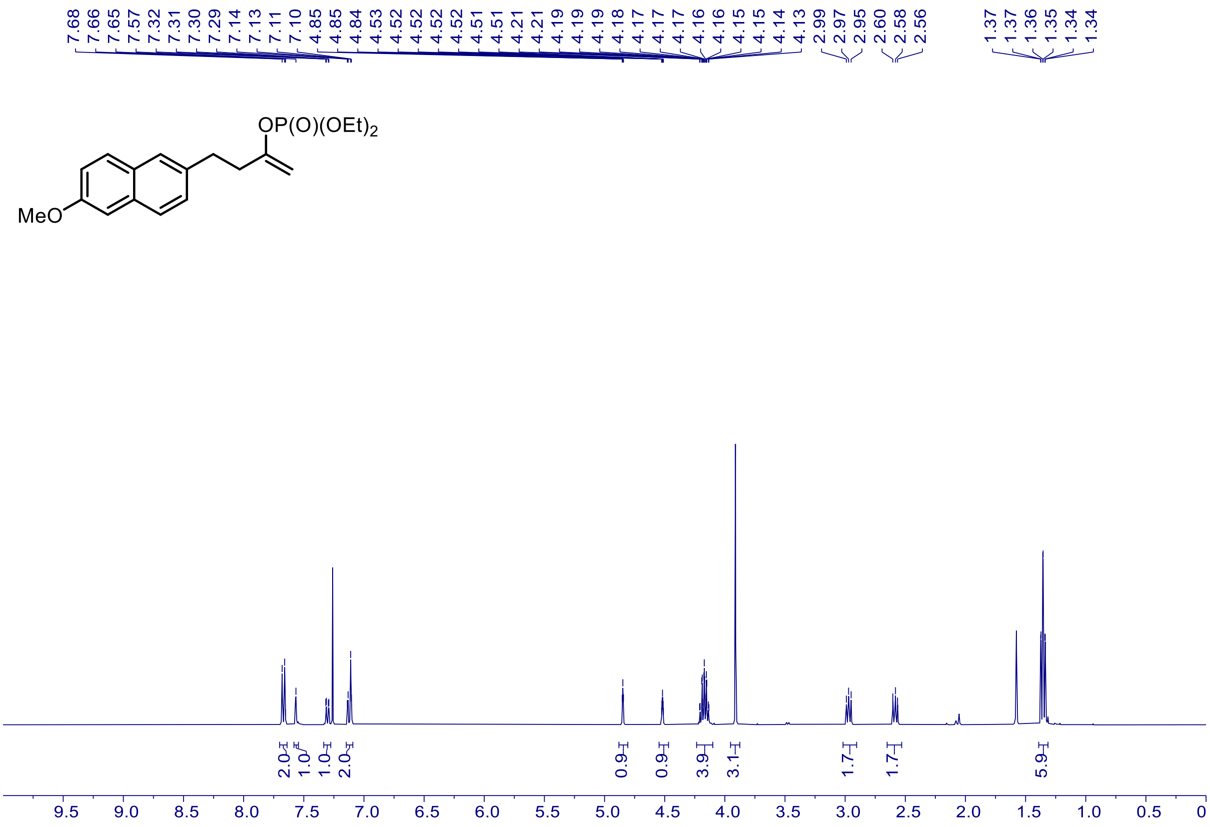

S35 – ^{13}C NMR (101 MHz, $CDCl_3$)

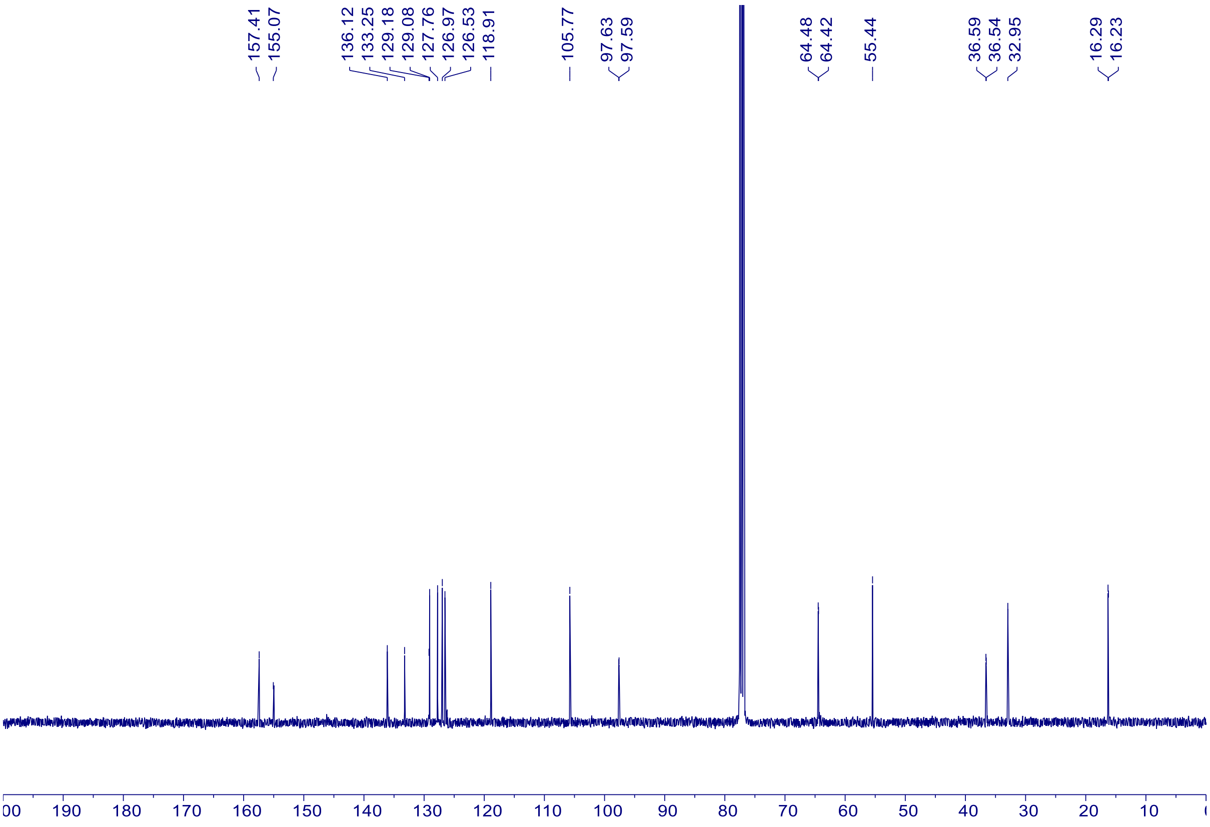

S35 – ^{31}P NMR (162 MHz, $CDCl_3$)

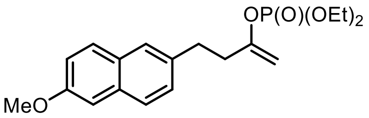

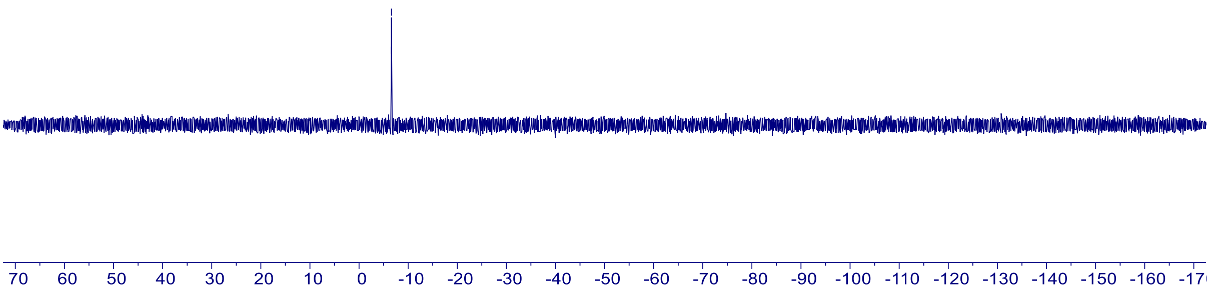

S36 – ^{1}H NMR (500 MHz, $CDCl_3$)

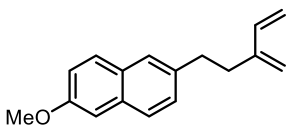

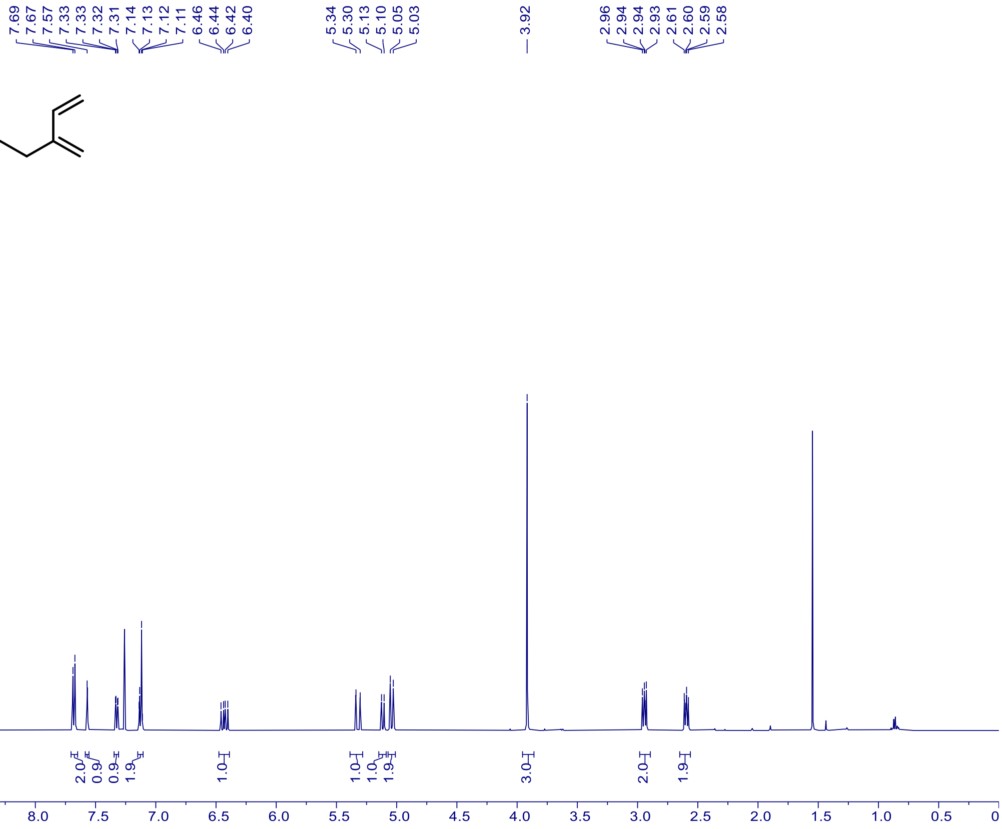

S36 – ^{13}C NMR (126 MHz, $CDCl_3$)

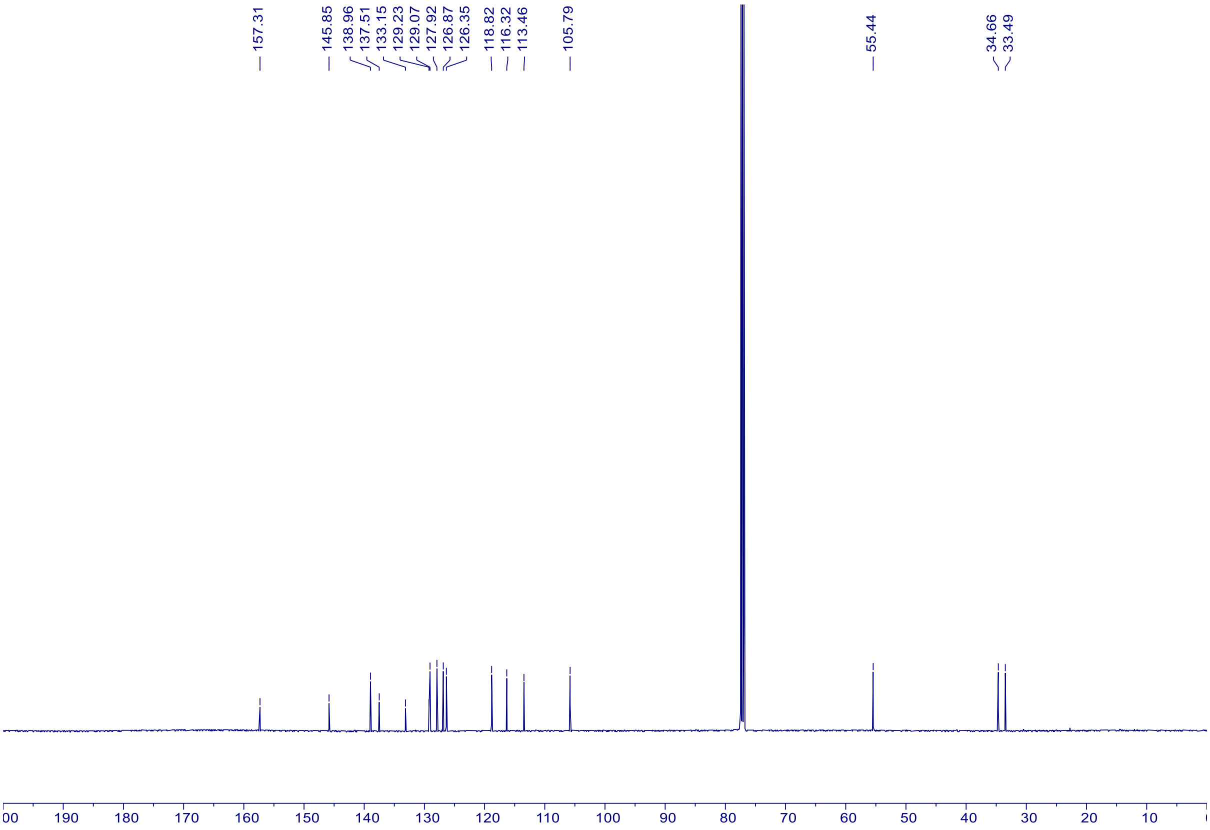

42 – ^{1}H NMR (500 MHz, $CDCl_3$)

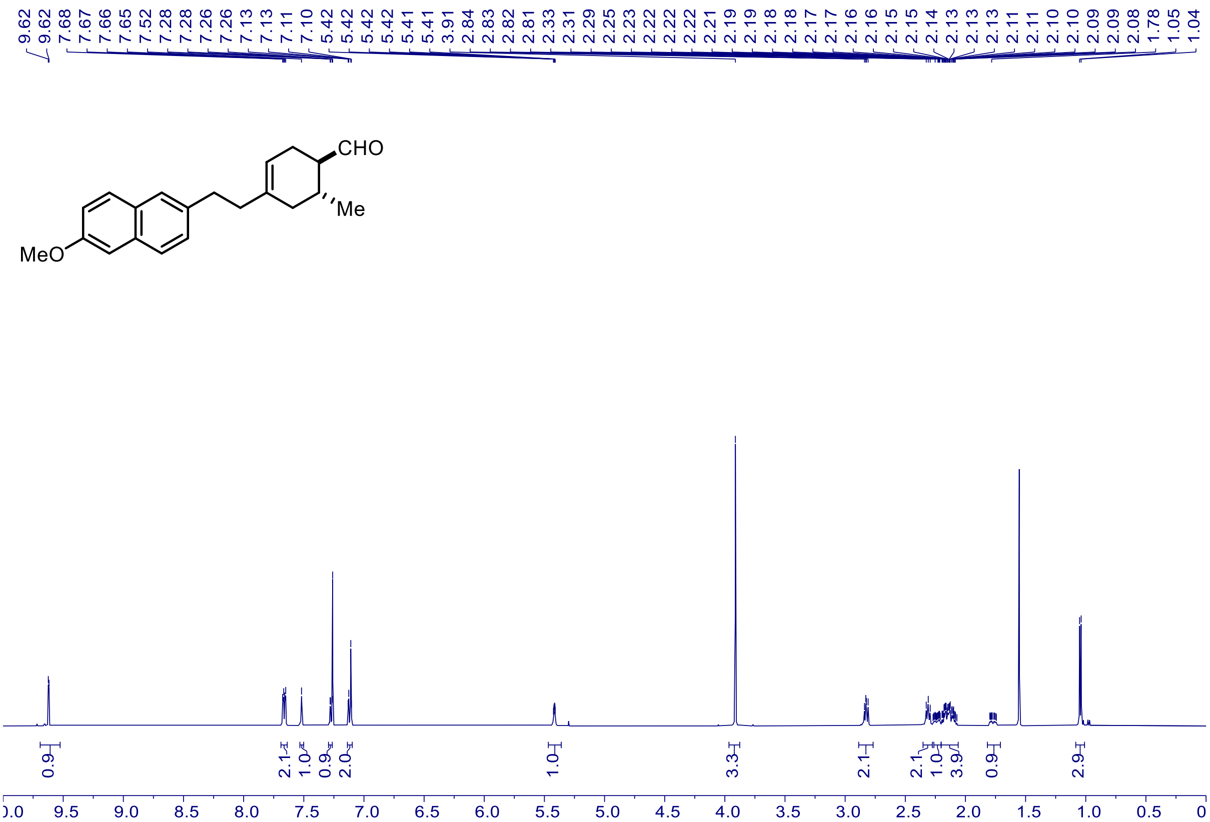

42 – ^{13}C NMR (126 MHz, $CDCl_3$)

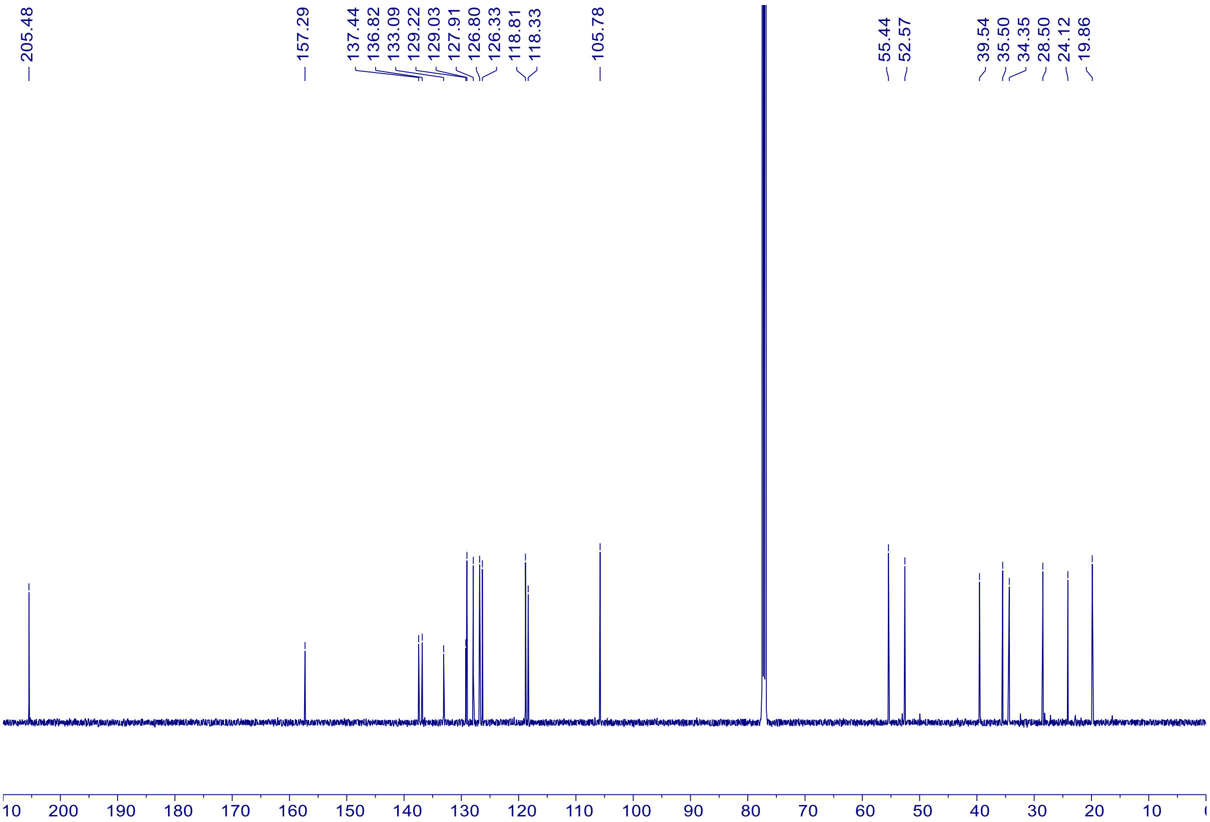

S38 – ^{1}H NMR (500 MHz, $CDCl_3$)

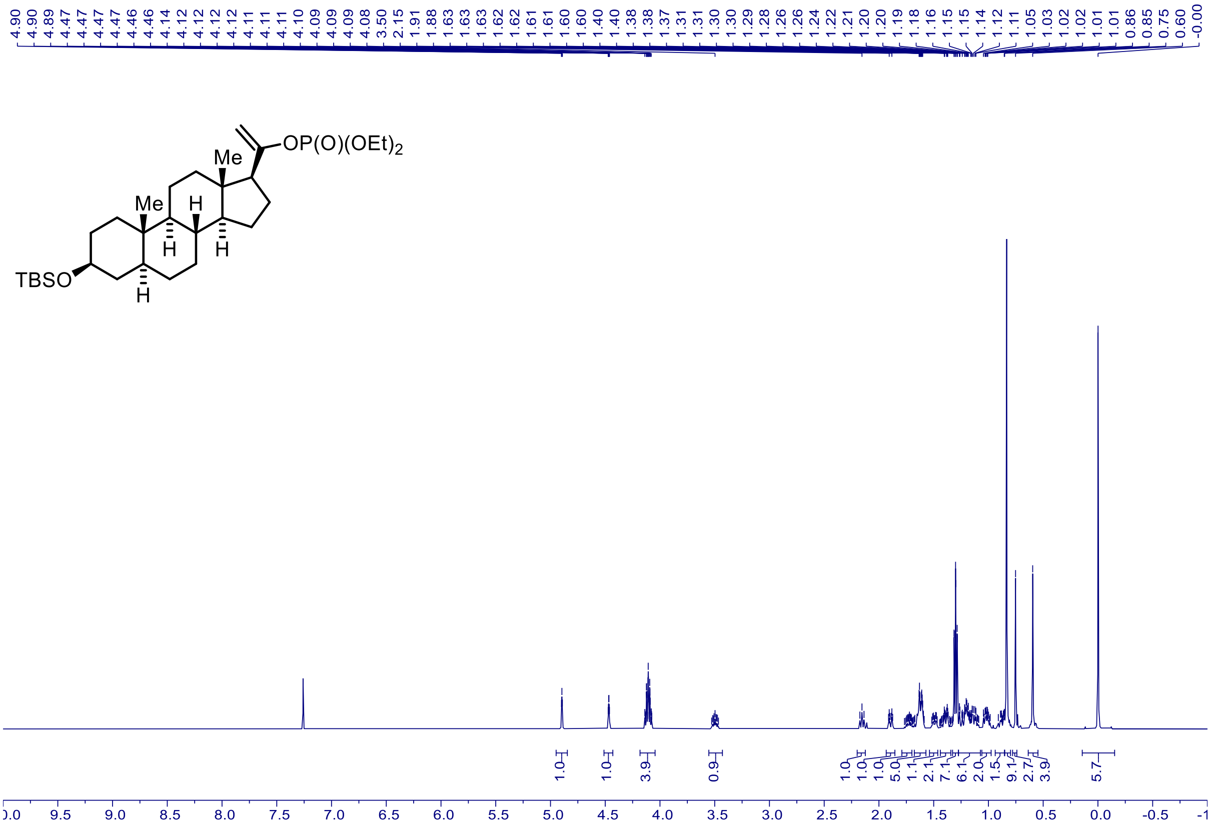

S38 – ^{13}C NMR (126 MHz, $CDCl_3$)

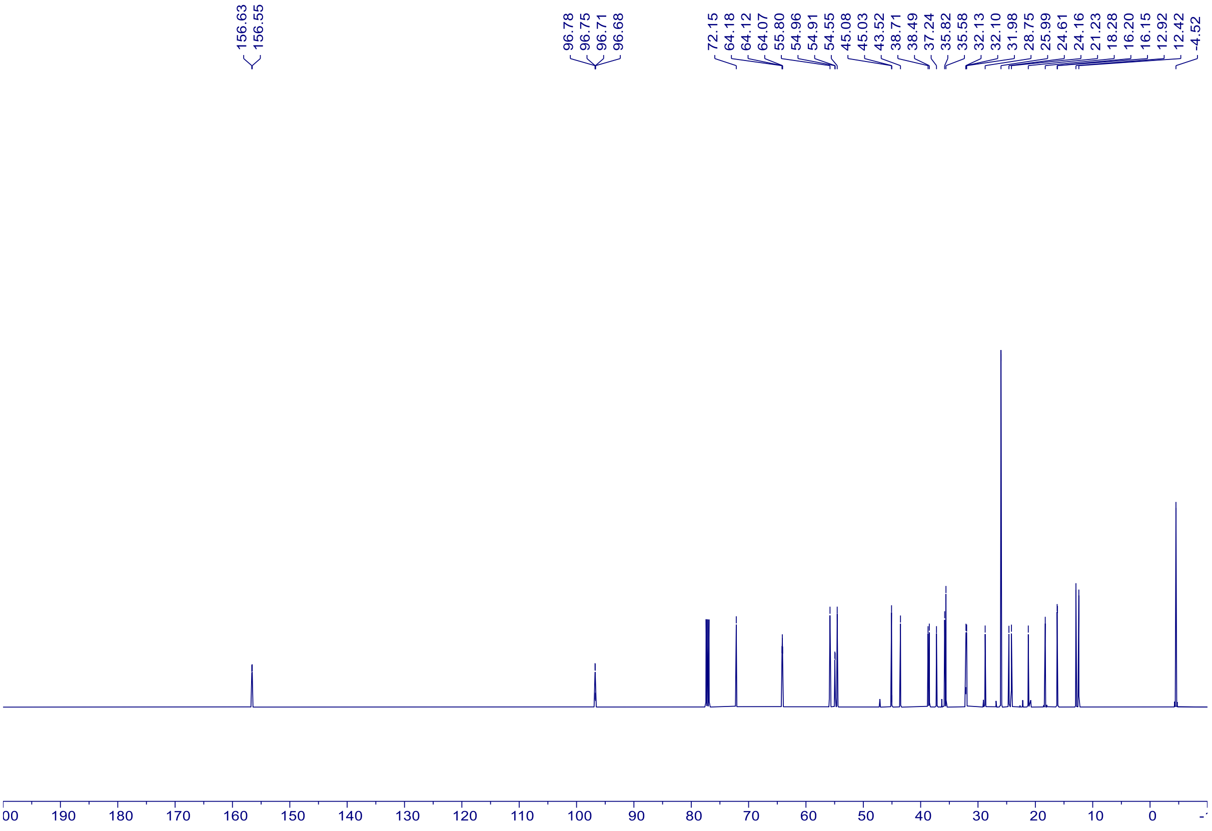

S38 – ^{31}P NMR (162 MHz, $CDCl_3$)

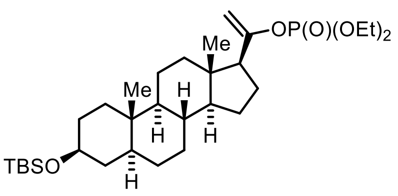

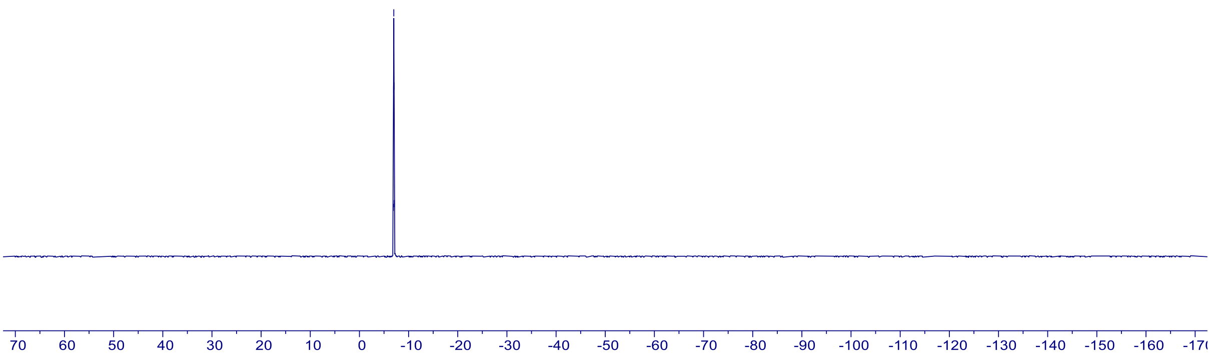

S39 – ^{1}H NMR (400 MHz, $CDCl_3$)

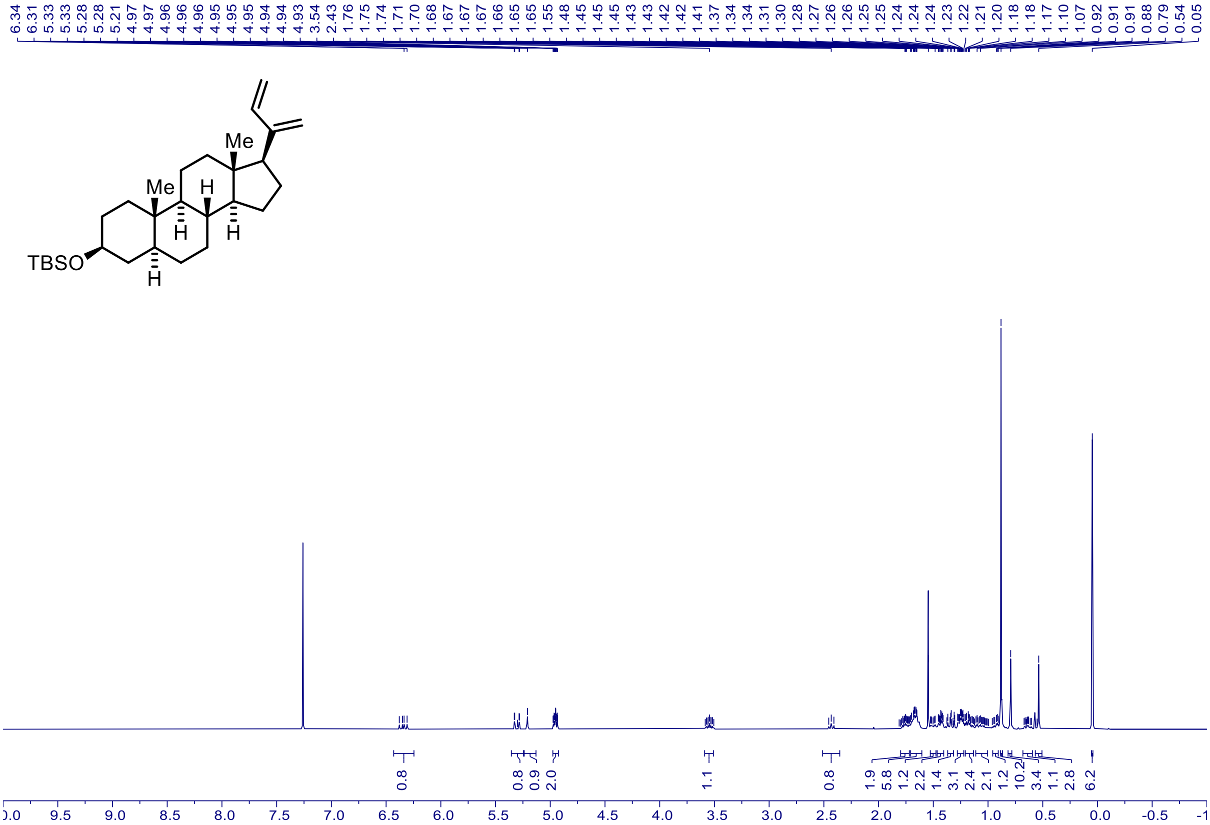

S39 – ^{13}C NMR (101 MHz, $CDCl_3$)

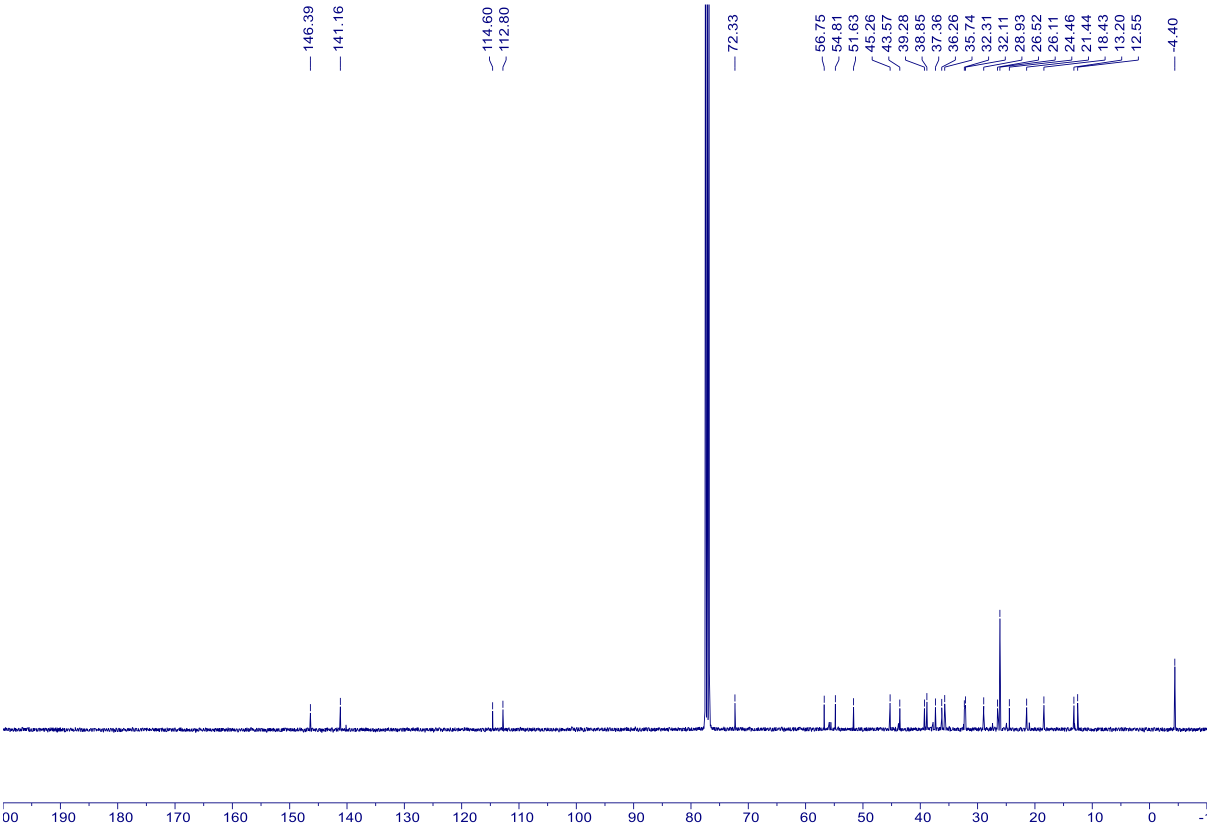

45 – ^{1}H NMR (500 MHz, $CDCl_3$)

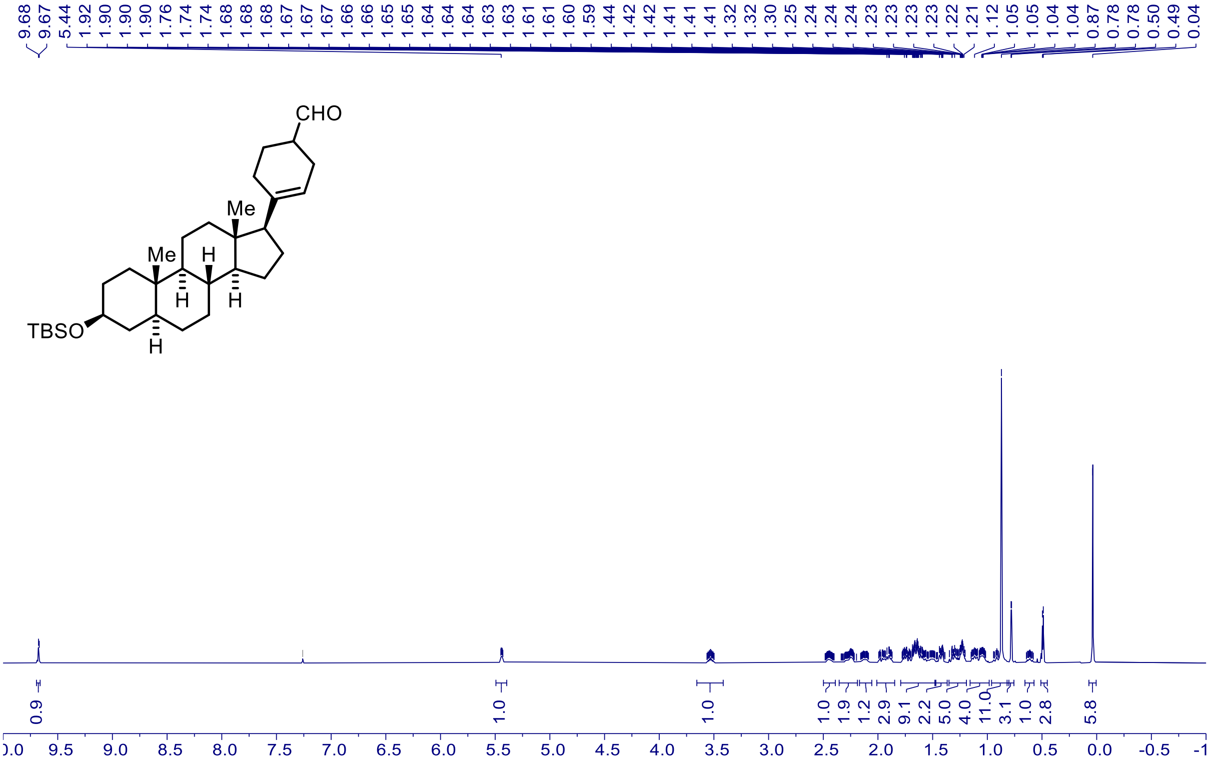

45 – ^{13}C NMR (126 MHz, $CDCl_3$)

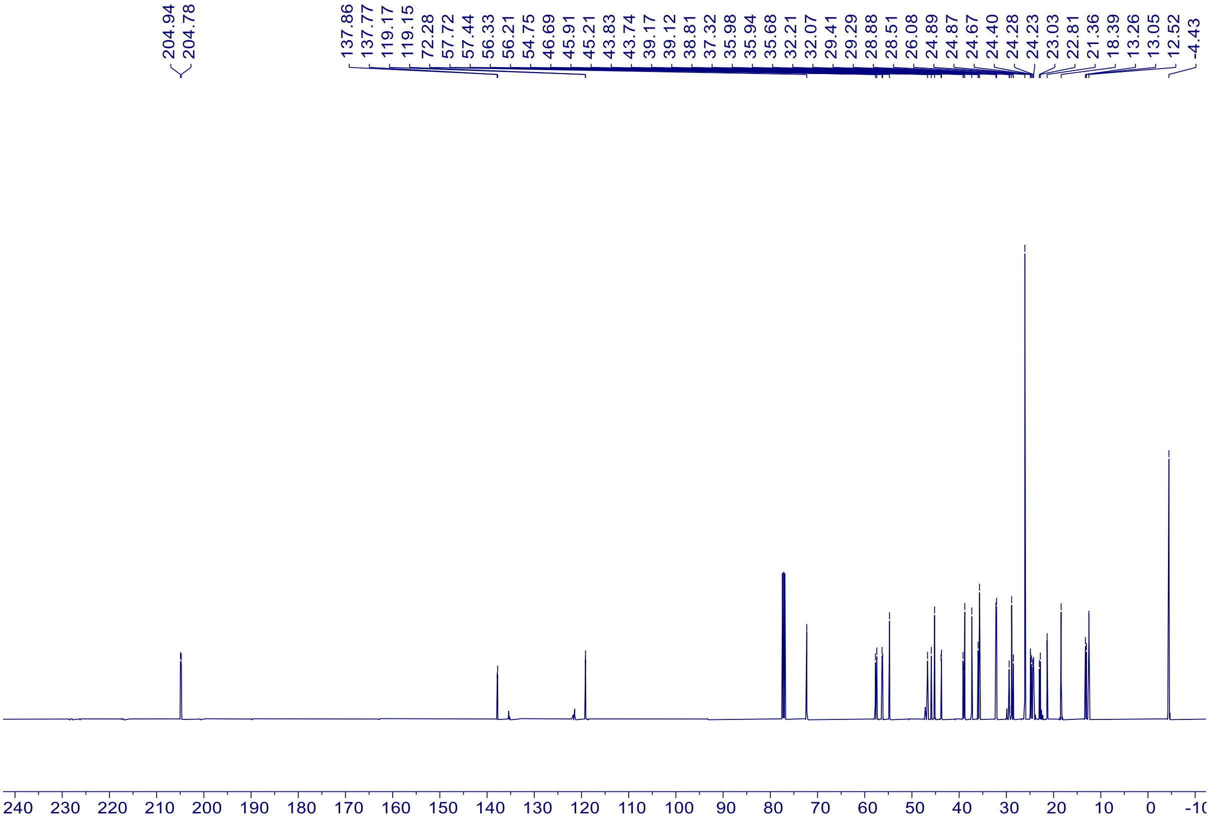

3 – ^{1}H NMR (400 MHz, C_6D_6)

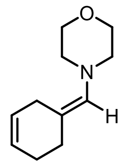

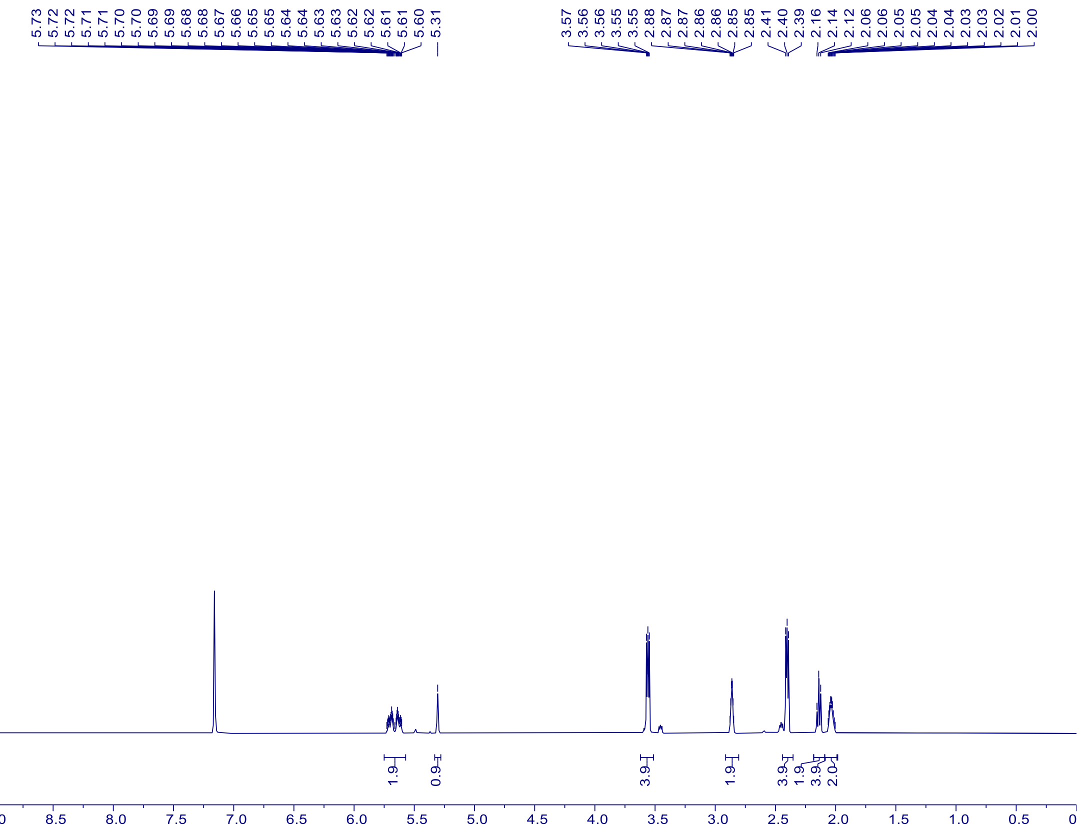

3 – ^{13}C NMR (101 MHz, C_6D_6)

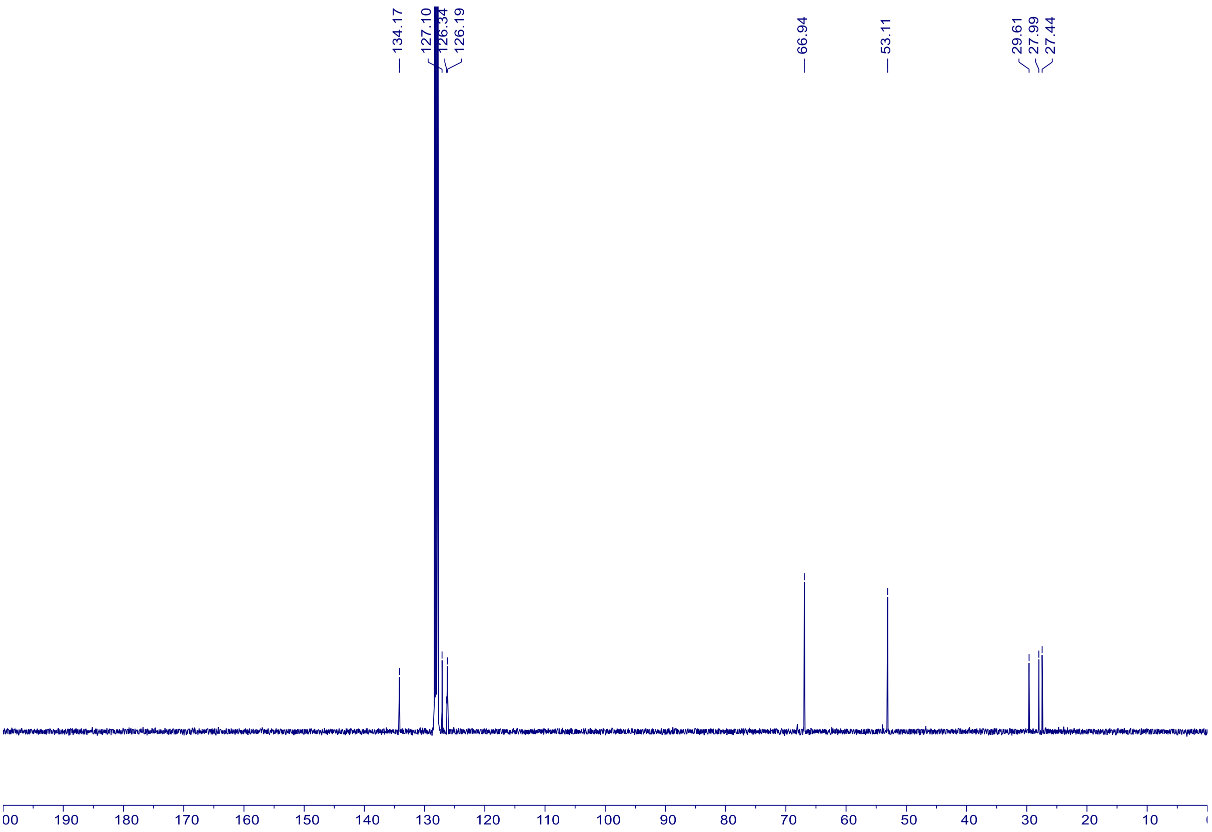

7 – ^{1}H NMR (400 MHz, $CDCl_3$)

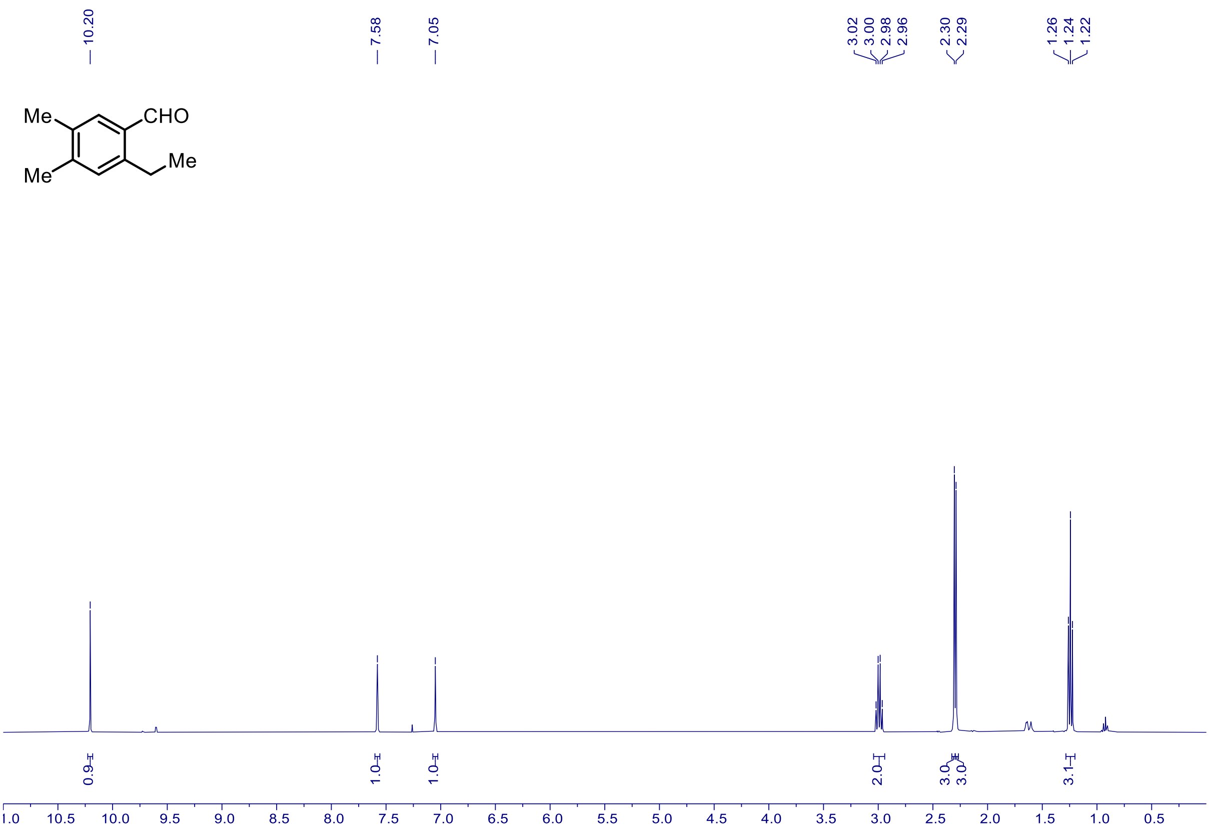

7 – ^{13}C NMR (101 MHz, $CDCl_3$)

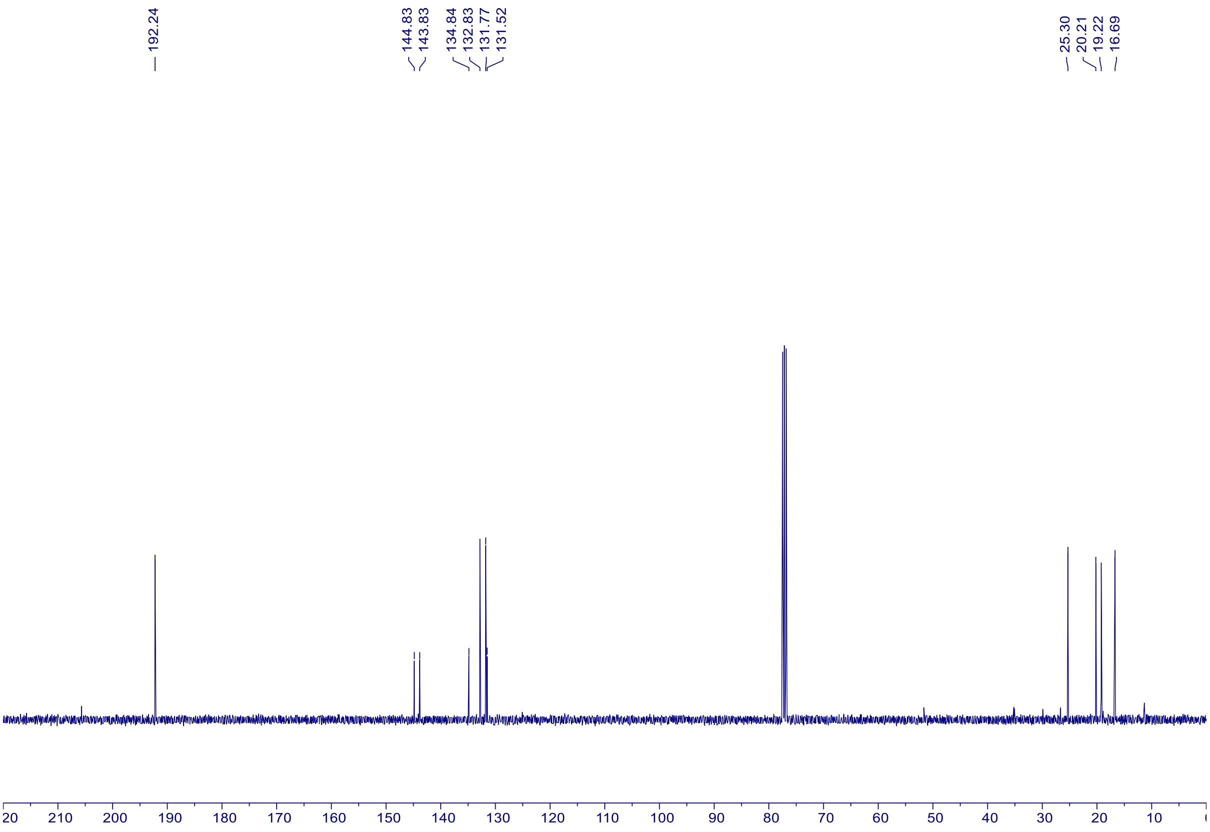

8 – ^{1}H NMR (400 MHz, CDCl$_3$)

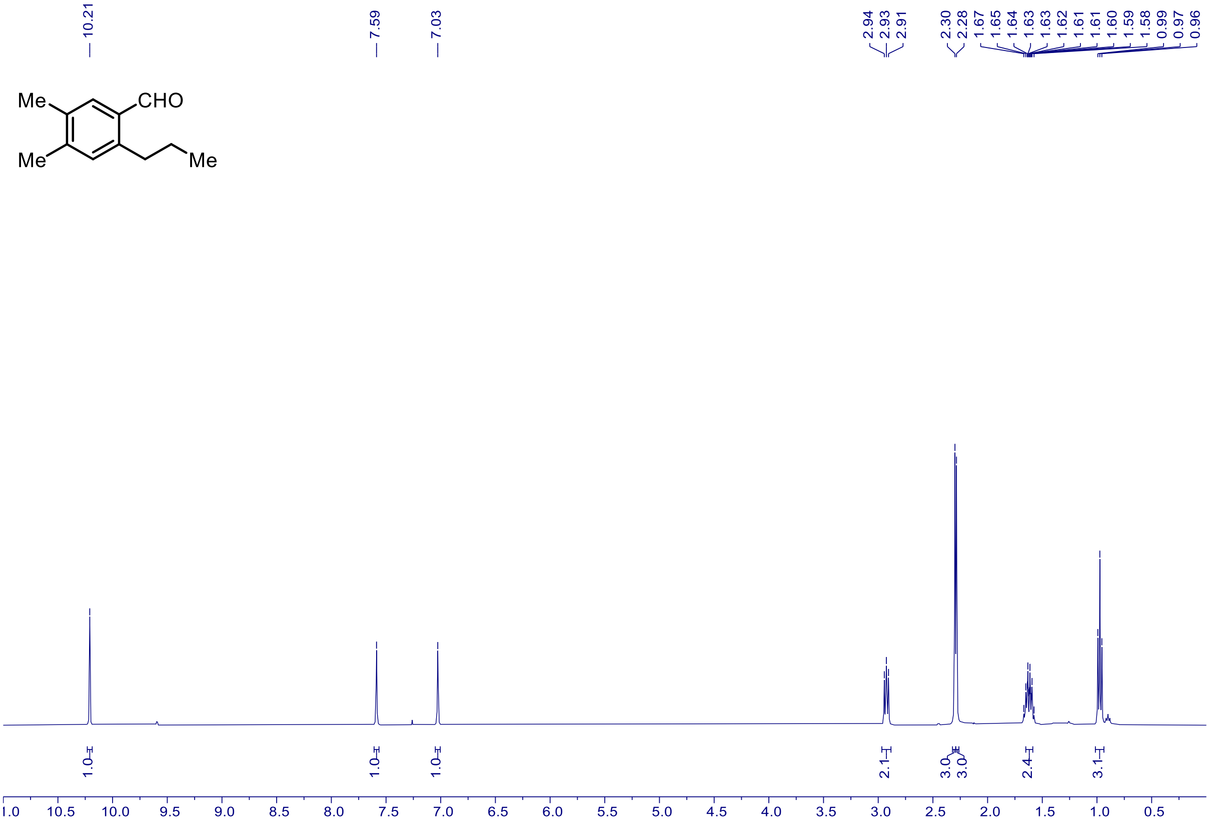

8 – ^{13}C NMR (101 MHz, CDCl$_3$)

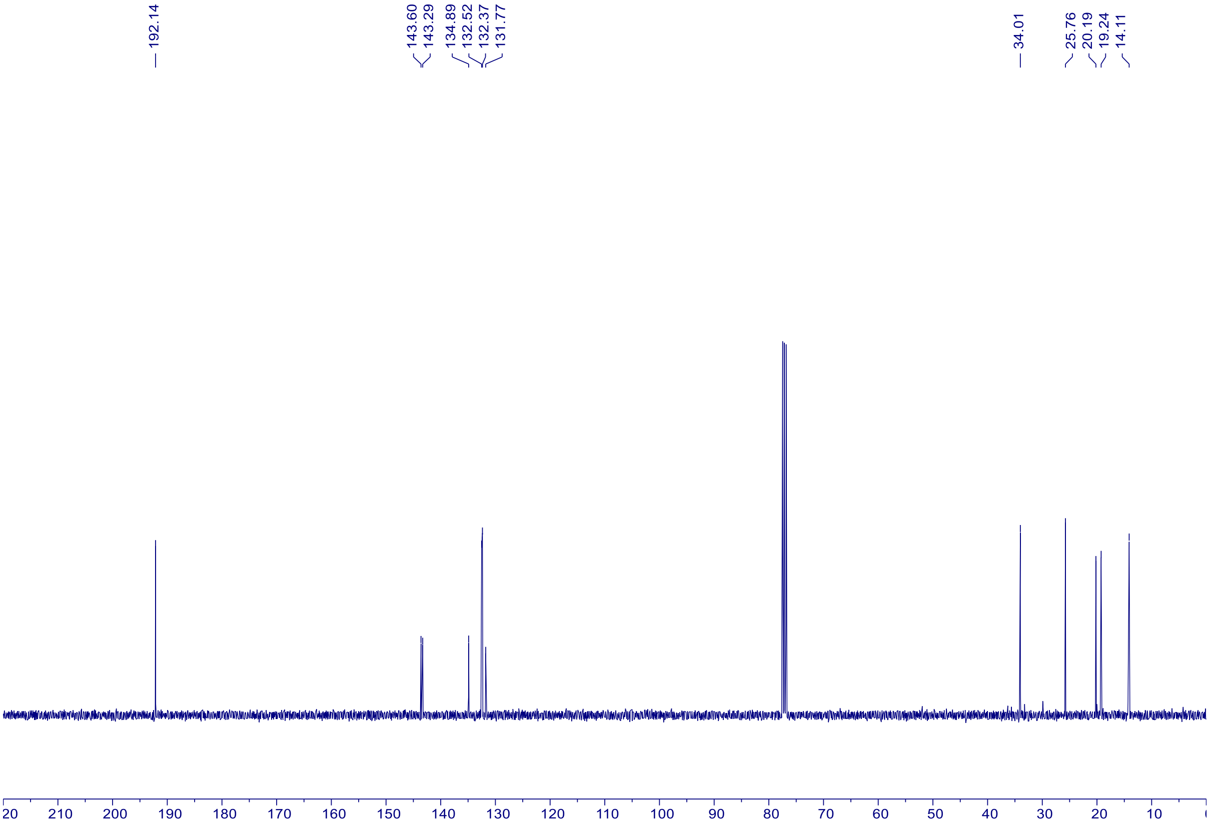

9 – ^{1}H NMR (400 MHz, $CDCl_3$)

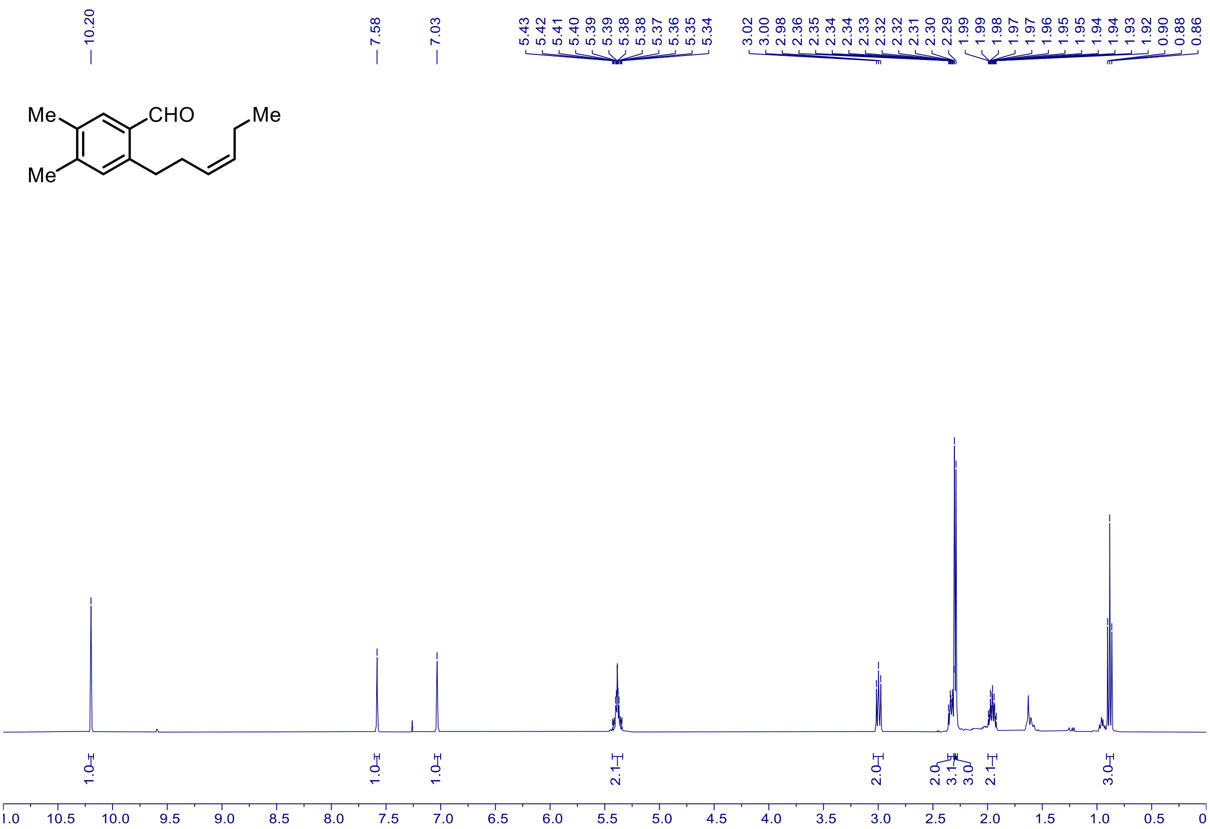

9 – ^{13}C NMR (101 MHz, $CDCl_3$)

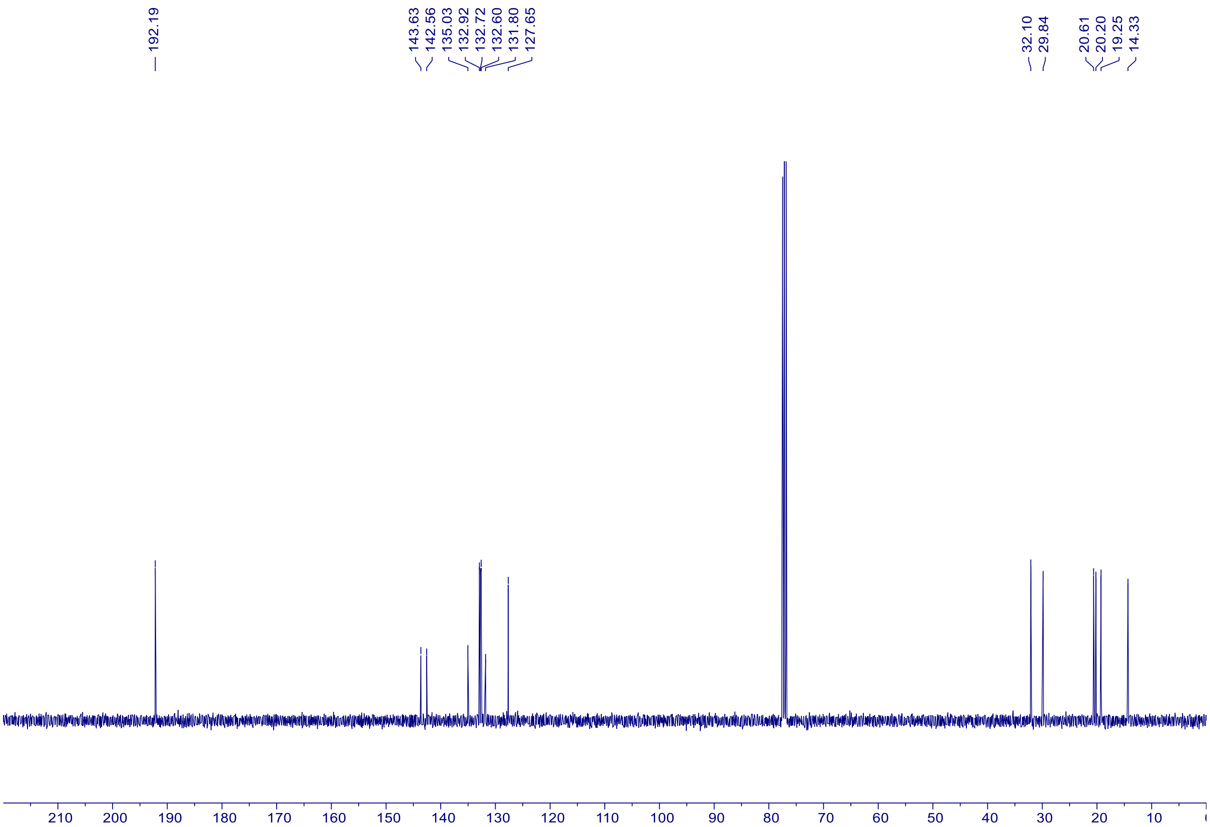

12 – ^{1}H NMR (400 MHz, $CDCl_3$)

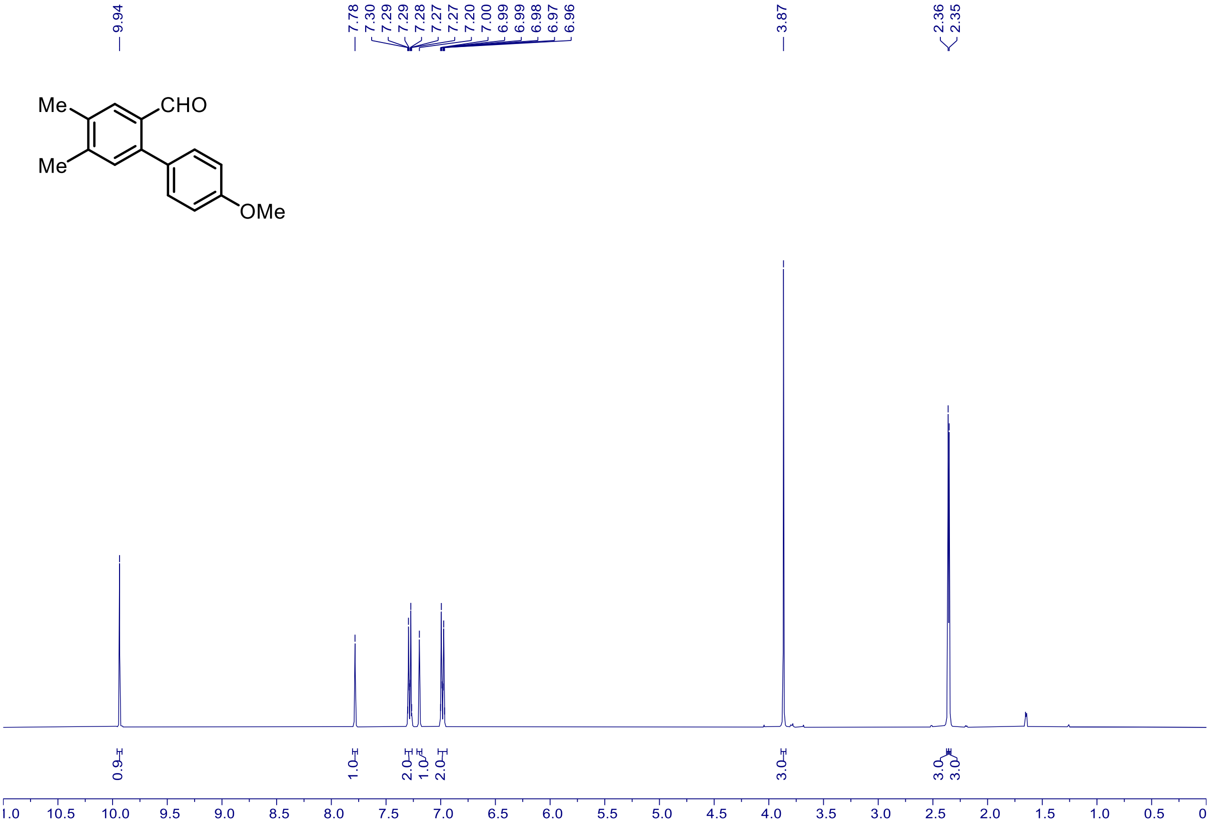

12 – ^{13}C NMR (101 MHz, $CDCl_3$)

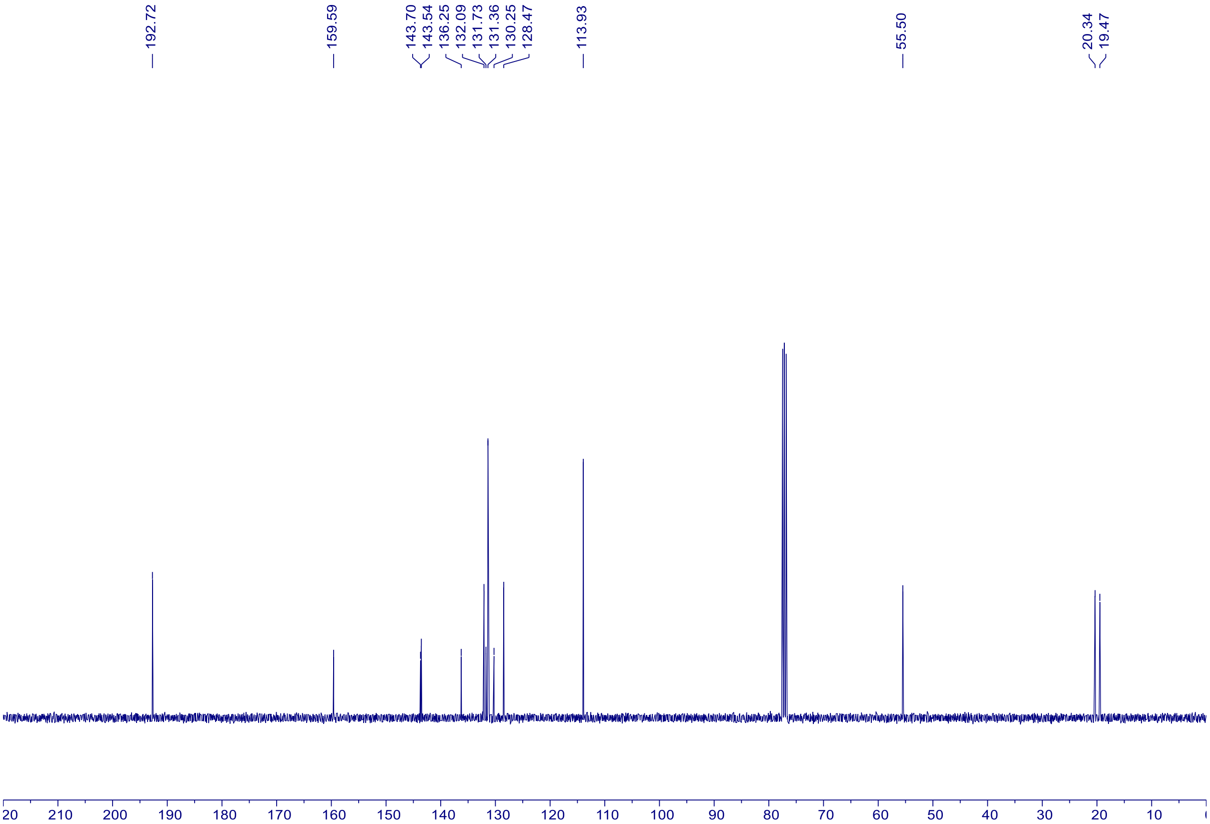

13 – ^{1}H NMR (500 MHz, $CDCl_3$)

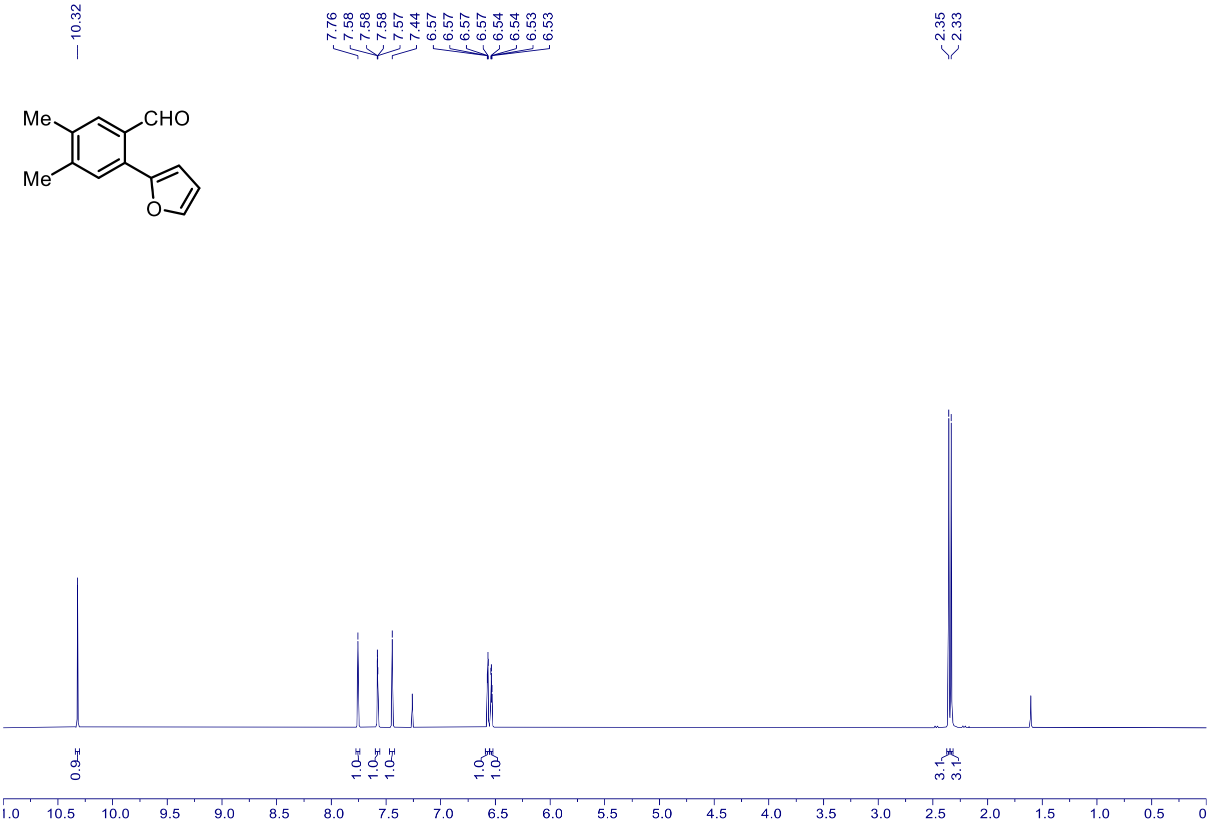

13 – ^{13}C NMR (126 MHz, $CDCl_3$)

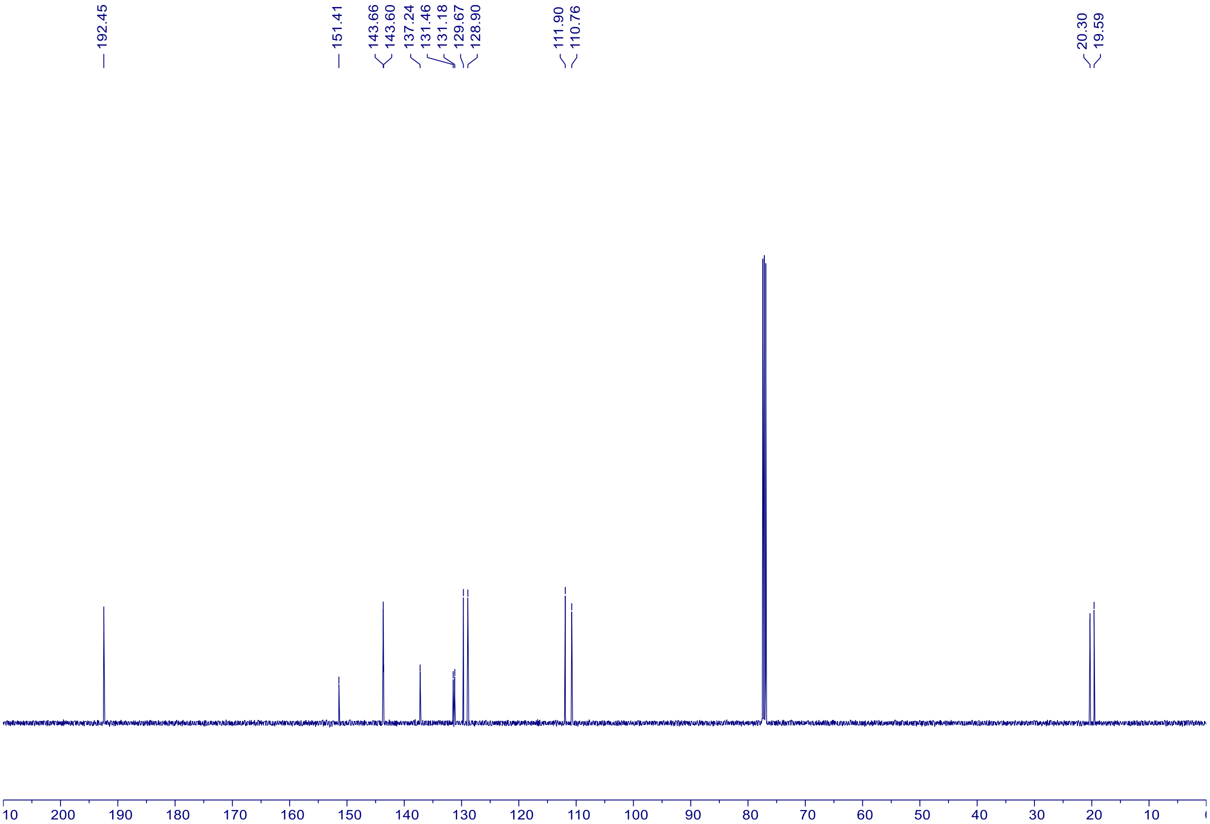

16 – ^{1}H NMR (400 MHz, $CDCl_3$)

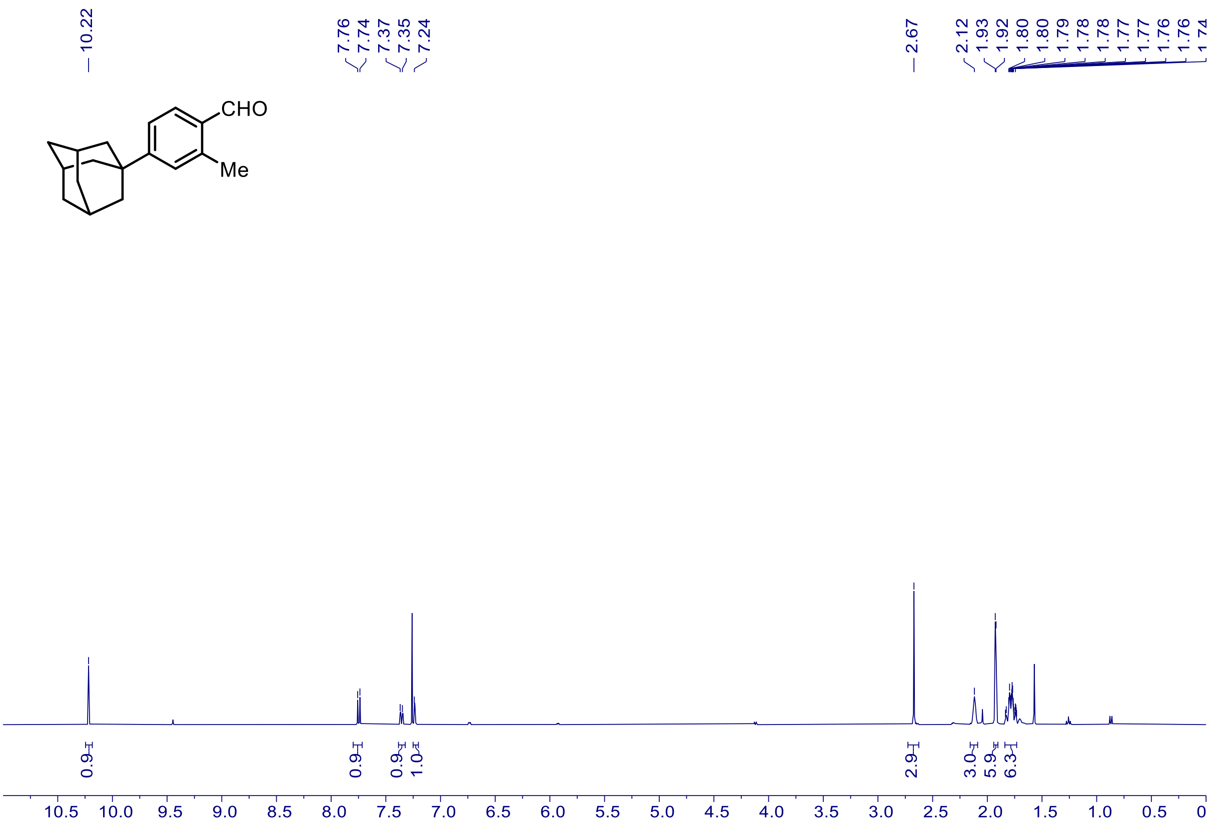

16 – ^{13}C NMR (101 MHz, $CDCl_3$)

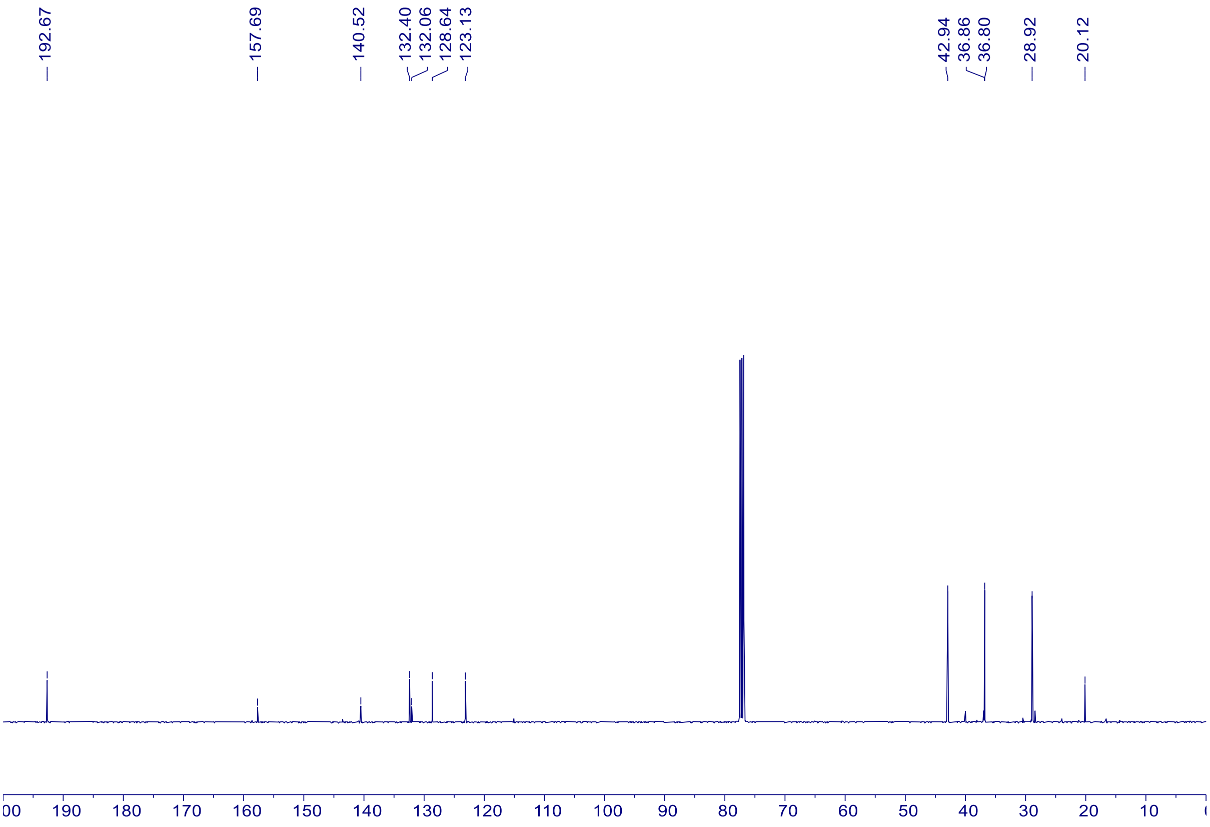

17 – ^{1}H NMR (400 MHz, $CDCl_3$)

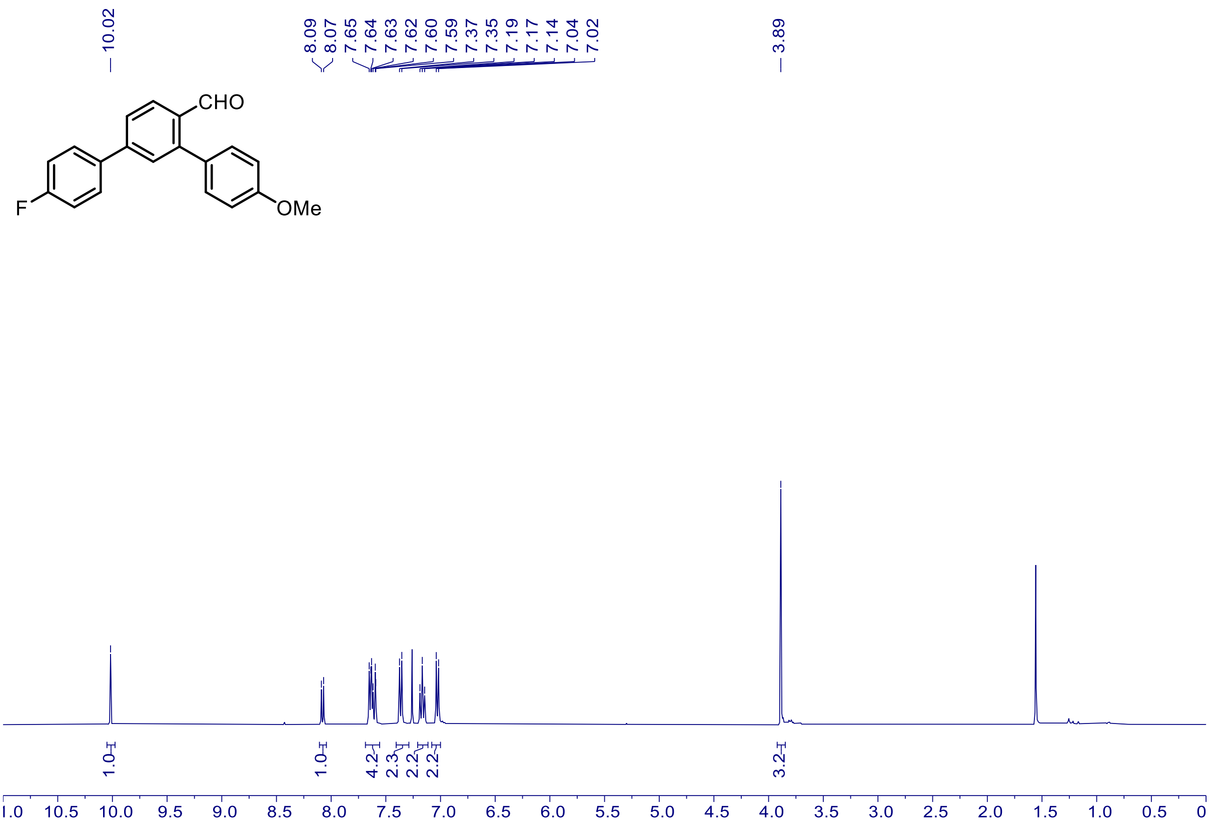

17 – ^{13}C NMR (101 MHz, $CDCl_3$)

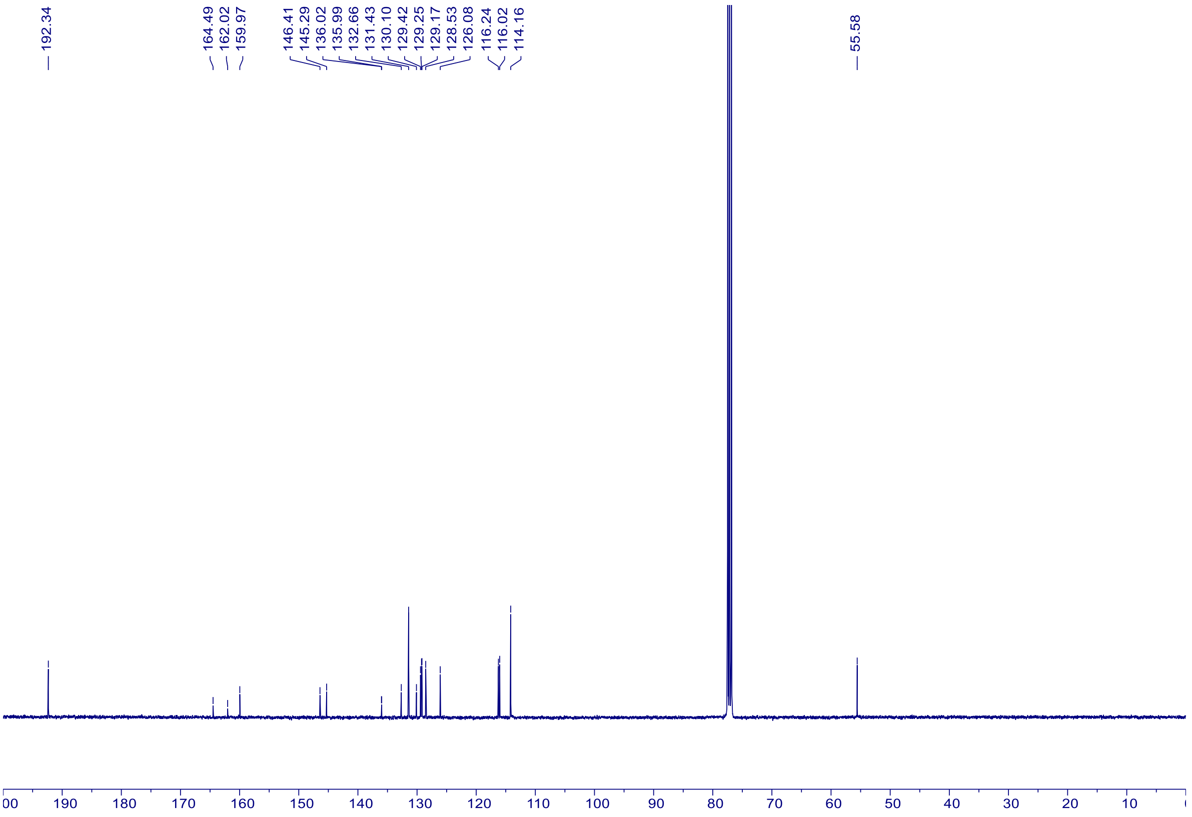

17 – ^{19}F NMR (376 MHz, $CDCl_3$)

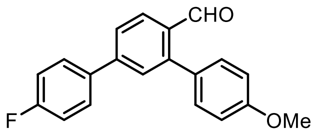

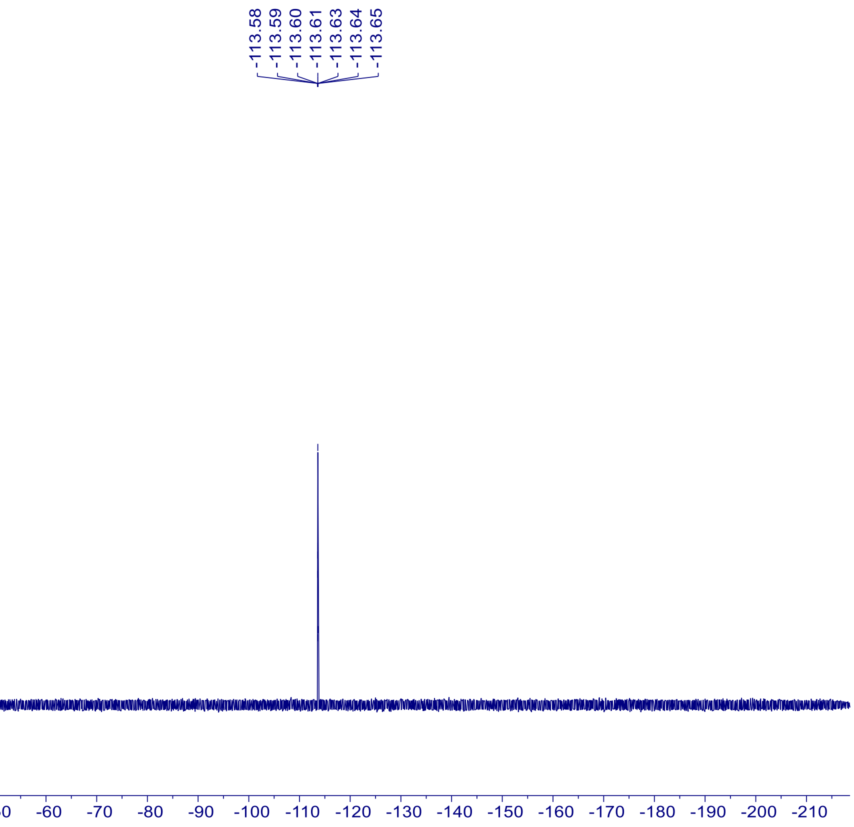

18 – ^{1}H NMR (500 MHz, $CDCl_3$)

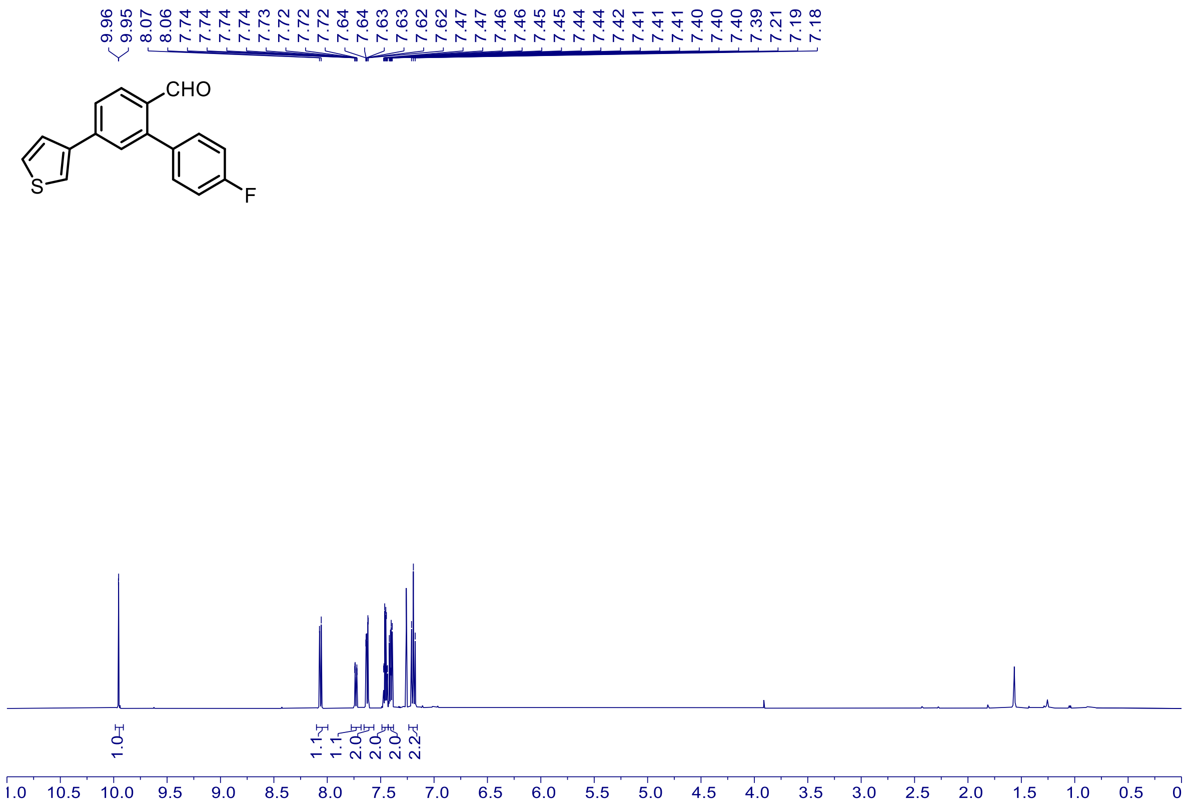

18 – ^{13}C NMR (126 MHz, $CDCl_3$)

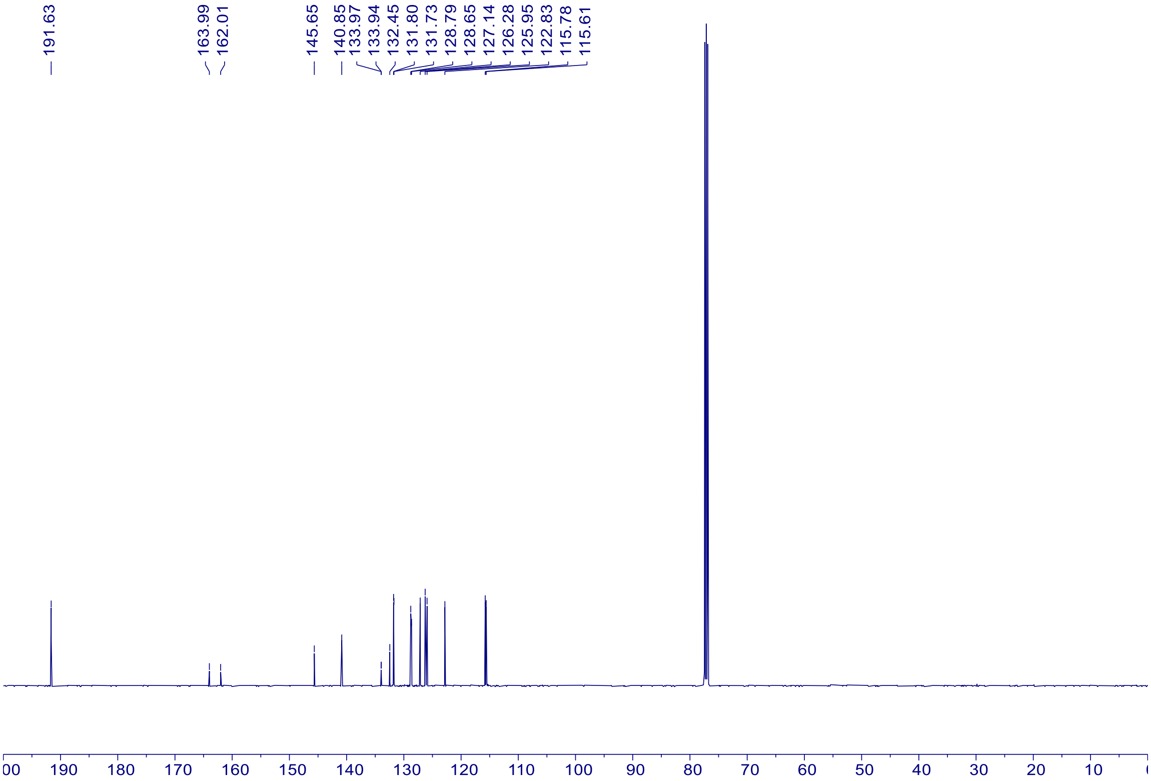

18 – ^{19}F NMR (376 MHz, $CDCl_3$)

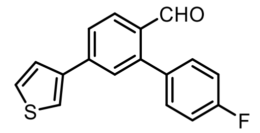

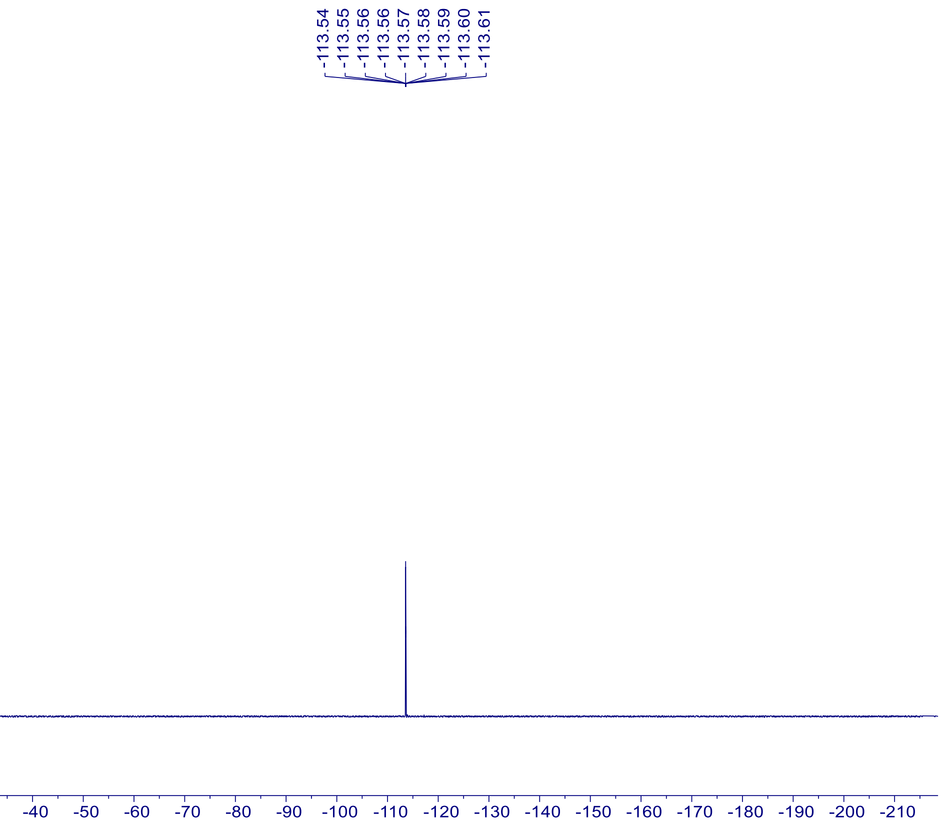

19 – ^{1}H NMR (400 MHz, $CDCl_3$)

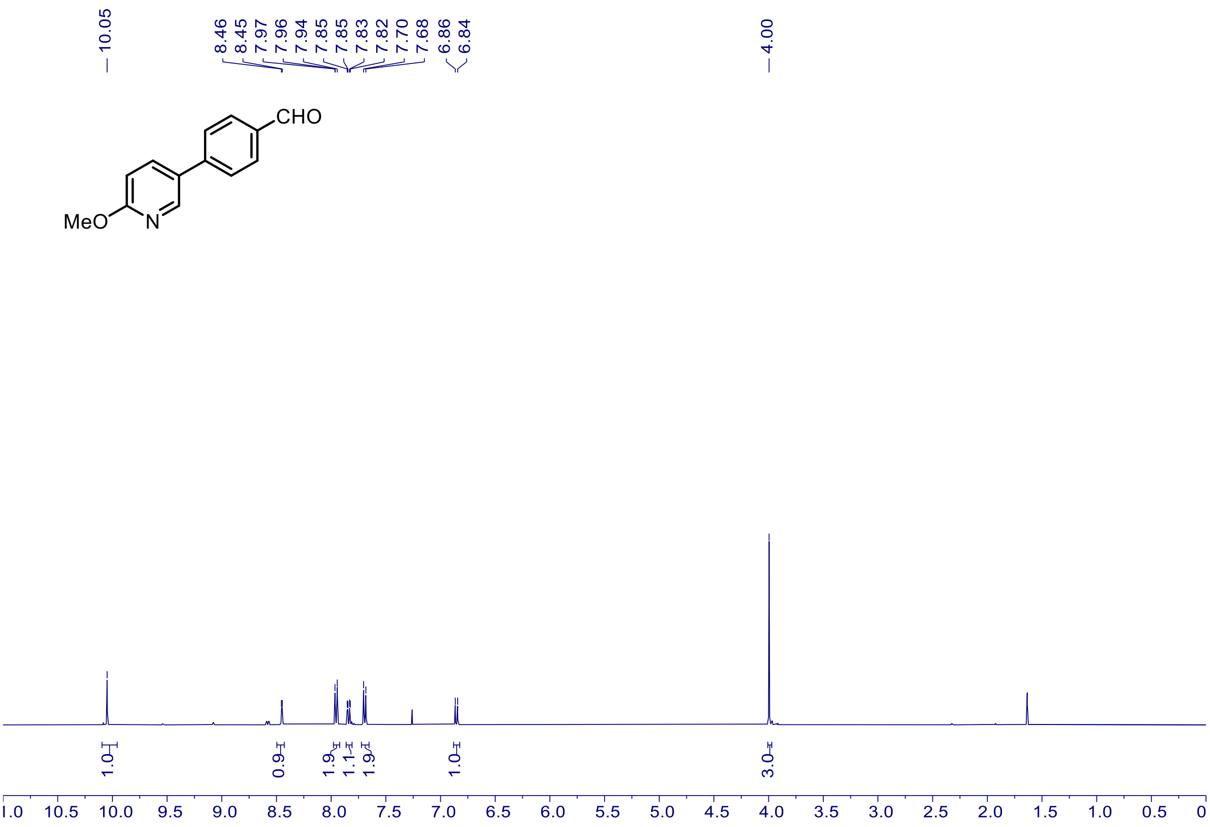

19 – ^{13}C NMR (101 MHz, $CDCl_3$)

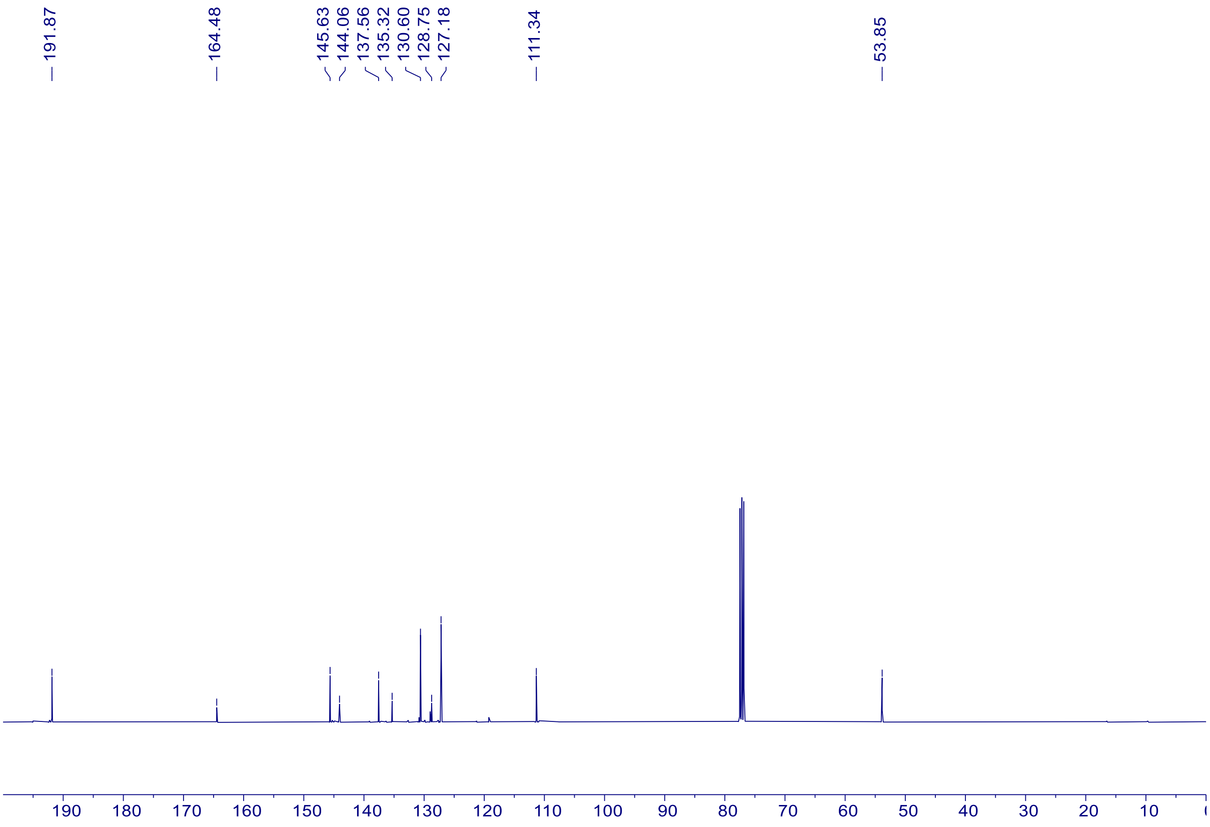

23 – ^{1}H NMR (400 MHz, $CDCl_3$)

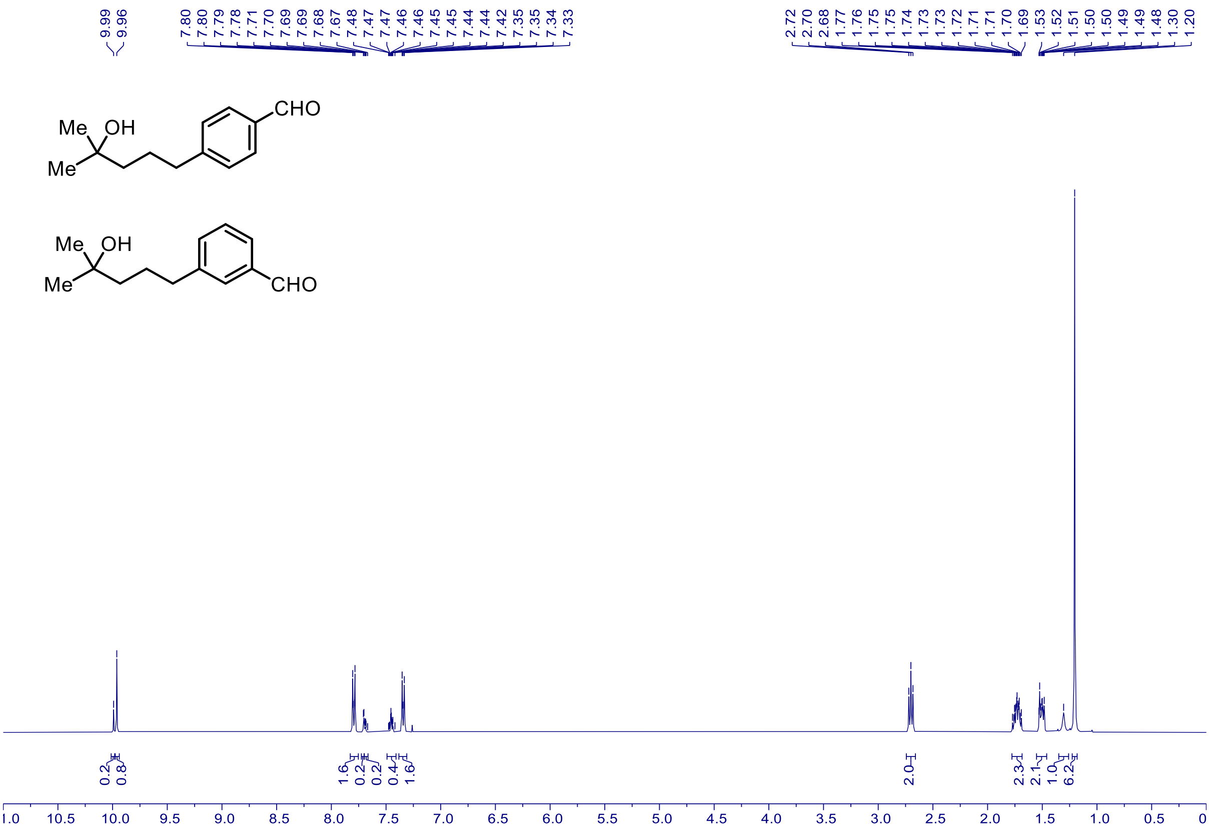

23 – ^{13}C NMR (101 MHz, $CDCl_3$)

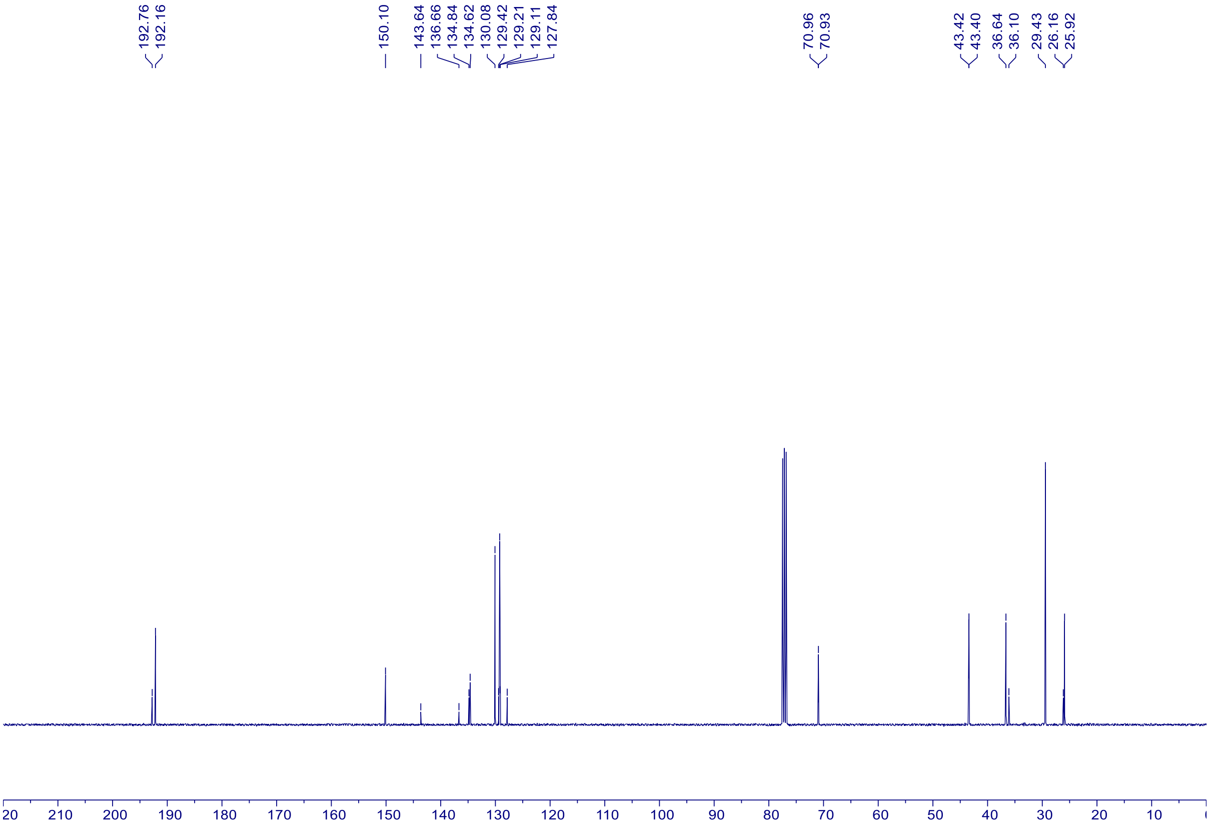

31 – ^{1}H NMR (500 MHz, CDCl$_3$)

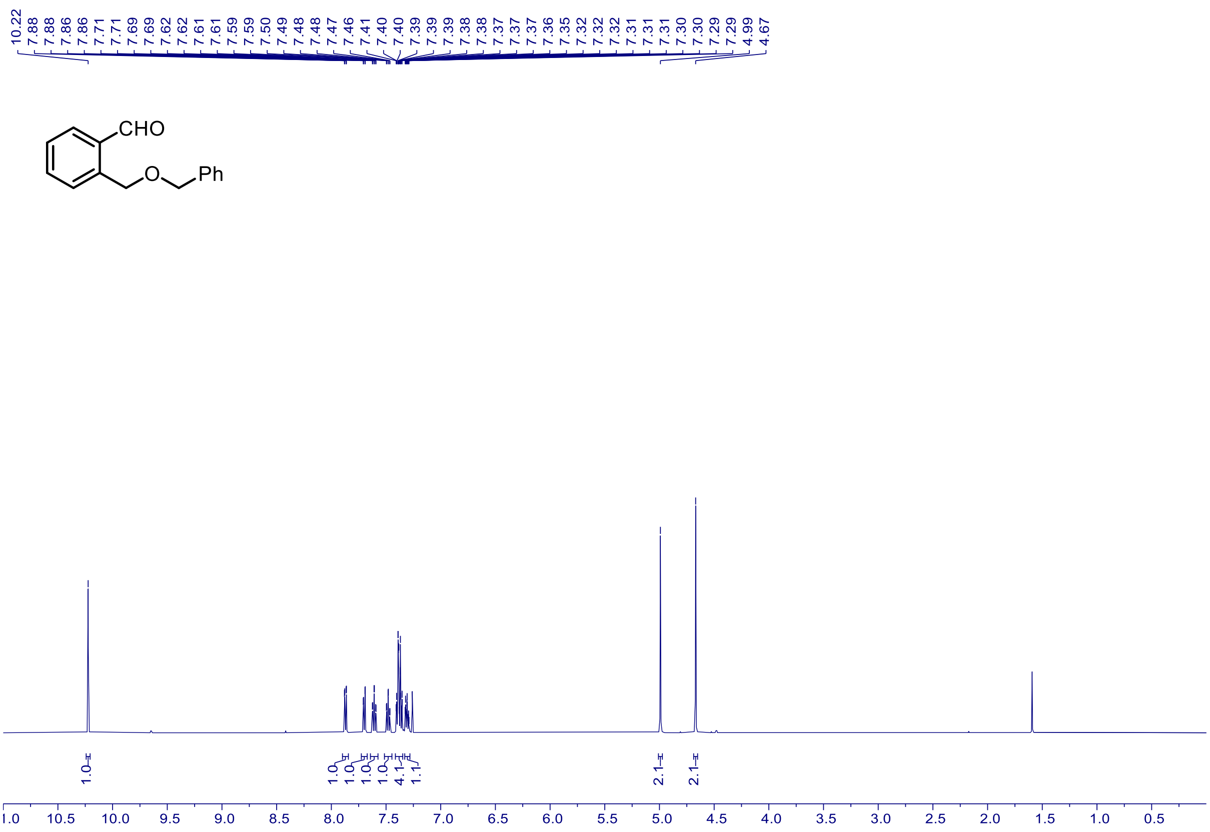

31 – ^{13}C NMR (126 MHz, CDCl$_3$)

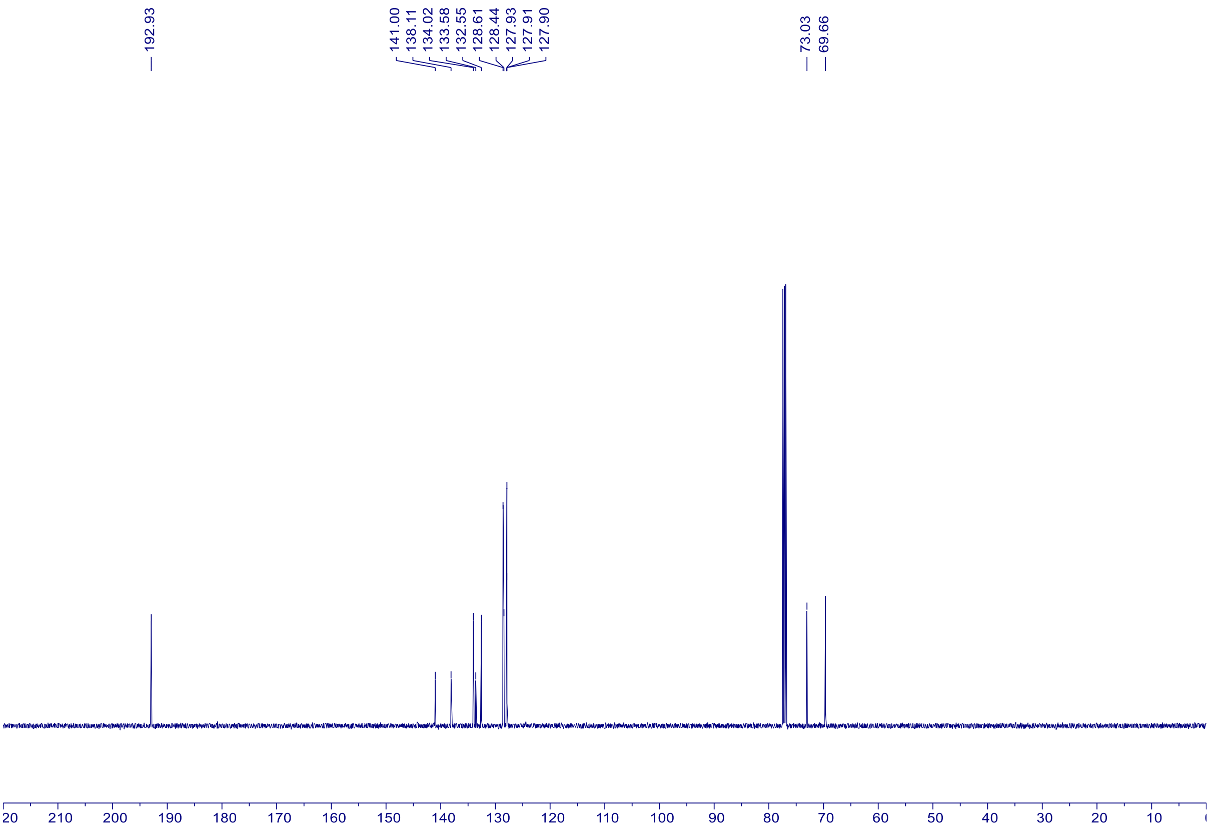

33 – ^{1}H NMR (500 MHz, $CDCl_3$)

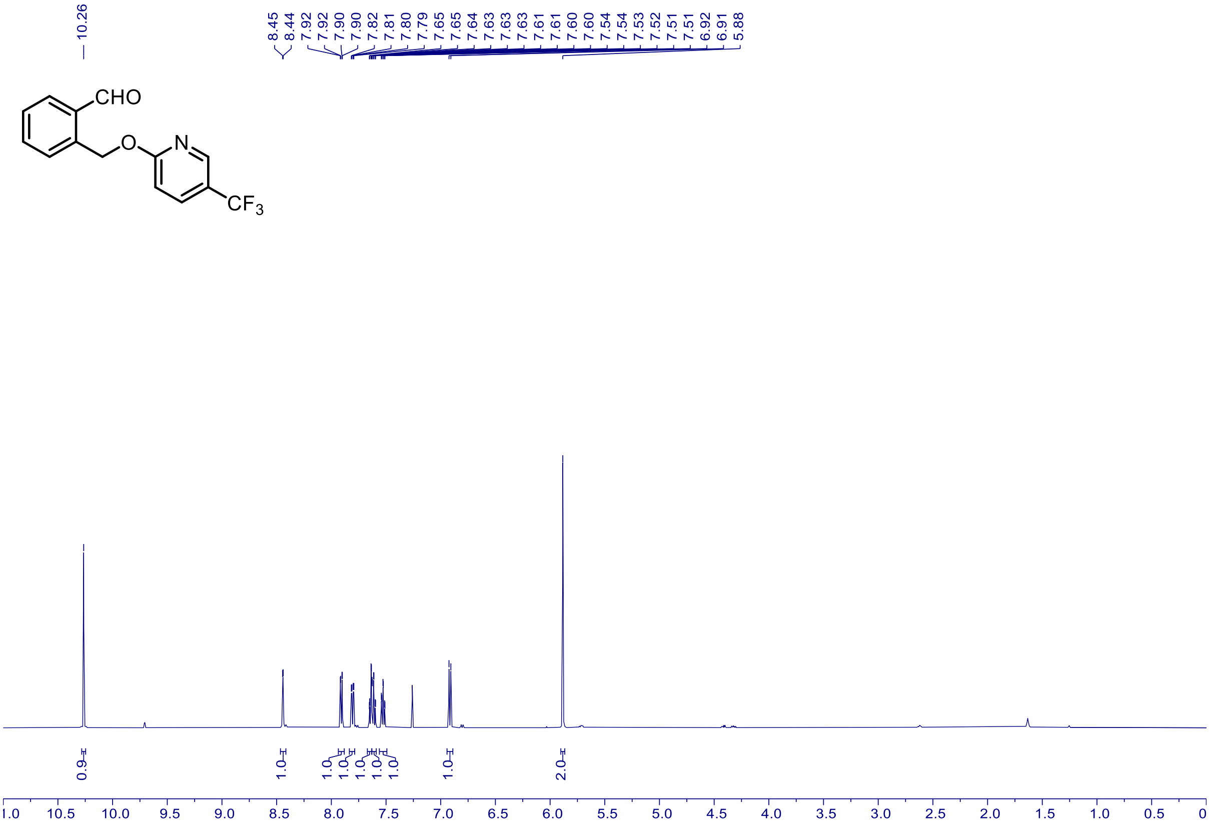

33 – ^{13}C NMR (126 MHz, $CDCl_3$)

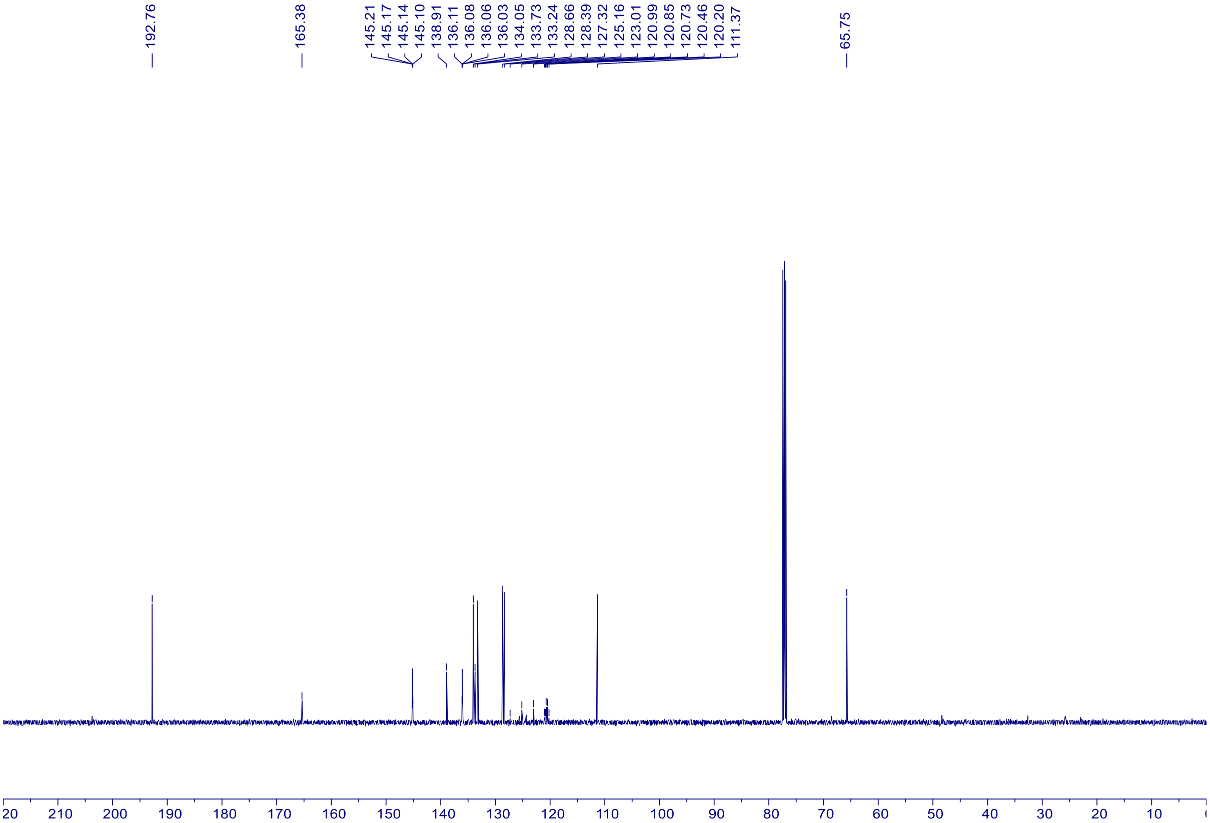

33 – ^{19}F NMR (376 MHz, $CDCl_3$)

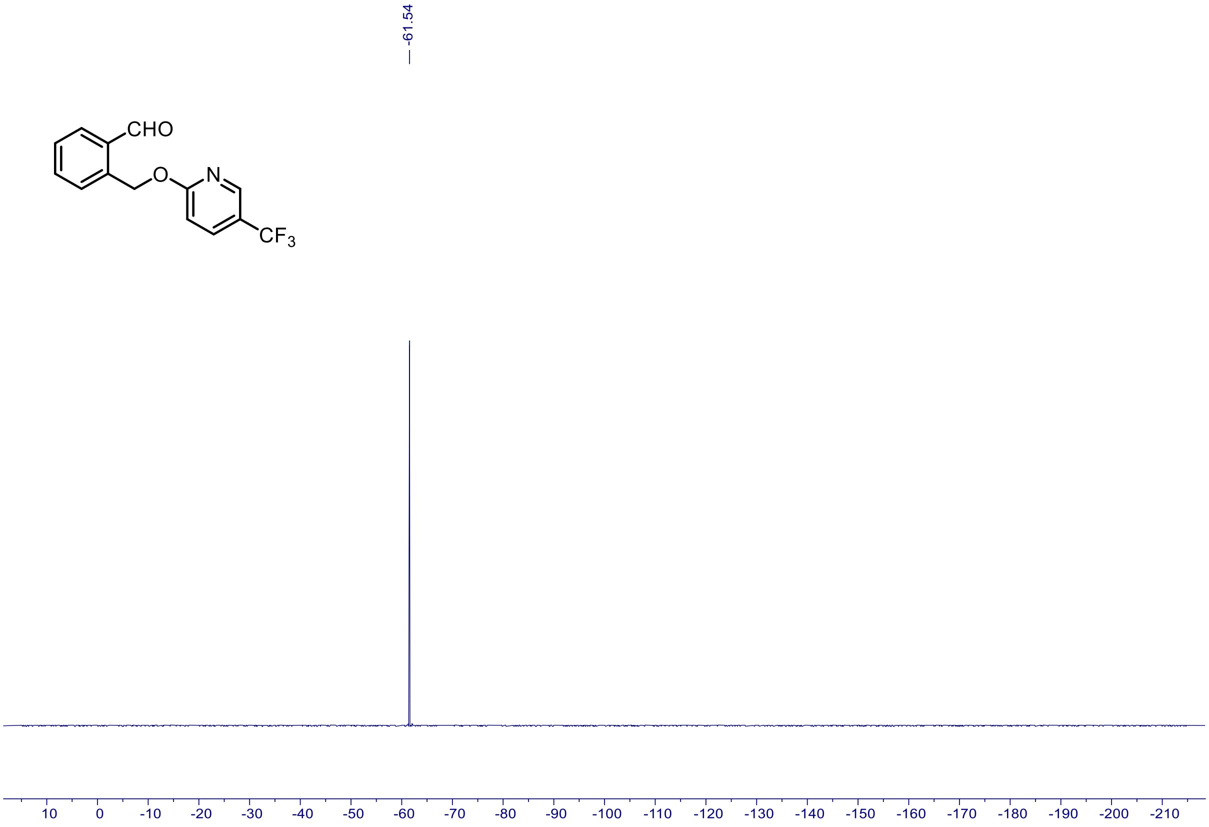

35 – ^{1}H NMR (400 MHz, $CDCl_3$)

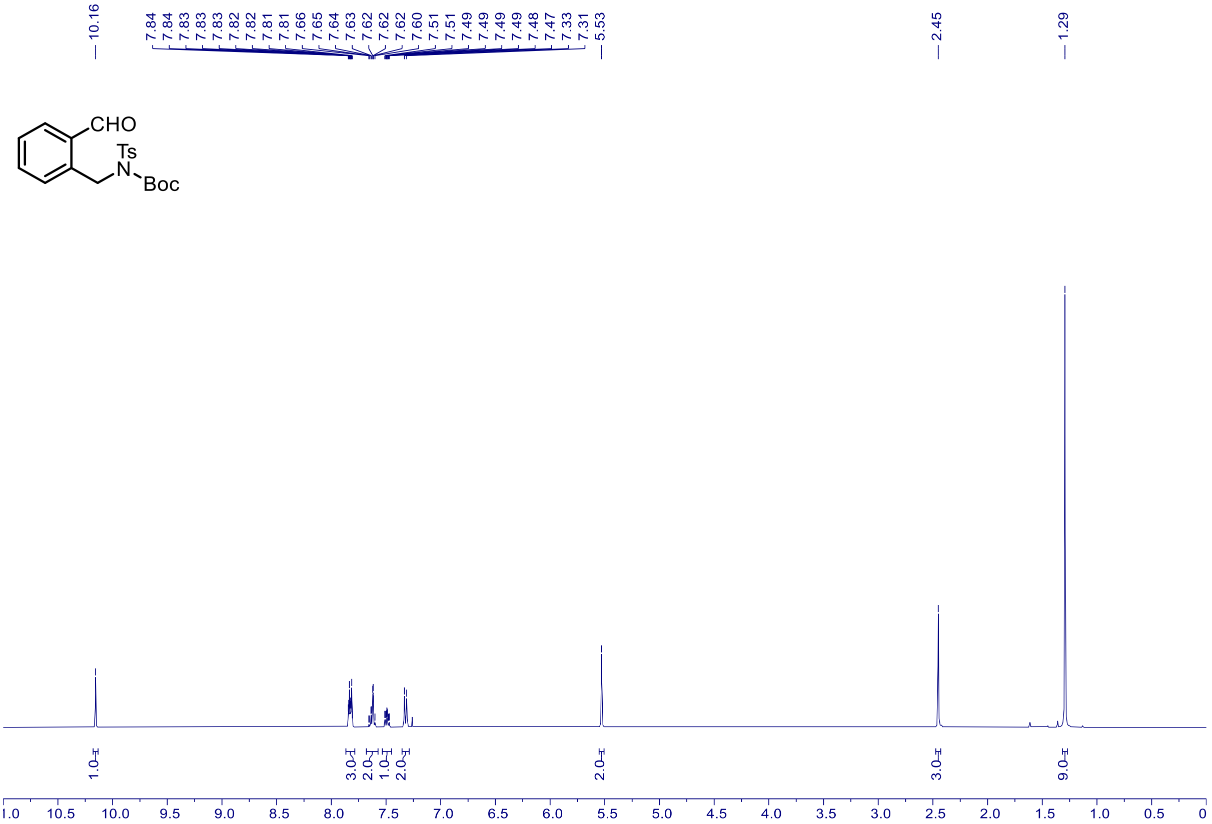

35 – ^{13}C NMR (101 MHz, $CDCl_3$)

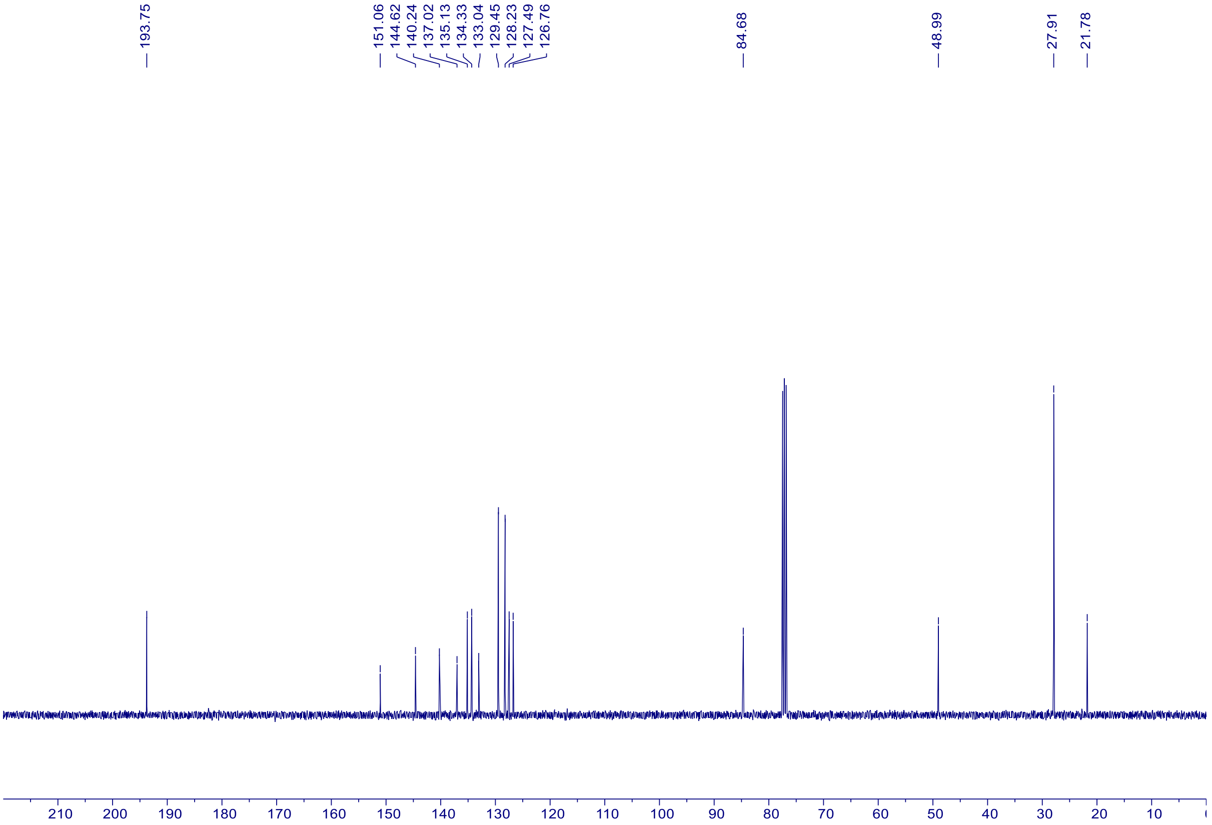

36 – ^{1}H NMR (400 MHz, $CDCl_3$)

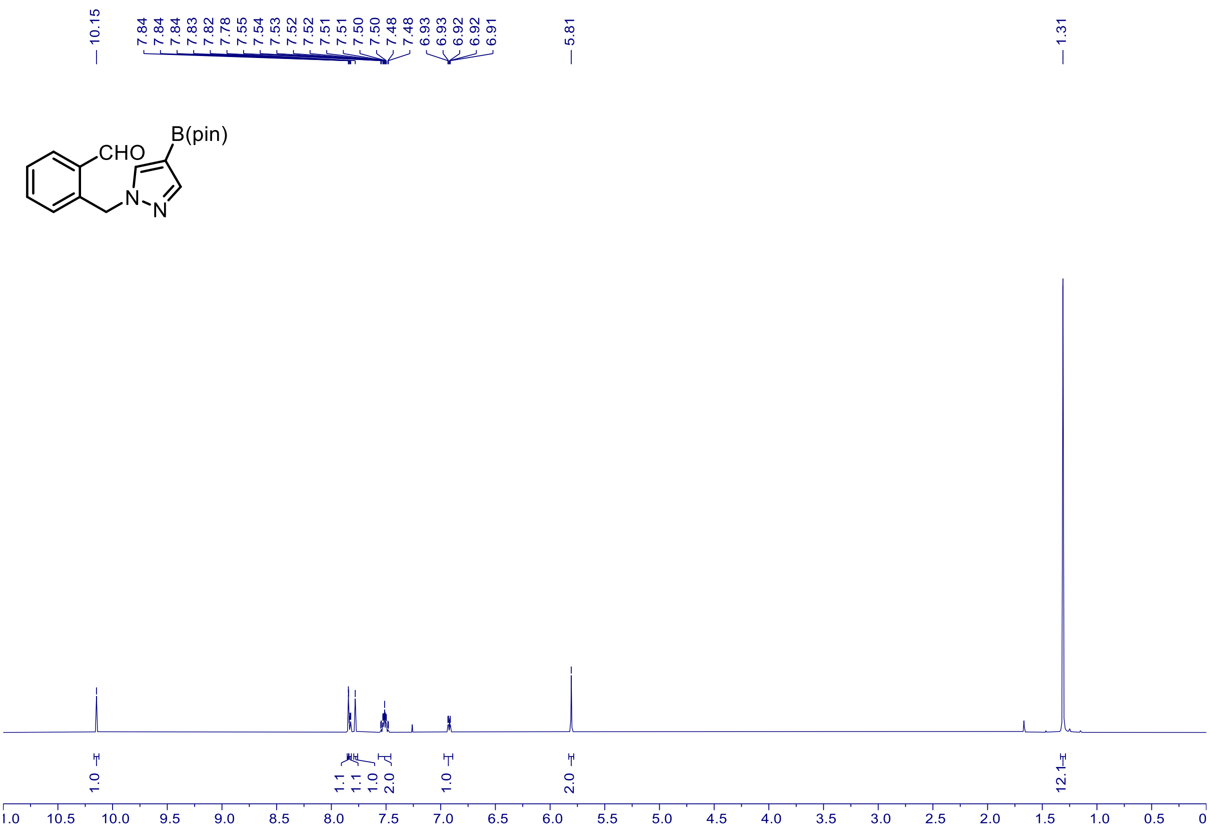

36 – ^{13}C NMR (101 MHz, $CDCl_3$)

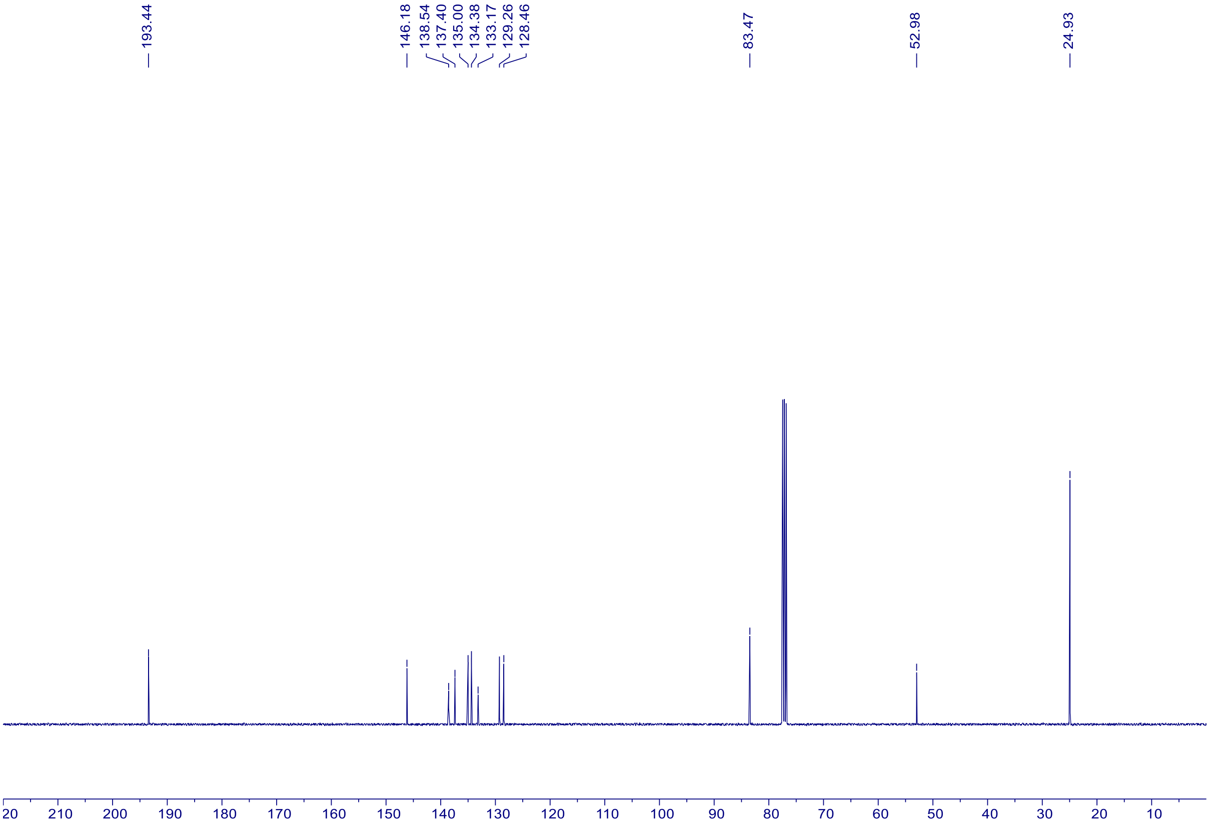

36 – ^{11}B NMR (128 MHz, $CDCl_3$)

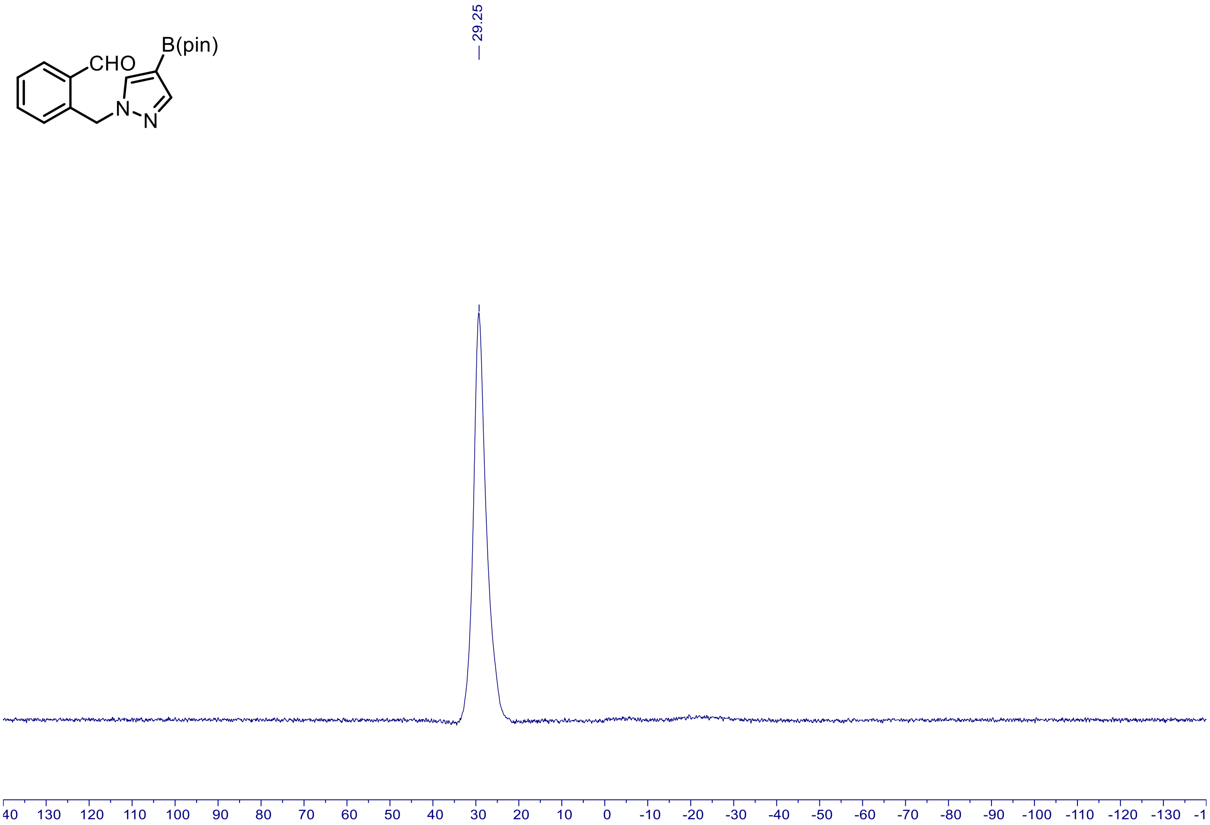

37 – ^{1}H NMR (500 MHz, $CDCl_3$)

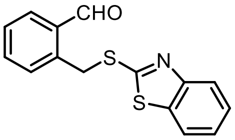

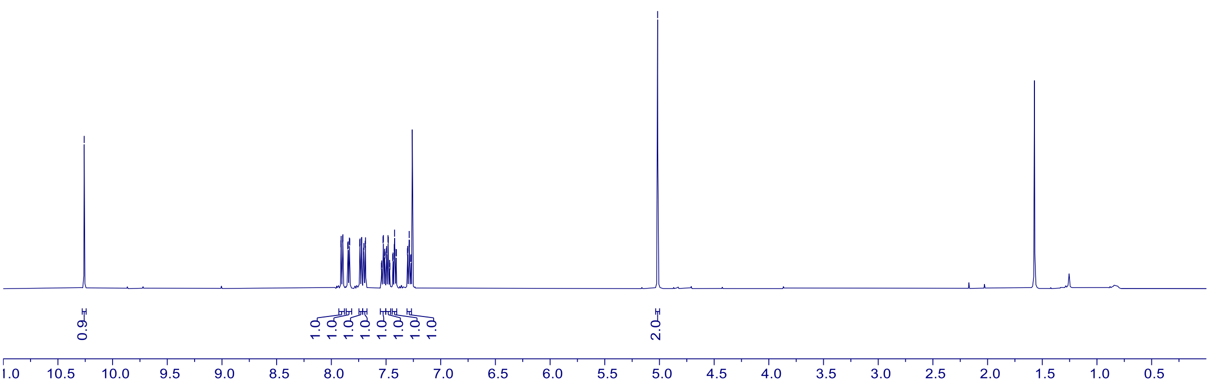

37 – ^{13}C NMR (126 MHz, $CDCl_3$)

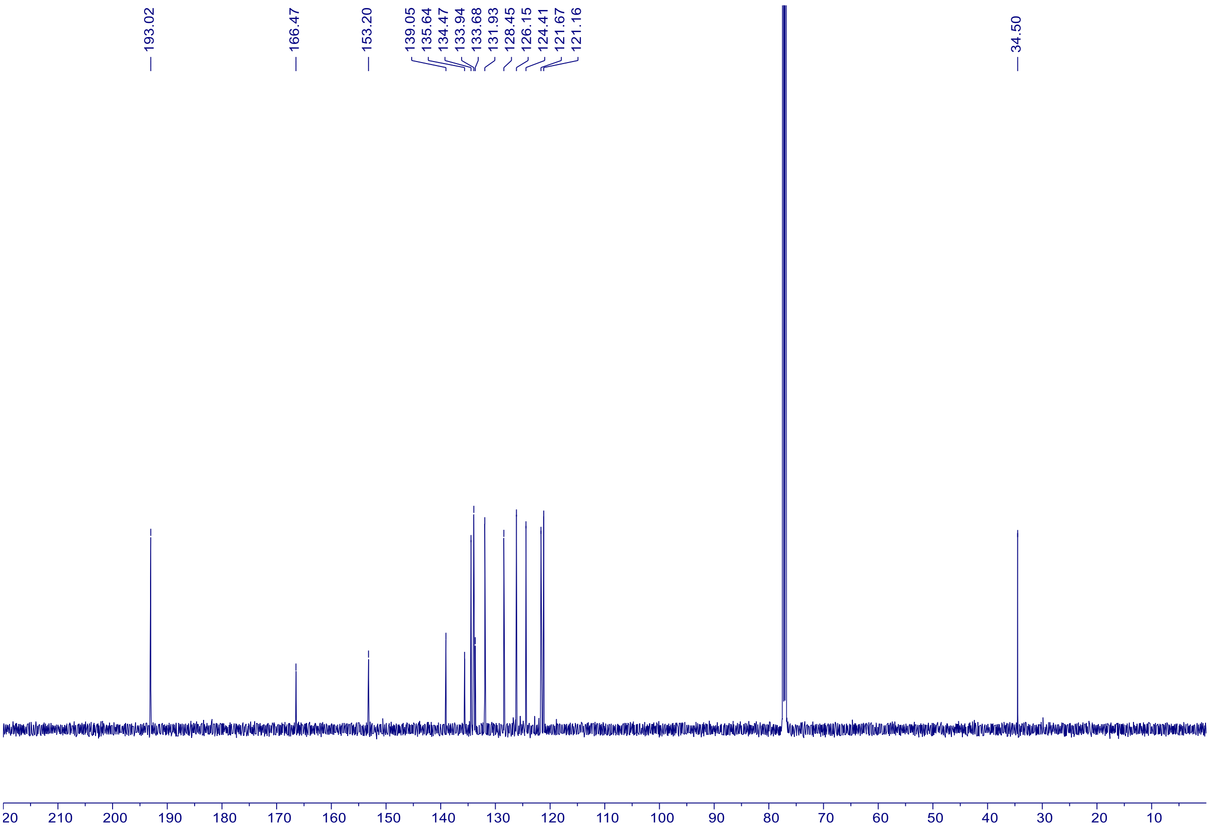

40 – ^{1}H NMR (500 MHz, CDCl$_3$)

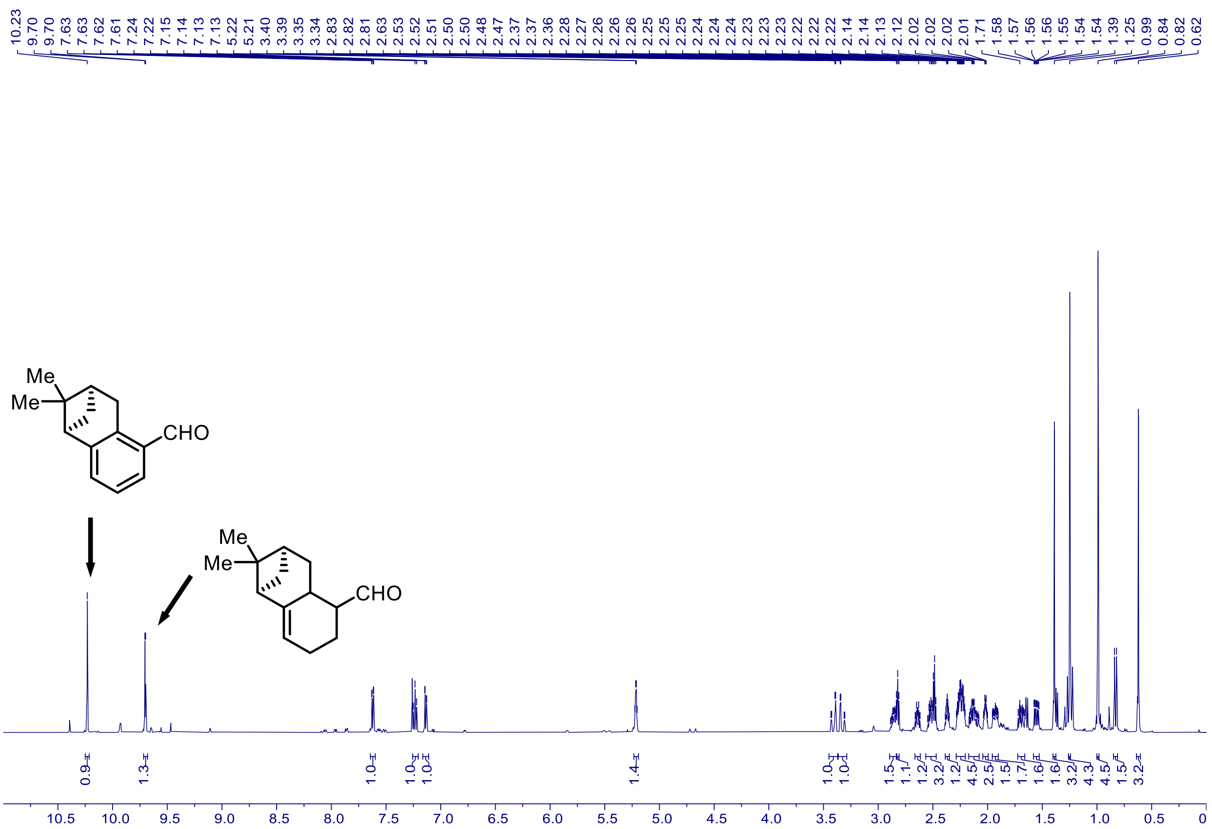

40 – ^{13}C NMR (126 MHz, CDCl$_3$)

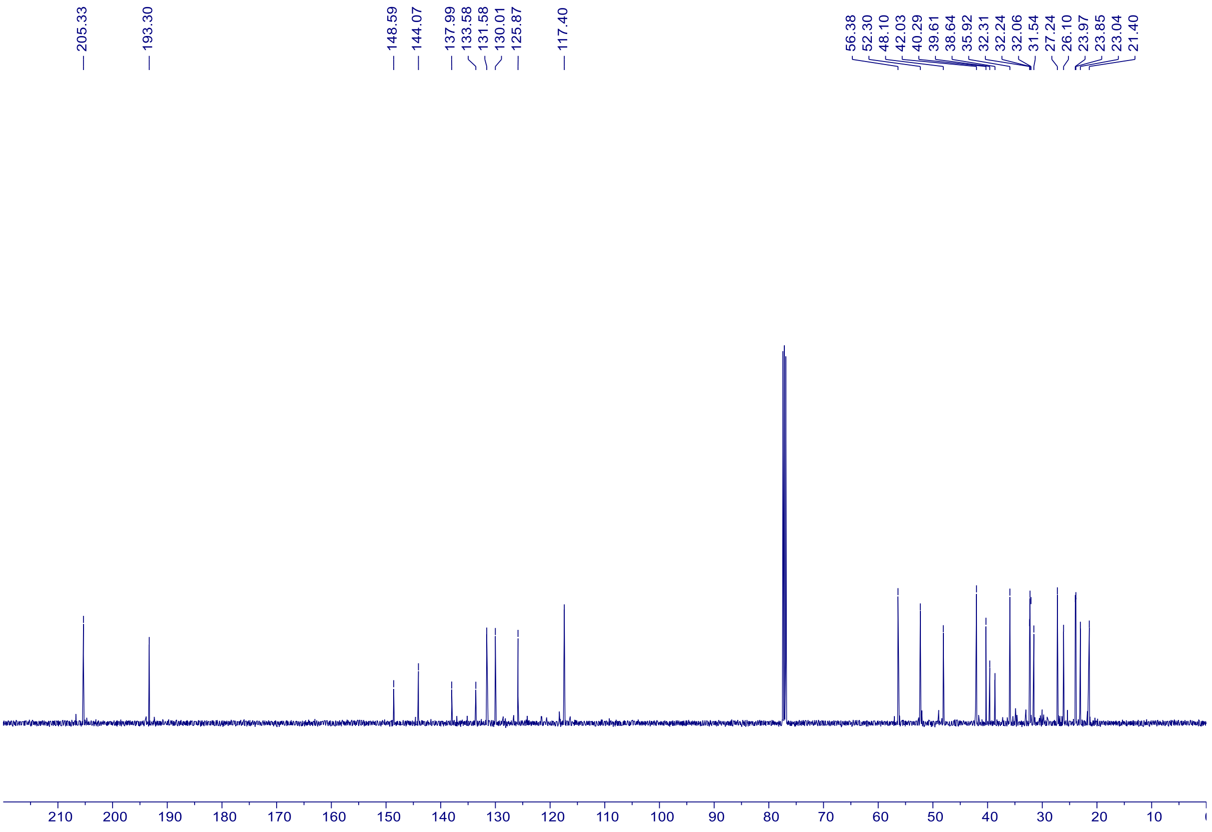

43 – ^{1}H NMR (400 MHz, $CDCl_3$)

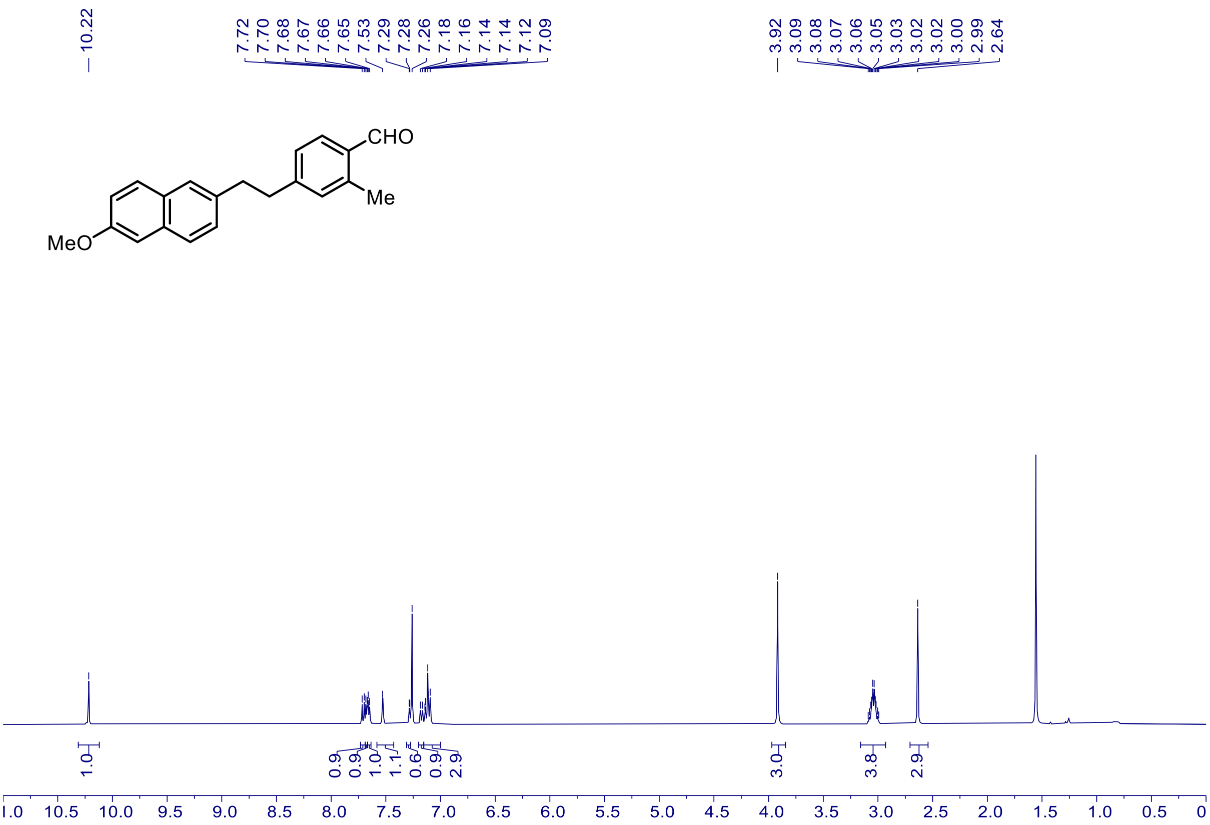

43 – ^{13}C NMR (126 MHz, $CDCl_3$)

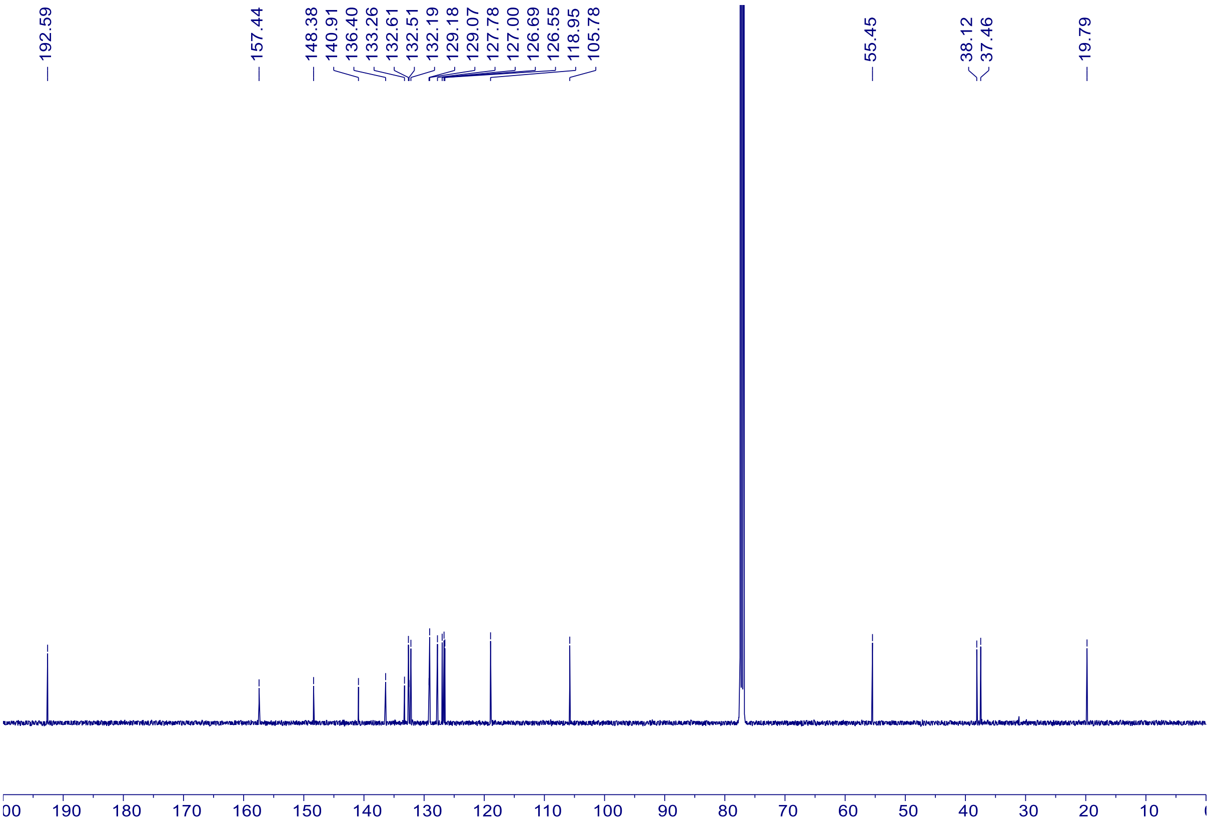

46 – ^{1}H NMR (400 MHz, $CDCl_3$)

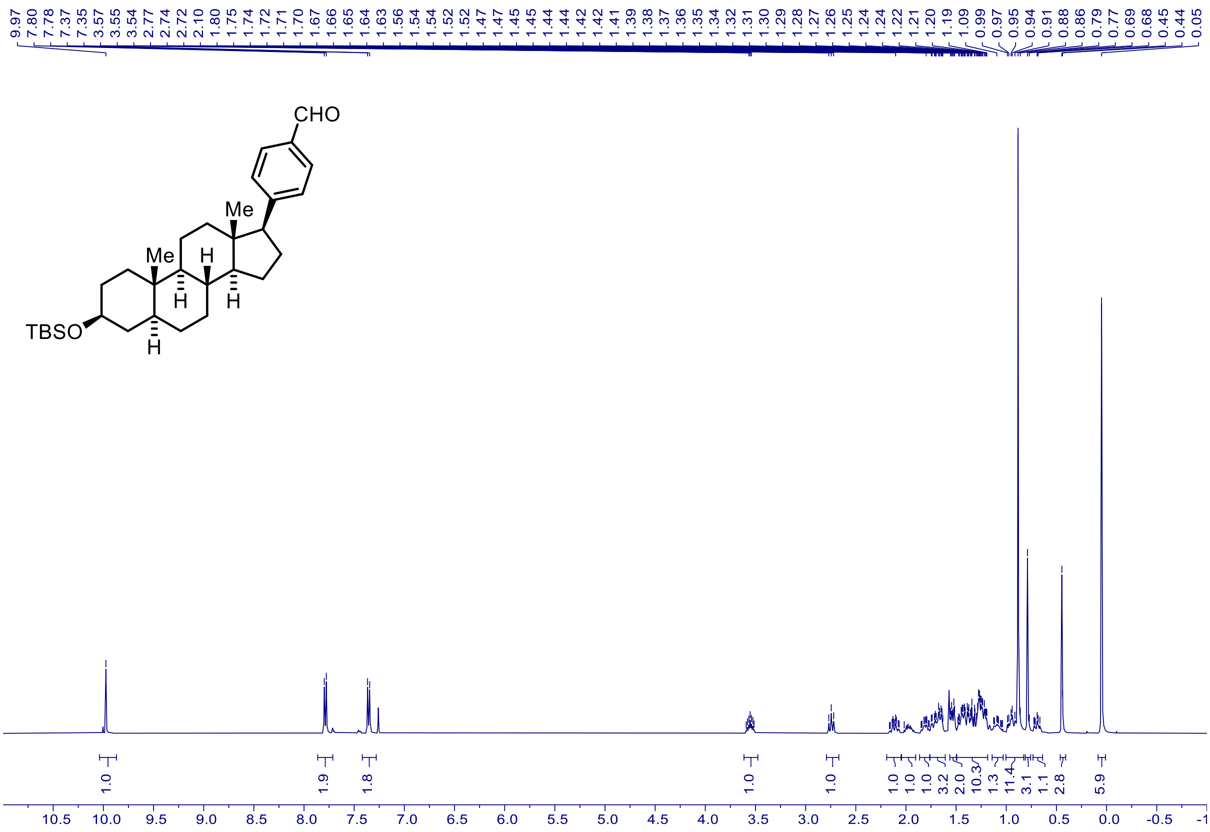

46 – ^{13}C NMR (101 MHz, $CDCl_3$)

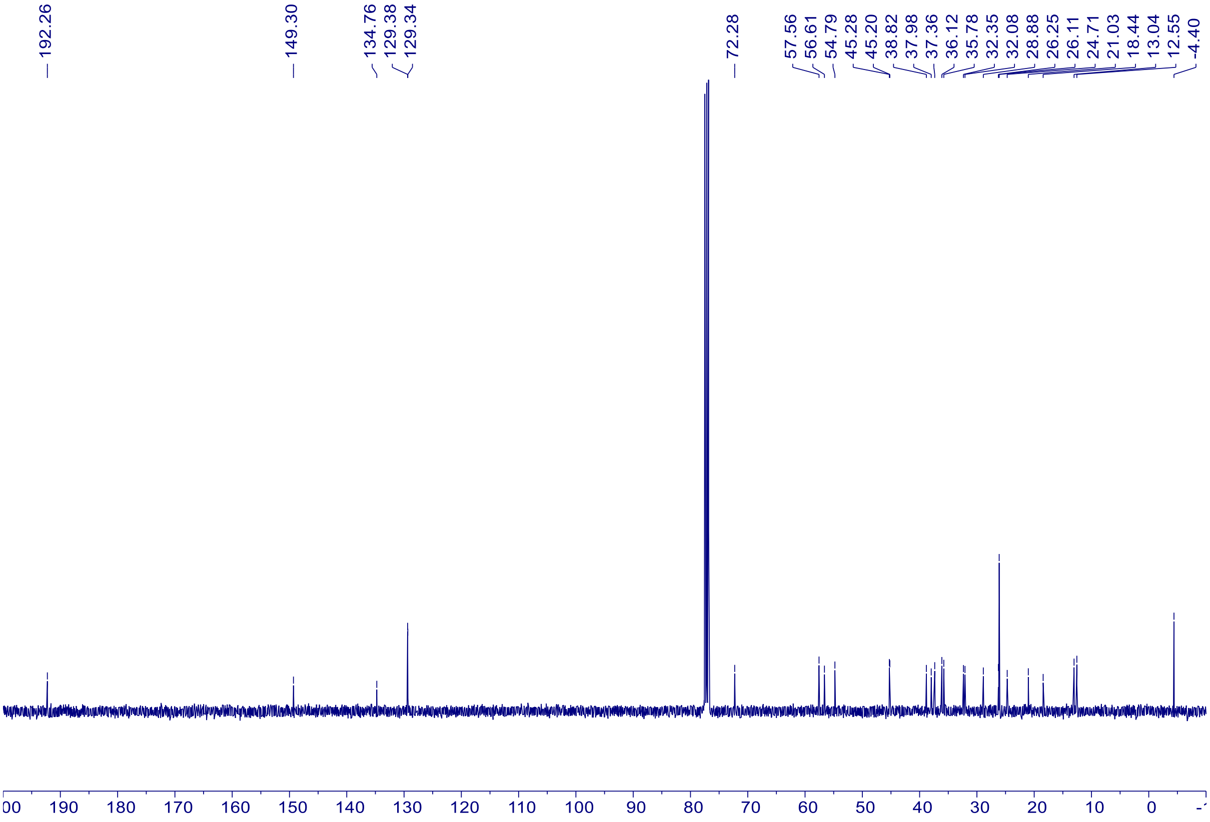

9 References

[1] E. Taarning, R. Madsen, *Chemistry – A European Journal* **2008**, *14*, 5638-5644.
[2] H. Gotoh, Y. Hayashi, *Organic Letters* **2007**, *9*, 2859-2862.
[3] F. K. Cheung, A. M. Hayes, D. J. Morris, M. Wills, *Organic & Biomolecular Chemistry* **2007**, *5*, 1093-1103.
[4] X.-H. Hu, X.-F. Yang, T.-P. Loh, *Angewandte Chemie International Edition* **2015**, *54*, 15535-15539.
[5] A. Köpfer, B. Sam, B. Breit, M. J. Krische, *Chemical Science* **2013**, *4*, 1876-1880.
[6] C. Li, K. Shin, R. Y. Liu, S. L. Buchwald, *Angewandte Chemie International Edition* **2019**, *58*, 17074-17080.
[7] K. D. Nguyen, D. Herkommer, M. J. Krische, *Journal of the American Chemical Society* **2016**, *138*, 14210-14213.
[8] D. Fiorito, S. Folliet, Y. Liu, C. Mazet, *ACS Catalysis* **2018**, *8*, 1392-1398.
[9] D. Akalay, G. Dürner, J. W. Bats, M. Bolte, M. W. Göbel, *The Journal of Organic Chemistry* **2007**, *72*, 5618-5624.
[10] A. Misale, S. Niyomchon, M. Luparia, N. Maulide, *Angewandte Chemie International Edition* **2014**, *53*, 7068-7073.
[11] F. K. Sheffy, J. K. Stille, *Journal of the American Chemical Society* **1983**, *105*, 7173-7175.
[12] S. M. Sarkar, Y. Uozumi, Y. M. A. Yamada, *Angewandte Chemie International Edition* **2011**, *50*, 9437-9441.
[13] J. S. Alford, N. C. Abascal, C. R. Shugrue, S. M. Colvin, D. K. Romney, S. J. Miller, *ACS Central Science* **2016**, *2*, 733-739.
[14] C. Mayato, R. L. Dorta, J. M. Palazón, J. T. Vázquez, *Carbohydrate Research* **2012**, *352*, 101-108.
[15] M. Nakatsuka, J. A. Ragan, T. Sammakia, D. B. Smith, D. E. Uehling, S. L. Schreiber, *Journal of the American Chemical Society* **1990**, *112*, 5583-5601.
[16] T. Gündemir-Durmaz, F. Schmid, Y. El Baz, A. Häusser, C. Schneider, U. Bilitewski, G. Rauhut, D. Garnier, A. Baro, S. Laschat, *Organic & Biomolecular Chemistry* **2016**, *14*, 8261-8269.
[17] S. Nagasawa, Y. Sasano, Y. Iwabuchi, *Angewandte Chemie International Edition* **2016**, *55*, 13189-13194.
[18] A. K. Ghosh, A. Sarkar, *European Journal of Organic Chemistry* **2016**, *2016*, 6001-6009.
[19] J. Y. Wu, B. Moreau, T. Ritter, *Journal of the American Chemical Society* **2009**, *131*, 12915-12917.
[20] B. B. Shingate, B. G. Hazra, D. B. Salunke, V. S. Pore, F. Shirazi, M. V. Deshpande, *European Journal of Medicinal Chemistry* **2011**, *46*, 3681-3689.
[21] A. Schmidt, G. Hilt, *Organic Letters* **2013**, *15*, 2708-2711.
[22] T. Niu, S. Chen, M. Hong, T. Zhang, J. Chen, X. Dong, B. Ni, *Green Chemistry* **2020**, *22*, 5042-5049.
[23] P. Hu, M. Tan, L. Cheng, H. Zhao, R. Feng, W.-J. Gu, W. Han, *Nature Communications* **2019**, *10*, 2425.
[24] S. Rej, N. Chatani, *Journal of the American Chemical Society* **2021**, *143*, 2920-2929.
[25] T. M. Barhoumi-Slimi, M. Ourévitch, *Journal of the Iranian Chemical Society* **2018**, *15*, 629-636.
[26] G.-F. Zha, W.-Y. Fang, J. Leng, H.-L. Qin, *Advanced Synthesis & Catalysis* **2019**, *361*, 2262-2267.
[27] I. Ramos-Tomillero, M. Paradís-Bas, I. De Pinho Ribeiro Moreira, J. M. Bofill, E. Nicolás, F. Albericio, *Molecules* **2015**, *20*.

[28] X. Li, B. Fu, Q. Zhang, X. Yuan, Q. Zhang, T. Xiong, Q. Zhang, *Angewandte Chemie International Edition* **2020**, *59*, 23056-23060.
[29] R. A. Fernandes, S. P. Gholap, S. V. Mulay, *RSC Advances* **2014**, *4*, 16438-16443.
[30] S. Kim, Y. Kim, H. Jin, M. H. Park, Y. Kim, K. M. Lee, M. Kim, *Advanced Synthesis & Catalysis* **2019**, *361*, 1259-1264.
[31] M. García-Díaz, D. Sánchez-García, J. Soriano, M. L. Sagristà, M. Mora, Á. Villanueva, J. C. Stockert, M. Cañete, S. Nonell, *MedChemComm* **2011**, *2*, 616-619.
[32] K. Chojnacka, S. Santoro, R. Awartani, N. G. J. Richards, F. Himo, A. Aponick, *Organic & Biomolecular Chemistry* **2011**, *9*, 5350-5353.
[33] J. Chen, C. Bai, X. Tong, D. Liu, Y.-S. Bao, *RSC Advances* **2020**, *10*, 12192-12196.
[34] N. Kise, S. Isemoto, T. Sakurai, *The Journal of Organic Chemistry* **2011**, *76*, 9856-9860.
[35] C. B. Kelly, J. M. Ovian, R. M. Cywar, T. R. Gosselin, R. J. Wiles, N. E. Leadbeater, *Organic & Biomolecular Chemistry* **2015**, *13*, 4255-4259.
[36] V. V. Pavlishchuk, A. W. Addison, *Inorganica Chimica Acta* **2000**, *298*, 97-102.
[37] H. G. Roth, N. A. Romero, D. A. Nicewicz, *Synlett* **2016**, *27*, 714.
[38] C. K. Prier, D. A. Rankic, D. W. C. MacMillan, *Chem. Rev.* **2013**, *113*, 5322-5363.
[39] M. A. Cismenia, T. P. Yoon, *Chem. Sci.* **2015**, *6*, 5426.
